# A survey of the spider family Nesticidae (Arachnida, Araneae) in Asia and Madagascar, with the description of forty-three new species

**DOI:** 10.3897/zookeys.627.8629

**Published:** 2016-10-27

**Authors:** Yucheng Lin, Francesco Ballarin, Shuqiang Li

**Affiliations:** 1 Key Laboratory of Bio-resources and College of Eco-environment (Ministry of Education), College of Life Sciences, Sichuan University, Chengdu, Sichuan 610064, China; 2 Southeast Asia Biodiversity Research Institute, Chinese Academy of Sciences, Menglun, Mengla, Yunnan 666303, China; 3 Institute of Zoology, Chinese Academy of Sciences, Beijing 100101, China

**Keywords:** Nesticids, taxonomy, new taxa, cave, leaf litter

## Abstract

Forty-three new species of Nesticidae are described from China, Indonesia, Philippines, Singapore, Thailand, Vietnam and Madagascar, and two new junior synonyms are suggested. A new genus, *Speleoticus* **gen. n.**, is described with *Nesticus navicellatus* Liu & Li, 2013 as the type species, and four species are transferfed from *Nesticus*, i.e., *Speleoticus globosus* (Liu & Li, 2013), **comb. n.**, *Speleoticus libo* (Chen & Zhu, 2005), **comb. n.**, *Speleoticus navicellatus* (Liu & Li, 2015), **comb. n.** and *Speleoticus uenoi* (Yaginuma, 1972), **comb. n.** The new species described in this paper belong to four genera and are: *Hamus cornutus* **sp. n.** (♂♀), *Hamus kangdingensis* **sp. n.** (♂), *Hamus luzon* **sp. n.** (♀), *Hamus mangunensis* **sp. n.** (♂), *Nescina kohi* **sp. n.** (♂♀), *Nesticella baiseensis* **sp. n.** (♂♀), *Nesticella baobab* **sp. n.** (♂), *Nesticella caeca* **sp. n.** (♂♀), *Nesticella chongqing* **sp. n.** (♀), *Nesticella dazhuangensis* **sp. n.** (♂♀), *Nesticella fuliangensis* **sp. n.** (♂♀), *Nesticella gazuida* **sp. n.** (♀), *Nesticella gongshanensis* **sp. n.** (♀), *Nesticella griswoldi* **sp. n.** (♂♀), *Nesticella hongheensis* **sp. n.** (♂♀), *Nesticella huomachongensis* **sp. n.** (♂♀), *Nesticella jingpo* **sp. n.** (♀), *Nesticella kaohsiungensis* **sp. n.** (♂♀), *Nesticella lisu* **sp. n.** (♂♀), *Nesticella liuzhaiensis* **sp. n.** (♀), *Nesticella nandanensis* **sp. n.** (♂♀), *Nesticella phami* **sp. n.** (♂♀), *Nesticella potala* **sp. n.** (♀), *Nesticella qiaoqiensis* **sp. n.** (♀), *Nesticella qiongensis* **sp. n.** (♂♀), *Nesticella robusta* **sp. n.** (♂♀), *Nesticella rongtangensis* **sp. n.** (♂), *Nesticella sanchaheensis* **sp. n.** (♂♀), *Nesticella sulawesi* **sp. n.** (♀), *Nesticella sumatrana* **sp. n.** (♂), *Nesticella tibetana* **sp. n.** (♂♀), *Nesticella vanlang* **sp. n.** (♀), *Nesticella wanzaiensis* **sp. n.** (♂♀), *Nesticella xiongmao* **sp. n.** (♂♀), *Nesticella xixia* **sp. n.** (♂♀), *Nesticella yanbeiensis* **sp. n.** (♂♀), *Nesticella yao* **sp. n.** (♀), *Nesticella zhiyuani* **sp. n.** (♂♀), *Pseudonesticus dafangensis* **sp. n.** (♂♀), *Pseudonesticus miao* **sp. n.** (♂♀), *Pseudonesticus spinosus* **sp. n.** (♂♀), *Pseudonesticus wumengensis* **sp. n.** (♀), *Pseudonesticus ziyunensis* **sp. n.** (♂♀). *Nesticella inthanoni* (Lehtinen & Saaristo, 1980), **syn. n.** is synonymised with *Nesticella mollicula* (Thorell, 1898); *Nesticella taiwan* Tso & Yoshida, 2000, **syn. n.** is synonymised with *Nesticella odonta* (Chen, 1984). The female of *Nesticella connectens* Wunderlich, 1995, so far unknown, is described and recorded from Thailand. Nesticidae are reported from Madagascar for the first time. *Nesticella nepalensis* (Hubert, 1973) is recorded for the first time from China. Types of *Nesticella odonta* (Chen, 1984), *Nesticella songi* Chen & Zhu, 2004 and *Nesticella yui* Wunderlich & Song, 1995 are re-examined and photographed. The entire genus *Nesticella* is reviewed, and four species groups are recognised. DNA barcodes of the new species are obtained to confirm their correct identifications.

## Introduction

Nesticidae is a small family of spiders with an almost worldwide distribution, being absent only in Siberia, Central Asia, Northern and Southern Africa and at high latitudes. So far, 233 extant species belonging to 13 genera (World Spider Catalog 2016) and 12 fossil species belonging to four extinct genera (Dunlop et al. 2016) are considered in this family. The majority of nesticids occur in temperate areas of the Holarctic realm where they are mainly restricted to cave-like environments and are medium-sized and long-legged with different levels of adaptation to the troglophylic life. By contrast, nesticids living in tropical or subtropical areas of the Afrotropic, Australian, Neotropic and Oriental realm are mostly characterized by being smaller than their Holarctic counterparts and they have shorter legs and only a minor bond to cave environments. They frequently occur outside caves in forest litter, on grass and under stones (Lehtinen and Saaristo 1980).

Traditionally the family Nesticidae has been considered rather uniform since established by Simon in 1894, with only one or very few genera included. Only in 1980 Lehtinen & Saaristo made a preliminary worldwide revision, grouping them in 10 genera and two tribes, Nesticini and Nesticellini, with the exception of the Nearctic and Neotropical genera *Eidmannella* Roewer, 1935 and *Gaucelmus* Keyserling, 1884 which were considered separately. The tribe Nesticini included the “long-legged” species restricted to caves and consisted of the genera *Nesticus* Thorell, 1869 from Europe and the Americas, *Cyclocarcina* Komatsu, 1942 endemic to Japan and *Carpathonesticus* Lehtinen & Saaristo, 1980 and *Typhlonesticus* Kulczyński, 1914 distributed in the Carpathian Mountains, Caucasus and around the Mediterranean Basin. The tribe Nesticellini contained all the so called “short-legged” nesticids distributed in the Oriental-Australian region that mostly live in leaf litter of tropical forests. According to the authors, this tribe consists of two main genera, *Howaia* Lehtinen & Saaristo, 1980 and *Nesticella* Lehtinen & Saaristo, 1980 separated by palpal morphological characters; these genera were subsequently synonymized by Wunderlich (1986). On the contrary, Marusik and Guseinov (2003) considered the diagnosis provided by Lehtinen and Saaristo reliable, supporting the split into two distinct genera and rejecting the synonymization by Wunderlich (1986). The systematics of *Nesticella* remains unsolved and is a cause of debate.

Since the work of Lehtinen and Saaristo (1980), Nesticide publications have been small-scale and separate rather than large-scale taxonomic surveys. The contrasting qualitative levels of descriptions and illustrations, sometimes only basic or very schematic, made further progress on the study of this family more complex. Therefore, new species discovered in Asia or Africa, including the majority of the relatively well-studied Japanese nesticids, were all assigned to the genera *Nesticella* and *Nesticus*. Only recently has the increasing interest in spider research in China and Southeast Asia allowed a better knowledge of local species systematics and ecology. Thus, the genus *Pseudonesticus* was created by Liu and Li (2013a) to accommodate *Pseudonesticus clavatus* Liu & Li, 2013, a new blind troglobitic species from Yunnan, China. Three other monotypic genera, *Hamus*, *Nescina* and *Wraios* were erected by Ballarin and Li (2015) to include three Chinese species (*Hamus bowoensis*, *Nescina minuta* and *Wraios longiembolus*) from Tibet and Yunnan that have peculiar morphological characters. Moreover, a recent study by Zhang and Li (2013) on the Chinese *Nesticella* revealed that the species of the Yunnan-Guizhou Plateau represent an ancient lineage with a strong bond to the subterranean environment despite the absence of morphological adaptations. According to the authors, this troglobitic lifestyle probably evolved through multiple, independent colonization events during the Pleistocene.

In the last 15 years several collecting trips were carried out by Chinese, American and European arachnologists in Madagascar, China and Southeast Asia including Laos, Myanmar, Vietnam, Thailand, Singapore, Indonesia and Philippines. This effort allowed the collection many spiders, including numerous specimens of the family Nesticidae and was followed by the preliminary description of some new species (Liu and Li 2013a, 2013b; Grall and Jäger 2016; Li and Lin 2016). However, deeper morphological and molecular studies of this material revealed a further, extraordinary species diversity in South China and in the adjacent areas that was previously widely underestimated.

The main aim of this paper is to report forty-three new species belonging to the genera *Hamus*, *Nescina*, *Nesticella* and *Pseudonesticus*. The new genus, *Speleoticus* gen. n., which includes the Japanese species *Speleoticus uenoi* (Yaginuma, 1972), and the remaining Chinese species previously placed in *Nesticus*, is established based on palpal and epigynal morphology.

## Material and methods

Specimens used in this study were collected by hand or sifting leaf litter in China, Vietnam, Thailand, Philippines, Indonesia and Madagascar and immediately preserved in a 95% ethanol solution. All samples were examined using a Leica M205 C stereomicroscope and photographed with an Olympus c7070 wide zoom digital camera (7.1 megapixels). Images were mounted using Helicon Focus 3.10.3 software (Khmelik et al. 2006) and Combine ZP image stacking software. Male palps and epigynes were examined and photographed after dissection. Epigynes were treated in lactic acid before being embedded in Arabic gum to take the photos of the vulva. To reveal the course of the spermatic ducts, in some cases male palps, were also cleared using lactic acid and subsequently mounted in Hoyer’s Solution. The left palp was photographed and described. All measurements are in millimeters, with leg measurements given in the following sequence: total length (femur, patella, tibia, metatarsus, and tarsus).

A partial fragment (625 bp) of the mitochondrial gene cytochrome c oxidase subunit I (COI) was amplified and sequenced to obtain the genetic distances between morphologically similar species and to confirm identifications and the sex pairing accuracy. Additionally, sequences of *Hamus bowoensis* Ballarin & Li, 2015, *Nescina minuta* Ballarin & Li, 2015, *Nesticella connectens* Wunderlich, 1995 and *Nesticella odonta* (Chen, 1984) were included; however, we were unable to obtain good extractions from *Hamus kangdingensis* sp. n., *Hamus mangunensis* sp. n., *Nesticella lisu* sp. n., *Nesticella xiongmao* sp. n. and *Nesticella rongtangensis* sp. n., and these were consequently excluded.

The primers used are: LCO1490 (5’-GGTCAACAAATCATCATAAAGATATTGG-3’) and CHR2 (5’-GGATGGCCAAAAAATCAAAATAAATG-3’). Raw sequences were edited and assembled using BioEdit v.7.2.5 (Hall 1999), and uncorrected pairwise distances between sequences were calculated using MEGA v.6.0 (Tamura et al. 2013) and are shown in Appendix B. All sequences were deposited in GenBank, and the accession numbers are provided in Table 1.

**Table 1.** Voucher specimen information.

Species	GenBank accession number	Collection localities
*Hamus bowoensis* Ballarin & Li, 2015	KX866931	China, Tibet, Bowo Co., Kaduo Village
*Hamus cornutus* sp. n.	KX866932	China, Guangxi, Pingxiang Co., Sanzhishan Cave
*Hamus luzon* sp. n.	KX866933	Philippines, Luzon Island, Mountain, Bontoc Town, rain forest
*Nescina kohi* sp. n.	KX866934	Singapore, Central Catchment Nature Reserve
*Nescina minuta* Ballarin & Li, 2015	KX866935	China, Yunnan, Xishuangbanna, Mengla Co., Xiaolongha Village, Gougu forest
*Nesticella baiseensis* sp. n.	KX866936	China, Guangxi, Lingyun Co., Shuiyuan Cave
*Nesticella baobab* sp. n.	KX866937	Madagascar, Fianarantsoa, Parc National Ranomafana, Vohiparara
*Nesticella caeca* sp. n.	KX866938	China, Guizhou, Tianzhu Co., Liuhe Village, Jinshan Cave
*Nesticella chongqing* sp. n.	KX866939	China, Chongqing City, Beibei Dist., Dajiang Village, Xiaofang Cave
*Nesticella connectens* Wunderlich, 1995	KX866940	Thailand, Satun, Trang Dist., Beating Cave
*Nesticella dazhuangensis* sp. n.	KX866941	China, Yunnan, Tengchong Co., Dazhuang Village
*Nesticella fuliangensis* sp. n.	KX866942	China, Jiangxi, Fuliang Co., Zhuxian Cave
*Nesticella gazuida* sp. n.	KX866943	China, Guizhou, Kaili City, Gazuida Cave
*Nesticella gongshanensis* sp. n.	KX866944	China, Yunnan, Gongshan Co., Langdang Village
*Nesticella griswoldi* sp. n.	KX866945	Madagascar, Toliara, Forest Classee Tsitongambarika, Cascade hiking trails
*Nesticella hongheensis* sp. n.	KX866946	China, Yunnan, Pingbian Co.
*Nesticella huomachongensis* sp. n.	KX866947	China, Hunan Province, Huaihua City, Chenxi Co., Huomachong Town, Yanzi Cave
*Nesticella jingpo* sp. n.	KX866948	China, Yunnan, Tengchong Co., Gaoligongshan Mountain National Park
*Nesticella kaohsiungensis* sp. n.	KX866949	China, Taiwan, Nantou Co., Hui Sun
*Nesticella liuzhaiensis* sp. n.	KX866950	China, Guangxi, Nandan Co., Longli Village, near the Dixia River
*Nesticella nandanensis* sp. n.	KX866951	China, Guangxi, Nandan Co., Encun Village, Encun Cave
*Nesticella odonta* (Chen, 1984)	KX866952	China, Jiangxi, Shangli Co., Zhanshan Village, Xiongxin Cave
*Nesticella phami* sp. n.	KX866953	Vietnam, Quang Ninh, Phong Nha Ke bang National Park, Sung Sot Cave
*Nesticella potala* sp. n.	KX866954	China, Tibet, Gyirong Co., Zalong Village
*Nesticella qiaoqiensis* sp. n.	KX866955	China, Sichuan, Baoxing Co., Zeyin Village
*Nesticella qiongensis* sp. n.	KX866956	China, Hainan, Diaoluoshan Mountain National Nature Reserve, Diaoluoshan Holiday Village
*Nesticella robusta* sp. n.	KX866957	China, Hunan, Yuanling Co., Qixian Cave
*Nesticella sanchaheensis* sp. n.	KX866958	China, Guizhou, Libo Co., Sanchahe Village, Sanchahe Cave
*Nesticella sulawesi* sp. n.	KX866959	Indonesia, South Sulawesi, Maros City, Cenrana Village
*Nesticella sumatrana* sp. n.	KX866960	Indonesia, West Sumatra, Payakumbuh City, Koto Tiggi Village, a cave without name, close to Imam Bonjol Cave
*Nesticella tibetana* sp. n.	KX866961	China, Tibet, Bowo Co., Yigong Town
*Nesticella vanlang* sp. n.	KX866962	Vietnam, Ninh Thuan, Nui Chua National Park
*Nesticella wanzaiensis* sp. n.	KX866963	China, Jiangxi, Wanzai Co., Dongkou Village, Zhushan Cave
*Nesticella xixia* sp. n.	KX866964	China, Henan, Xixia Co., Baihe Village, Yunhuabianfu Cave
*Nesticella yanbeiensis* sp. n.	KX866965	China, Guangxi, Lingchuan Co., Yanbei Village, Yanbei Cave
*Nesticella yao* sp. n.	KX866966	China, Guangxi, Gongcheng Co., Songlin Village, Houyan Cave
*Nesticella zhiyuani* sp. n.	KX866967	Indonesia, West Sumatra, Payakumbuh City, Koto Tiggi Village, a cave without name, close to Imam Bonjol Cave
*Pseudonesticus dafangensis* sp. n.	KX866970	China, Guizhou, Dafang Co., Sanhe Village, Yelaoda Cave
*Pseudonesticus miao* sp. n.	KX866968	China, Guizhou, Anshun City, Xixiu Dist., Xiaguantun Village, Duofan Cave
*Pseudonesticus spinosus* sp. n.	KX866969	China, Guizhou, Suiyang Co., Guihua Village, Mahuang Cave
*Pseudonesticus wumengensis* sp. n.	KX866971	China, Guizhou, Hezhang Co., Gaoyan Village, Tanjiayan Cave
*Pseudonesticus ziyunensis* sp. n.	KX866972	China, Guizhou, Ziyun Co., Mt. Wufeng, Wufeng Cave

Abbreviations used in the text or figures are given in Table 2. References to figures in the cited papers are listed in lowercase (fig. or figs); figures from this paper are noted with an initial capital (Fig. or Figs). All molecular vouchers are preserved in the Institute of Zoology, Chinese Academy of Sciences (IZCAS) in Beijing. Institutions hosting the type material and other specimens used in this work is reported in Table 2.

**Table 2.** List of abbreviations used in the text or figures.

**Male palp**	
**Ac**	apex of the conductor
**C**	conductor
**Cp (I-III)**	processes of the conductor (I-III)
**Da**	dorsal apophysis of the paracymbium
**Dp (I-II)**	distal processes of the paracymbium (I-II)
**E**	embolus
**Es**	embolic spur
**Lf**	lateral cymbial furrow
**Tg (I-II)**	tegular apophysis (I-II)
**P**	paracymbium
**St**	subtegulum
**T**	tegulum
**Ta (I-II)**	terminal apophysis (I-II)
**Va (I-III)**	ventral apophysis of the paracymbium (I-III)
**Epigyne**	
**Co**	copulatory opening
**Ep**	epigynal plate
**Cd**	copulatory duct
**Fd**	fertilization duct
**S**	spermatheca
**Sp**	scape
**Vp**	vulval pocket
**Ocular area**	
**AER**	anterior eye row
**ALE**	anterior lateral eye
**AME**	anterior median eye
**MOA**	median ocular area
**PER**	posterior eye row
**PLE**	posterior lateral eye
**PME**	posterior median eye
**Institutions**	
**CASC**	California Academy of Sciences, San Francisco, USA
**IZCAS**	Institute of Zoology, Chinese Academy of Sciences, Beijing, China
**IBPN**	Institute for Biological Problems of the North of the Russian Academy of Sciences, Magadan, Russia
**LKCNHM**	Lee Kong Chian Natural History Museum, National University of Singapore
**MHBU**	Museum of Hebei University, Baoding, China

## Taxonomy

### Genus Hamus

Taxon classificationAnimaliaAraneaeNesticidae

Ballarin & Li, 2015

Hamus Ballarin & Li, 2015: 180.

#### Type species.

*Hamus bowoensis* Ballarin & Li, 2015 from Tibet, China.

#### Diagnosis.

*Hamus* is easily distinguished from all the other Nesticidae, with the exception of *Nescina*, by the following combination of characters: for the males, a short paracymbium ending in only a tiny, hooked process (Dp) (usually more developed and ramificated in most other Nesticidae), a remarkable, almost round and ventrally flat bulb, a long, laminar conductor (C) with a counterclockwise course around the bulb (both absent in all the other genera except *Nescina*) and a compact and hook-like process of the conductor (Cp-I). Females are distinguished by the considerably large, almost round spermathecae (S), smaller in other genera. Males of the genus *Hamus* are separated from those of *Nescina* by the larger size, the larger terminal apophysis II (Ta-II) and the massive hook-like process I of the conductor (Cp-I) (Cp-I is reduced and flat in *Nescina*). Females can be separated by the wider spermathecae (S) and the shorter and simpler fertilization ducts (Fd).

#### Description.

Total length: 1.48–1.87 (male), 2.00–2.15 (female). Carapace rounded in males, more ovate in females, yellow or pale yellow. Cephalic area slightly raised, with sparse setae. Eyes ALE>PME=PLE>AME. Cervical groove and fovea indistinct. Chelicera with three promarginal teeth and multiple retromarginal tiny denticles on the fang furrow. Legs uniformly yellow; in male of *Hamus bowoensis* a tuft of hairs present on the prolateral margin of femur I. Opisthosoma grey or light yellow as the carapace, with long setae.

Male palp: tibia short, wider than long. Cymbium wide with well-developed lateral furrow about 2/3 of the cymbial length. Paracymbium short, compact and simple, weakly sclerotized, with a tiny, hooked distal process, a lobed ventral apophysis and a flat, translucent dorsal apophysis. Bulb almost round and ventrally flat. Terminal apophysis well-developed and strongly sclerotized with one or two processes, process I elongate or reduced, sometimes absent, process II always present and hook shaped. Tegular apophysis absent. Conductor long and laminar, starting at the prolateral margin of the bulb and following the embolus on the prolateral side with a counterclockwise course. Two strongly sclerotized processes located at the base of the conductor; the first short and compact, hook-like, the other elongate leading the terminal part of the embolus to the center of the bulb. Embolus long and slender starting from the retrolateral side of the bulb and bordering the tegulum with a semicircular clockwise course.

Epigyne: posterior margin of the epigynal plate weakly sclerotized and straight. Copulatory openings near the epigynal posterior margin. Spermathecae and fertilization ducts visible through the transparent tegument. Fertilization ducts relatively long and simple, copulatory ducts short, ventrally oriented. Spermathecae close to each other, wide and almost round or pyriform.

#### Composition.

*Hamus bowoensis* Ballarin & Li, 2015, *Hamus cornutus* sp. n., *Hamus kangdingensis* sp. n., *Hamus luzon* sp. n., and *Hamus mangunensis* sp. n.

#### Distribution.

China (Guangxi, Sichuan, Tibet, Yunnan), Laos, Philippines (Luzon Island).

### Hamus bowoensis

Taxon classificationAnimaliaAraneaeNesticidae

Ballarin & Li, 2015

Hamus bowoensis Ballarin & Li, 2015: 180–183, figs 1–2 (♂♀).

#### Diagnosis.

**Figure 1.**
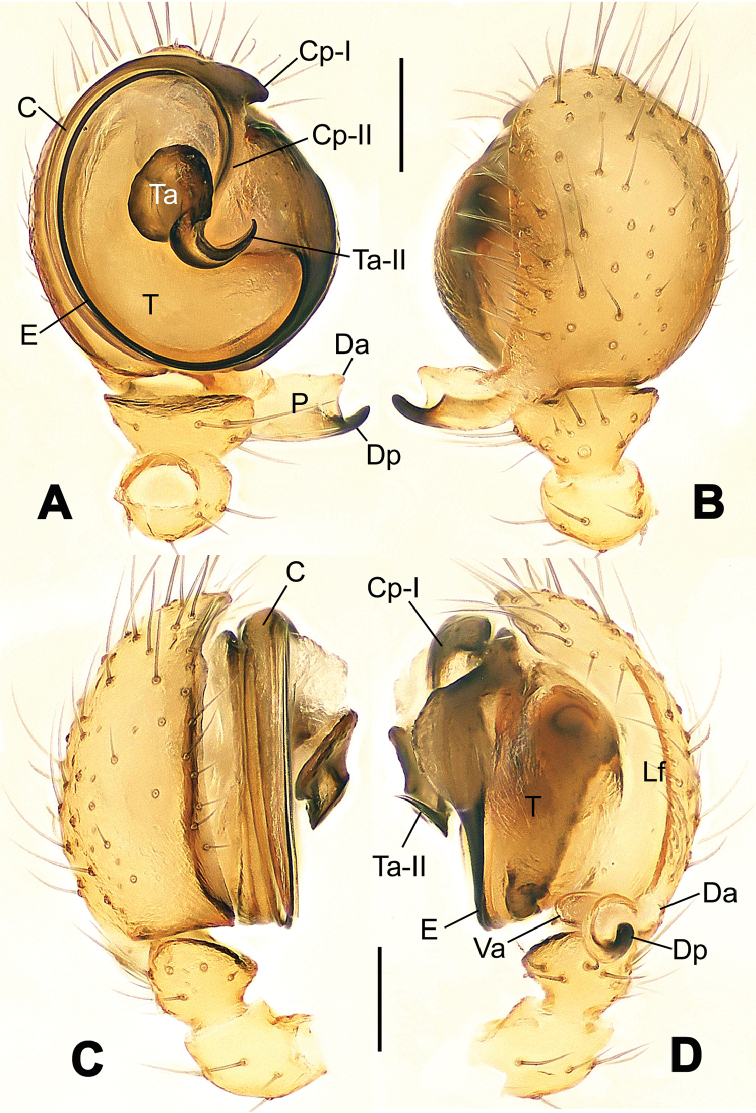
*Hamus cornutus* sp. n., holotype (male). **A** Palp, ventral view **B** Ditto, dorsal view **C** Ditto, prolateral view **D** Ditto, retrolateral view. Scale bars: 0.10 mm.

**Figure 2.**
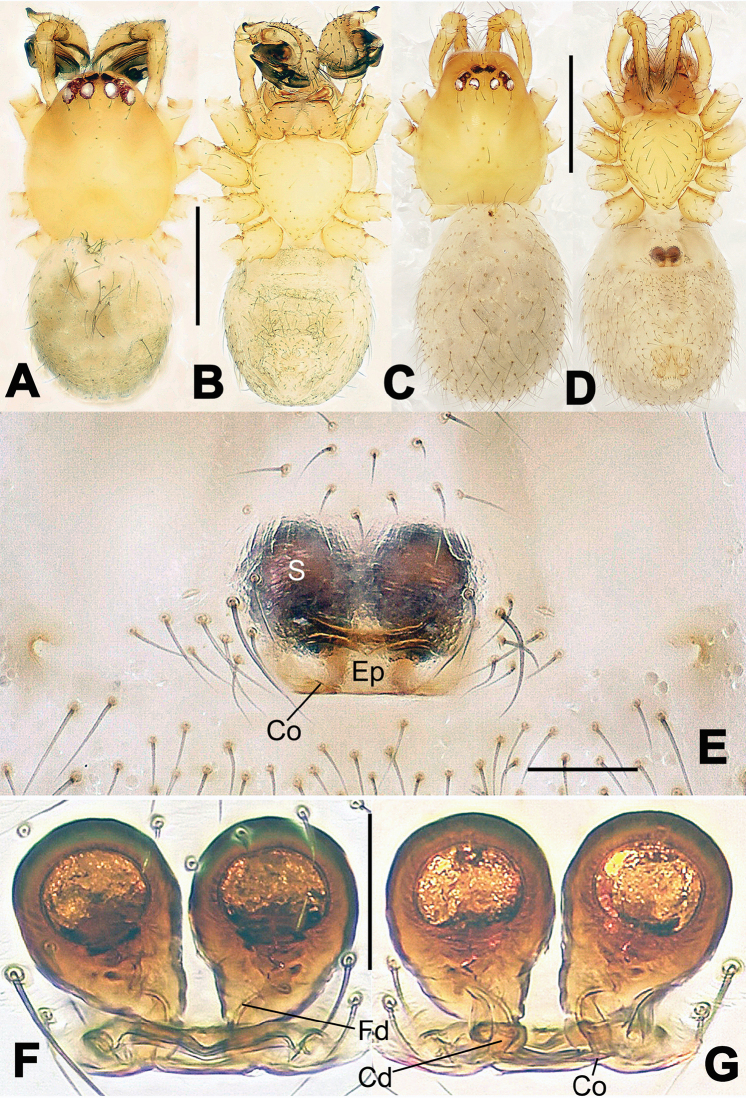
*Hamus cornutus* sp. n., holotype (male) and paratype (female). **A** Male habitus, dorsal view **B** Ditto, ventral view **C** Female habitus, dorsal view **D** Ditto, ventral view **E** Epigyne, ventral view **F** Vulva, ventral view **G** Vulva, dorsal view. Scale bars: **A–D** = 0.50 mm; **E–G** = 0.10 mm.

**Figure 3.**
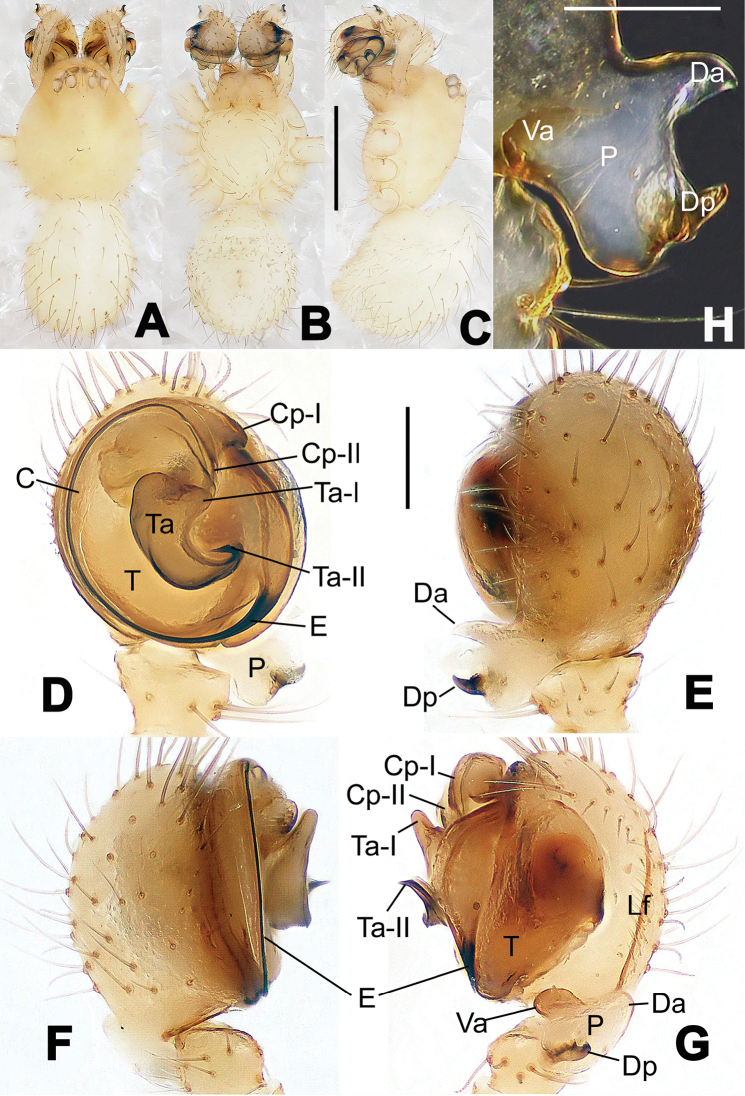
*Hamus kangdingensis* sp. n., holotype (male). **A** Habitus, dorsal view **B** Ditto, ventral view **C** Ditto, lateral view **D** Palp, ventral view **E** Ditto, dorsal view **F** Ditto, prolateral view **G** Ditto, retrolateral view **H** Paracymbium, ventral view. Scale bars: **A–C** = 0.50 mm; **D–H** = 0.10 mm.

**Figure 4.**
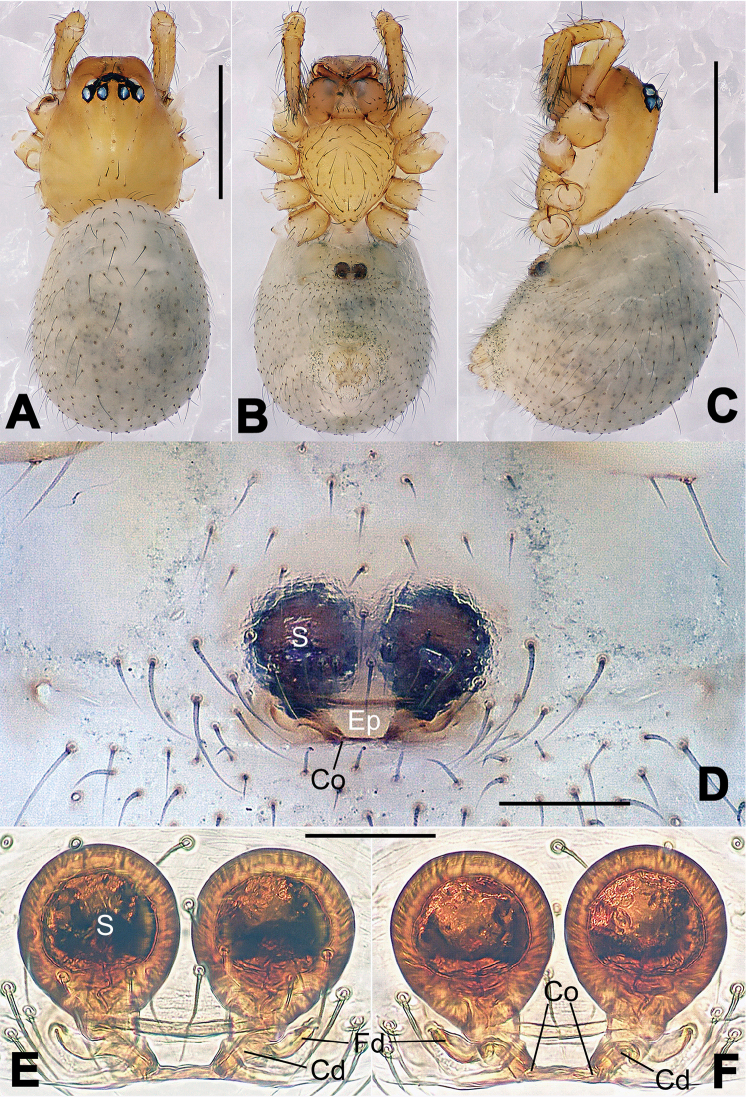
*Hamus luzon* sp. n., holotype (female). **A** Female habitus, dorsal view **B** Ditto, ventral view **C** Ditto, lateral view **D** Epigyne, ventral view **E** Vulva, ventral view **F** Vulva, dorsal view. Scale bars: **A–C** = 0.50 mm; **D–F** = 0.10 mm.

**Figure 5.**
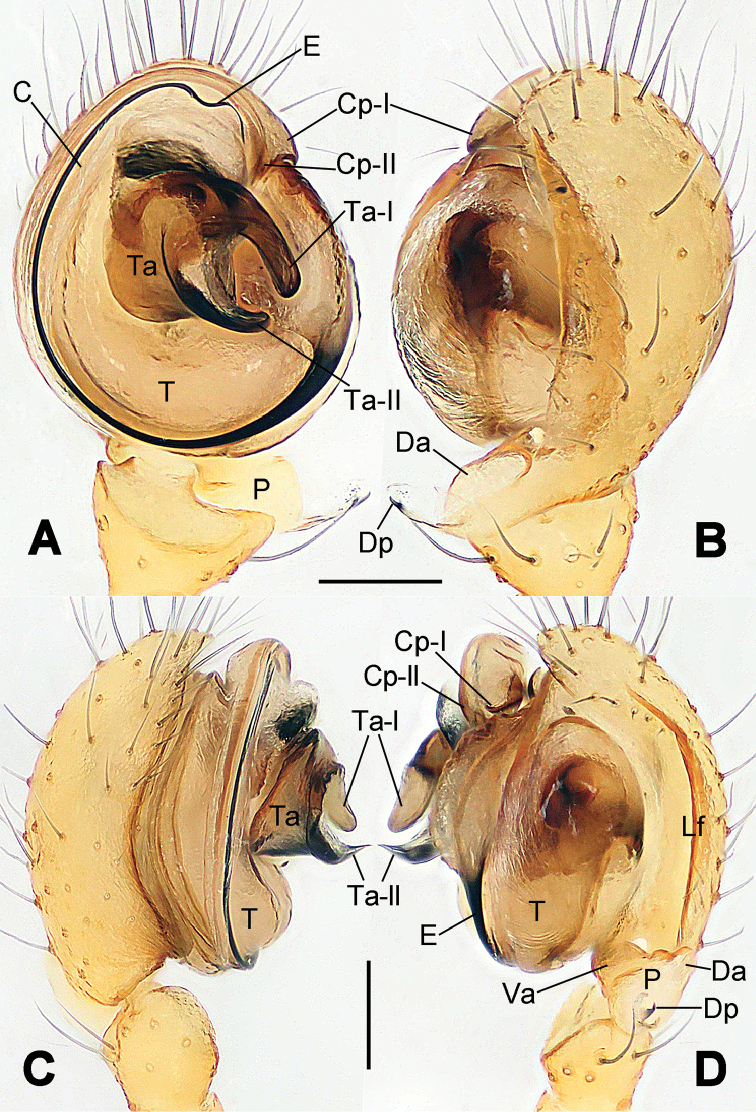
*Hamus mangunensis* sp. n., holotype (male). **A** Palp, ventral view **B** Ditto, dorsal view **C** Ditto, prolateral view **D** Ditto, retrolateral view. Scale bars: 0.10 mm.

Males can be separated from those of *Hamus cornutus* sp. n. and *Hamus kangdingensis* sp. n. by the tiny distal process of the paracymbium (Dp) (thicker in the other two species), by the presence of a well-developed terminal apophysis process I (Ta-I) and by the thicker apophysis process II with a less sharp point (Ta-II) (see Ballarin and Li 2015: 180, fig. 1B, D vs. Fig. 1A, D vs. Fig. 3D, G). They are distinguished from those of *Hamus mangunensis* sp. n. by the narrower terminal apophysis process I (Ta-I) (fig. 1B, D vs. Fig. 5A, D). Females are distinguished from those of *Hamus cornutus* sp. n. by the nearly round spermathecae and the strongly twisted copulatory ducts (Cd) (fig. 2A–C with Cd reported as Fd vs. Fig. 2E–G), and from specimens of *Hamus luzon* sp. n. by the more pyriform shaped spermathecae and the longer and wider fertilization ducts (Fd) (fig. 2A–C with Fd reported as Id vs. Fig. 4D–F).

#### Description.

See Ballarin and Li (2015).

#### Habitat.

Forest leaf litter.

#### Distibution.

China (Tibet).

### Hamus cornutus sp. n.

Taxon classificationAnimaliaAraneaeNesticidae

http://zoobank.org/7CF47809-19D3-4348-9876-0D63D8EF95AB

Figs 1 , 2 , 81

#### Type material.

Holotype ♂ and paratypes 3♂3♀ (IZCAS), CHINA: Guangxi Zhuang Autonomous Region: Chongzuo City, Pingxiang, Sanzhishan Cave (22.07567°N, 106.73773°E, 257 m), 7.V.2015, Z. Chen & Y. Li leg. Paratype 1♂ (IZCAS), LAOS: Bolikhamxay Province, Khamkeut District, 17.11 km west of Ban Laksao Town, Tham Mankone (18.22156°N, 104.81268°E, 495 m), 27.IX.2012, S. Li leg.

#### Etymology.

The specific name derives from the Latin word “*cornutus*” = horned, and refers to the horn-like distal process of the paracymbium; adjective.

#### Diagnosis.

Males can be distinguished from those of *Hamus bowoensis*, *Hamus mangunensis* sp. n. and *Hamus kangdingensis* sp. n. by the absence of the terminal apophysis process I (Ta-I) (Fig. 1A), the sharp, hooked terminal apophysis process II (Ta-II) (Fig. 1A, D) and the horn-like distal process of the paracymbium (Dp) (Fig. 1A–B), as opposed to a long Ta-I and a needle-like, very short or caniniform Dp in *Hamus bowoensis* (see Ballarin and Li 2015: 180, fig. 1A–D) and *Hamus mangunensis* sp. n. (Fig. 5A–B, D) and as opposed to a blunt Ta-I and a pointed Dp in *Hamus kangdingensis* sp. n. (Fig. 3D, E, G). Females can be separated from those of the similar species *Hamus bowoensis* and *Hamus luzon* sp. n. by the more pear-shaped spermathecae (Fig. 2E–F) and the relatively straight copulatory ducts (Cd) (Fig. 2G), as opposed to the almost round spermathecae and the strongly twisted Cd in *Hamus bowoensis* (see Ballarin and Li 2015: 180, fig. 2A–C with Cd reported as Fd) and *Hamus luzon* sp. n. (Fig. 4D–F).

#### Description.

Habitus as in Fig. 2A–D. Carapace yellowish. Mouthparts yellow in the male, light brown in the female. Sternum pale yellow in males, yellow in females. Legs uniformly pale yellow. Opisthosoma yellowish, slightly dark in males.

Male palp (Fig. 1A–D): paracymbium with a flat, semi-transparent dorsal apophysis and a strongly sclerotized, horn-like distal process (Fig. 1A). Ventral apophysis nearly semi-circular (Fig. 1C). Terminal apophysis almost round and strongly sclerotized (Fig. 1A). Short, sclerotized horned process (Cp-I) at the subapical part of the bulb together with a laminar, elongate process (Cp-II) forming a curved groove (Fig. 1A, D).

Epigyne (Fig. 2E–G): square, fertilization ducts long and twisted, originating at the ventro-lateral base of the spermathecae (Fig. 2F). (Fig. 2G). Spermathecae wide, pear-shaped, and close to each other.

Male (holotype). Total length 1.56. Carapace 0.80 long, 0.73 wide. Opisthosoma 0.77 long, 0.69 wide. Clypeus height 0.15. Sternum 0.47 long, 0.47 wide. Leg measurements: see Appendix A.

Female (one of the paratypes). Total length 2.02. Carapace 0.90 long, 0.78 wide. Opisthosoma 1.22 long, 0.87 wide. Clypeus height 0.18. Sternum 0.56 long, 0.49 wide. Leg measurements: see Appendix A.

#### Habitat.

Forest leaf litter, cave.

#### Distribution.

China (Guangxi), Laos (Fig. 81).

### Hamus kangdingensis sp. n.

Taxon classificationAnimaliaAraneaeNesticidae

http://zoobank.org/0E265247-1616-4EAA-AFAB-BE08E79DFAE7

Figs 3 , 81

#### Type material.

Holotype ♂ and paratype 1♂ (IZCAS), CHINA: Sichuan Province, Garze Prefecture, Kangding County, Jintang-Kongyu Nature Reserve (30.52280°N, 102.06210°E), 18.IV.2004, S. Li leg.

#### Etymology.

The specific name is derived from the type locality; adjective.

#### Diagnosis.

Males can be easily distinguished from those of the other congeners by the caniniform distal process of the paracymbium (Dp) (Fig. 3E, H). Another character to separate them from males of *Hamus bowoensis*, *Hamus cornutus* sp. n. and *Hamus mangunensis* sp. n. is the wider terminal apophysis with a blunt and short process I (Ta-I) (Fig. 3F–G), whereas the entire Ta is a large C-shape in *Hamus bowoensis* (see Ballarin and Li 2015: 180, fig. 1B–C) and *Hamus mangunensis* sp. n. (Fig. 5A, C) and shaped as an inverted comma with a missing Ta-I in *Hamus cornutus* sp. n. (Fig. 1A).

#### Description.

Habitus as in Fig. 3A–C. Carapace faint yellow. Mouthparts and sternum pale yellow. Legs uniformly faint yellow. Opisthosoma uniformly yellowish without dark pigmentation.

Male palp (Fig. 3D–H): paracymbium broad, with an lobed ventral apophysis and a lamellar, translucent dorsal apophysis, ending with a strongly sclerotized, dentiform distal process (Fig. 3G–H). Ventral apophysis lobed, slightly sclerotized (Fig. 3G–H). Terminal apophysis well-developed, strongly sclerotized, Ta-I blunt and short, Ta-II sharp and turned up (Fig. 3D, F–G). Two blunt processes at the base of the conductor, Cp-I short and stumpy, Cp-II laminar and elongate forming a curved groove (Fig. 3D).

Male (holotype). Total length 1.48. Carapace 0.79 long, 0.68 wide. Opisthosoma 0.78 long, 0.60 wide. Clypeus height 0.16. Sternum 0.48 long, 0.48 long. Leg measurements: see Appendix A.

Female. Unknown.

#### Habitat.

Forest leaf litter.

#### Distribution.

Known only from the type locality (Fig. 81).

### Hamus luzon sp. n.

Taxon classificationAnimaliaAraneaeNesticidae

http://zoobank.org/223A32DD-62F6-4D51-9CD5-EE328F68D7F7

Figs 4 , 81

#### Type material.

Holotype ♀ and paratype 1♀ (IZCAS), Philippines: Luzon Island, Mountain Province, in rainforest along the road from Bontoc to Gawana Village, leaf litter (17.06066°N, 121.05067°E, 1674 m), 26.V.2015, F. Ballarin & Y. Li leg.

#### Etymology.

The specific name is derived from the type locality, the island of Luzon in the Philippines; noun in apposition.

#### Diagnosis.

Females can be distinguished from those of *Hamus bowoensis* and *Hamus cornutus* sp. n. by the almost perfectly round spermathecae (S) and the shorter, narrower fertilization ducts (Fd) (Fig. 4E–F). In the other two species, the shape of the spermathecae are more pyriform, and the fertilization ducts are longer and wider (see Fig. 2F–G vs. Ballarin and Li 2015: 180, fig. 2B–C with Fd reported as Id).

#### Description.

Habitus as in Fig. 4A–C. Carapace yellow. Thoracic area with four long setae in the center. Mouthparts brown-yellowish. Sternum yellow. Legs uniformly yellow. Opisthosoma, grey-bluish.

Epigyne (Fig. 4D–F): translucent (Fig. 4D). Spermathecae wide, strongly sclerotized, almost round and close to each other. (Fig. 4E–F). Fertilization ducts short and narrow, connected to the base of the spermathecae and located in the front of the copulatory ducts. Together they form an upturned structure (Fig. 4E–F). Copulatory ducts short, forming a coiled structure in the center (Fig. 4F).

Female (holotype). Total length 2.10. Carapace 0.95 long, 0.80 wide. Opisthosoma 1.34 long, 0.94 wide. Clypeus height 0.20. Sternum 0.57 long, 0.55 wide. Leg measurements: see Appendix A.

Male. Unknown.

#### Habitat.

Rainforest leaf litter.

#### Distribution.

Known only from the type locality (Fig. 81).

### Hamus mangunensis sp. n.

Taxon classificationAnimaliaAraneaeNesticidae

http://zoobank.org/30B1833D-C6B3-4050-8106-84D2EEBB5F29

Figs 5 , 81

#### Type material.

Holotype ♂ (IZCAS), CHINA: Yunnan Province, Xishuangbanna Dai Autonomous Prefecture, Menghai County, Mangun Village, Xishuangbanna Nature Reserve, secondary forest (22.02953°N, 100.39518°E), 1.I.2013, Q. Zhao & Z. Chen leg.

#### Etymology.

The specific name is derived from the type locality, Mangun Village; adjective.

#### Diagnosis.

*Hamus mangunensis* sp. n. is easily distinguished from the other *Hamus* species by a tiny dentiform distal process of the paracymbium (Dp) (Fig. 5B, D), more developed in the other species, and by the wide terminal apophysis process I (Ta-I) with a blunt tip (Fig. 5A, D) which is absent in *Hamus cornutus* sp. n. (Fig. 1A, D), very short in *Hamus kangdingensis* sp. n. (Fig. 3D, G) and narrower with a sharp point in *Hamus bowoensis* (see Ballarin and Li 2015: 180, fig. 1B–D).

#### Description.

Habitus cannot be properly described due to the very poor condition of the sample.

Male palp (Fig. 5A–D): paracymbium weakly sclerotized, dorsal apophysis laminar and translucent, ventral apophysis broad and earlobe-shaped, distal process very tiny, sclerotized (Fig. 5A–B, D). Terminal apophysis well-developed and strongly sclerotized, with two elongate processes (Ta-I-II) forming together a C-like structure (Fig. 5A, C, D). Conductor with two processes near its base, Cp-I short and flat, hook-like, Cp-II elongate and laminar, forming a curved groove. (Fig. 5A, C).

Female. Unknown.

#### Habitat.

Forest leaf litter.

#### Distribution.

Known only from the type locality (Fig. 81).

### Genus Nescina

Taxon classificationAnimaliaAraneaeNesticidae

Ballarin & Li, 2015

Nescina Ballarin & Li, 2015: 183.

#### Type species.

*Nescina minuta* Ballarin & Li, 2015 from Yunnan, China.

#### Diagnosis.

Males of *Nescina* are distinguished from those of the other genera of Nesticidae with the exception of *Hamus* and *Wraios* by a short and very simple paracymbium having only a lobed ventral apophysis (Va) and a sclerotized, hook-shaped distal process (Dp), by the almost round, ventrally flat bulb and by the elongate, laminar conductor (C) with only one small process (Cp-I). Males of *Nescina* can be separated from those of *Hamus* by the smaller terminal apophysis II (Ta-II) and the reduced, laminar process I of the conductor (Cp-I), and from those of *Wraios* by the shape of embolus. Diagnostic characters for the females are the long and twisted fertilization ducts (Fd) which have no similarities in the other genera. Furthermore *Nescina* is easily distinguished by the remarkably small size of the adults (total length less than 1.60) and the protruding cephalic area clearly separated from the thoracic area by a distinct cervical groove.

#### Description.

Total length: 1.30–1.56 (male), 1.44–1.57 (female). Carapace ovate, pale yellow. Cephalic area strongly elevated and clearly differentiated from the rest of the carapace. Eyes ALE=PLE>PME>AME, AER and PER procurved, MOA square. Cervical groove and fovea indistinct. Chelicera with six promarginal teeth and two retromarginal tiny denticles on the fang furrow. Sternum yellowish, heart-shaped. Opisthosoma yellowish, usually with four darker marks more or less visible. Spinnerets and colulus pale yellow.

Male palp: tibia short, as long as wide, with two retrodorsal trichobothria and several long setae. Cymbium almost round with long setae and long furrow on the retrolateral margin. Paracymbium simple, with a wide, lobed ventral apophysis and a hook-like distal process bent ventrally. Bulb ventrally flat. Terminal apophysis small and flat with two processes, Ta-I laminar, Ta-II spur-like. Tegular apophysis missing. Conductor laminar and long, starting from the anterior margin of the tegulum and following the embolus on the prolateral side with a counterclockwise course. One laminar process (Cp-I) at the subapical margin. Embolus filamentous starting from the retrolateral side of the bulb and continuing bordering the tegulum with a semicircular clockwise course.

Epigyne: wide, copulatory openings located near the epigynal posterior margin. Spermathecae and fertilization ducts can be observed through the transparent tegument. Spermathecae wide and globular, fertilization ducts long and twisted, laterally oriented. Copulatory ducts short.

#### Composition.

*Nescina minuta* Ballarin & Li, 2015 and *Nescina kohi* sp. n.

#### Distribution.

China (Yunnan), Singapore (Fig. 81).

### Nescina minuta

Taxon classificationAnimaliaAraneaeNesticidae

Ballarin & Li, 2015

Nescina minuta Ballarin & Li, 2015: 183, 188, figs 3–4 (♂♀).

#### Diagnosis.

**Figure 6.**
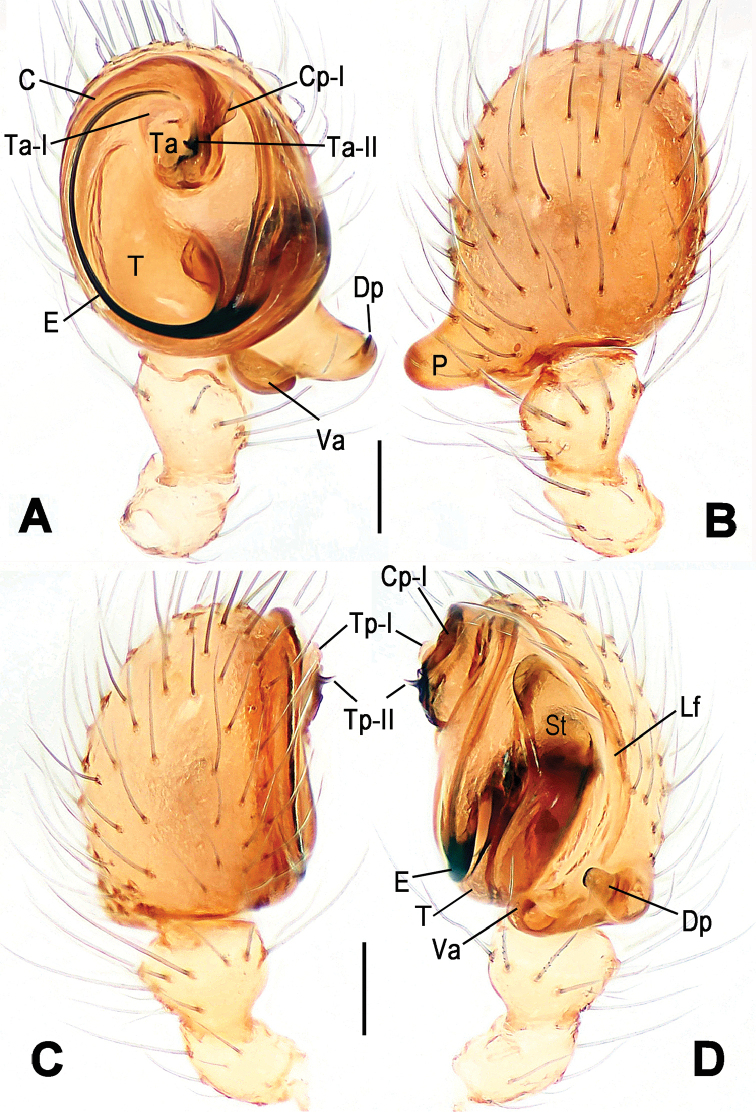
*Nescina kohi* sp. n., holotype (male). **A** Palp, ventral view **B** Ditto, dorsal view **C** Ditto, prolateral view **D** Ditto, retrolateral view. Scale bars: 0.10 mm.

**Figure 7.**
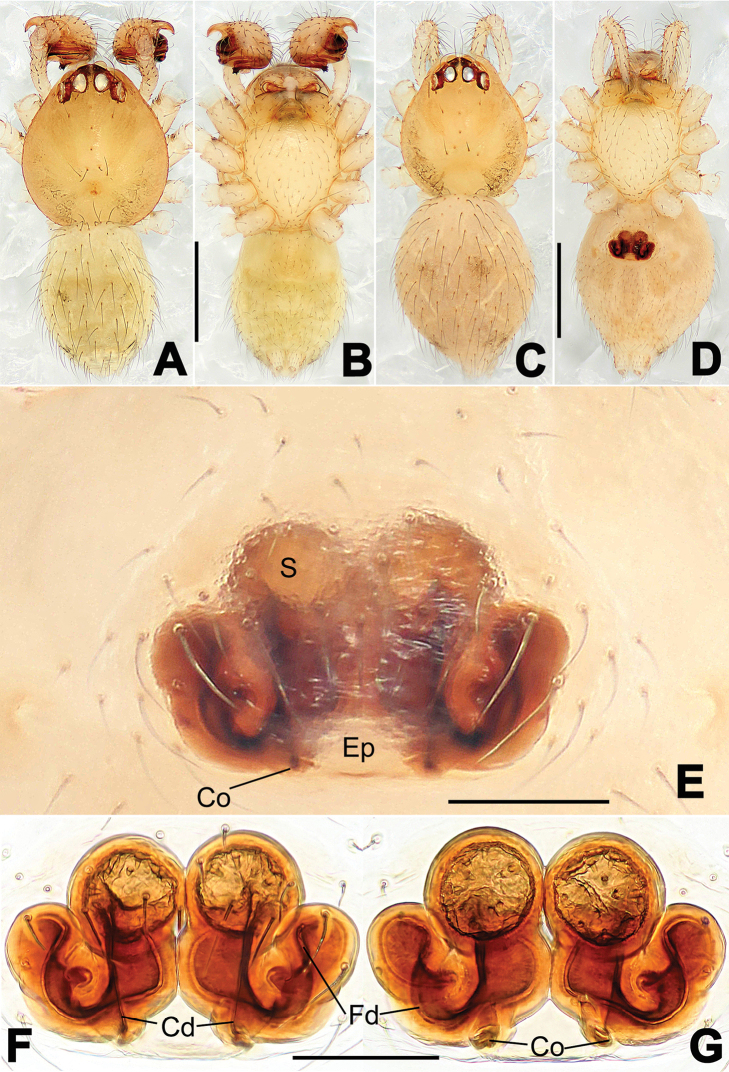
*Nescina kohi* sp. n., holotype (male) and paratype (female). **A** Male habitus, dorsal view **B** Ditto, ventral view **C** Female habitus, dorsal view **D** Ditto, ventral view **E** Epigyne, ventral view **F** Vulva, ventral view **G** Vulva, dorsal view. Scale bars: **A–D** = 0.50 mm; **E–G** = 0.10 mm. Arrows indicate cervical groove in **A** and **C**.

Males can be separated from those of *Nescina kohi* sp. n. by the thicker terminal apophysis (Ta), by the different shape of the process I of the conductor (Cp-I) (see Ballarin and Li 2015: 183, fig 3B–D vs. Fig. 6A, D) and by the slightly larger body size. Females are recognized by the shorter fertilization ducts (Fd) at the sides of the spermathecae (fig. 4A–C with Fd reported as Id vs. Fig. 7E–G).

#### Description.

See Ballarin and Li (2015).

#### Habitat.

Forest leaf litter.

#### Distibution.

China (Yunnan).

### Nescina kohi sp. n.

Taxon classificationAnimaliaAraneaeNesticidae

http://zoobank.org/649FC921-7751-4DA4-9CBC-B2FA76537646

Figs 6 , 7 , 81

#### Type material.

Holotype ♂ (LKCNHM), paratypes 2♀ (LKCNHM) and 1♂1♀ (IZCAS), SINGAPORE: Central Catchment Nature Reserve, near Mandai Agrotechnology Park (1.41492°N, 103.79894°E, 46 m), 1.IX.2015, Y. Tong & S. Li leg.

#### Etymology.

The new species is named after Joseph K. H. Koh, a pioneer spider researcher in Singapore; noun (name) in genitive case.

#### Diagnosis.

*Nescina kohi* sp. n. morphologically is very similar to *Nescina minuta* (see Figs 6A–D, 7E–G and Ballarin and Li 2015: 183, figs 3A–D, 4A–C). Males of *Nescina kohi* sp. n. can be distinguished from those of *Nescina minuta* by the narrower terminal apophysis (Ta) (Fig. 6A vs. fig. 3B) and the different shape of process I of the conductor (Cp-I) (Fig. 6D vs. fig. 3D). Diagnostic characters for the females are the straighter posteromedial margin of the epigyne (Fig. 7G vs. fig. 4B) and the longer fertilization ducts (Fd) at the sides of the spermathecae (Fig. 7F vs. fig. 4C with Fd reported as Id). Furthermore *Nescina kohi* sp. n. is slightly smaller than *Nescina minuta* (1.30–1.44 vs. 1.56–1.57).

#### Description.

Habitus as in Fig. 7A–D. Carapace yellowish, faintly dark at the margins, with several long setae along the midline, two between AMEs. Mouthparts and sternum pale yellow. Legs and female palps uniformly pale yellow, lacking spines. Opisthosoma yellowish, in the male dorsal side with four faint darker marks.

Male palp (Fig. 6A–D): paracymbium with a wide, lobed ventral apophysis (Fig. 6A) and a short, fingerlike distal process bent ventrally (Fig. 6D). Terminal apophysis small (Fig. 6A), with two processes, Ta-I laminar and translucent apically oriented (Fig. 6A, C–D), and Ta-II small, spur-like and sclerotized, ventrally oriented (Fig. 6A, C–D). Conductor with a small laminar process (Cp-I) at the subapical margin (Fig. 6A).

Epigyne (Fig. 7E–G): posterior margin straight (Fig. 7E–G). Copulatory openings separated from each other by approximately the diameter of the spermathecae (Fig. 7G). Spermathecae globular, close to each other (Fig. 7G). Fertilization ducts long and wide, twisted to form two conjoined saccules below the spermathecae (Fig. 7F–G). Copulatory ducts short and straight (Fig. 7F).

Male (holotype). Total length 1.30. Carapace 0.69 long, 0.62 wide. Opisthosoma 0.65 long, 0.50 wide. Clypeus height 0.13. Sternum 0.46 long, 0.43 wide. Leg measurements: see Appendix A.

Female (one of the paratypes). Total length 1.44. Carapace 0.65 long, 0.58 wide. Opisthosoma 0.82 long, 0.64 wide. Clypeus height 0.09. Sternum 0.45 long, 0.41 wide. Leg measurements: see Appendix A.

#### Habitat.

Forest leaf litter.

#### Distribution.

Known only from the type locality (Fig. 81).

#### Remarks.

The molecular analysis confirms that *Nescina kohi* sp. n. differs from the type species, *Nescina minuta* (see Appendix B). Futhermore *Nescina kohi* sp. n. is distributed in Singapore while the type locality of the other species is in Yunnan, China, with a linear distance of more than 2000 kilometers separating the two sites.

### Genus Nesticella

Taxon classificationAnimaliaAraneaeNesticidae

Lehtinen & Saaristo, 1980

Nesticella Lehtinen & Saaristo, 1980: 55; Wunderlich 1986: 93; Gray 1989: 88; Marusik and Guseinov 2003: 37.

#### Type species.

*Nesticus nepalensis* Hubert, 1973 from Nepal.

#### Diagnosis.

*Nesticella* belongs to the tribe Nesticellini *sensu* Lehtinen & Saaristo (1980). The characters are rather variable within the different species-groups of *Nesticella*, however males can be recognized by the presence of a complex paracymbium usually lacking any dorsal apophysis (a few species with only one), with a simple or bifurcate distal process and one or two ventral processes. By contrast, males of the other small-sized nesticid genera distributed in the Old World (*Hamus*, *Nescina*, *Wraios*) have a shorter and more compact paracymbium. The uncoiled embolus instead of a long and coiled embolus, easily separate *Nesticella* from the small *Pseudonesticus* species. Females of *Nesticella* are recognized by the short, rectangular or lobed and often protruding scape and by the general shape of the copulatory ducts with thick, compact copulatory ducts lacking deep convolutions and located just below the coiled, thin fertilization ducts. The above-mentioned characters are not similar to any other genus of Nesticidae.

#### Description.

Total length: 1.60–2.94 (male), 2.00–3.36 (female). Carapace almost round in males, ovate in females, yellow or pale yellowish, only rarely dark. Cephalic area slightly elevated with sparse setae. Eight eyes in two rows with exceptions in cave-adapted species, ALE>PLE=PME>AME. AER slightly procurved, PER straight MOA trapezoidal, narrower in the front. In troglobitic species (e.g. *Nesticella caeca* sp. n., *Nesticella gazuida* sp. n.) eyes strongly reduced with AME sometimes absent. Cervical groove and fovea usually distinct, sometimes indistinct. Chelicera with three promarginal teeth and multiple retromarginal tiny denticles. Opisthosoma with long setae usually with a yellowish or greyish background and pairs of dark spots partially merged each other on the dorsal side, in some species uniformly black or greyish. Colors and marks can be more or less visible and sometimes reduced to being faint.

Male palp: tibia short and swollen, wider than long, with three retrodorsal trichobothria and sparse long setae. Cymbium with dense and thick long setae dorsal-distally. Paracymbium compact with a stocky, laminar or bifurcate distal process and a ventral apophysis with one or two processes of different length. Dorsal apophysis absent in some species. Terminal apophysis blunt, crest-like or elongate and pointed. Tegular apophysis usually well-developed, missing in *mogera* and *quelpartensis*-groups. Tegulum with a small additional tegular apophysis (Tg-II) in the species belonging to the *nepalensis*-group. Conductor long and curved, basally wide, gradually narrower near the tip, sometimes distally twisted and always with a sclerotized, short beak-shaped process. Embolus long and filamentous, starting from the retroventral margin of the tegulum and coiling with a half loop until reaching the apex of the conductor.

Epigyne: weakly sclerotized showing the internal structures. Scape lobed or squared, sometimes strongly reduced, sometimes well-developed. Copulatory openings at the lateral corners of the scape, under the scape in the *phami*-group. Spermathecae ovoid or almost round, usually small. Fertilization ducts long and thin reaching the spermathecae with one to five coils. Copulatory ducts short and thick, ventrally oriented, sometimes bent in the middle and directed laterally.

#### Distribution.

Mostly Pantropical (Brazil; Central and East Africa; East, South and Southeast Asia; Indochina; Melanesia) with few species occurring in the Russian Far East. *Nesticella mogera* is also found in Azerbaijan, Fiji, Hawaii and Europe, probably introduced by human activities.

#### Remarks.

Although the morphological characters of *Nesticella* species may support the hypothesis of two possible distinct genera as claimed by Lehtinen and Saaristo (1980) and Marusik and Guseinov (2003), the variability within this group is wide, and a further analyses that incorporate molecular data are required to solve the question. Therefore, we prefer to follow the current classification as given in the World Spider Catalog (2016) with all the species included in a single genus corresponding to the tribe Nesticellini *sensu* Lehtinen & Saaristo (1980). We propose five species-groups (*brevipes*, *mogera*, *nepalensis*, *phami* and *quelpartensis*-groups) based on male palpal and epigynal morphology and preliminary results of our molecular analysis of the entire Nesticidae. Two species-groups already established by Lehtinen and Saaristo (1980) (*brevipes*-group and *mogera*-group) are followed while the *nepalensis*-group coincides with the genus *Nesticella* *sensu* Lehtinen & Saaristo (1980). Two more new groups (*phami*-groups and *quelpartensis*-group) are here established.

#### Incertae sedis.

Two recently-described species from Laos and Myanmar, *Nesticella foelixi* Grall & Jäger, 2016 and *Nesticella michaliki* Grall & Jäger, 2016, show peculiar palpal and epigynal characters which don’t allow their clear placement into any *Nesticella* species-group in this paper. Some characters, such as shape of the scape, the wide spermathecae and the flat distal process of the paracymbium, are shared with *phami*-group species; however the thick embolus, the general shape of the process of the conductor, the terminal apophysis and the paracymbium are not shared with this species group. It is possible that these new species represent separate monospecific species-groups. Nevertheless, in absence of further data, these species are temporarily considered as *incertae sedis* until further analyses have been conducted.

### *Nesticella brevipes*-group

**Group features.** Males of this species-group are characterized by the following combination of characters: a ventral apophysis of the paracymbium with two parallel processes (Va-I, Va-II), the first longer than the second and usually ending with a sharp tip; a distal process of the paracymbium with two branches (Dp-I, Dp-II) more or less developed and in some cases heavily reduced (e.g. *Nesticella hongheensis* sp. n.) or partially absent (e.g. *Nesticella dazhuangensis* sp. n. and *Nesticella caeca* sp. n.); a well-developed tegular apophysis (Tg) directed retrolaterally and a small process of the conductor. Females belonging to this species-group are characterized by a protruding lobed scape (Sp) with a rounded tip. The scape can be more rarely short and very wide (wider then long). These features allow a relatively easy separation from females of the *mogera* and *nepalensis*-groups which have a squarer or narrower scape. The smaller spermathecae (S) easily distinguish females of the *brevipes*-group from those of the *phami*-group whereas the bent copulatory ducts (Cd) further separate them from those of the *mogera* and *quelpartensis*-groups.

**Composition.** *Nesticella arcuata* Liu & Li, 2013; *Nesticella baiseensis* sp. n.; *Nesticella brevipes* (Yaginuma, 1970); *Nesticella buicongchieni* (Lehtinen & Saaristo, 1980); *Nesticella caeca* sp. n.; *Nesticella chongqing* sp. n.; *Nesticella dazhuangensis* sp. n.; *Nesticella falcata* Liu & Li, 2013; *Nesticella gazuida* sp. n.; *Nesticella gracilenta* Liu & Li, 2013; *Nesticella hongheensis* sp. n.; *Nesticella jingpo* sp. n.; *Nesticella kerzhneri* (Marusik, 1987); *Nesticella lisu* sp. n.; *Nesticella liuzhaiensis* sp. n.; *Nesticella machadoi* (Hubert, 1971)?; *Nesticella mollicula* (Thorell, 1898); *Nesticella nandanensis* sp. n.; *Nesticella odonta* (Chen, 1984); *Nesticella okinawaensis* (Yaginuma, 1979)?; *Nesticella qiaoqiensis* sp. n.; *Nesticella qiongensis* sp. n.; *Nesticella robusta* sp. n.; *Nesticella sanchaheensis* sp. n.; *Nesticella semicircularis* Liu & Li, 2013; *Nesticella shanlinensis* Liu & Li, 2013; *Nesticella songi* Chen & Zhu, 2004; *Nesticella verticalis* Liu & Li, 2013; *Nesticella xiongmao* sp. n.; *Nesticella xixia* sp. n. and *Nesticella yao* sp. n.

**Remarks.** The placement of *Nesticella machadoi* (Hubert, 1971) and *Nesticella okinawaensis* (Yaginuma, 1979) in the *brevipes*-group is provisional due to the poor original illustrations and descriptions which do not allow a clear understanding the diagnostic characters. A direct and detailed examination of the type material is necessary to confirm their affinity with this species-group.

#### Nesticella baiseensis sp. n.

Taxon classificationAnimaliaAraneaeNesticidae

http://zoobank.org/1AFE674F-991D-4F40-8EB9-7DC7EA90CD61

Figs 8 , 9 , 82

##### Type material.

Holotype ♂ and paratypes 2♀ (IZCAS), CHINA: Guangxi Zhuang Autonomous Region, Baise Prefecture, Lingyun County, Shuiyuan Cave, under rock and stone in caves (24.36656°N, 106.57775°E, 400 m), 27.III.2015, Y. Li & X. Chen leg.

##### Etymology.

The specific name is derived from the type locality; adjective.

##### Diagnosis.

**Figure 8.**
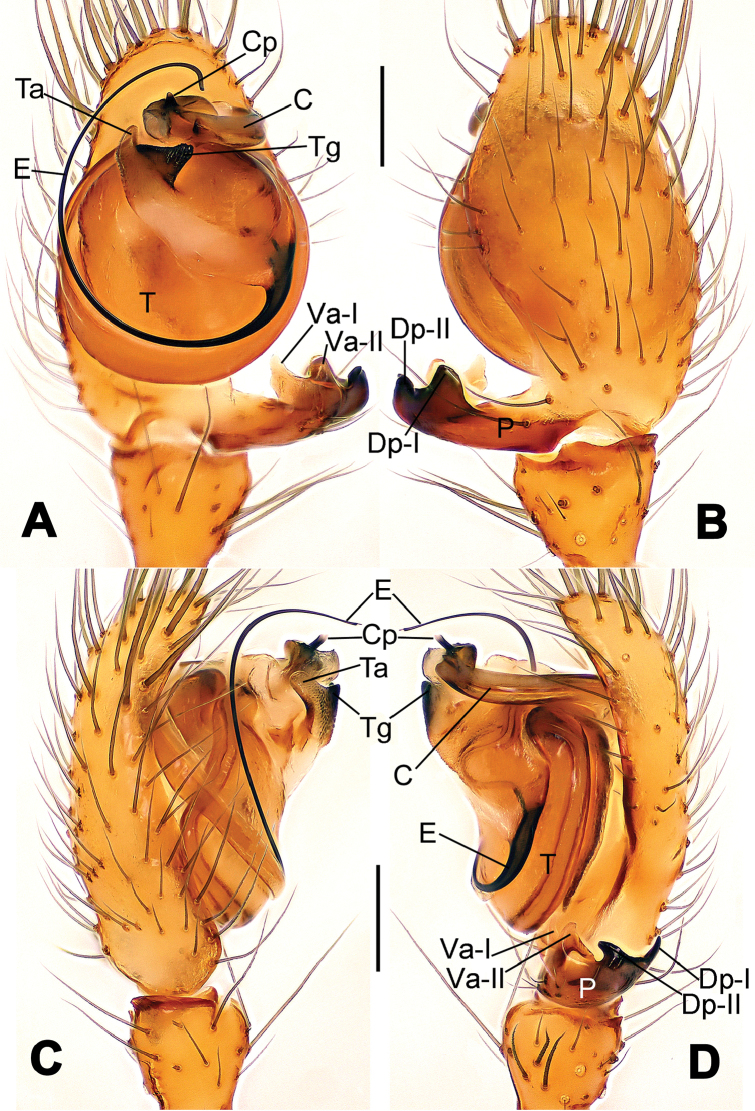
*Nesticella baiseensis* sp. n., holotype (male). **A** Palp, ventral view **B** Ditto, dorsal view **C** Ditto, prolateral view **D** Ditto, retrolateral view. Scale bars: 0.10 mm.

**Figure 9.**
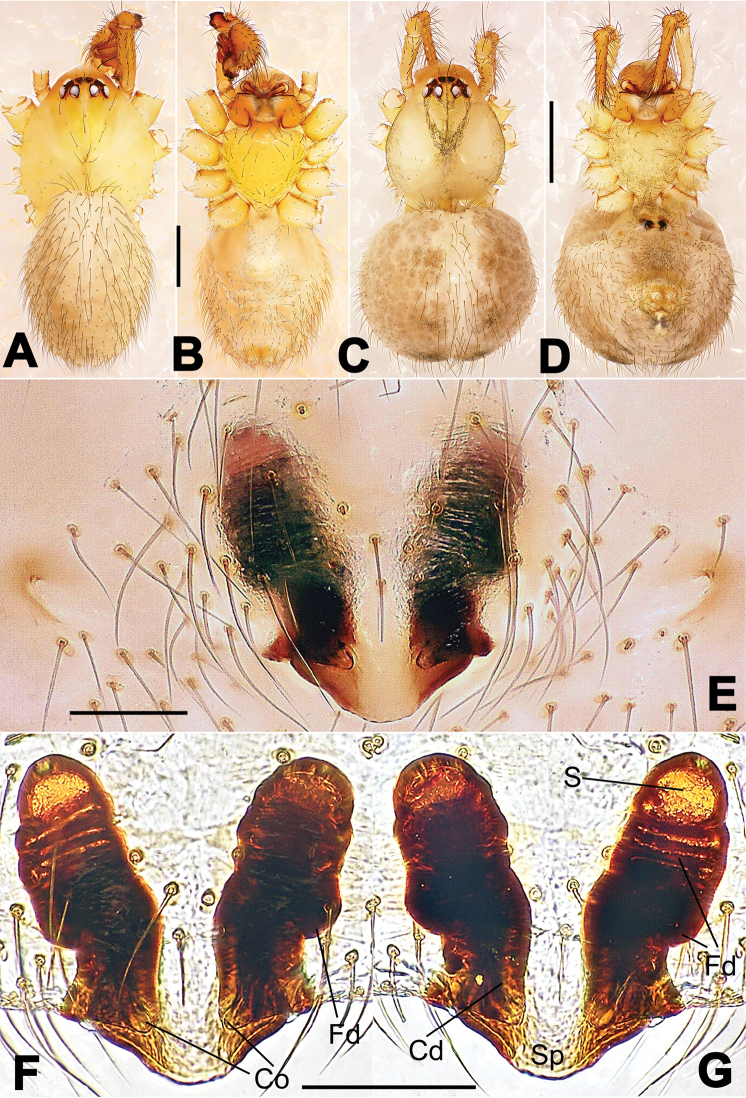
*Nesticella baiseensis* sp. n., holotype (male) and paratype (female). **A** Male habitus, dorsal view **B** Ditto, ventral view **C** Female habitus, dorsal view **D** Ditto, ventral view **E** Epigyne, ventral view **F** Vulva, ventral view **G** Vulva, dorsal view. Scale bars: **A–D** = 0.50 mm; **E–G** = 0.10 mm.

The new species is closely related to *Nesticella nandanensis* sp. n., *Nesticella songi* and *Nesticella verticalis*. It can be separated from *Nesticella nandanensis* sp. n. by the longer tegular apophysis (Ta), the larger angle formed by the distal processes of the paracymbium (Dp-I, Dp-II) (Fig. 8A vs. Fig. 22A in *Nesticella nandanensis*), by the narrower scape and the closer spermathecae (Fig. 9G vs. Fig. 23G in *Nesticella nandanensis*); from *Nesticella songi* by the stockier distal process I (Dp-I) (Fig. 8B vs. Fig. 33B in *Nesticella songi*), the differently bifurcated distal processes (Dp-I, Dp-II) (Fig. 8D vs. Fig. 33D in *Nesticella songi*) and the narrower scape (Sp) (Fig. 9E vs. Fig. 34E in *Nesticella songi*); from *Nesticella verticalis* (see Liu and Li 2013b: 522, figs 27–30) by the longer tegular apophysis (Tg), the thicker distal process I (Dp-I) (Fig. 8A–D vs. figs 27B, 28A, E, D, 29B) and the more coiled fertilization ducts (Fd) (Fig. 9G vs. figs 28C, 30C). The absence of a dorsal apophysis (Da), the different shape of the distal processes of the paracymbium, in particular the Dp-I, the shape of the tegular apophysis (Tg), and, for the females, the narrow scape without a lobe (Sp) clearly distinguish *Nesticella baiseensis* sp. n. from all the other species of the group.

##### Description.

Habitus as in Fig. 9A–D. Carapace uniformly pale yellow in males, yellowish with a “V”-shaped dark mark on the cephalic area in females. Ocular area with a long seta between AMEs. Cervical groove and fovea indistinct. Mouthparts brown yellow. Sternum orange in males, faintly dark in females, with sparse long setae in the center, thin and short near the margins. Legs and female palps yellowish, distally darker. Opisthosoma yellowish with faint dark areas.

Male palp (Fig. 8A–D): paracymbium with Va-I long and laminar, Va-II short and swollen (Fig. 8A, D), distal process wide and bifurcated, strongly sclerotized (Fig. 8B, D); dorsal apophysis absent. Terminal apophysis wide and blunt, finger-like, and granulated (Fig. 8A, C). Tegular apophysis well-developed, triangular, strongly sclerotized and rugose (Fig. 8A). Conductor with a short, sclerotized, finger-like process (Fig. 8A, C–D).

Epigyne (Fig. 9E–G): light yellow (Fig. 9E). Scape arrow-shaped with a blunt tip and weakly sclerotized sides (Fig. 9E–G). Spermathecae small, ovoid, separate from each other by approximately 1.5 of their diameters (Fig. 9G). Fertilization ducts long and thin, reaching the spermathecae with 4.5 coils (Fig. 9F–G). Copulatory ducts short and thick, basally wider and narrower in the middle (Fig. 9F–G).

Male (holotype). Total length 2.97. Carapace 1.47 long, 1.31 wide. Opisthosoma 1.72 long, 1.27 wide. Clypeus height 0.25. Sternum 0.90 long, 0.83 wide. Leg measurements: see Appendix A.

Female (one of the paratypes). Total length 3.28. Carapace 1.37 long, 1.19 wide. Opisthosoma 2.09 long, 1.72 wide. Clypeus height 0.25. Sternum 0.84 long, 0.79 wide. Leg measurements: see Appendix A.

##### Habitat.

Cave.

##### Distribution.

Known only from the type locality (Fig. 82).

#### Nesticella brevipes

Taxon classificationAnimaliaAraneaeNesticidae

(Yaginuma, 1970)

**Figure 10.**
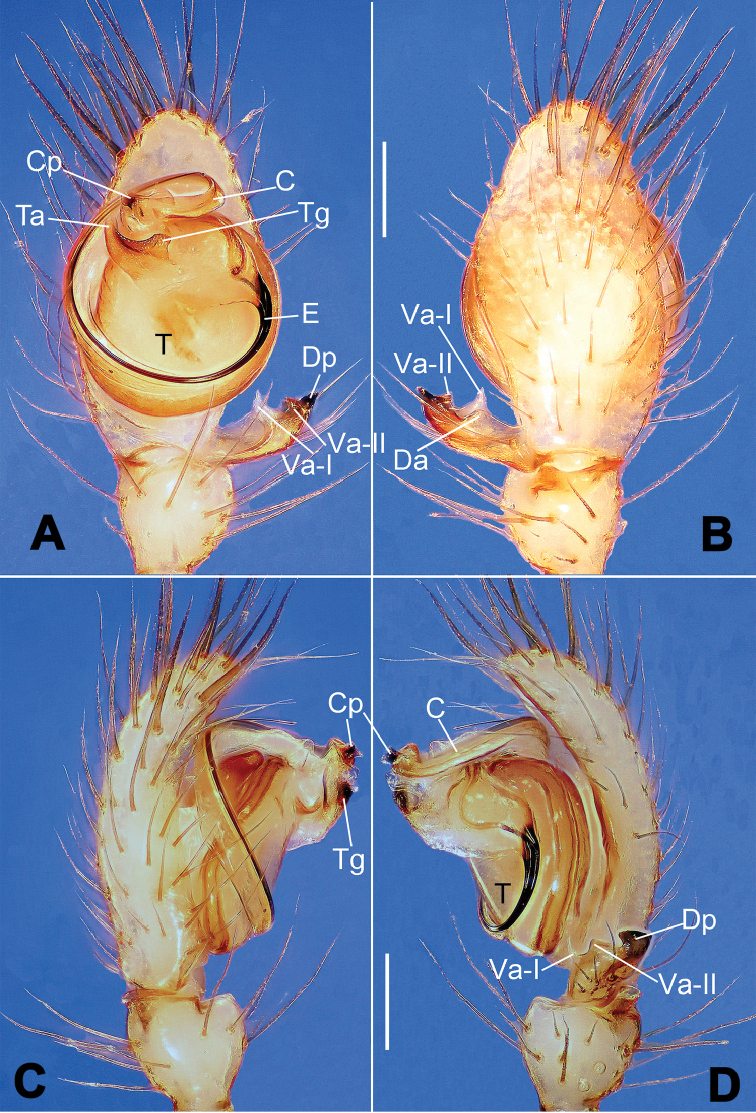
*Nesticella caeca* sp. n., holotype (male). **A** Palp, ventral view **B** Ditto, dorsal view **C** Ditto, prolateral view **D** Ditto, retrolateral view. Scale bars: 0.10 mm.

Nesticus brevipes Yaginuma, 1970: 386 figs 1–2, 9–10 (♂♀).
Howaia brevipes : Lehtinen and Saaristo 1980: 54 (transfer from Nesticus).
Nesticella brevipes : Song et al. 1999: 85, fig. 37G (♂).
Nesticella brevipes : Kamura and Irie 2009: 353, figs 103–107 (♂♀).
Howaia brevipes : Marusik and Kovblyuk 2011: 199, figs 25.1–2, 4–5 (♂♀). See the World Spider Catalog (2016) for the full list of references.

##### Diagnosis.

Males of *Nesticella brevipes* are distinguished from those of all the other species belonging to the *brevipes*-group by the well-developed and protruding dorsal apophysis (missing or only slightly protruding in the other species) and by the single, thin and sharp distal process of the paracymbium (usually bifurcated or, if single, thinner or blunt in the other species of the brevipes-group) (see Yaginuma 1970: 386 figs 1–2 and Kamura and Irie 2009: 353, figs 103–105). Females are recognized by the squared but usually well protruding scape and by the short and straight V-shaped copulatory ducts lacking undulations as in the other members of the group (see Kamura and Irie 2009: 353, figs 106–107).

##### Description.

See Yaginuma (1970).

##### Habitat.

Forest leaf litter, cave.

##### Distribution.

China (Zhejiang), Japan, Korea, Russia (Sakhalin, South Kuril Islands).

#### Nesticella caeca sp. n.

Taxon classificationAnimaliaAraneaeNesticidae

http://zoobank.org/0A119D74-26FC-40E5-8EF7-1BD24763563A

Figs 10 , 11 , 82

##### Type material.

Holotype ♂ and paratypes 2♀ (IZCAS), CHINA: Guizhou Province, Tianzhu County, Liuhe Village, Jinshanrongdong Cave (26.96115°N, 109.20617°E, 476 m), 24.III.2011, H. Chen & Z. Zha leg.

##### Etymology.

The epithet derives from the Latin word “*caecus*” = blind, referring to the vestigial eyes; adjective.

##### Diagnosis.

**Figure 11.**
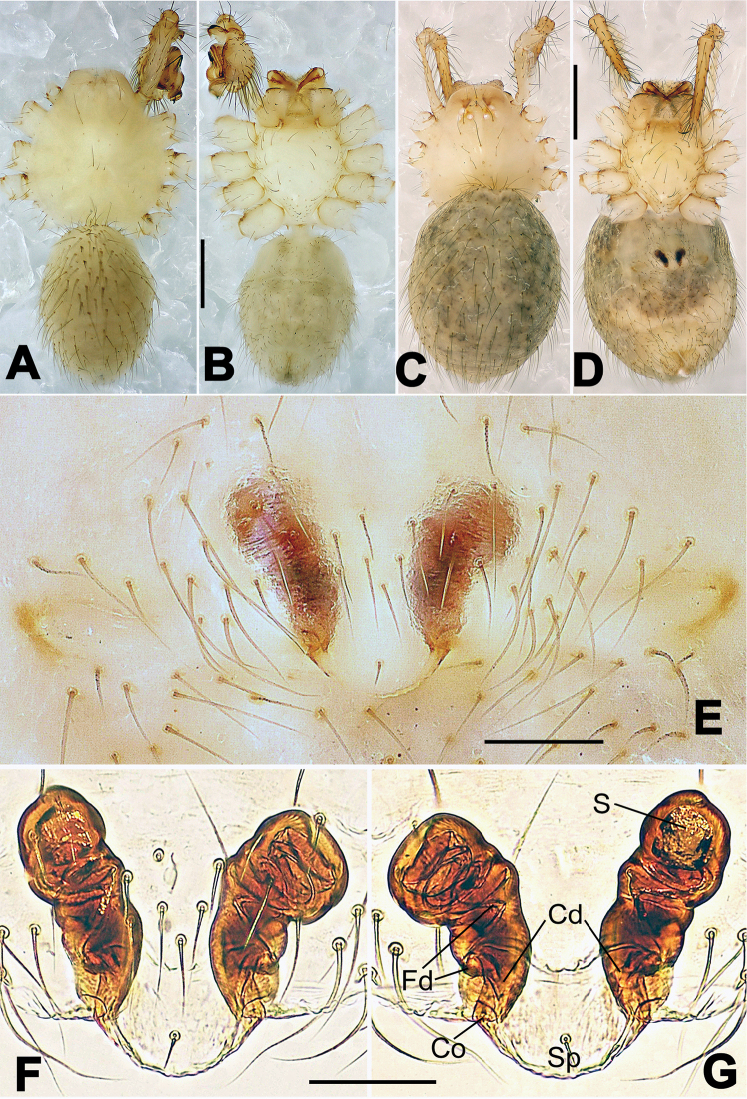
*Nesticella caeca* sp. n., holotype (male) and paratype (female). **A** Male habitus, dorsal view **B** Ditto, ventral view **C** Female habitus, dorsal view **D** Ditto, ventral view **E** Epigyne, ventral view **F** Vulva, ventral view **G** Vulva, dorsal view. Scale bars: **A–D** = 0.50 mm; **E–G** = 0.10 mm.

Males and females of *Nesticella caeca* sp. n. can be easily recognized from those of the other species belonging to the *brevipes*-group, with the exception of *Nesticella gazuida* sp. n. (Fig. 15A–F), by the vestigial eyes (Fig. 11A, 11C); further differences can be found in the shape of the ventral (Va) and distal (Dp) processes of the paracymbium (Fig. 10A–B, D) and in the epigynal scape. *Nesticella caeca* sp. n. can be separated from *Nesticella gazuida* sp. n. by the different configuration of the fertilization (Fd) and copulatory ducts (Cd) (straighter, thicker and shorter in *Nesticella caeca* sp. n. than in *Nesticella gazuida* sp. n.) (Fig. 11G vs. Fig. 15F).

##### Description.

Habitus as in Fig. 11A–D. Carapace pale yellow. Six vestigial eyes, AME absent, the others strongly reduced. Cervical groove and fovea indistinct. Mouthparts pale yellow. Sternum pale yellowish with long setae. Legs with the same color of the carapace but slightly darker. Opisthosoma uniformly greyish with faint darker areas.

Male palp (Fig. 10A–D): paracymbium relatively small, with four or five dorsal setae (Fig. 10D), Va-I elongate, with a sharp tip, Va-II stocky and strongly reduced (Fig. 10A–B, D), distal process blunt and strongly sclerotized, unbranched (Fig. 10D). Terminal apophysis finger-like with a granulated surface, longer than the tegular apophysis. Tegular apophysis blunt and rugose (Fig. 10A). Conductor with a very short, sclerotized process near its apex (Fig. 10A, C–D).

Epigyne (Fig. 11E–F): weakly sclerotized, with sparse long setae (Fig. 11E). Scape laminar, lobed, approximately two times wider than long (Fig. 11F). Spermathecae globular, separated from each other by about 1.4 of their diameters (Fig. 11F–G). Fertilization ducts long and thin, reaching the spermathecae with two to three coils (Fig. 11G). Copulatory ducts straight and thick (Fig. 11G).

Male (holotype). Total length 2.43. Carapace 1.19 long, 1.08 wide. Opisthosoma 1.30 long, 0.87 wide. Clypeus height 0.22. Sternum 0.74 long, 0.66 wdie. Leg measurements: see Appendix A.

Female (one of the paratypes). Total length 2.50. Carapace 1.14 long, 1.00 wide. Opisthosoma 1.66 long, 1.25 wide. Clypeus height 0.20. Sternum 0.69 long, 0.67 wide. Leg measurements: see Appendix A.

##### Habitat.

Cave.

##### Distribution.

Known only from the type locality (Fig. 82).

##### Remarks.

The molecular analysis shows that there are certain differences between *Nesticella caeca* sp. n. and the close related *Nesticella gazuida* sp. n. (see Appendix B).

#### Nesticella chongqing sp. n.

Taxon classificationAnimaliaAraneaeNesticidae

http://zoobank.org/E71031C7-3807-4AD0-8581-10525DD7BAFF

Figs 12 , 82

##### Type material.

Holotype ♀ and paratype 1♀ (IZCAS), CHINA: Chongqing Municipality, Beibei District, Dajiang Village, Xiaofang Cave (30.04050°N, 106.59263°E, 799 m), 30.IV.2014, Y. Lin, H. Zhao, Y. Li, J.Wu & F. Li leg.

##### Etymology.

The specific name is derived from the type locality, the city of Chongqing; noun in apposition.

##### Diagnosis.

**Figure 12.**
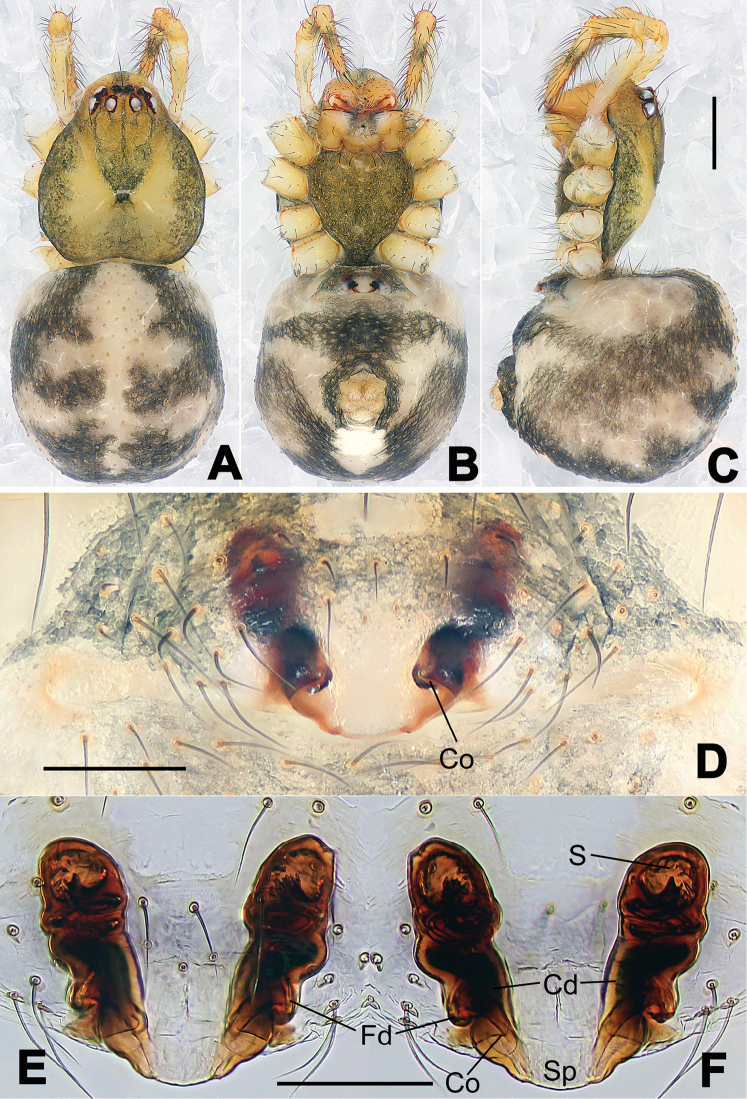
*Nesticella chongqing* sp. n., holotype (female). **A** Habitus, dorsal view **B** Ditto, ventral view **C** Ditto, lateral view **D** Epigyne, ventral view **E** Vulva, ventral view **F** Vulva, dorsal view. Scale bars: **A–C** = 0.50 mm; **D–F** = 0.10 mm.

The new species is closely related to *Nesticella odonta* (see Figs 24A–D, 25A–G and Chen and Zhang 1991: 158, figs 1–6) and *Nesticella xiongmao* sp. n. (Figs 35A–D, 36A–G). It can be separated from the former species by the more protruding and almost round scape (Sp) (Fig. 12D vs. Fig. 25E) and the narrower copulatory ducts (Cd) (Fig. 12F vs. Fig. 25G); it can be distinguished from the latter species by the less protruding, wider scape (Fig. 12D vs. Fig. 36E) and the larger distance between the copulatory ducts (Cd) (Fig. 12F vs. Fig. 36G). The short and slightly round scape and the straight and almost parallel copulatory (Cd) ducts allow the separaton of *Nesticella chongqing* sp. n. from all the other species of the *brevipes*-group.

##### Description.

Habitus as in Fig. 12A–C. Carapace yellow with wide dark areas. Sternum dark grey. Cephalic area with several long setae around the ocular area and along the midline. Cervical groove and fovea distinct. Mouthparts yellow. Sternum with sparse black setae. Legs and palps proximally yellowish, distally darker with dark rings around each tibia and metatarsus. Opisthosoma with a light background and pairs of dark areas partially merged into each other on the dorsal, lateral and ventral sides forming a light, double cross-like mark.

Epigyne (Fig. 12D–F): dark with a lighter mark in the center. Scape short, approximately two to three times wider than long. Posterior and lateral margin weakly sclerotized, slightly protruding beyond the epigynal posterior margin (Fig. 12D). Spermathecae nearly ovoid, separated by about 1.5 diameters (Fig. 12F). Fertilization ducts thin and long, reaching the spermathecae with 3.5 coils. Copulatory ducts short, slightly narrower at their distal end (Fig. 12E–F).

Female (holotype). Total length 2.75. Carapace 1.30 long, 1.13 wide. Opisthosoma 1.47 long, 1.41 wide. Clypeus height 0.23. Sternum 0.75 long, 0.72 wide. Leg measurements: see Appendix A.

Male. Unknown.

##### Habitat.

Cave.

##### Distribution.

Known only from the type locality (Fig. 82).

#### Nesticella dazhuangensis sp. n.

Taxon classificationAnimaliaAraneaeNesticidae

http://zoobank.org/F2DBAEA4-983A-407E-9E29-91940EFDE4CC

Figs 13 , 14 , 82

##### Type material.

Holotype ♂ and paratypes 2♂2♀ (IZCAS), CHINA: Yunnan Province, Tengchong County, Dazhuang Village (25.02670°N, 98.45563°E, 1600 m), 30.XI.2013, Y. Li & J. Liu leg.

##### Etymology.

The specific name is derived from the type locality; adjective.

##### Diagnosis.

**Figure 13.**
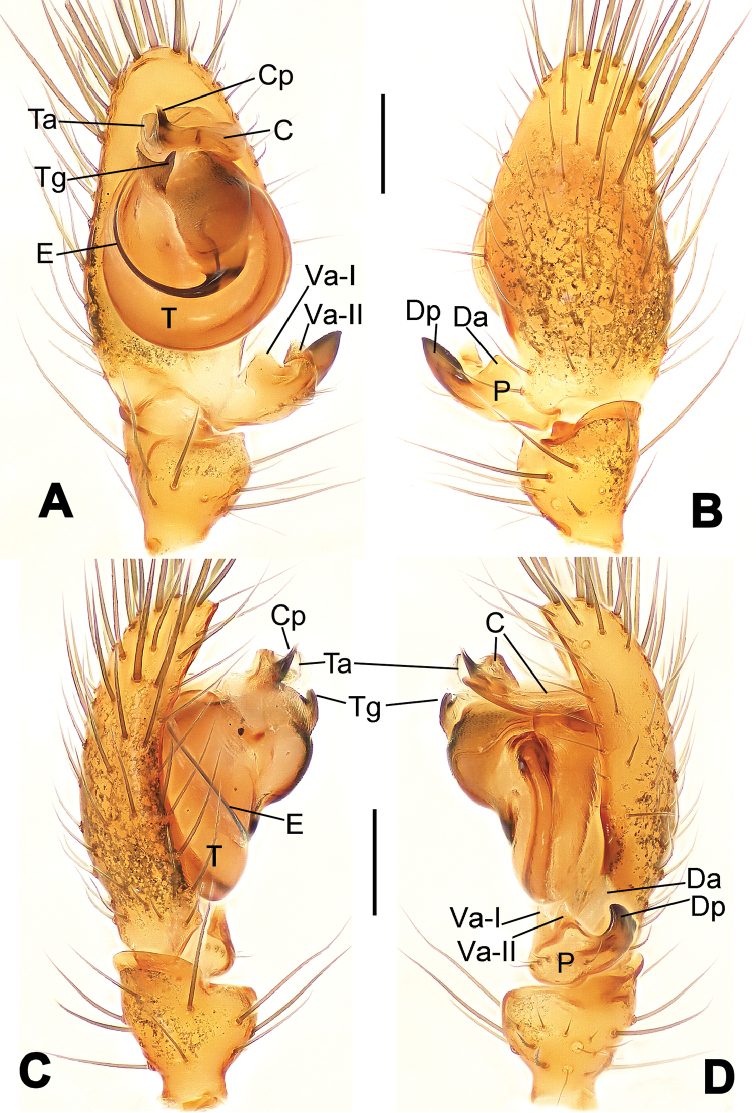
*Nesticella dazhuangensis* sp. n., holotype (male). **A** Palp, ventral view **B** Ditto, dorsal view **C** Ditto, prolateral view **D** Ditto, retrolateral view. Scale bars: 0.10 mm.

**Figure 14.**
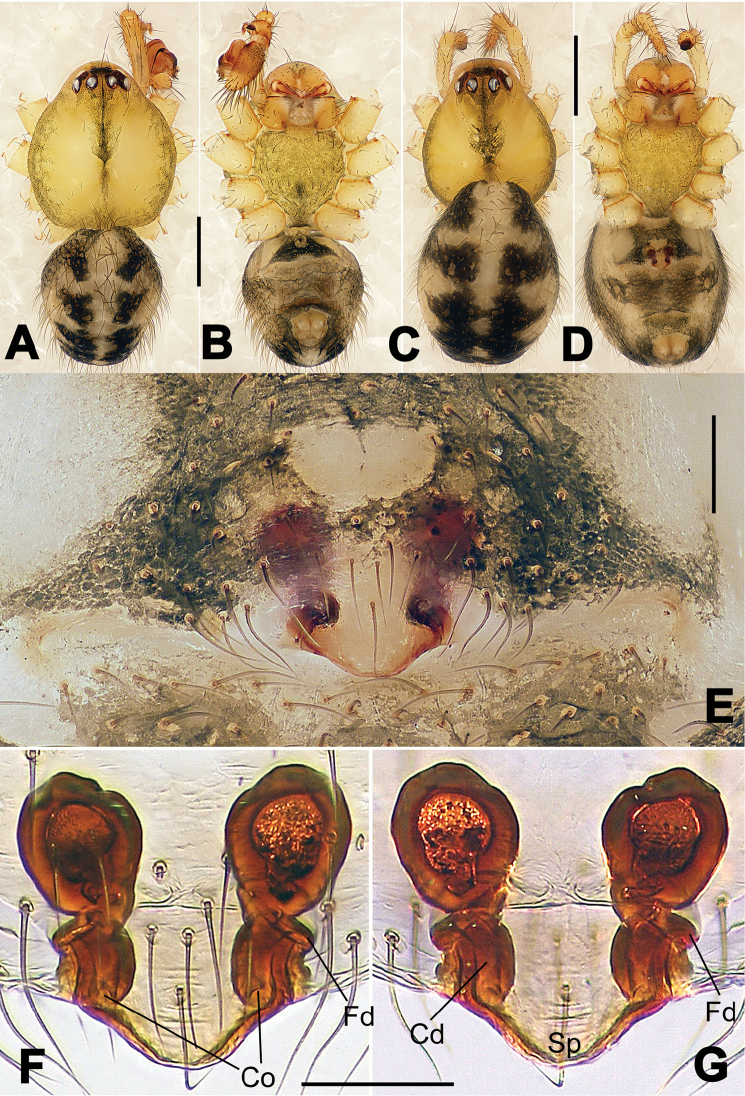
*Nesticella dazhuangensis* sp. n., holotype (male) and paratype (female). **A** Male habitus, dorsal view **B** Ditto, ventral view **C** Female habitus, dorsal view **D** Ditto, ventral view **E** Epigyne, ventral view **F** Vulva, ventral view **G** Vulva, dorsal view. Scale bars: **A–D** = 0.50 mm; **E–G** = 0.10 mm.

Males can be distinguished from those of the other species belonging to the *brevipes*-group by the stocky, unbranched distal process (Dp) of the paracymbium (Fig. 13A–B); by the thin and sharp tegular apophysis (Tg) (Fig. 13A) and by the narrower cymbium when observed in dorsal view (Fig. 13B). Diagnostic characters for the females are the remarkably wide spermathecae (S) (Fig. 14F) and the short, fertilization (Fd) and copulatory (Cd) ducts each with only a few coils (Fig. 14G) which are respectively smaller and longer/more coiled in the other species of the same group.

##### Description.

Habitus as in Fig. 14A–D. Carapace yellow, dark at margins and in the midline. Clypeal area yellowish. A single long seta present between AMEs. Cervical groove faintly visible, fovea distinct. Mouthparts orange. Sternum faintly dark, with sparse long setae. Legs and female palps yellowish, distally darker in each segment. Opisthosoma with a yellowish background and distinct dark pattern formed by transverse bands. Wide dark band between the epigastral furrow and spinnerets.

Male palp (Fig. 13A–D): cymbium approximately two times longer than wide (Fig. 13B). Both ventral processes of the paracymbium stocky and triangular (Fig. 13A), distal process wide and strongly sclerotized, lacking any branch (Fig. 13A–B, D). Dorsal apophysis flat and triangular (Fig. 13B). Terminal apophysis wide and membranous, wrinkled (Fig. 13A). Tegular apophysis sharp, with a wrinkled surface (Fig. 13A, C–D). Conductor ending with a sharp sclerotized process (Fig. 13A, C–D).

Epigyne (Fig. 14E–G): dark, with a pale area in the center (Fig. 14E). Scape short, tongue-like, marginally sclerotized (Fig. 14E–G). Spermathecae broad, ovoid, separated by about 2/3 of their diameters (Fig. 14G). Fertilization ducts short and thin, reaching the spermathecae with only one coil (Fig. 14F–G). Copulatory ducts short and thick (Fig. 14F–G).

Male (holotype). Total length 2.56. Carapace 1.38 long, 1.25 wide. Opisthosoma 1.28 long, 1.00 wide. Clypeus height 0.23. Sternum 0.78 long, 0.75 wide. Leg measurements: see Appendix A.

Female (one of the paratypes). Total length 2.59. Carapace 1.25 long, 1.09 wide. Opisthosoma 1.56 long, 1.16 wide. Clypeus height 0.19. Sternum 0.75 long, 0.68 wide. Leg measurements: see Appendix A.

##### Habitat.

Forest leaf litter.

##### Distribution.

Known only from the type locality (Fig. 82).

#### Nesticella gazuida sp. n.

Taxon classificationAnimaliaAraneaeNesticidae

http://zoobank.org/DCB06612-F086-4FB1-8868-534B4D852952

Figs 15 , 82

##### Type material.

Holotype ♀ (IZCAS), CHINA: Guizhou Province, Kaili City, Zhouxi Town, Gazuida Cave (27.48642°N, 107.92362°E, 680 m), 21.III.2011, H. Chen & Z. Zha leg.

##### Etymology.

The specific name is derived from the name of the cave where the species was collected; noun in apposition.

##### Diagnosis.

**Figure 15.**
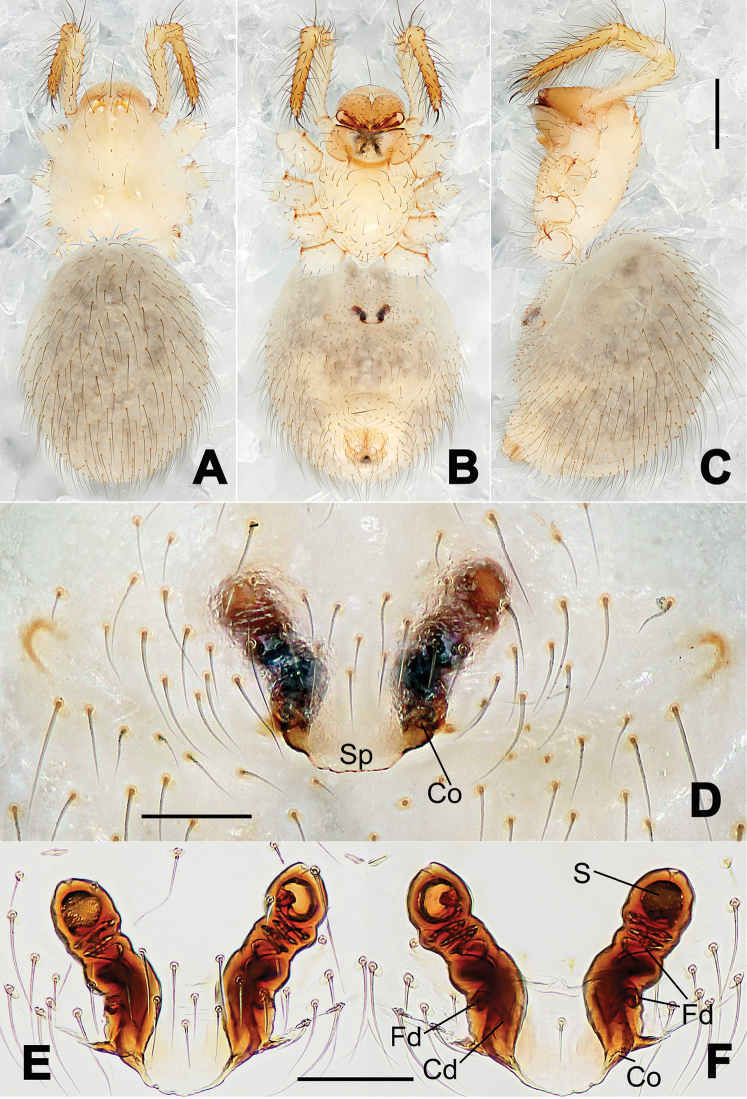
*Nesticella gazuida* sp. n., holotype (female). **A** Habitus, dorsal view **B** Ditto, ventral view **C** Ditto, lateral view **D** Epigyne, ventral view **E** Vulva, ventral view **F** Vulva, dorsal view. Scale bars: **A–C** = 0.50 mm; **D–F** = 0.10 mm.

Females of *Nesticella gazuida* sp. n. are easily distinguished from those of the other species belonging to the *brevipes*-group, with the exception of *Nesticella caeca* sp. n. (Fig. 11A–G), by their pale coloration and the reduced eyes (Fig. 15A). *Nesticella gazuida* sp. n. can be separated from *Nesticella caeca* sp. n. by the smaller spermathecae and especially by the longer fertilization (Fd) and copulatory (Cd) ducts, clearly bent in the middle, which are shorter, more compact and straighter in the latter species (Fig. 15F vs. Fig. 11G).

##### Description.

Habitus as in Fig. 15A–C. Carapace pale yellow. Cephalic area with several long setae along the midline and the cervical groove. Ocular area reduced to white eyespots. Six eyes grouped in two triads, AME absent. Cervical groove and fovea indistinct. Mouthparts and labium pale yellow. Sternum with sparse long setae (Fig. 15B). Legs and female palps pale yellowish, metatarsi and tarsi distally darker. Opisthosoma pale grey. Spinnerets and colulus pale yellow.

Epigyne (Fig. 15D–F): pale yellow, translucent (Fig. 15D). Scape short, translucent, slightly protruding at the epigastric groove (Fig. 15D), about two times wider than the diameters of the spermathecae (Fig. 15E). Spermathecae small, separated by at least 2.5 diameters (Fig. 15E–F). Fertilization ducts thin and long, coiling about 3.5 times before reaching the spermathecae (Fig. 15F). Copulatory ducts thick and short, curved in the middle (Fig. 15E).

Female (holotype). Total length 2.83. Carapace 1.17 long, 1.16 wide. Opisthosoma 1.75 long, 1.34 wide. Clypeus height 0.22. Sternum 0.68 long, 0.66 wide. Leg measurements: see Appendix A.

Male unknown.

##### Habitat.

Cave.

##### Distribution.

Known only from the type locality (Fig. 82).

#### Nesticella hongheensis sp. n.

Taxon classificationAnimaliaAraneaeNesticidae

http://zoobank.org/41EC630A-5862-4309-A07C-25D7409CCDD9

Figs 16 , 17 , 82

##### Material examined.

Holotype ♂ and paratypes 1♂4♀ (IZCAS), CHINA: Yunnan Province, Pingbian County, a mountain without name, close to Honghe River (22.99625°N, 103.65815°E, 1244 m), 20.V.2015, Z. Chen & Y. Li leg.

##### Etymology.

The specific name is derived from the type locality; adjective.

##### Diagnosis.

**Figure 16.**
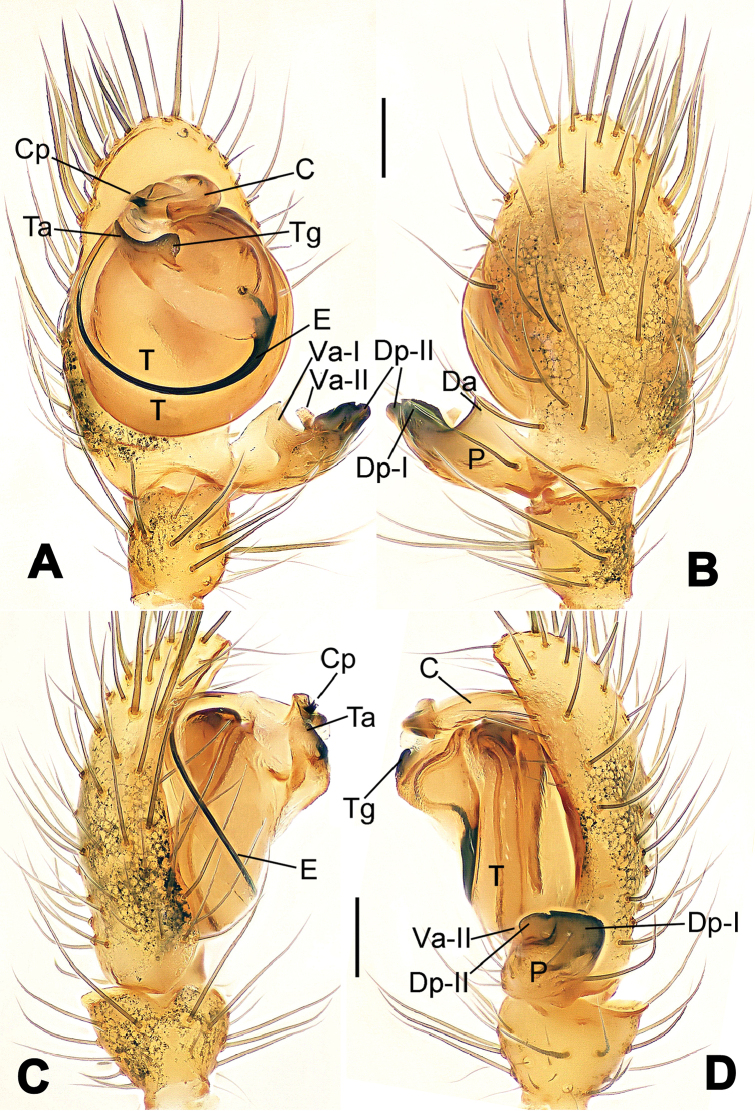
*Nesticella hongheensis* sp. n., holotype (male). **A** Palp, ventral view **B** Ditto, dorsal view **C** Ditto, prolateral view **D** Ditto, retrolateral view. Scale bars: 0.10 mm.

**Figure 17.**
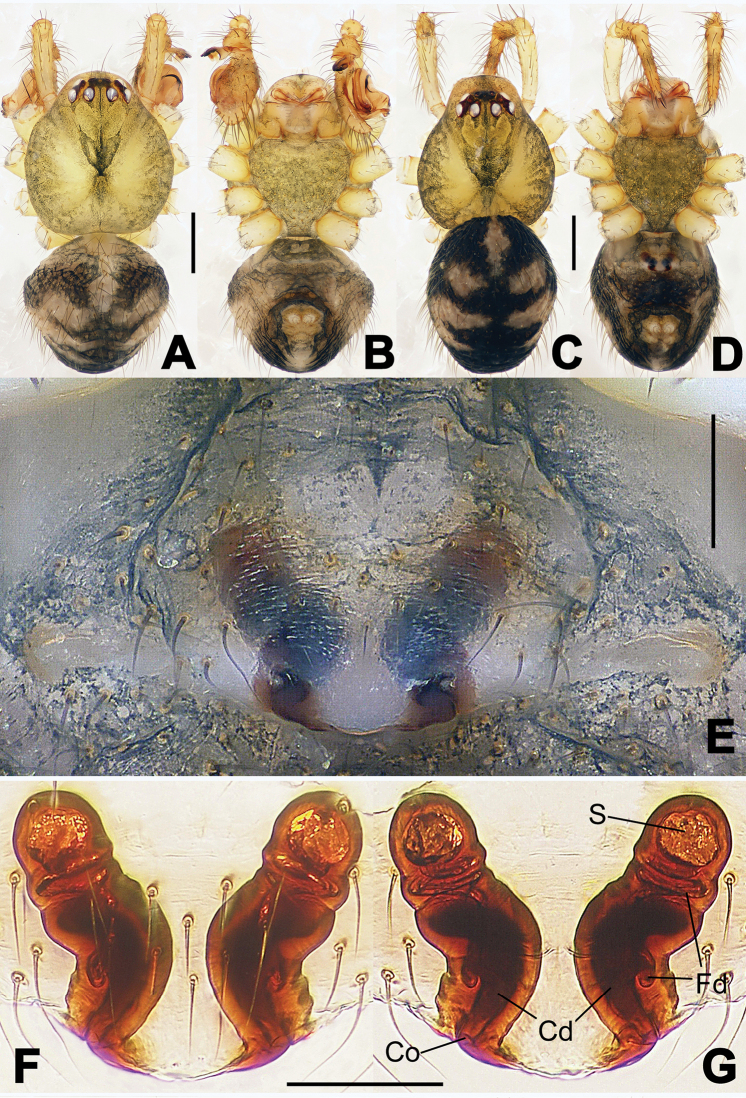
*Nesticella hongheensis* sp. n., holotype (male) and paratype (female). **A** Male habitus, dorsal view **B** Ditto, ventral view **C** Female habitus, dorsal view **D** Ditto, ventral view **E** Epigyne, ventral view **F** Vulva, ventral view **G** Vulva, dorsal view. Scale bars: **A–D** = 0.50 mm; **E–G** = 0.10 mm.

*Nesticella hongheensis* sp. n. is easily distinguished from the majority of the species belonging to the *brevipes*-group, with the exception of *Nesticella semicircularis* and *Nesticella lisu* sp. n., by having a wide, triangular dorsal apophysis (Da) (Fig. 16B), a rectangular distal process on the paracymbium (Dp-I) (Fig. 16D) and, for the females, by the appearance of the short scape (Fig. 17E) and the thick copulatory ducts (Fig. 17F–G). Males can be separated from those of *Nesticella semicircularis* (see Liu and Li 2013b: 521, figs 19–22) by the dark pattern, the wider dorsal apophysis (Da), by the wider distal process (Dp-I), and the stockier tegular apophysis (Tg) (Fig. 16A, 16D vs. figs 20A, 21A–C). Females of *Nesticella hongheensis* sp. n. are recognized by the black marks on the opisthosoma, the wider and much shorter scape and the shape of the copulatory ducts (Cd), strongly bent in the middle (Fig. 17A–G vs. fig. 22A–E). Males can be separated from those of *Nesticella lisu* sp. n. (see Figs 19A–D, 20A–G) by the wider tegular apophysis (Tg) and the smooth distal process (Dp-I) lacking a serrated margin and laterally compressed ranther than dorsoventrally compressed (Fig. 16A–B, D vs. Fig. 19A–B, D). Females are recognized from those of *Nesticella lisu* sp. n. by the wider and shorter scape (Sp), the thick and strongly sclerotized copulatory ducts (Cd) and by the thinner and less coiled fertilization ducts (Fd) (Fig. 17F–G vs. Fig. 20F–G).

**Figure 18.**
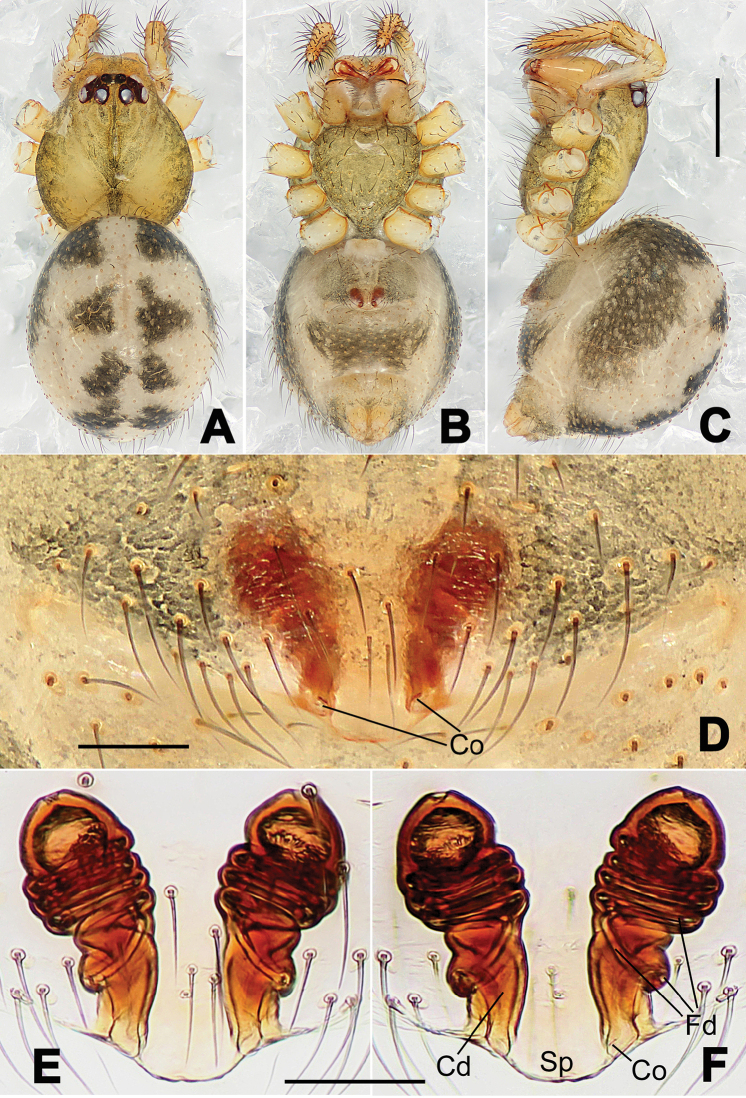
*Nesticella jingpo* sp. n., holotype (female). **A** Habitus, dorsal view **B** Ditto, ventral view **C** Ditto, lateral view **D** Epigyne, ventral view **E** Vulva, ventral view **F** Vulva, dorsal view. Scale bars: **A–C** = 0.50 mm; **D–F** = 0.10 mm.

**Figure 19.**
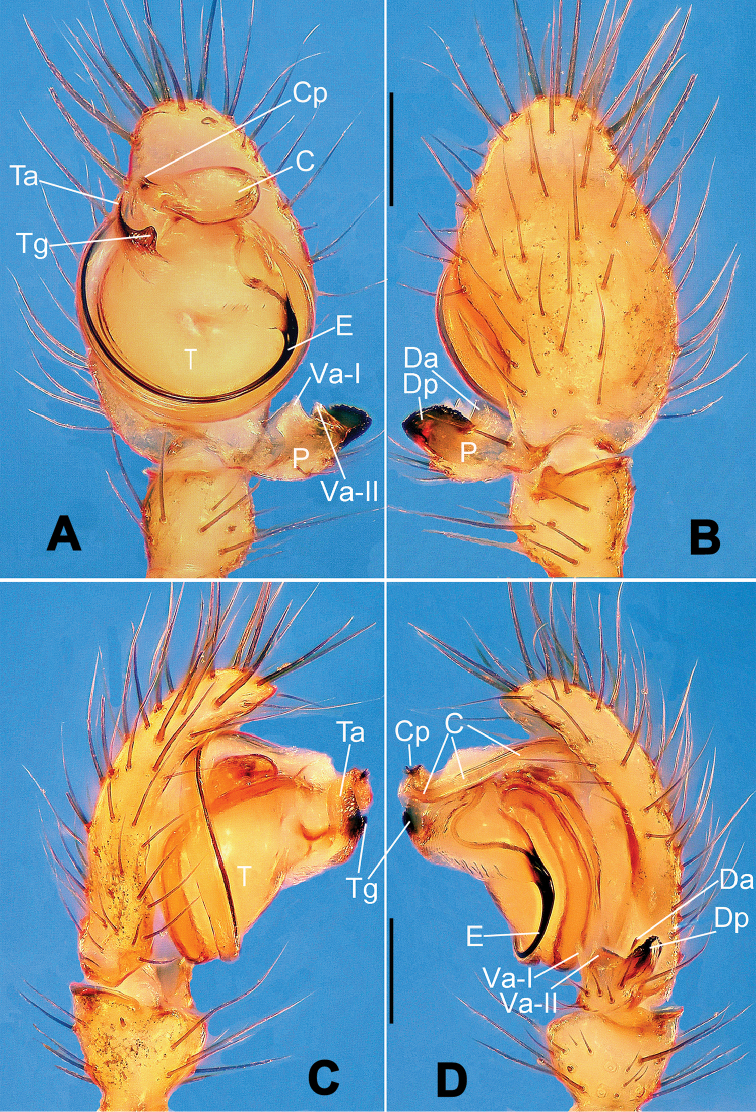
*Nesticella lisu* sp. n., holotype (male). **A** Palp, ventral view **B** Ditto, dorsal view **C** Ditto, prolateral view **D** Ditto, retrolateral view. Scale bars: 0.10 mm.

**Figure 20.**
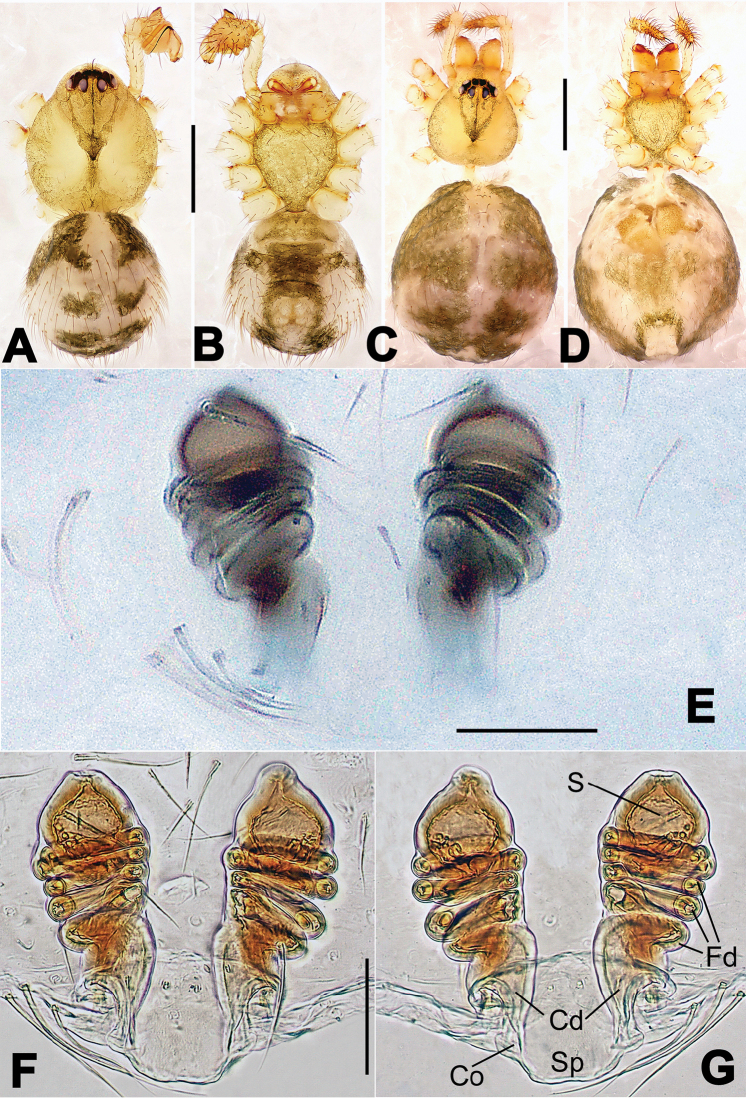
*Nesticella lisu* sp. n., holotype (male) and paratype (female). **A** Male habitus, dorsal view **B** Ditto, ventral view **C** Female habitus, dorsal view **D** Ditto, ventral view **E** Epigyne, ventral view **F** Vulva, ventral view **G** Vulva, dorsal view. Scale bars: **A–D** = 0.50 mm; **E–G** = 0.10 mm.

##### Description.

Habitus as in Fig. 17A–D. Carapace pale yellow in males, darker in females; dark in the midline, at margins and at the cephalic area. Cephalic area with several setae on the clypeus and along the cervical groove. Cervical groove and fovea distinct. Mouthparts pale yellow. Sternum greysh in males, darker in females. Legs and female palps yellowish, distally darker in each segment. Opisthosoma black with a light mark followed by three pairs of spots on the dorsal and lateral sides. The whole pattern is more evident in females.

Male palp (Fig. 16A–D): cymbial surface finely reticulated, densely covered with thick, long setae (Fig. 16B). Paracymbium with the first ventral process wide with a sharp tip, the second smaller and blunt (Fig. 16A, D). Two compact distal processes laterally compressed, Dp-I wide and strongly sclerotized, Dp-II short and stocky. Dorsal apophysis triangular, broad and flat (Fig. 16B). Terminal apophysis blunt, translucent and fingerlike (Fig. 16A, C). Tegular apophysis short and stocky (Fig. 16A). Conductor with a thin process (Fig. 16A, C).

Epigyne (Fig. 17E–G): dark with sparse setae. Scape almost absent, wide and very short (Fig. 17E–G). Spermathecae small, globular, separated by approximately 1.8 diameters (Fig. 17F–G). Fertilization ducts thin, long, reaching the spermathecae with three coils (Fig. 17G). Copulatory ducts short, thick, curved in the middle (Fig. 17F–G).

Male (holotype). Total length 2.15. Carapace 1.13 long, 1.05 wide. Opisthosoma 1.05 long, 1.02 wide. Clypeus height 0.21. Sternum 0.66 long, 0.72 wide. Leg measurements: see Appendix A.

Female (one of the paratypes). Total length 2.25. Carapace 1.18 long, 1.03 wide. Opisthosoma 1.28 long, 0.95 wide. Clypeus height 0.22. Sternum 0.72 long, 0.71 wide. Leg measurements: see Appendix A.

##### Habitat.

In humid and shaded areas, including anthropogenic habitats.

##### Distribution.

Known only from the type locality (Fig. 82).

#### Nesticella jingpo sp. n.

Taxon classificationAnimaliaAraneaeNesticidae

http://zoobank.org/71979843-866F-4758-A7E7-870F57562E8C

Figs 18 , 82

##### Type material.

Holotype ♀ (IZCAS), CHINA: Yunnan Province, Tengchong County, Gaoligongshan Mountain National Park (24.82898°N, 98.76758°E, 2177 m), 3.XII.2013, Y. Li & J.C. Liu leg.

##### Etymology.

The species is named after the Jingpo people, an ethnic minority living in the Gaoligongshan region of Yunnan Province; noun in apposition.

##### Diagnosis.

This new species is closely related to *Nesticella lisu* sp. n. (Fig. 20F–G), but can be distinguished from the latter species by the subglobular spermathecae (S) (piriform in *Nesticella lisu* sp. n.), the more compact and less coiled fertilization ducts (Fd) and the wider copulatory openings (Co) (Fig. 18E–F vs. Fig. 20F–G). The strongly coiled fertilization ducts (Fd) and the general shape of scape (Sp) and copulatory ducts (Cd) allow the separation of *Nesticella jingpo* sp. n. from all other species of the *brevipes*-group.

##### Description.

Habitus as in Fig. 18A–C. Carapace pale yellow, darker near the center and the margins. Ocular area with several setae. Cervical groove and fovea distinct. Mouthparts pale yellow. Sternum dark greyish, bearing sparse setae. Legs and female palps yellowish, distally darker in metatarsi and tarsi. Opisthosoma with four pairs of pigmented marks on the dorsal side, the first pair extended to laterals. Ventral side with a wide median band.

Epigyne (Fig. 18D–F): reddish brown surrounded by an extensive dark area (Fig. 18D). Scape short, translucent, nearly as wide as the diameter of the spermathecae (Fig. 18E). Spermathecae ovoid, separated from each other by about 1.5 diameters (Fig. 18E–F). Fertilization ducts thin and long, coiling into 4.5 loops before reaching the spermathecae (Fig. 18E–F). Copulatory ducts thick, short, basally narrower (Fig. 18F).

Female (holotype). Total length 2.18. Carapace 0.98 long, 0.89 wide. Opisthosoma 1.30 long, 1.08 wide. Clypeus height 0.17. Sternum 0.65 long, 0.62 wide. Leg measurements: see Appendix A.

Male. Unknown.

##### Habitat.

Forest leaf litter, under stone and rock.

##### Distribution.

Known only from the type locality (Fig. 82).

#### Nesticella lisu sp. n.

Taxon classificationAnimaliaAraneaeNesticidae

http://zoobank.org/4C28022E-6A17-47AA-BF28-FF83271A30B7

Figs 19 , 20 , 82

##### Type material.

Holotype ♂ and paratype 1♀ (IZCAS), CHINA: Yunnan Province, Yongde County, Qingquan Cave (23.86933°N, 98.20697°E, 1841 m), 3.VIII.2010, C. Wang, Q. Zhao & L. Lin leg.

##### Etymology.

The species is named after the Lisu people, an ethnic minority living in Yunnan Province; noun in apposition.

##### Diagnosis.

Males of *Nesticella lisu* sp. n. can be easily distinguished from those of the other species belonging to the *brevipes*-group by the squared, dorso-ventrally flat distal process of the paracymbium (Dp) with serrated margin (Fig. 19A–B, D). Females are recognized by the thick and strongly coiled fertilization ducts (Fd) and the piriform spermathecae (S) (Fig. 20F–G). Furthermore, females can be separated from those of the closely related species *Nesticella jingpo* sp. n. (see Fig. 18E–F) by the weakly sclerotized vulva, the piriform spermathecae (S), the less compact but more coiled fertilization ducts (Fd) and the smaller copulatory openings (Co) (Fig. 20F–G vs. Fig. 18E–F).

##### Description.

Habitus as in Fig. 20A–D. Carapace pale yellow and faintly pigmented near the cervical groove, the midline and the margins. Cervical groove and fovea distinct. Mouthparts yellow. Sternum with sparse long setae. Legs and female palps yellowish, distally darker in the metatarsi and tarsi. Opisthosoma light yellow with paired dorsal dark marks, the anterior pair extended to the sides as a large, round spot. Ventral side dark near the hypogastric area.

Male palp (Fig. 19A–D): paracymbium with seven dorsal setae (Fig. 19D), dorsal apophysis triangular, translucent, (Fig. 19B), ventral processes flat, Va-I wide and triangular, Va-II shorter and more pointed (Fig. 19A, D). Distal process strongly sclerotized, dorso-ventrally flattened, with a serration on the margin and lacking any ramifications. (Fig. 19A–B). Terminal apophysis fingerlike with a wrinkled surface. Tegular apophysis strongly sclerotized, small and triangular, with a textured surface (Fig. 19A, C). Conductor ending with a short process (Fig. 19A, C–D).

Epigyne (Fig. 20E–G): weakly sclerotized, covered with sparse long setae (Fig. 20E). Scape short and translucent, about three times wider than long (Fig. 20F–G). Copulatory openings small and translucent (Fig. 20G). Spermathecae piriform, dorsally pointed and separated from each other by nearly 0.8 their width (Fig. 20G). Fertilization ducts long and thick, basally constricted, reaching the spermathecae with at least four coils (Fig. 20F–G). Copulatory ducts short and translucent, distally swollen (Fig. 20G).

Male (holotype). Total length 2.14. Carapace 1.08 long, 0.97 wide. Opisthosoma 1.08 long, 1.00 wide. Clypeus height 0.18. Sternum 0.68 long, 0.62 wide. Leg measurements: see Appendix A.

Female (one of the paratypes). Total length 3.03. Carapace 1.16 long, 1.00 wide. Opisthosoma 2.09 long, 1.43 wide. Clypeus height 0.21. Sternum 0.73 long, 0.68 wide. Leg measurements: see Appendix A.

##### Habitat.

Cave.

##### Distribution.

Known only from the type locality (Fig. 82).

#### Nesticella liuzhaiensis sp. n.

Taxon classificationAnimaliaAraneaeNesticidae

http://zoobank.org/3DDB9E99-53F1-4F19-931F-9D35BE6560A5

Figs 21 , 82

##### Type material.

Holotype ♀ and paratype 1♀ (IZCAS), CHINA: Guangxi Zhuang Autonomous Region, Nandan County, Longli Village, near the Dixia River, subterranean river cave (25.27530°N, 107.44731°E, 880 m), 29.I.2015, Y. Li & X. Chen leg.

##### Etymology.

The specific name is derived from the type locality; adjective.

##### Diagnosis.

**Figure 21.**
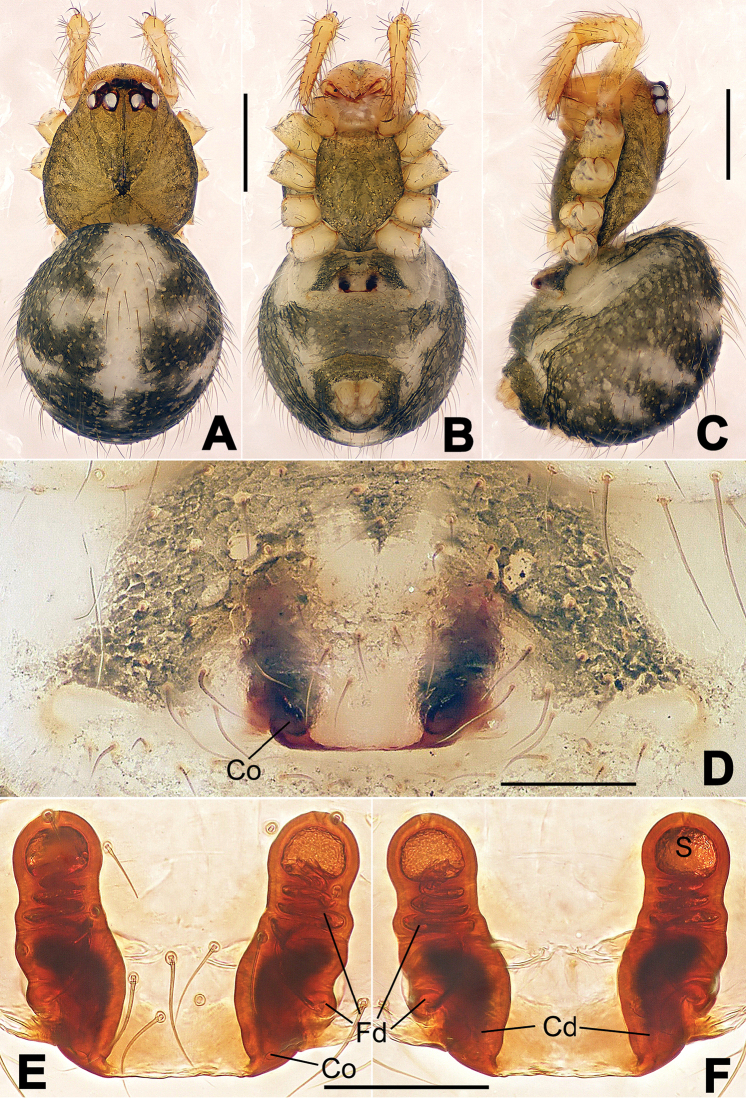
*Nesticella liuzhaiensis* sp. n., holotype (female). **A** Habitus, dorsal view **B** Ditto, ventral view **C** Ditto, lateral view **D** Epigyne, ventral view **E** Vulva, ventral view **F** Vulva, dorsal view. Scale bars: **A–C** = 0.50 mm; **D–F** = 0.10 mm.

The new species can be easily distinguished from all the others belonging to the *brevipes*-group by the very large and squared short scape, the flat posterior margin and the nearly straight copulatory ducts visible through the transparent tegument which give the epigyne a U-shape. *Nesticella robusta* sp. n. (Fig. 30E–G) and *Nesticella machadoi* from Angola (see Hubert, 1971: 75, figs 1–5) are morphologically similar to *Nesticella liuzhaiensis* sp. n. The new species can be separated from *Nesticella robusta* sp. n. by the straighter and less tortuous copulatory ducts (Cd) (Fig. 21F vs. Fig. 30G) and from *Nesticella machadoi* by the smaller spermathecae (S), the more widely separated copulatory ducts (Cd) and the straight rather than concave posterior margin of the scape (Fig. 21G vs. fig. 3). Furthermore, in *Nesticella liuzhaiensis* sp. n., the PME are equal to ALE in size while the PME are larger than the ALE in *Nesticella machadoi* (Fig. 21A vs. fig. 1).

##### Description.

Habitus as in Fig. 21A–C. Carapace dark yellow, with extended dark areas near the center and the margins. Cephalic area with several long setae at the midline and along the cervical groove. Cervical groove and fovea distinct. Thoracic area with faint radial furrows. Mouthparts brown-yellowish. Sternum dark, with long setae. Legs and female palps yellowish, tarsus distally darker. Opisthosoma light yellow with paired dark spots on the dorsal side, often merged with each other and forming a lighter cross-shaped mark on the background.

Epigyne (Fig. 21D–F): dark, posterior margin truncated and straight (Fig. 21D). Scape strongly squared, wide and short (Fig. 21E–F). Copulatory openings near the posterior margin of the epigyne, separated from each other by about two times the spermathecal diameters (Fig. 21D–E). Spermathecae small and ovoid, separated from each other by about two diameters (Fig. 21F). Fertilization ducts thin and long, reaching the spermathecae with 3.5 coils (Fig. 21F). Copulatory ducts thick and short, swollen and nearly straight (Fig. 21E).

Female (holotype). Total length 2.13. Carapace 1.00 long, 0.87 wide. Opisthosoma 1.36 long, 1.11 wide. Clypeus height 0.15. Sternum 0.64 long, 0.49 wide. Leg measurements: see Appendix A.

Male. Unknown.

##### Habitat.

Cave.

##### Distribution.

Known only from the type locality (Fig. 82).

#### Nesticella mollicula

Taxon classificationAnimaliaAraneaeNesticidae

(Thorell, 1898)

Erigone mollicula Thorell, 1898: 318 (♂♀).
Howaia inthanoni Lehtinen & Saaristo, 1980: 55, fig. 13 (♀), **syn. n.**
Nesticella mollicula : Tanasevitch 2010: 107.

##### Remarks.

The authors had the opportunity to see photos of the type material of *Nesticella mollicula* from Myanmar preserved in the collection of the Museo di Storia Naturale di Genova, Italy. The morphology of the species is comparable with *Nesticella inthanoni* described from Thailand by Lehtinen and Saaristo (1980) on the basis of a single female; the specimens have the very short and wide, squared scape, rarely seen in the *Nesticella* species of the *brevipes*-group. The small, almost round spermathecae and the long copulatory ducts that are slightly curved outward and visible through the tegument are also the same. Although described from two different countries, the type localities are close to the common border between Myanmar and Thailand and are approximately 200 km apart. Based on the evidence mentioned above, we propose the synonymy of *Nesticella inthanoni* Lehtinen & Saaristo, 1980 with *Nesticella mollicula* (Thorell, 1898).

##### Distribution.

Myanmar, Thailand.

#### Nesticella nandanensis sp. n.

Taxon classificationAnimaliaAraneaeNesticidae

http://zoobank.org/19CB4C51-CC10-4BDF-9B1A-C5CBB1CF00AE

Figs 22 , 23 , 82

##### Type material.

Holotype ♂ and paratypes 1♂4♀ (IZCAS), CHINA: Guangxi Zhuang Autonomous Region, Hechi Prefecture, Nandan County, Encun Village, Encun Cave (25.08342°N, 107.59106°E, 635 m), 30.I.2015, X. Chen & Y. Li leg.

##### Etymology.

The specific name is derived from the type locality; adjective.

##### Diagnosis.

**Figure 22.**
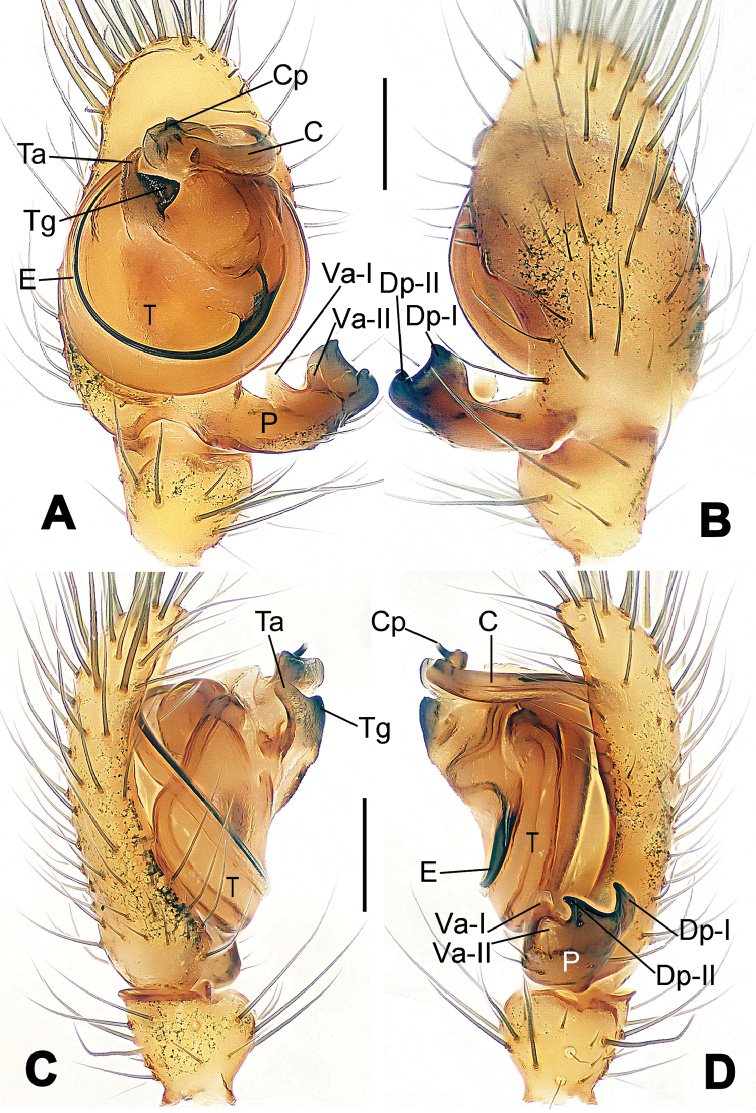
*Nesticella nandanensis* sp. n., holotype (male). **A** Palp, ventral view **B** Ditto, dorsal view **C** Ditto, prolateral view **D** Ditto, retrolateral view. Scale bars: 0.10 mm.

**Figure 23.**
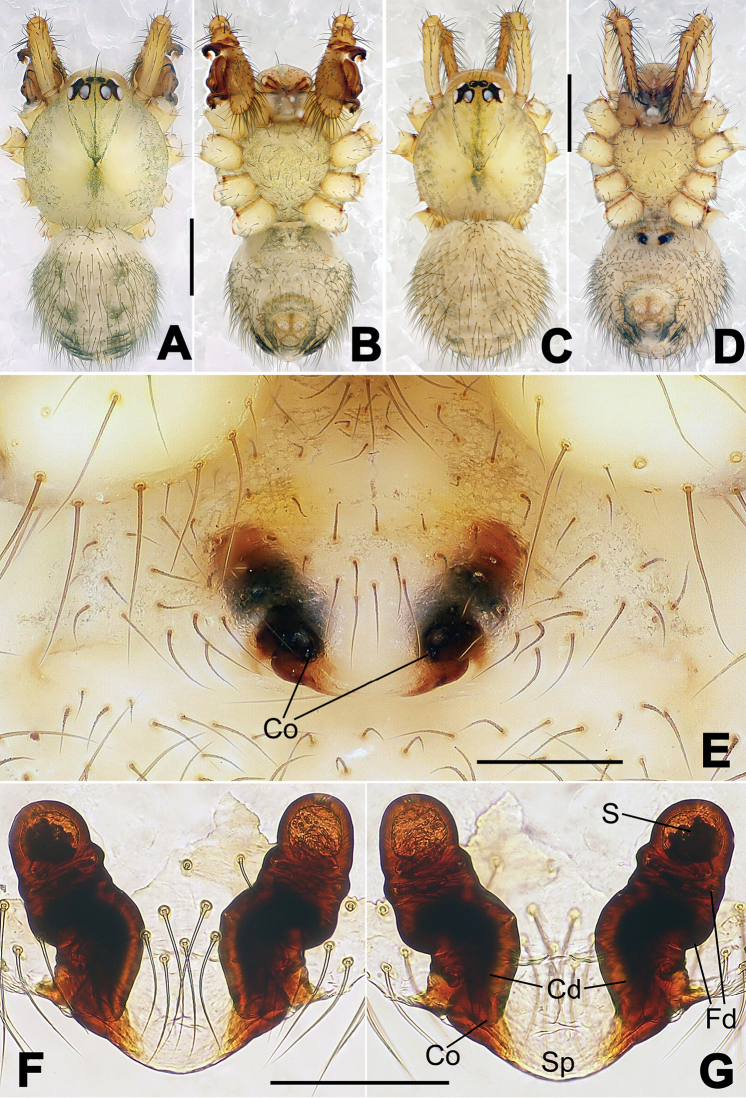
*Nesticella nandanensis* sp. n., holotype (male) and paratype (female). **A** Male habitus, dorsal view **B** Ditto, ventral view **C** Female habitus, dorsal view **D** Ditto, ventral view **E** Epigyne, ventral view **F** Vulva, ventral view **G** Vulva, dorsal view. Scale bars: **A–D** = 0.50 mm; **E–G** = 0.10 mm.

*Nesticella nandanensis* is closely related to *Nesticella baiseensis* sp. n., *Nesticella songi*, and *Nesticella verticalis*. It can be separated from *Nesticella baiseensis* sp. n. by the shorter, stockier tegular apophysis (Tg), the smaller angle formed by the distal processes (Dp) of the paracymbium (Fig. 22A vs. Fig. 8A) and by the larger scape (Sp) and the wider space between the spermathecae (S) (Fig. 23G vs. Fig. 9G). It is recognized from *Nesticella songi* by the wider triangular tegular apophysis (Tg) bearing a sharper tip, the smaller process of the conductor (Cp), the shorter process I of the ventral apophysis (Va-I), the more pointed ventral process II (Va-II) (Fig. 22A, D vs. Fig. 33A, D), the wider distance between the spermathecae (S) and the ducts (Cd and Fd) oriented outward rather than almost parallel (Fig. 23G vs. Fig. 34G). The new species can be distinguished from *Nesticella verticalis* (see Liu and Li 2013b: 522, figs 27–30) by the sharper tegular apophysis (Tg), the wider and more squared distal process I of the paracymbium (Dp-I) when observed dorsally, the shorter and more compact copulatory ducts (Cd), the shorter scape (Sp) and the spermathecae (S) being more oriented outward (Figs 22A, D, 23G vs. figs 27B, 29B, D, 28A, D, 30C). The absence of a dorsal apophysis (Da), the different shape of the distal processes of the paracymbim (Dp) (especially the Dp-I) and the shape of the tegular apophysis (Tg) help to distinguish males of *Nesticella nandanensis* sp. n. from those of all the other species of the group.

##### Description.

Habitus as in Fig. 23A–D. Carapace pale yellow to beige, with faint dark areas around the cervical furrow, the fovea and near the margins. Cervical groove and fovea distinct. Mouthparts yellow. Sternum yellowish with sparse setae. Leg uniformly yellowish, distally darker tibiae, metatarsi and tarsi. Opisthosoma pale greyish, faintly darker on the dorsal and posterior side and around the spinnerets.

Male palp (Fig. 22A–D): paracymbium with laminar, weakly sclerotized ventral processes, Va-I long and sharp, Va-II shorter and blunt (Fig. 22A). Distal process strongly sclerotized divided into two sharp branches (Fig. 22A–B, D). Tegular apophysis triangular, strongly sclerotized, with a rugose surface (Fig. 22A). Conductor distally twisted, ending with a sclerotized short and thin process (Fig. 22A, C–D).

Epigyne (Fig. 23E–G): yellowish. Scape broad, translucent with a convex posterior margin and slightly protruding outside the epigynal posterior margin (Fig. 23F–G). Spermathecae small, globular, separated by approximately 2.5 diameters (Fig. 23F–G). Fertilization ducts thin, reaching the spermathecae with several coils (Fig. 23G), copulatory ducts thick, bent in the middle.

Male (holotype). Total length 2.56. Carapace 1.34 long, 1.25 wide. Opisthosoma 1.30 long, 1.06 wide. Clypeus height 0.21. Sternum 0.80 long, 0.76 wide. Leg measurements: see Appendix A.

Female (one of the paratypes). Total length 2.91. Carapace 1.44 long, 1.30 wide. Opisthosoma 1.65 long, 1.28 wide. Clypeus height 0.23. Sternum 0.86 long, 0.84 wide. Leg measurements: see Appendix A.

##### Habitat.

Cave.

##### Distribution.

Known only from the type locality (Fig. 82).

#### Nesticella odonta

Taxon classificationAnimaliaAraneaeNesticidae

(Chen, 1984)

Figs 24 , 25 , 82

Nesticus odontus Chen, 1984: 34, figs 1–6 (♂♀).
Nesticella odonta : Platnick 1989: 184.
Nesticus odontus : Chen and Zhang 1991: 158, fig. 156.1–6 (♂♀).
Nesticella odonta : Song et al. 1999: 86, figs 12E, 37E–F, 37I (♂♀).
Nesticella odonta : Yin et al. 2012: 237, fig. 75a–d (♀).
Nesticella taiwan Tso & Yoshida, 2000: 13, figs 1–6 (♂), **syn. n.**

##### Material examined.

Paratypes of *Nesticella odonta* 1♂ and 2♀ (IZCAS), CHINA: Zhejiang Province, Hangzhou City, Qixialing Hill, Huanglong Cave, 18.V.1981, Z. Chen leg.

##### Diagnosis.

**Figure 24.**
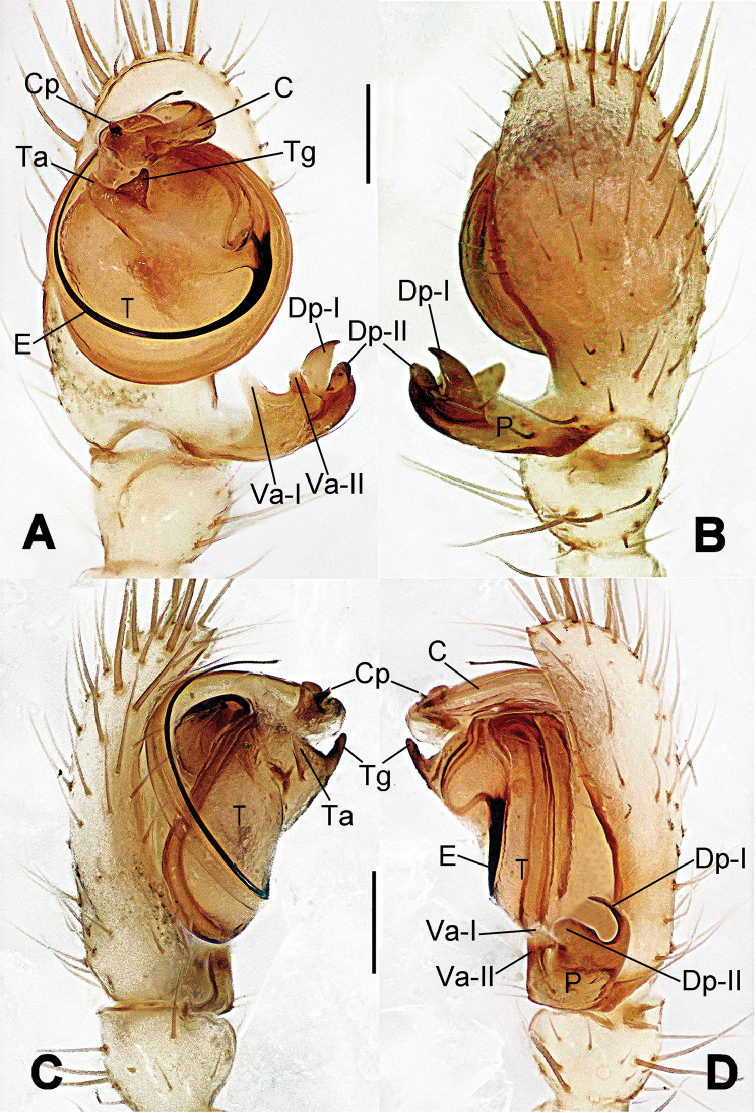
*Nesticella odonta*, paratype (male). **A** Palp, ventral view **B** Ditto, dorsal view **C** Ditto, prolateral view **D** Ditto, retrolateral view. Scale bars: 0.10 mm.

**Figure 25.**
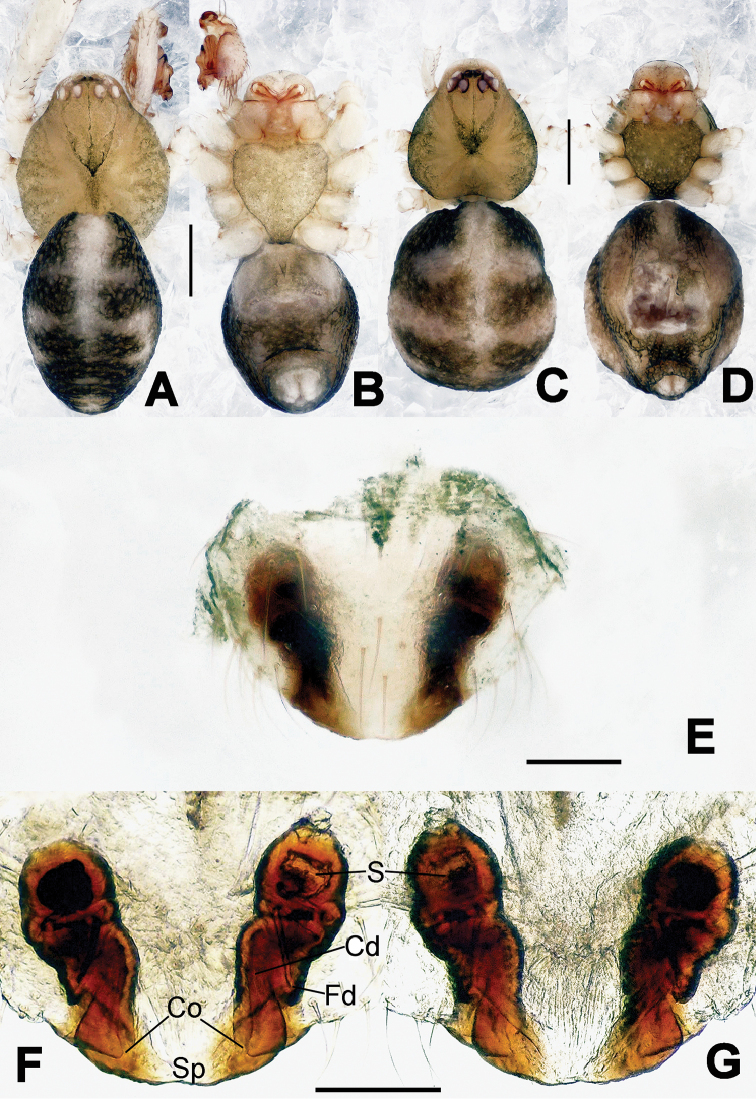
*Nesticella odonta*, paratypes (male and female). **A** Male habitus, dorsal view **B** Ditto, ventral view **C** Female habitus, dorsal view **D** Ditto, ventral view **E** Epigyne, ventral view **F** Vulva, dorsal view **G** Vulva, ventral. Scale bars: **A–D** = 0.50 mm; **E–G** = 0.10 mm.

This species can be easily recognized from the others belonging to the *brevipes*-group, with the exception of *Nesticella xiongmao* sp. n. (Fig. 35A–D, 36A–G) and *Nesticella chongqing* sp. n. (Fig. 12A–F) by the sharp, hook-like distal process I of the paracymbium (Dp-I) that is bent downward (Fig. 24A, D), the lobed distal process II (Dp-II) (Fig. 24D) and the sharp tegular apophysis (Tg) (Fig. 24A, C–D). Females can be distinguished by the short scape (Sp) with a flat posterior margin (Fig. 25E) and by the compact, almost straight ducts (Fd and Cd) (Fig. 25F–G). Males of *Nesticella odonta* can be separated from those of *Nesticella xiongmao* sp. n. by the wider distal process I of the paracymbium (Dp-I) (Fig. 24A vs. Fig. 35A) and the broader and more lobed distal process II (Dp-II) (Fig. 24D vs. Fig. 35D). Females are distinguished by the shorter, squared scape (Sp) with a flat rather than rounded posterior margin (Fig. 25E vs. Fig. 36E), the folded rather than straight copulatory ducts (Cd) and the greater distance between the spermathecae (S) (Fig. 25F–G vs. Fig. 36F–G). The same combination of characters allows females of *Nesticella odonta* to be distinguished from those of *Nesticella chongqing* sp. n.

##### Description.

See Chen (1984).

##### Habitat.

Forest leaf litter, cave.

##### Distribution.

South China (Fig. 82).

##### Remarks.

The type material of *Nesticella taiwan* Tso & Yoshida, 2000 was not found in the Department of Biology at Tung Hai University, Taichung. However, the drawings of the male holotype by Tso & Yoshida clearly show the diagnostic characters of the species: the hook-like, pointed distal process I, the lobed distal process II, the general shape of the paracymbium when viewed ventrally and the pointed, sclerotized tegular apophysis. All these characters are compatible with the the palpal morphology of *Nesticella odonta* from mainland China (compare Fig. 24A–D and Tso and Yoshida 2000: 14, figs 4–6). Furthermore, *Nesticella odonta* seems to be a widely distributed species in throughout southern China, and its presence in Taiwan is very likely. In addition, the in progress morphological and molecular analysis of newly collected male and female specimens from the type locality of *Nesticella taiwan* demonstrate that the females illustrated by Tso & Yoshida as *Nesticella taiwan* were mismatched, belonging instead to a different species, *Nesticella kaohsiungensis* sp. n.. Based on the above mentioned evidence, we propose the synonymy of *Nesticella taiwan* Tso & Yoshida, 2000 with *Nesticella odonta* (Chen, 1984).

#### Nesticella qiaoqiensis sp. n.

Taxon classificationAnimaliaAraneaeNesticidae

http://zoobank.org/DC65BB7B-5306-41DF-9264-9128B1535374

Figs 26 , 82

##### Type material.

Holotype ♀ (IZCAS), CHINA: Sichuan Province, Baoxing County, Qiaoqi Town, Zeyin Village (30.73852°N, 102.74850°E, 2247 m), cave without a name, 7.V.2015, Y. Li & X. Chen leg.

##### Etymology.

The specific name is derived from the type locality; adjective.

##### Diagnosis.

**Figure 26.**
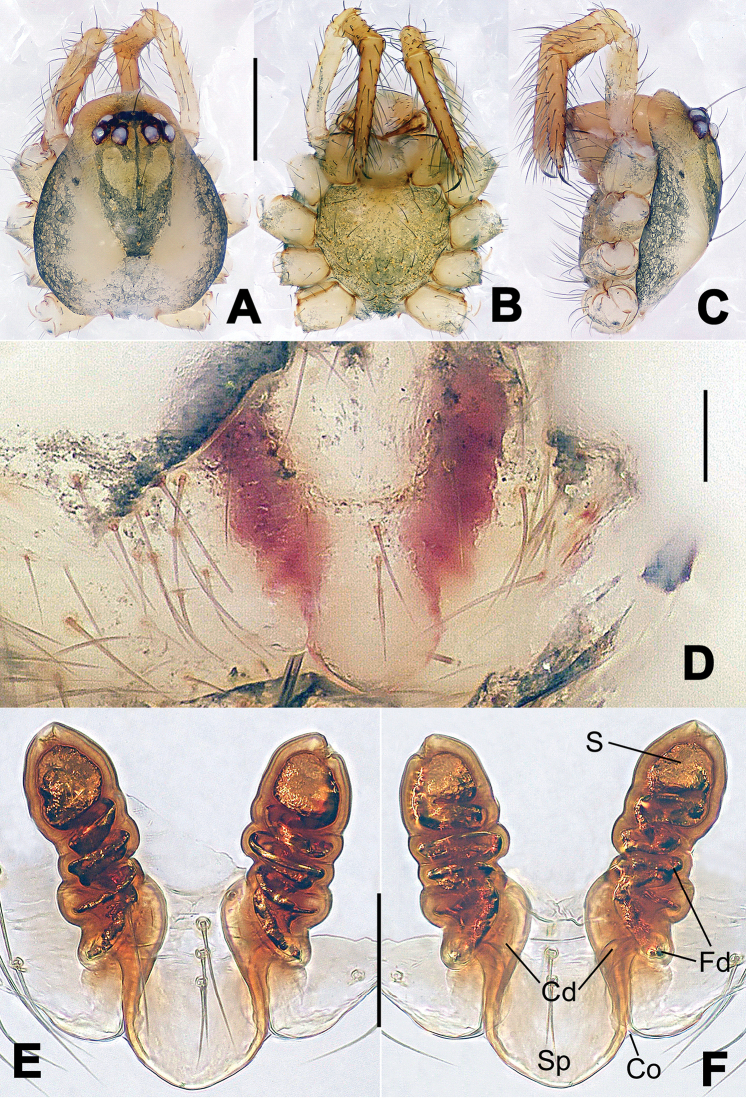
*Nesticella qiaoqiensis* sp. n., holotype (female). **A** Prosoma, dorsal view **B** Ditto, ventral view **C** Ditto, lateral view **D** Epigyne, ventral view **E** Vulva, ventral view **F** Vulva, dorsal view. Scale bars: **A–C** = 0.50 mm; **D–F** = 0.10 mm.

This new species is similar to *Nesticella gracilenta* (see Liu and Li 2013b: 512, figs 13–16), *Nesticella xixia* sp. n. (Figs 37A–D, 38E–G), *Nesticella semicircularis* (see Liu and Li 2013b: 521, figs 19–22) and *Nesticella shanlinensis* (see Liu and Li 2013b: 521–522, figs 23–26) for the general shape of vulva. It can be separated from the first three species by the wider and longer scape (Sp) (Fig. 26D), the longer and straighter ducts (Fd and Cd) (Fig. 26F), the more coiled fertilization ducts (Fd) (Fig. 26F) and the darker carapace (Fig. 26A, C). *Nesticella qiaoqiiensis* sp. n. can be distinguished from *Nesticella shanlinensis* by the longer ducts provided with more coils (Fig. 26F vs. figs 24C, 26D). The wide, lobed scape, the short copulatory ducts and the general shape of the strongly coiled fertilization ducts (Fd) (Fig. 26E–F) easily separate the new species from all other species of the *brevipes*-group.

##### Description.

Habitus as in Fig. 26A–C. Carapace pale yellow, dark at margins and in the center. Cervical groove and fovea indistinct. Mouthparts yellowish brown. Sternum yellow, strongly pigmented, with sparse long setae. Female palps and legs yellow, proximally lighter in femora. Opisthosoma greyish with irregular dark marks.

Epigyne (Fig. 26D–F): weakly sclerotized, translucent. Reddish brown vulva (Fig. 26D). Scape short, flat and lobed, with almost round margins, translucent, laterally sclerotized, protruding beyond the epigynal posterior margin (Fig. 26D). Spermathecae ovate, separated by about 1.6 diameters (Fig. 26F). Fertilization ducts long and strongly coiled, reaching the spermathecae with four loops. Copulatory ducts short, mesially swollen and basally narrower (Fig. 26E–F).

Female (holotype). Total length 2.77. Carapace 1.09 long, 0.96 wide. Opisthosoma 1.76 long, 1.55 wide. Clypeus height 0.19. Sternum 0.58 long, 0.65 wide. Leg measurements: see Appendix A.

Male. Unknown.

##### Habitat.

Cave.

##### Distribution.

Known only from the type locality (Fig. 82).

#### Nesticella qiongensis sp. n.

Taxon classificationAnimaliaAraneaeNesticidae

http://zoobank.org/65E4DC02-B722-4692-B71B-DE0F14FD5429

Figs 27 , 28 , 82

##### Type material.

Holotype ♂ and paratypes 2♀ (IZCAS), CHINA: Hainan Province, Diaoluoshan Mountain National Nature Reserve, Diaoluoshan Holiday Village (18.72943°N, 109.86358°E, 1010 m), 15.VIII.2007, S. Li leg.

##### Etymology.

The specific name derives from the Chinese pinyin for “Qiong”, and refers to an alias name of Hainan Island where the species was collected; adjective.

##### Diagnosis.

**Figure 27.**
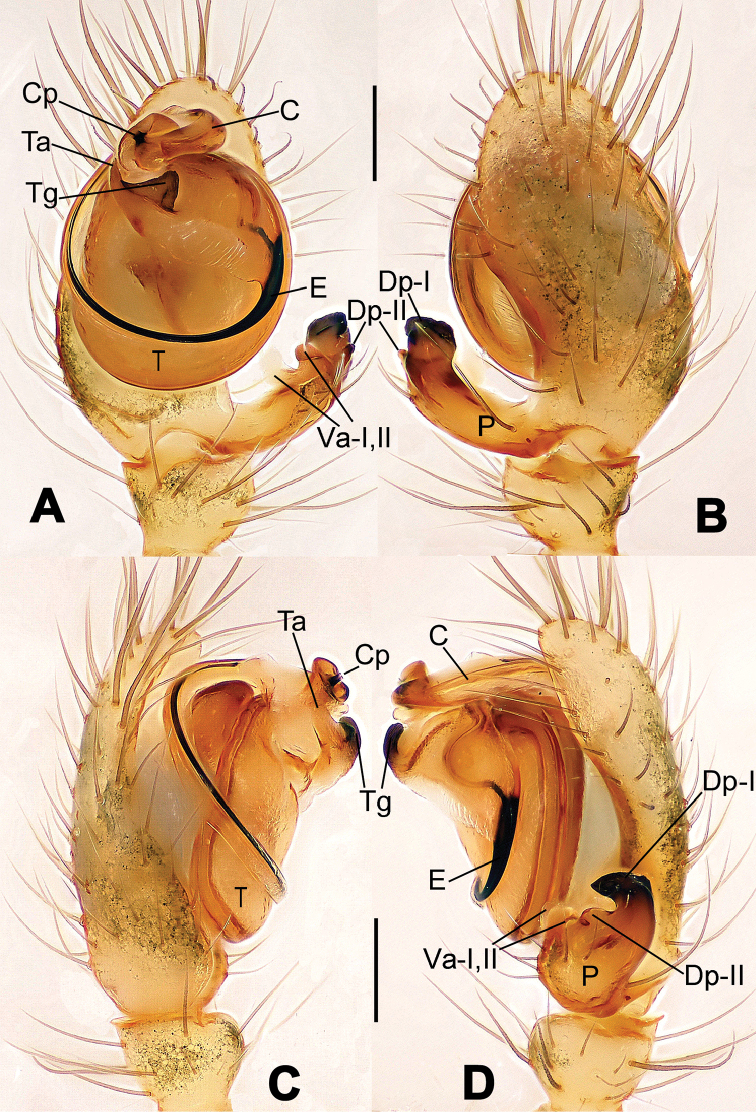
*Nesticella qiongensis* sp. n., holotype (male). **A** Palp, ventral view **B** Ditto, dorsal view **C** Ditto, prolateral view **D** Ditto, retrolateral view. Scale bars: 0.10 mm.

**Figure 28.**
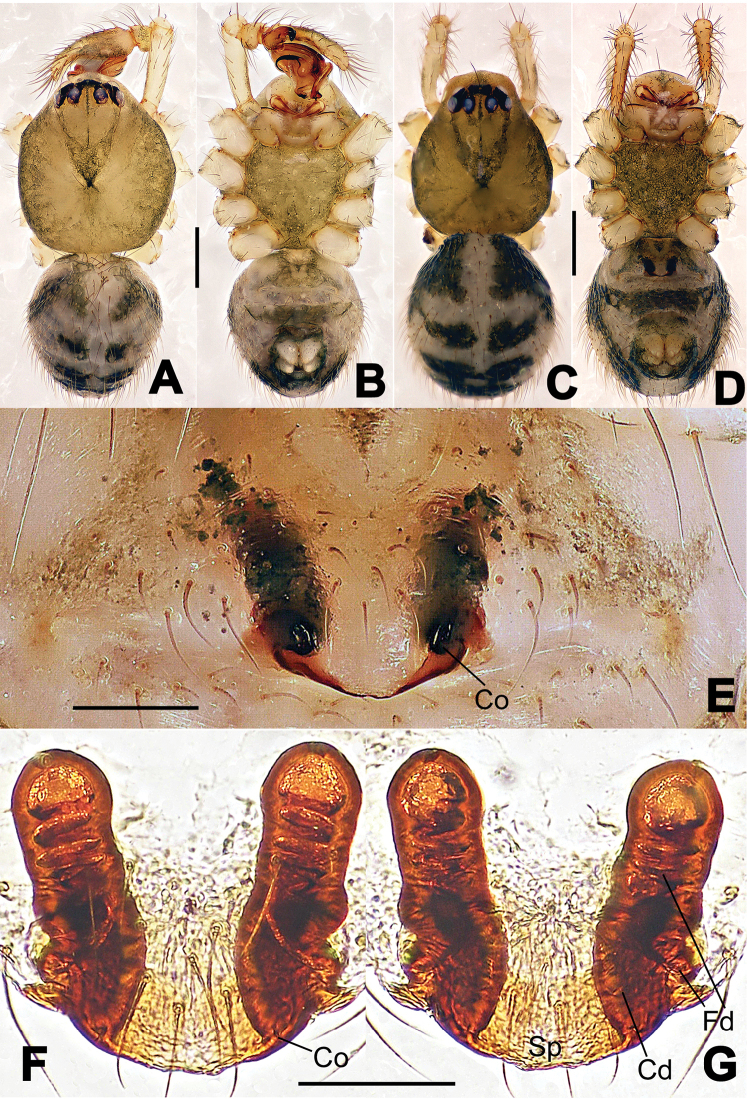
*Nesticella qiongensis* sp. n., holotype (male) and paratype (female). **A** Male habitus, dorsal view **B** Ditto, ventral view **C** Female habitus, dorsal view **D** Ditto, ventral view **E** Epigyne, ventral view **F** Vulva, ventral view **G** Vulva, dorsal view. Scale bars: **A–D** = 0.50 mm; **E–G** = 0.10 mm.

**Figure 29.**
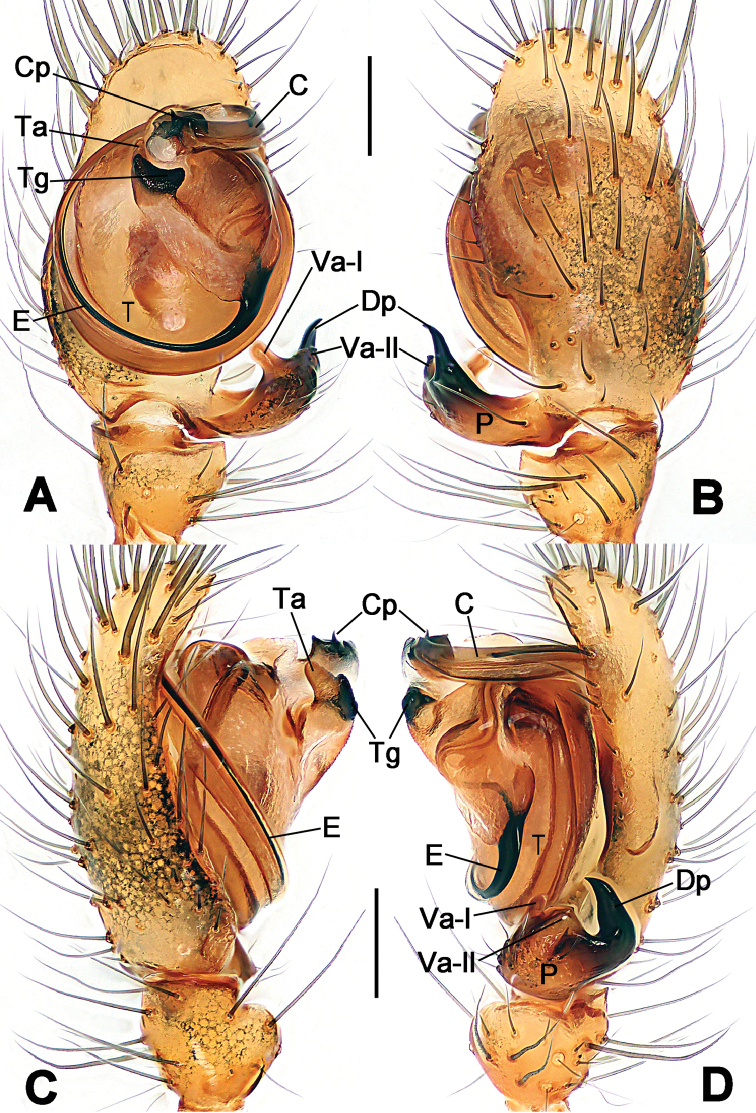
*Nesticella robusta* sp. n., holotype (male). **A** Palp, ventral view **B** Ditto, dorsal view **C** Ditto, prolateral view **D** Ditto, retrolateral view. Scale bars: 0.10 mm.

**Figure 30.**
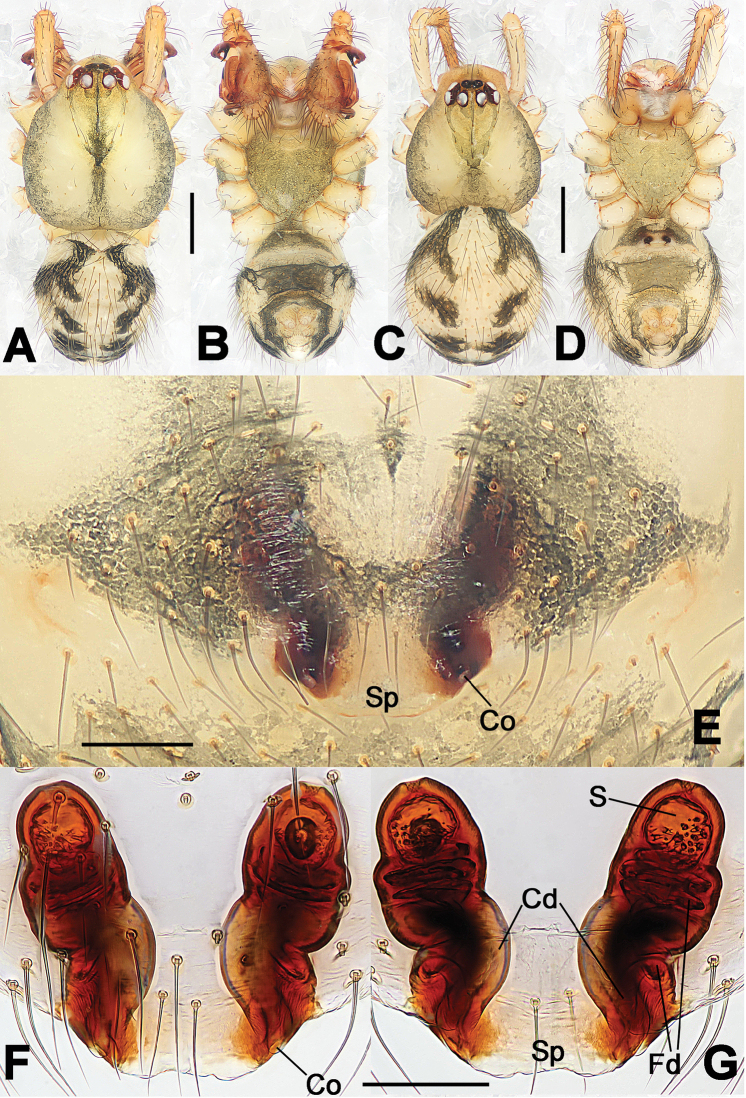
*Nesticella robusta* sp. n., holotype (male) and paratype (female). **A** Male habitus, dorsal view **B** Ditto, ventral view **C** Female habitus, dorsal view **D** Ditto, ventral view **E** Epigyne, ventral view **F** Vulva, ventral view **G** Vulva, dorsal view. Scale bars: **A–D** = 0.50 mm; **E–G** = 0.10 mm.

*Nesticella qiongensis* sp. n. can be distinguished from the majority of the species belonging to the *brevipes*-group, with the exception of *Nesticella falcata*, *Nesticella liuzhaiensis* sp. n. and *Nesticella robusta* sp. n., by the absence of a dorsal apophysis (Da) (Fig. 27B) and the stocky, hooked distal process I (Dp-I) (Fig. 27D) in the males; by the wide and short scape (Sp) with a convex posterior margin and the almost straight ducts (Fd and Cd) in the females (Fig. 28G). It can be differentiated from *Nesticella falcata* (see Liu and Li 2013b: 511, figs 9–12) by the shorter and blunt distal process II (Dp-II), the stockier distal process I (Dp-I) (Fig. 27D vs. figs 9B, 11D), the wider, triangular tegular apophysis (Fig. 27A vs. figs 9A, 11B), and by the wider spermathecae and the straighter, tubular ducts (Fig. 28F–G vs. figs 10B–C, 12B–C). It can be separated from *Nesticella liuzhaiensis* sp. n. by the narrower scape with a convex posterior margin (straight in *Nesticella liuzhaiensis* sp. n.) and by the shorter distance between the spermathecae and the copulatory ducts (Fig. 28G vs. Fig. 21F). It can be distinguished from *Nesticella robusta* sp. n. by the thicker distal process I (Dp-I), the longer ventral process II (Va-II), strongly reduced in *Nesticella robusta* sp. n. (Fig. 27A–B, D vs. Fig. 29A–B, D), and by the straighter copulatory ducts (Fig. 28G vs. Fig. 30G).

##### Description.

Habitus as in Fig. 28A–D. Carapace pale yellow in males, darker in females, with a dark color around the margins and at the cephalic area. Thoracic area marginally pigmented. Several sparse setae present at the ocular area and along the cervical groove. Cervical groove distinct. Fovea deep. Mouthparts pale yellow in the male, darker in the female. Sternum lighter in the male than in the female. Legs and female palps yellowish, distally darker in tibiae, metatarsi and tarsi. Opisthosoma yellowish with paired black marks on the dorsal side, partially merged each other; the dark spots are extended to the lateral and ventral sides. Darker and more evident pattern in females. Spinnerets and colulus pale yellow in males, darker in females.

Male palp (Fig. 27A–D): paracymbium with Va-I broad and laminar, ending in a rounded tip, Va-II stout and nodular (Fig. 27A). Two distal processes, Dp-I massive, strongly sclerotized and bent downward, Dp-II short and fingerlike (Fig. 27A–B, D). Terminal apophysis blunt, finger-shaped (Fig. 27A, C). Tegular apophysis wide, triangular and strongly sclerotized (Fig. 27A). Conductor distally twisted, surrounding a small, spur-shaped, sclerotized process (Fig. 27A, C).

Epigyne (Fig. 28E–G): strongly sclerotized (Fig. 28E). Scape very short and wide, marginally sclerotized, with a convex posterior margin (Fig. 28E). Spermathecae ovate, separated by about 1.7 diameters (Fig. 28F–G). Fertilization ducts thin and long, with approximately four coils before reaching the spermathecae (Fig. 28G). Copulatory ducts short and thick, almost straight and only slightly bent outward (Fig. 28F).

Male (holotype). Total length 1.88. Carapace 1.04 long, 0.92 wide. Opisthosoma 0.84 long, 0.79 wide. Clypeus height 0.17. Sternum 0.60 long, 0.59 wide. Leg measurements: see Appendix A.

Female (one of the paratypes). Total length 2.18. Carapace 1.09 long, 0.94 wide. Opisthosoma 1.16 long, 0.92 wide. Clypeus height 0.18. Sternum 0.63 long, 0.63 wide. Leg measurements: see Appendix A.

##### Habitat.

Forest leaf litter.

##### Distribution.

Known only from the type locality (Fig. 82).

#### Nesticella robusta sp. n.

Taxon classificationAnimaliaAraneaeNesticidae

http://zoobank.org/3CE8D60E-DB53-4385-8F9E-AE2C11EA60A6

Figs 29 , 30 , 82

##### Type material.

Holotype ♂ and paratype 1♀ (IZCAS), CHINA: Hunan Province, Yuanling County, Qixian Cave (28.44029°N, 110.56210°E, 243 m), 31.III.2016, Y. Li & Z. Chen leg.

##### Etymology.

The specific name is derived from the Latin word “*robustus*” = robust, sturdy, and refers to the strong distal process of the paracymbium in the male; adjective.

##### Diagnosis.

Males of *Nesticella robusta* sp. n. can be easily recognized by the single, thick and sturdy distal process of the paracymbium (Dp) with a beak-like shape and the very short ventral process II (Va-II) that has no similarities to any other species of the *brevipes*-group. The morphologically closest species is *Nesticella qiongensis* sp. n. from which *Nesticella robusta* sp. n. can be separated by the slimmer and sharper distal process of the paracymbium (Dp) and the shorter ventral process II (Va-II) (Fig. 29A, B, D). Females are distinguished from those of all the other species, with the exception of *Nesticella liuzhaiensis* sp. n. and *Nesticella qiongensis* sp. n., by the short and squared scape with a flat posterior border. *Nesticella robusta* sp. n. is separated from the two closely related species by the less straight copulatory ducts (Cd) (Fig. 30G vs. Fig. 21F vs. Fig. 28G).

##### Description.

Habitus as in Fig. 30A–D. Carapace pale yellow, dark around the margin, the median line and the cervical groove. Cervical groove and fovea distinct. Mouthparts light brown-yellowish. Sternum yellowish with light dark pigmentation, darker in the male. Legs and female palps yellowish, distally darker in tibiae, metatarsi and tarsi. Opisthosoma yellowish with paired black marks on the dorsal side, partially extended to the lateral and ventral sides and merged to each other near the spinnerets. Ventrally with a wide, dark area in the middle.

Male palp (Fig. 29A–D): paracymbium well-developed, Va-I long and finger-like, Va-II strongly reduced (Fig. 29A, D). Distal process sturdy, strongly sclerotized and ending in a sharp tip (Fig. 29B, D). Terminal apophysis short and blunt (Fig. 29A, C). Tegular apophysis strongly sclerotized, triangular, with a rugose surface (Fig. 29A). Conductor distally twisted with a tiny, pointed process (Fig. 29A, C–D).

Epigyne (Fig. 30E–G): darkish (Fig. 30E). Scape wide and squared with a translucent posterior margin (Fig. 30F–G). Spermathecae small, ovate, separated from each other by about 1.5 times their diameters (Fig. 30G). Fertilization ducts long and thin, reaching the spermathecae with approximately 2.5 loops (Fig. 30F–G). Copulatory ducts short and thick, slightly bent outward (Fig. 30F–G).

Male (holotype). Total length 1.98. Carapace 1.14 long, 1.02 wide. Opisthosoma 0.90 long, 0.88 wide. Clypeus height 0.17. Sternum 0.61 long, 0.58 wide. Leg measurements: see Appendix A.

Female (one of the paratypes). Total length 2.16. Carapace 1.07 long, 0.96 wide. Opisthosoma 1.15 long, 0.96 wide. Clypeus height 0.18. Sternum 0.60 long, 0.58 wide. Leg measurements: see Appendix A.

##### Habitat.

Cave.

##### Distribution.

Known only from the type locality (Fig. 82).

#### Nesticella sanchaheensis sp. n.

Taxon classificationAnimaliaAraneaeNesticidae

http://zoobank.org/A74B0278-2C73-40F0-B083-42BAF3C85771

Figs 31 , 32 , 82

##### Type material.

Holotype ♂ and paratypes 3♀ (IZCAS), CHINA: Guizhou Province, Libo County, Jialiang Town, Sanchahe Village, Sanchahe Cave (25.53333°N, 107.70000°E, 877 m), 16.III.2011, C. Wang & L. Lin leg.

##### Etymology.

The specific name is derived from the type locality; adjective.

##### Diagnosis.

**Figure 31.**
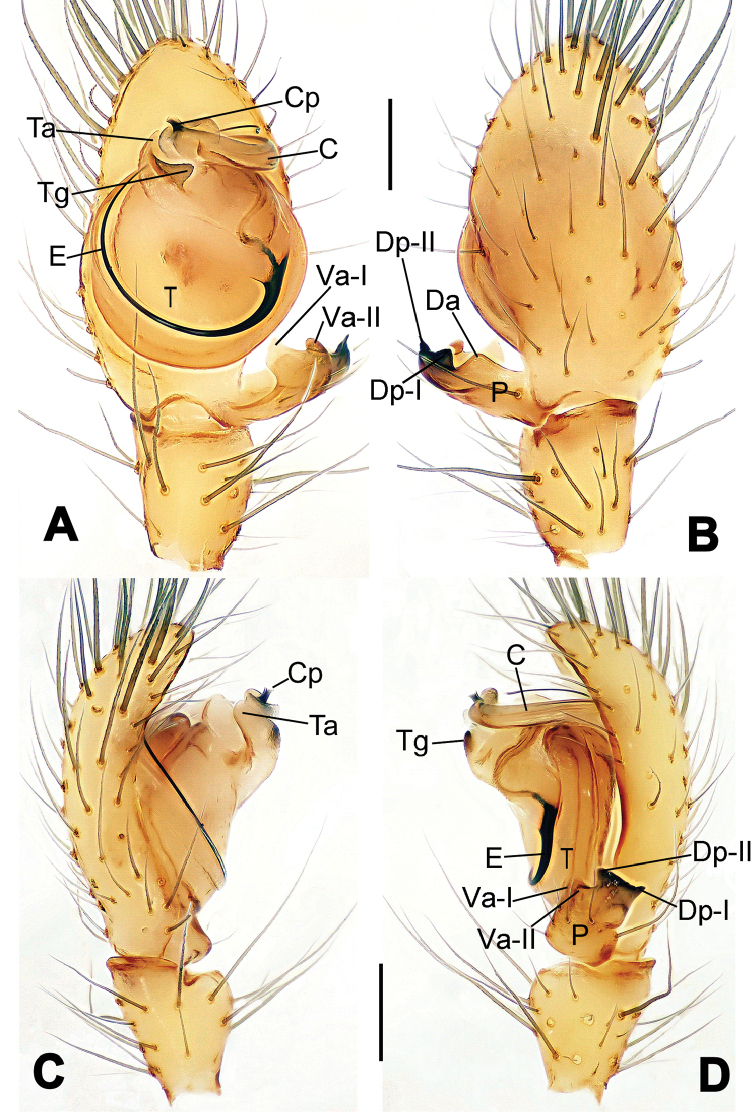
*Nesticella sanchaheensis* sp. n., holotype (male). **A** Palp, ventral view **B** Ditto, dorsal view **C** Ditto, prolateral view **D** Ditto, retrolateral view. Scale bars: 0.10 mm.

**Figure 32.**
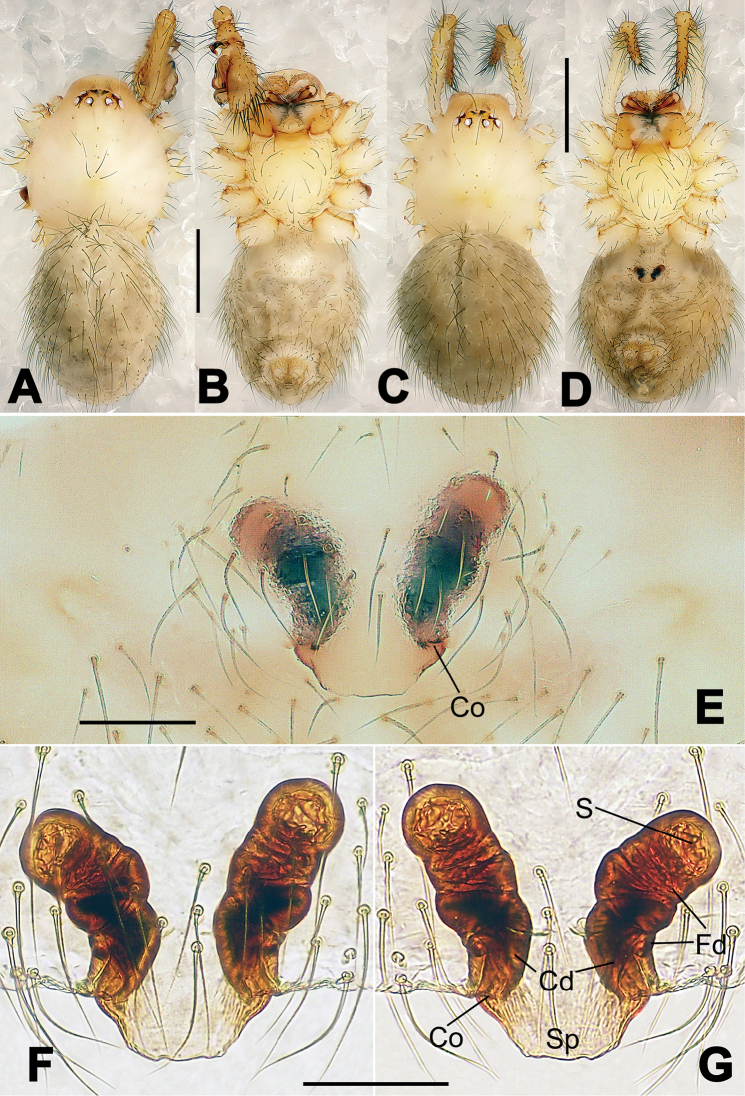
*Nesticella sanchaheensis* sp. n., holotype (male) and paratype (female). **A** Male habitus, dorsal view **B** Ditto, ventral view **C** Female habitus, dorsal view **D** Ditto, ventral view **E** Epigyne, ventral view **F** Vulva, ventral view **G** Vulva, dorsal view. Scale bars: **A–D** = 0.50 mm; **E–G** = 0.10 mm.

The new species is closely related to *Nesticella gazuida* sp. n. (see Fig. 15A–F), *Nesticella xixia* sp. n. (see Figs 37A–D, 38E–G) and *Nesticella semicircularis* (see Liu and Li 2013b: 521, figs 19–22). It can be easily separated from the first species by the presence of fully developed eyes (Fig. 32C vs. Fig. 15A), the wider spermathecae (S) and the thicker ducts (Fd and Cd) (Fig. 32G vs. Fig. 15F). Males can be recognized from those of *Nesticella xixia* sp. n. by the much wider ventral process I (Va-I), the shorter and thicker distal process I (Dp-I), the blunter tegular apophysis (Tg) (Fig. 31A, D vs. Fig. 37A, D); females are distinguished by the straight posterior margin and the longer and slimmer ducts (Fd and Cd) (Fig. 32G vs. Fig. 38G). *Nesticella sanchaheensis* sp. n. can be distinguished from *Nesticella semicircularis* by the wider tegular apophysis (Tg), the wider ventral process I (Va-I), the different shape of the distal process I (Dp-I) when viewed dorsally (Fig. 31A–B, D vs. figs 19B, 20A–B, 21A–B, D) for the males; by the wider scape (Sp) with a straight posterior margin (almost round in *Nesticella semicircularis*) and the rounder spermathecae (S) for the females (Fig. 32G vs. figs 20E, 22E).

##### Description.

Habitus as in Fig. 32A–D. Carapace pale yellow. Cervical groove faint, fovea indistinct. Mouthparts darker than the carapace. Sternum yellowish covered with sparse long setae. Legs and female palps yellowish, distally darker in each tibiae, metatarsi and tarsi. Opisthosoma uniformly grey, darker in females.

Male palp (Fig. 31A–D): paracymbium with two wide ventral processes, Va-I flat, wide and sharp, Va-II short, narrow and blunt (Fig. 31A, D); distal process strongly sclerotized with two branches (Fig. 31B), Dp-I small and stumpy, Dp-II bigger and pointed. Triangular dorsal apophysis laminar and wide (Fig. 31B). Terminal apophysis blunt, horn-like, translucent, and with a granulated surface. Tegular apophysis triangular, weakly sclerotized (Fig. 31A). Conductor ending with a short, spout-shaped, sclerotized process (Fig. 31A, C–D).

Epigyne (Fig. 32E–G): weakly sclerotized. Scape translucent, lobed and protruding beyond the epigynal posterior margin, with a straight posterior margin (Fig. 32E–F). Spermathecae small, globular, separated by about 1.8 diameters (Fig. 32F–G). Fertilization ducts long and thin, reaching the spermathecae with three or four coils (Fig. 32G). Copulatory ducts short, constricted at the base and slightly bent in the middle (Fig. 32F–G).

Male (holotype). Total length 2.78. Carapace 1.31 long, 1.18 wide. Opisthosoma 1.56 long, 1.15 wide. Clypeus height 0.23. Sternum 0.80 long, 0.78 wide. Leg measurements: see Appendix A.

Female (one of the paratypes). Total length 2.81. Carapace 1.31 long, 1.16 wide. Opisthosoma 1.66 long, 1.38 wide. Clypeus height 0.22. Sternum 0.81 long, 0.75 wide. Leg measurements: see Appendix A.

##### Habitat.

Cave.

##### Distribution.

Known only from the type locality (Fig. 82).

#### Nesticella songi

Taxon classificationAnimaliaAraneaeNesticidae

Chen & Zhu, 2004

Figs 33 , 34 , 82

Nesticella songi Chen & Zhu, 2004: 87, figs 1–7 (♂♀).

##### Material examined.

Holotype ♂ and paratypes 1♂2♀ (MHBU), CHINA: Guizhou Province, Libo County, Maolan National Nature Reserve, Shuipa Village, Shuipashui Cave (25.40000°N, 107.80000°E), 7.VII.2001, H. Chen leg.

##### Diagnosis.

**Figure 33.**
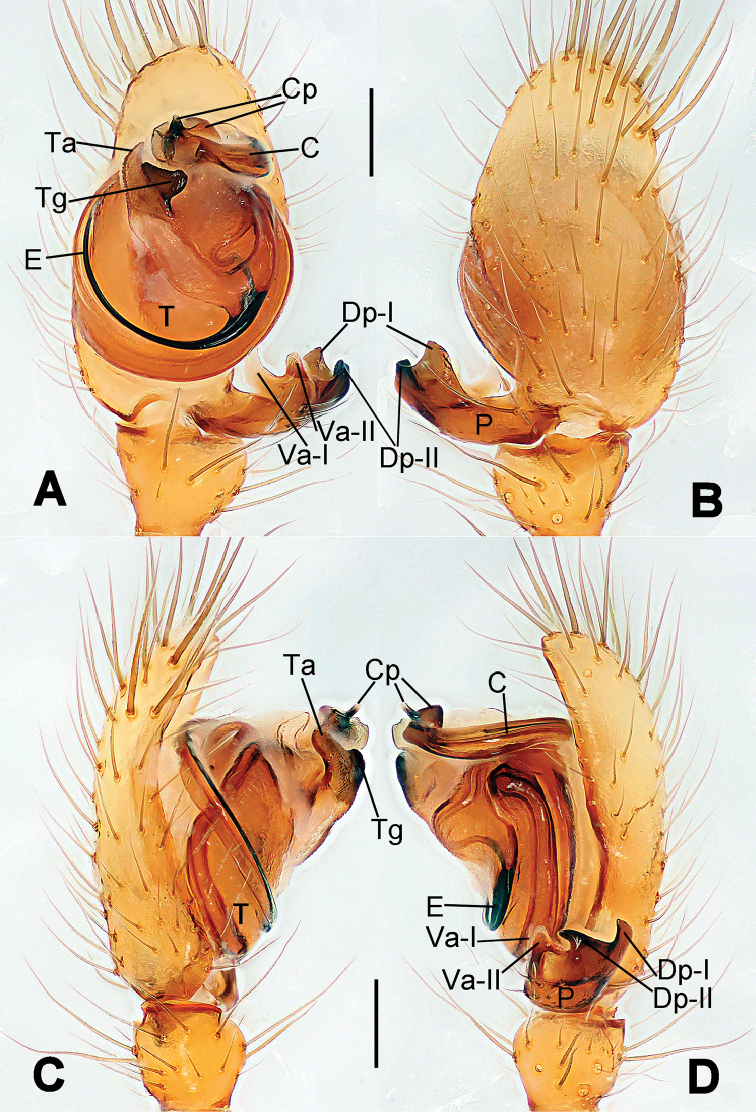
*Nesticella songi*, paratype (male). **A** Palp, ventral view **B** Ditto, dorsal view **C** Ditto, prolateral view **D** Ditto, retrolateral view. Scale bars: 0.10 mm.

**Figure 34.**
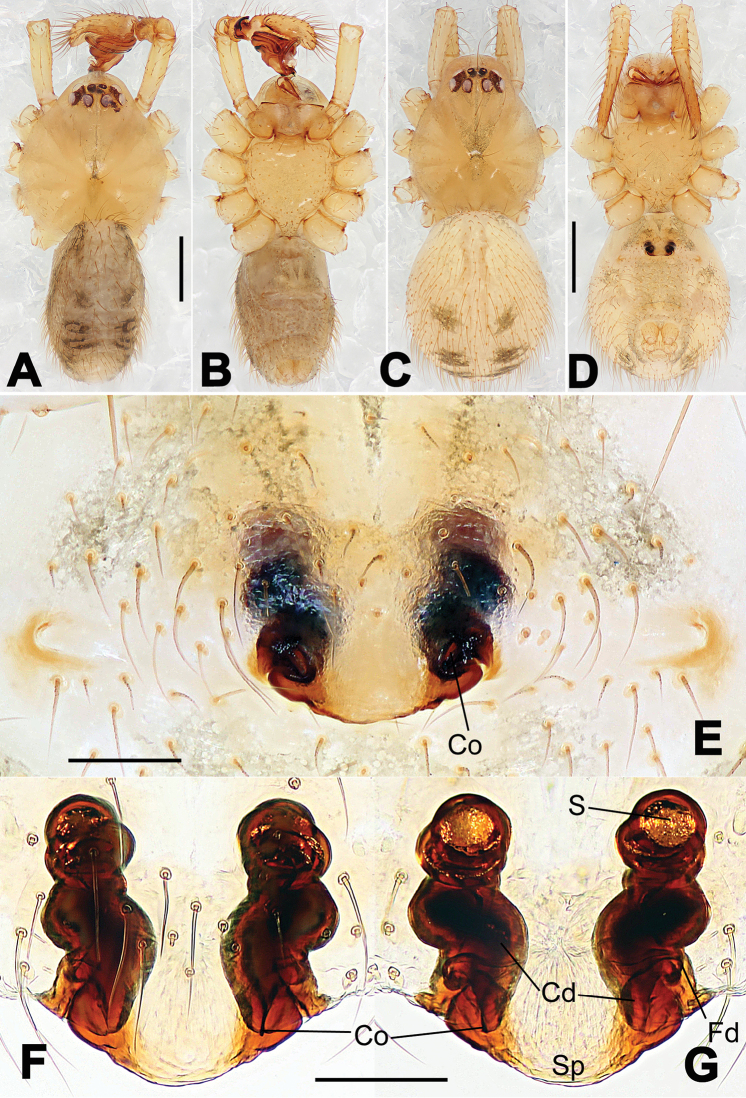
*Nesticella songi*, paratypes (male and female). **A** Male habitus, dorsal view **B** Ditto, ventral view **C** Female habitus, dorsal view **D** Ditto, ventral view **E** Epigyne, ventral view **F** Vulva, ventral view **G** Vulva, dorsal view. Scale bars: **A–D** = 0.50 mm; **E–G** = 0.10 mm.

*Nesticella songi* is closely related to *Nesticella nandanensis* sp. n., *Nesticella baiseensis* sp. n. and *Nesticella yao* sp. n. Males of the new species can be separated from those of *Nesticella nandanensis* sp. n. by the blunter tip of the tegular apophysis (Tg) (Fig. 33A vs. Fig. 22A), by the narrower distal process I of the paracymbium (Dp-I) in dorsal view (Fig. 33B vs. Fig. 22B), by the shorter ventral process I (Va-I) and the blunter ventral process II (Va-II) (Fig. 33A, D vs. Fig. 22A, D). They can be separated from males of *Nesticella baiseensis* sp. n. by the shorter and thicker tegular apophysis (Tg), by the more squared distal process I (Dp-I), by the longer ventral process I (Va-I) and the thicker ventral process II (Va-II) (Fig. 33A–B, D vs. Fig. 8A–B, D). Females of *Nesticella songi* can be recognized from those of *Nesticella nandanensis* sp. n. by the narrower space between the spermathecae (S) and the more parallel ducts (Fd and Cd) rather than ducts that are oriented outward (Fig. 34F–G vs. Fig. 23F–G). They are distinguished from females of *Nesticella baiseensis* sp. n. by the wider scape (Sp) and the narrower and more twisted ducts (Fd and Cd) (Fig. 34G vs. Fig. 9G). Females of *Nesticella songi* are distinguished from those of *Nesticella yao* sp. n. by the almost round scape (Sp) with a convex and smooth posterior margin rather than an almost flat and wrinkled scape, by less straight ducts (Fd and Cd) (Fig. 34E–G vs. Fig. 39D–F).

##### Description.

See Figs 33A–D, 34A–G and Chen and Zhu (2004).

##### Habitat.

Cave.

##### Distribution.

China (Guizhou) (Fig. 82).

#### Nesticella xiongmao sp. n.

Taxon classificationAnimaliaAraneaeNesticidae

http://zoobank.org/CA09CB30-527F-4CAF-868E-3E737EAC3ECB

Figs 35 , 36 , 82

##### Type material.

Holotype ♂ and paratypes 8♂5♀ (IZCAS), CHINA: Sichuan Province, Baoxing County, Longdong Town (30.47382°N, 102.70562°E, 1180 m), 28.VI.2004, S. Li leg.

##### Etymology.

The specific name derives from the Chinese pinyin “xióng māo” = panda, and refers to the type locality of the new spider species, located in the type locality of the giant panda; noun in apposition.

##### Diagnosis.

**Figure 35.**
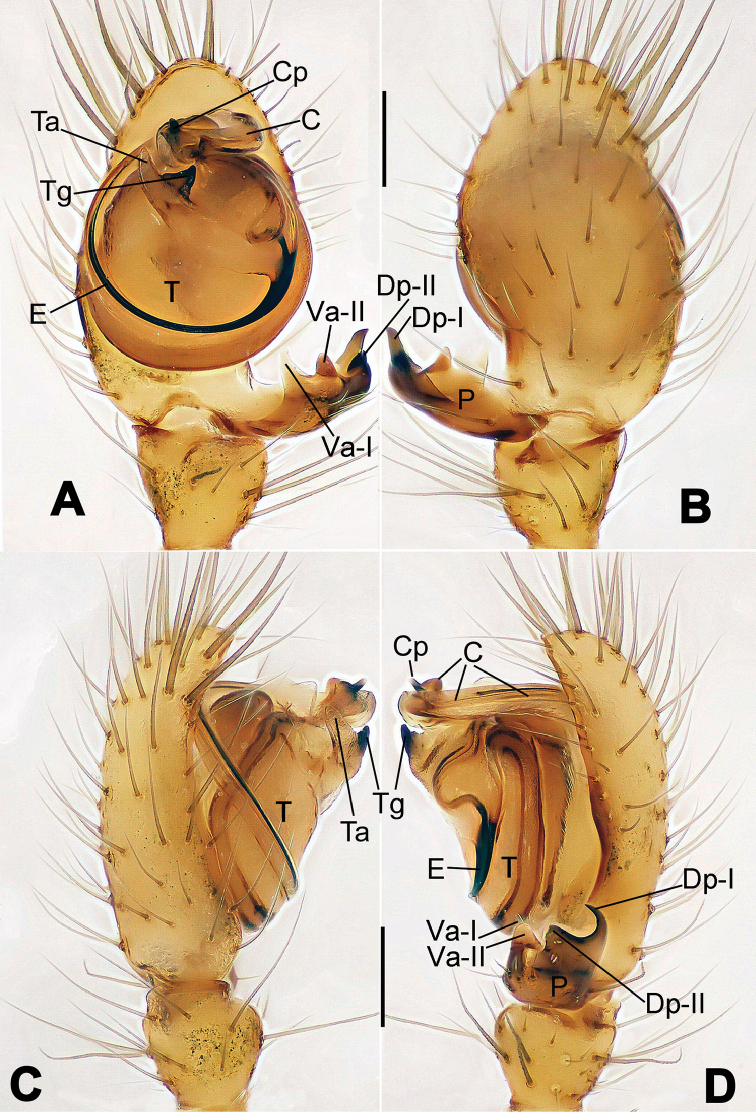
*Nesticella xiongmao* sp. n., holotype (male). **A** Palp, ventral view **B** Ditto, dorsal view **C** Ditto, prolateral view **D** Ditto, retrolateral view. Scale bars: 0.10 mm.

**Figure 36.**
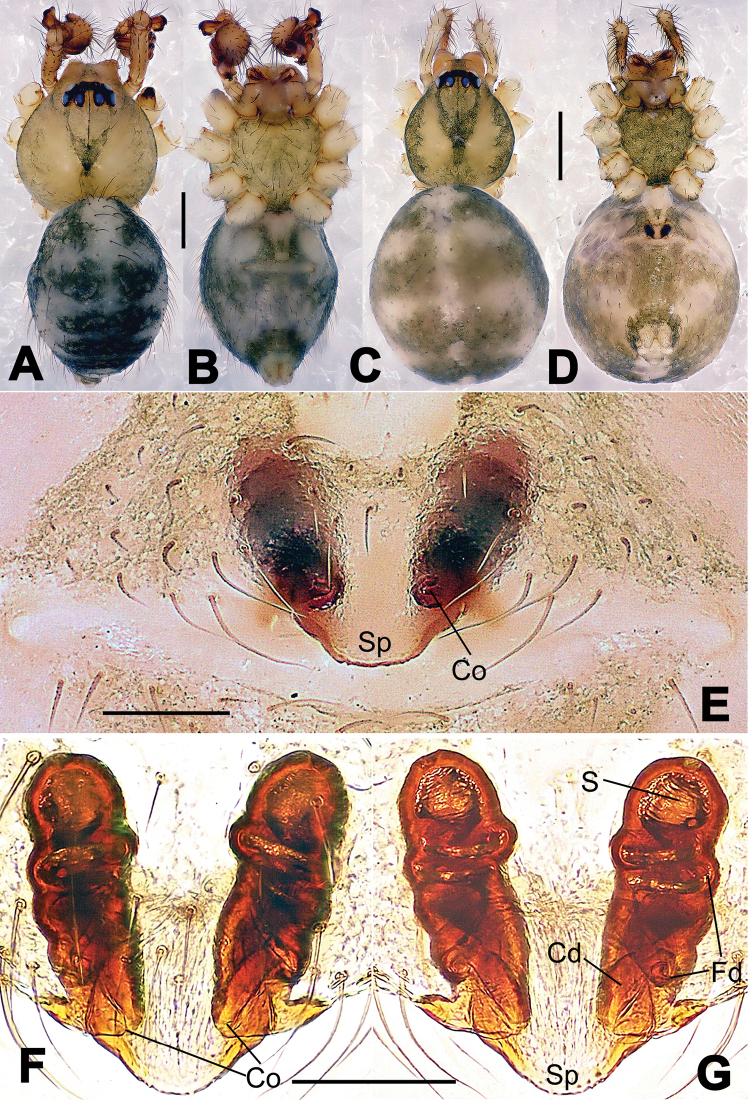
*Nesticella xiongmao* sp. n., holotype (male) and paratype (female). **A** Male habitus, dorsal view **B** Ditto, ventral view **C** Female habitus, dorsal view **D** Ditto, ventral view **E** Epigyne, ventral view **F** Vulva, ventral view **G** Vulva, dorsal view. Scale bars: **A–D** = 0.50 mm; **E–G** = 0.10 mm.

The new species can be easily distinguished from all the species belonging to the *brevipes*-group, with the exception of *Nesticella odonta* and *Nesticella chongqing* sp. n., by the sharp, hook-like distal process I of the paracymbium (Dp-I) that is bent downward (Fig. 35D), the lobed distal process II (Dp-II) (Fig. 35D) and the triangular tegular apophysis (Tg) with a sharp tip (Fig. 35A). Females can be distinguished by the tongue-like, protruding scape (Sp) with an almost round posterior margin (Fig. 36F) and by the compact, straight ducts that are almost parallel to each other (Fig. 36G). *Nesticella xiongmao* sp. n. can be recognized from *Nesticella odonta* by the thinner distal process I of the paracymbium (Dp-I) (Fig. 35A, D vs. Fig. 24A, D) and the shorter and less lobed distal process II (Dp-II) (Fig. 35D vs. Fig. 24D); females are distinguished by the longer, tongue-like scape (Sp) with an almost round posterior margin rather than a flat scape (Fig. 36E–F vs. Fig. 25E–F), by the more compact and straighter ducts (Fd and Cd) and by the shorter distance between the spermathecae (S) (Fig. 36F–G vs. Fig. 25F–G). Females of *Nesticella xiongmao* sp. n. can be separated from those of *Nesticella chongqing* sp. n. by the more protruding and narrower scape and by the narrower space between the copulatory ducts (Cd) (Fig. 36F–G vs. Fig. 12E–F).

##### Description.

Habitus as in Fig. 36A–D. Carapace pale yellow, dark at margins and near the cephalic area; darker in females. Ocular area dark. Cervical groove and fovea distinct. Mouthparts brown-yellowish. Sternum pigmented, darker in female. Legs and female palps yellowish, distally darker and with a dark ring at each tibia and metatarsus. Opisthosoma covered with long setae, greyish, with paired black marks on the dorsal, lateral and ventral sides, partially fused to each other. The whole pattern is more evident in males.

Male palp (Fig. 35A–D): Va-I long and laminar, Va-II short and stockier (Fig. 35A). Two sclerotized distal processes, Dp-I hook-like, long and sharp, Dp-II short and lobed, bent downward (Fig. 35D). Terminal apophysis blunt, finger-like, translucent and with a textured surface (Fig. 35C). Tegular apophysis strongly sclerotized, triangular, with a sharp tip (Fig. 35A, C–D). Conductor with a small, tooth-like process (Fig. 35A, C–D).

Epigyne (Fig. 36E–G): greyish. Scape lobed and protruding out of the epigynal posterior margin, tongue-like, about two times longer than wide, and showing an almost round posterior margin (Fig. 36F). Spermathecae small, ovate, separated each other by about 1.3 diameters (Fig. 36F–G). Ducts compact and straight, almost parallel to each other. Fertilization ducts thick and long, reaching the spermathecae with 2.5 loops (Fig. 36G). Copulatory ducts thick and short (Fig. 36F).

Male (holotype). Total length 2.45. Carapace 1.18 long, 1.09 wide. Opisthosoma 1.45 long, 1.06 wide. Clypeus height 0.23. Sternum 0.75 long, 0.70 wide. Leg measurements: see Appendix A.

Female (one of the paratypes). Total length 2.91. Carapace 1.17 long, 0.99 wide. Opisthosoma 1.84 long, 1.64 wide. Clypeus height 0.23. Sternum 0.71 long, 0.66 wide. Leg measurements: see Appendix A.

##### Habitat.

Forest leaf litter.

##### Distribution.

Known only from the type locality (Fig. 82).

#### Nesticella xixia sp. n.

Taxon classificationAnimaliaAraneaeNesticidae

http://zoobank.org/D41AEE29-A9C0-4BE6-9652-6D7D546202DE

Figs 37 , 38 , 82

##### Type material.

Holotype ♂ and paratypes 3♂5♀ (IZCAS), CHINA: Henan Province, Xixia County, Baihe Village, Yunhuabianfu Cave (33.30330°N, 111.42917°E, 373 m), 29.V.2014, Y. Li & J.C. Liu leg.

##### Etymology.

The specific name is derived from the type locality; adjective.

##### Diagnosis.

**Figure 37.**
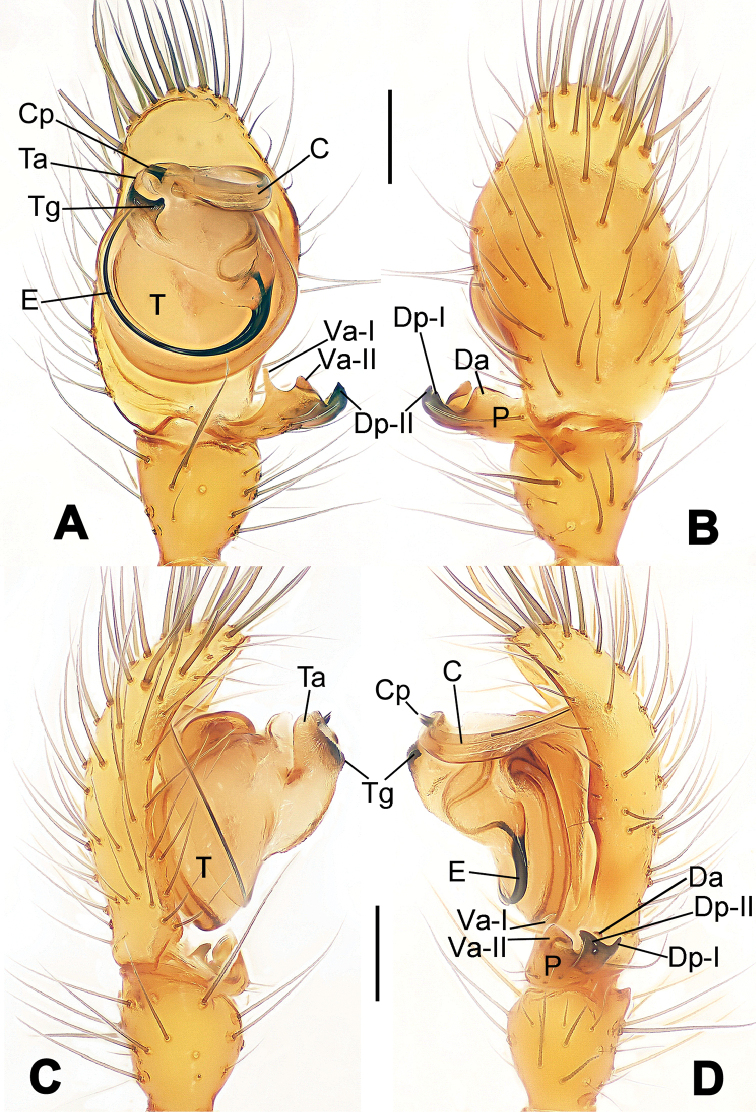
*Nesticella xixia* sp. n., holotype (male). **A** Palp, ventral view **B** Ditto, dorsal view **C** Ditto, prolateral view **D** Ditto, retrolateral view. Scale bars: 0.10 mm.

**Figure 38.**
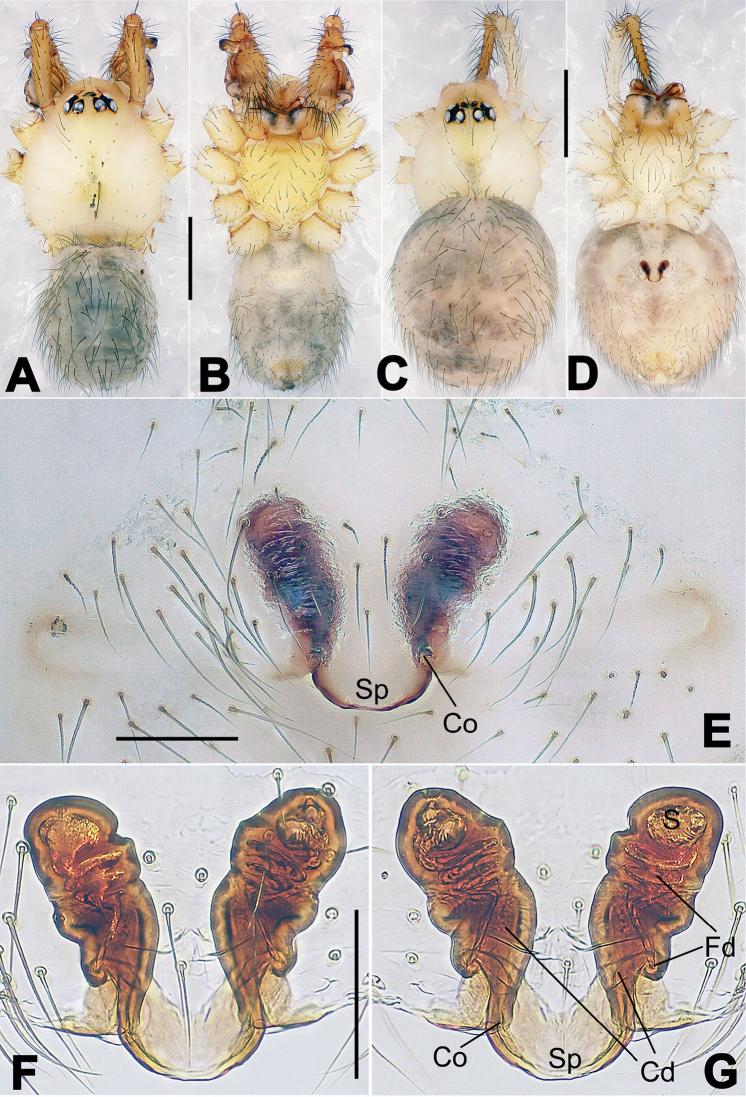
*Nesticella xixia* sp. n., holotype (male) and paratype (female). **A** Male habitus, dorsal view **B** Ditto, ventral view **C** Female habitus, dorsal view **D** Ditto, ventral view **E** Epigyne, ventral view **F** Vulva, ventral view **G** Vulva, dorsal view. Scale bars: **A–D** = 0.50 mm; **E–G** = 0.10 mm.

This new species is closely related to *Nesticella sanchaheensis* sp. n. and *Nesticella semicircularis*. It can be separated from the former species by the sharper tip of the tegular apophysis (Tg), the much thinner and sharp ventral process I (Va-I) and the smaller and narrower distal process I of the paracymbium (Dp-I) when observed in retrolateral view (Fig. 37A, D vs. Fig. 31A, D); females are separated by the shorter ducts (Fd and Cd), the smaller spermathecae (S) and the almost round posterior margin of the scape (Sp) (Fig. 38G vs. Fig. 32G) which is flat in *Nesticella sanchaheensis* sp. n.. *Nesticella xixia* sp. n. can be distinguished from *Nesticella semicircularis* (see Liu and Li 2013b: 521, figs 19–22) by the slimmer ventral process I of the paracymbium (Va-I), the sharper tegular apophysis (Tg) and the different shape of the distal processes (Dp-I, Dp-II) (Fig. 37A, D vs. figs 19A, 20A–B, 21A–B, D) for the males; females instead are recognized by the wider scape (Sp), the smaller spermathecae (S) and the larger distance between the copulatory ducts (Cd) (Fig. 38E–G vs. figs 20C–E, 22C–E). The general shape of the ventral and distal processes of the paracymbium (Dp-I and Dp-II), the short copulatory ducts (Cd) and the lobed scape (Sp) allow easy separation from all the other species of the *brevipes*-group.

##### Description.

Habitus as in Fig. 38A–D. Carapace pale yellow. Cervical groove faint, fovea distinct. Mouthparts slightly darker than the carapace. Legs uniformly pale yellow. Female palpal femur and patella yellowish, tibia and tarsus darker yellow. Opisthosoma uniformly greyish with faint dark marks. Dorsum darker than venter.

Male palp (Fig. 37A–D): paracymbium with Va-I long, very thin and sharp, Va-II shorter and with a blunt tip (Fig. 37A, D); distal process strongly sclerotized, bifurcated (Fig. 37B, D). Dorsal apophysis wide and blunt (Fig. 37B). Terminal apophysis blunt, wrinkled and with a thick protuberance (Fig. 37C). Tegular apophysis short, strongly sclerotized with a sharp tip (Fig. 37A). Conductor with a short, spout-shaped, sclerotized process at the apex (Fig. 37A, D).

Epigyne (Fig. 38E–G): wrinkled and translucent (Fig. 38E). Scape short, translucent, lobed with an almost round posterior margin, about two times wider than long (Fig. 38E). Spermathecae approximately round, separated by about their diameter (Fig. 38F). Fertilization ducts thin and long, reaching the spermathecae with more than three coils (Fig. 38F–G). Copulatory ducts thick and short, basally constricted (Fig. 38G).

Male (holotype). Total length 2.84. Carapace 1.48 long, 1.33 wide. Opisthosoma 1.50 long, 1.06 wide. Clypeus height 0.26. Sternum 0.88 long, 0.82 wide. Leg measurements: see Appendix A.

Female (one of the paratypes). Total length 2.97. Carapace 1.42 long, 1.25 wide. Opisthosoma 1.88 long, 1.56 wide. Clypeus height 0.27. Sternum 0.84 long, 0.80 wide. Leg measurements: see Appendix A.

##### Habitat.

Cave.

##### Distribution.

Known only from the type locality (Fig. 82).

#### Nesticella yao sp. n.

Taxon classificationAnimaliaAraneaeNesticidae

http://zoobank.org/0906AC6C-D457-4EE1-9697-4CD8BEF0D699

Figs 39 , 82

##### Type material.

Holotype ♀ and paratype 1♀ (IZCAS), CHINA: Guangxi Zhuang Autonomous Region, Gongcheng County, Songlin Village, Houyan Cave (24.96742°N, 110.88112°E, 221 m), 28.VIII.2009, Z. Yao leg.

##### Etymology.

The name derives from the Yao people, an ethnic minority living in the type locality; noun in apposition.

##### Diagnosis.

**Figure 39.**
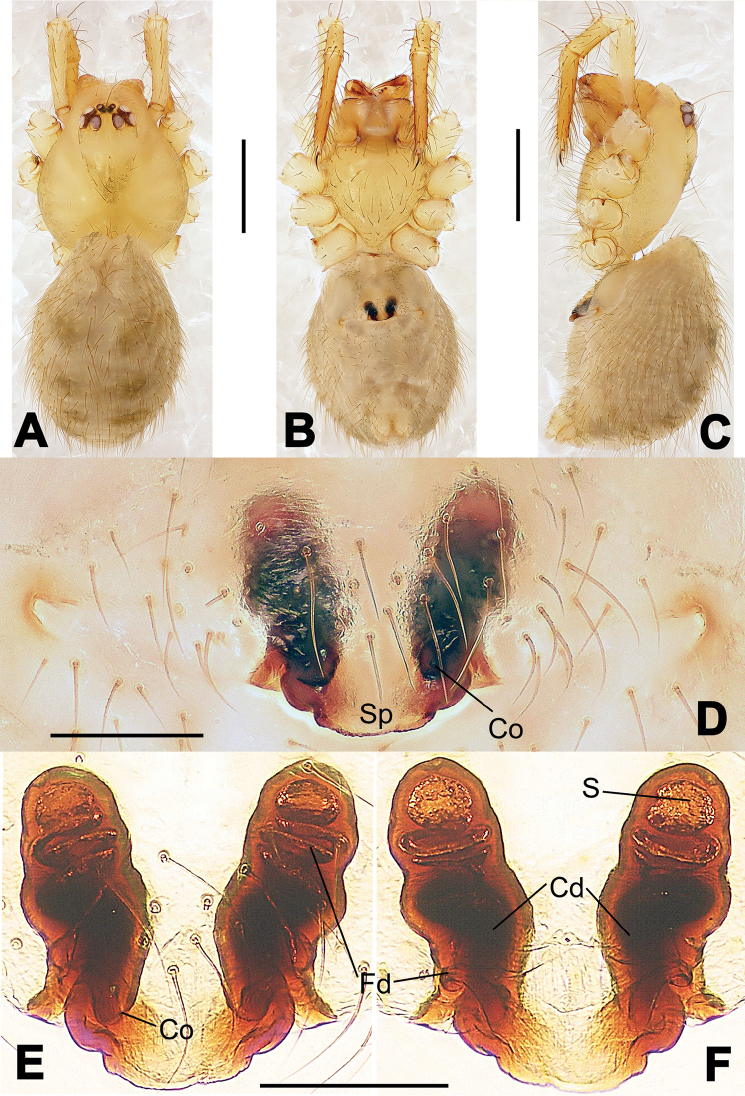
*Nesticella yao* sp. n., holotype (female). **A** Habitus, dorsal view **B** Ditto, ventral view **C** Ditto, lateral view **D** Epigyne, ventral view **E** Vulva, ventral view **F** Vulva, dorsal view. Scale bars: **A–C** = 0.50 mm; **D–F** = 0.10 mm.

The new species is closely related to *Nesticella songi* Chen & Zhu, 2004 (see Fig. 34E–G, and Chen and Zhu 2004: 87, figs 6–7) and *Nesticella nandanensis* sp. n. (see Fig. 23E–G). It can be separated from the former species by the squarer scape (Sp) with a flat and furrowed posterior margin, by the straighter and more compact ducts (Fd and Cd) and by the lack of a clear constriction around the coils of the fertilization ducts (Fd) (Fig. 39D–F vs. Fig. 34E–G). It can be recognized from *Nesticella nandanensis* sp. n. by the squarer scape (Sp) with a flat rather than convex posterior margin, by the straighter copulatory ducts (Cd) (bent outward in *Nesticella nandanensis* sp. n.) and the spermathecae (S) closer to each other (Fig. 39D–F vs. Fig. 23E–G).

##### Description.

Habitus as in Fig. 39A–C. Carapace yellowish, with several setae along the cervical groove. Cervical groove and fovea indistinct. Mouthparts yellow. Sternum yellow. Female palps and legs yellowish, distally darker in metatarsi and tarsi. Opisthosoma uniformly yellowish with three paired, greyish dorsal marks.

Epigyne (Fig. 39D–F): slightly wrinkled and translucent (Fig. 39D). Scape short and wide, laminar, and laterally sclerotized, with a flat and furrowed posterior margin (Fig. 39D). Spermathecae small and globular, separated by about 1.4 diameters (Fig. 39F). Fertilization ducts thin, reaching the spermathecae with three coils. Copulatory ducts short, swollen in the central part, about as wide as the spermathecae (Fig. 39E–F).

Female (holotype). Total length 2.84. Carapace 1.36 long, 1.14 wide. Opisthosoma 1.72 long, 1.13 wide. Clypeus height 0.22. Sternum 0.80 long, 0.78 wide. Leg measurements: see Appendix A.

Male. Unknown.

##### Habitat.

Cave.

##### Distribution.

Known only from the type locality (Fig. 82).

### *Nesticella mogera*-group

**Group features.** Males belonging to this species-group are characterized by the following combination of characters: paracymbium having a single ventral apophysis (Va) with a sharp tip bent inward and a squared, stumpy single distal process (Dp); a terminal apophysis (Ta) that is usually flat, basally broad and with a coarse, granulate surface; tegular apophysis absent (Tg); a wide, strongly sclerotized, hook-like process of the conductor (Cp). Females are recognized by the straight and almost parallel copulatory ducts (Cd) and only slightly diverging distally. The massive squared scape (Sp) (wider, narrower or lobed in the other species-groups) that is always protruding far beyond the epigastric furrow further separates this group from the others.

**Composition.** *Nesticella apiculata* Liu & Li, 2013, *Nesticella fuliangensis* sp. n., *Nesticella helenensis* (Hubert, 1977), *Nesticella huomachongensis* sp. n., *Nesticella mogera* (Yaginuma, 1972), *Nesticella rongtangensis* sp. n., *Nesticella wanzaiensis* sp. n. and *Nesticella yanbeiensis* sp. n.

#### Nesticella fuliangensis sp. n.

Taxon classificationAnimaliaAraneaeNesticidae

http://zoobank.org/AA75D82B-0D18-449C-B78A-27742A944D07

Figs 40 , 41 , 83

##### Type material.

Holotype ♂ and paratypes 2♂6♀ (IZCAS), CHINA: Jiangxi Province, Fuliang County, Shouan Town, Zhuxian Cave (29.21353°N, 117.30285°E, 191 m), 22.V.2013, Y. Luo & J. Liu leg.

##### Etymology.

The specific name is derived from the type locality; adjective.

##### Diagnosis.

**Figure 40.**
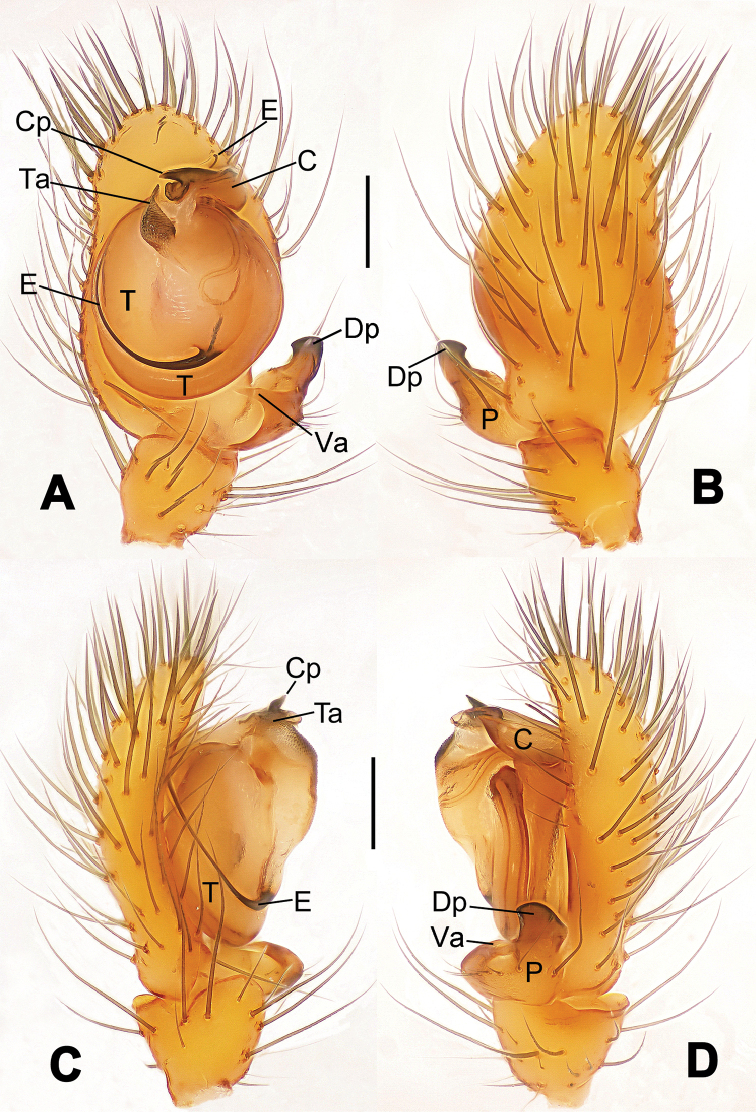
*Nesticella fuliangensis* sp. n., holotype (male). **A** Palp, ventral view **B** Ditto, dorsal view **C** Ditto, prolateral view **D** Ditto, retrolateral view. Scale bars: 0.10 mm.

**Figure 41.**
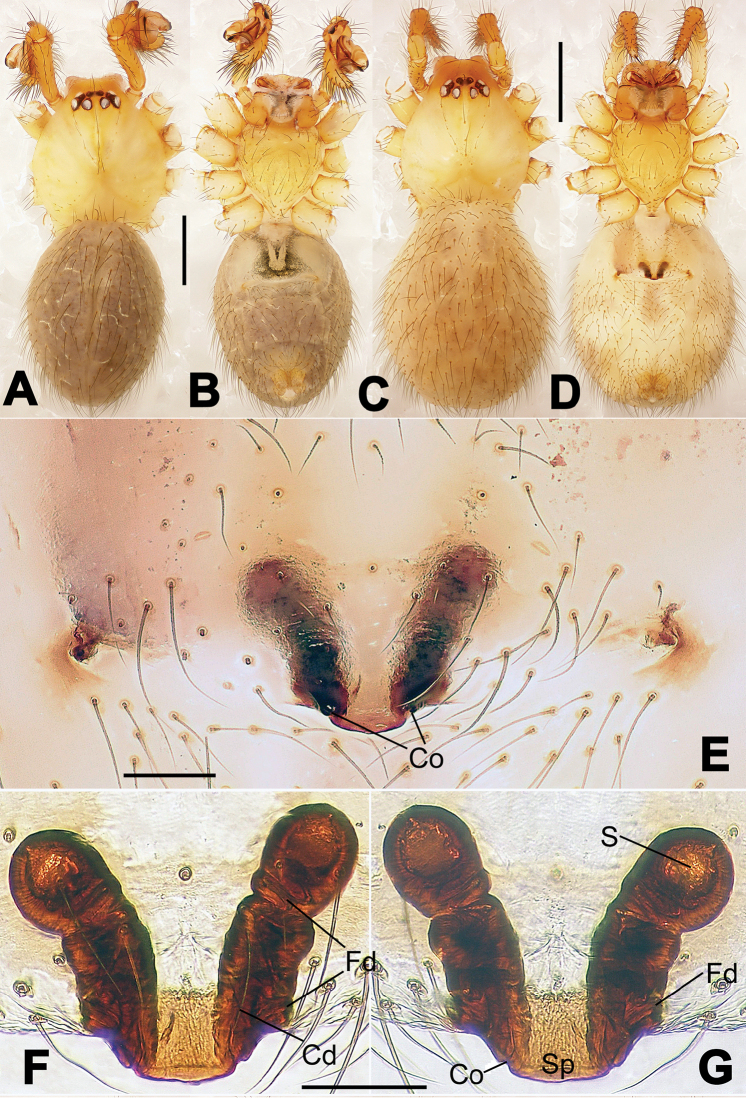
*Nesticella fuliangensis* sp. n., holotype (male) and paratype (female). **A** Male habitus, dorsal view **B** Ditto, ventral view **C** Female habitus, dorsal view **D** Ditto, ventral view **E** Epigyne, ventral view **F** Vulva, ventral view **G** Vulva, dorsal view. Scale bars: **A–D** = 0.50 mm; **E–G** = 0.10 mm.

**Figure 42.**
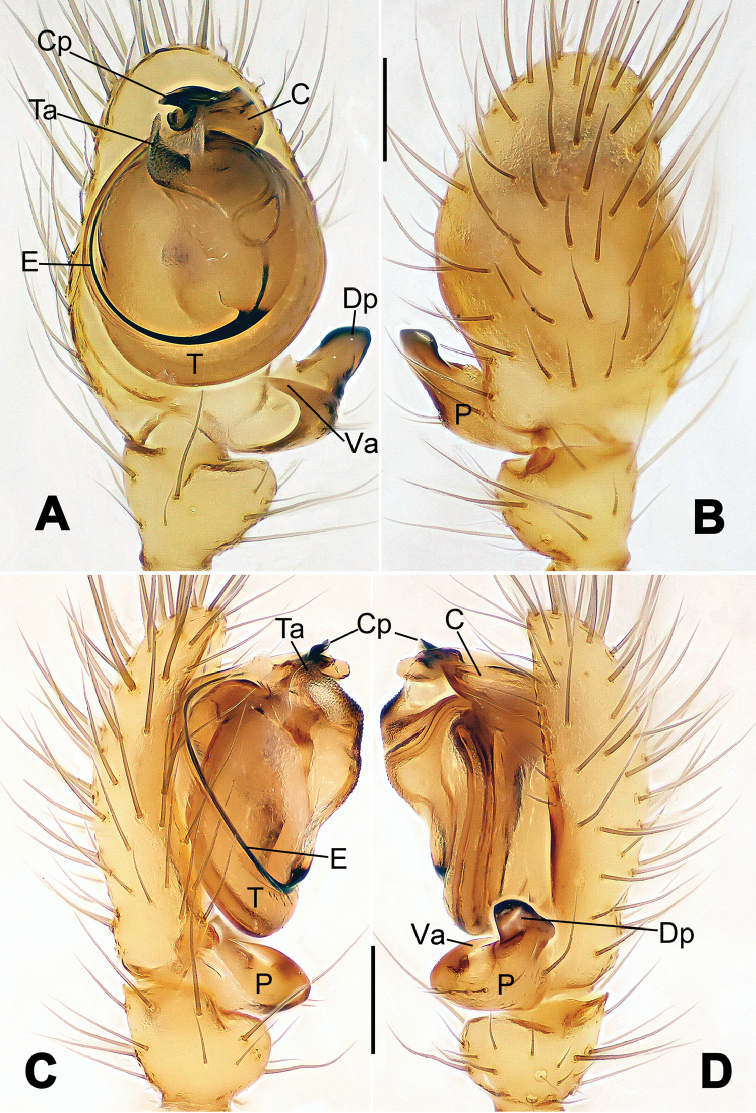
*Nesticella huomachongensis* sp. n., holotype (male). **A** Palp, ventral view **B** Ditto, dorsal view **C** Ditto, prolateral view **D** Ditto, retrolateral view. Scale bars: 0.10 mm.

**Figure 43.**
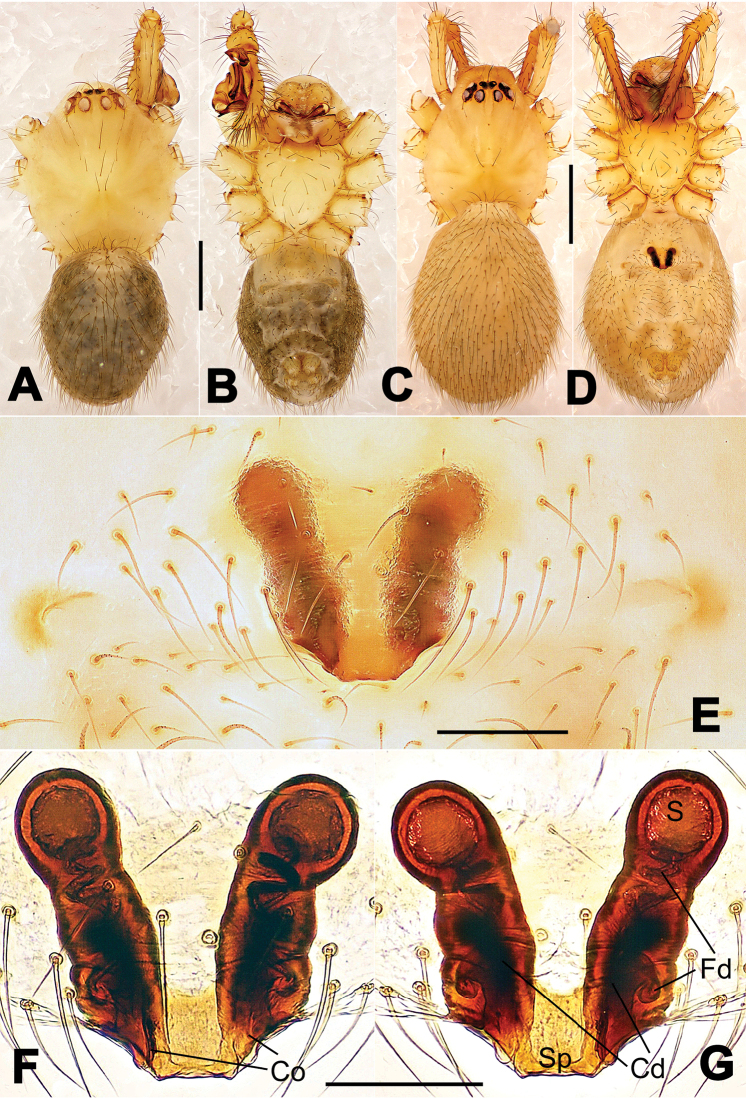
*Nesticella huomachongensis* sp. n., holotype (male) and paratype (female). **A** Male habitus, dorsal view **B** Ditto, ventral view **C** Female habitus, dorsal view **D** Ditto, ventral view **E** Epigyne, ventral view **F** Vulva, ventral view **G** Vulva, dorsal view. Scale bars: **A–D** = 0.50 mm; **E–G** = 0.10 mm.

This new species is closely related to *Nesticella huomachongensis* sp. n. (Figs 42A–D, 43A–G) and *Nesticella yanbeiensis* sp. n. (Figs 47A–D, 48A–G). Males can be distinguished from those of the other two species by the smaller and slimmer process of the conductor (Cp) and by the narrower distal process of the paracymbium (Dp) (Fig. 40A, D vs. Fig. 42A, D vs. Fig. 47A, D). Females can be separated from those of *Nesticella huomachongensis* sp. n. by the different shape of the posterior margin of scape (convex in *Nesticella fuliangensis* sp. n., straight in *Nesticella huomachongensis* sp. n.) (Fig. 41E–G vs. Fig. 43E–G); from those of *Nesticella yanbeiensis* sp. n. by the shorter and wider scape (Sp) and by the greater distance between the copulatory ducts (Cd) (Fig. 41E–G vs. Figs 48E–G). The same combination of characters allows an easy separation from all the other species of the group.

##### Description.

Habitus as in Fig. 41A–D. Carapace yellowish. Cervical groove and fovea indistinct. Mouthparts yellow in males, brown-yellowish in females. Sternum yellow, brighter in females. Legs yellowish, metatarsi and tarsi distally darker. Female palpal femur pale yellow, tibia and tarsus brownish. Opisthosoma uniformly faint grey, pigmented epigastric area in males, grey-yellowish in females.

Male palp (Fig. 40A–D): paracymbium with a row of short setae (Fig. 40D), ventral apophysis sharp and triangular, distal process stumpy, strongly sclerotized (Fig. 40A–B, D). Terminal apophysis flat, well-developed and distinctly sclerotized, basally broad and granulate (Fig. 40A, C). Conductor, ending with a sharp, horn-like, sclerotized apophysis (Fig. 40A, C–D).

Epigyne (Fig. 41E–G): posterior margin of the scape weakly sclerotized and straight (Fig. 41E). Scape short and squared, wider than the spermathecae, with a convex posterior margin (Fig. 41F). Copulatory ducts straight and swollen (Fig. 41G), fertilization ducts twisted into two or three loops before reaching the spermathecae (Fig. 41G). Spermathecae approximately globular, as wide as the copulatory ducts, separated by about 1.5 diameters (Fig. 41F–G).

Male (holotype). Total length 3.16. Carapace 1.47 long, 1.28 wide. Opisthosoma 1.91 long, 1.34 wide. Clypeus height 0.23. Sternum 0.91 long, 0.80 wide. Leg measurements: see Appendix A.

Female (one of the paratypes). Total length 3.56. Carapace 1.56 long, 1.31 wide. Opisthosoma 2.28 long, 1.69 wide. Clypeus height 0.22. Sternum 0.97 long, 0.81 wide. Leg measurements: See Appendix A.

##### Habitat.

Cave.

##### Distribution.

Known only from the type locality (Fig. 83).

#### Nesticella huomachongensis sp. n.

Taxon classificationAnimaliaAraneaeNesticidae

http://zoobank.org/63D10571-1698-489F-8E2F-94D8422213D4

Figs 42 , 43 , 83

##### Type material.

Holotype ♂ and paratypes 2♀ (IZCAS), CHINA: Hunan Province, Chenxi County, Huomachong Town, Yanzi Cave (27.85746°N, 110.26079°E, 439 m), 3.V.2011, Z. Zha leg.

##### Etymology.

The specific name is derived from the type locality; adjective.

##### Diagnosis.

*Nesticella huomachongensis* sp. n. is closely related to *Nesticella wanzaiensis* sp. n. (see Figs 45A–D, 46A–G) and *Nesticella fuliangensis* sp. n. (see Figs 40A–D, 41A–G). Males can be separated from those of *Nesticella wanzaiensis* sp. n. by the shorter and more ovate cymbium (Fig. 42B vs. Fig. 45B), the shorter process of the conductor (Cp) (Fig. 42D vs. Fig. 45D) and the different shape of the distal process of the paracymbium (Dp) which appears stockier in *Nesticella wanzaiensis* sp. n. (Fig. 42A vs. Fig. 45A). The wider process of the conductor (Cp) and the more squared distal process of the paracymbium (Dp) (Fig. 42A, D vs. Fig. 40A, D) allow the separation of males of the new species from those of *Nesticella fuliangensis* sp. n. Females can be recognized by the wider and squarer scape (Sp) (narrower in *Nesticella wanzaiensis* sp. n.) and by the truncate posterior margin (curved in *Nesticella fuliangensis* sp. n.) (Fig. 43F–G vs. Fig. 46F–G vs. Fig. 41F–G). The same combination of characters allows easy separation of *Nesticella huomachongensis* sp. n. from the other species of the *mogera*-group.

##### Description.

Habitus as in Fig. 43A–D. Carapace yellowish. Ocular area slightly protruding in males. Cervical groove and fovea indistinct. Mouthparts yellowish, darker in females than in males. Sternum pale yellow. Legs uniformly yellowish. Female palpal femur pale yellow, tibia and tarsus brown-yellowish. Opisthosoma grey in males, grey-yellowish in females.

Male palp (Fig. 42A–D): cymbium relatively shorter and rounder then usual, paracymbium strongly sclerotized, with a row of short setae (Fig. 42D), a sharp ventral apophysis and a stout, broad, distinctly sclerotized distal process (Fig. 42A, D). Terminal apophysis broad and distinctly sclerotized, with a granulate surface (Fig. 42A, C). Conductor ending with a strongly sclerotized, horn-like process (Fig. 42A, C–D).

Epigyne (Fig. 43E–G): brown-reddish (Fig. 43D–E). Scape very short and stumpy, posterior margin straight, about as wide as the spermathecae (Fig. 43F). Spermathecae globular, separated by about 1.2 diameters (Fig. 43F–G). Fertilization ducts short, swollen. Copulatory ducts long and coiled (Fig. 43G).

Male (holotype). Total length 2.66. Carapace 1.38 long, 1.21 wide. Opisthosoma 1.44 long, 1.02 wide. Clypeus height 0.18. Sternum 0.71 long, 0.75 wide. Leg measurements: see Appendix A.

Female (one of the paratypes). Total length 3.28. Carapace 1.44 long, 1.22 wide. Opisthosoma 1.88 long, 1.43 wide. Clypeus height 0.24. Sternum 0.81 long, 0.78 wide. Leg measurements: see Appendix A.

##### Habitat.

Cave.

##### Distribution.

Known only from the type locality (Fig. 83).

#### Nesticella mogera

Taxon classificationAnimaliaAraneaeNesticidae

(Yaginuma, 1972)

Nesticus terrestris Yaginuma, 1970: 390, fig. 7 (♂, mismatched).
Nesticus mogera Yaginuma, 1972: 621, fig. 1 (♂♀).
Howaia mogera : Lehtinen and Saaristo 1980: 53, figs 7–9, 22–23, 29b (♂♀) (transfer from Nesticus).
Howaia mogera : Marusik and Guseinov 2003: 38, figs 17–21 (♂♀).
Nesticella mogera : Liu and Li 2013b: 521, figs 17A–D, 18A–D (♂♀). See the World Spider Catalog for the full list of references.

##### Diagnosis.

**Figure 44.**
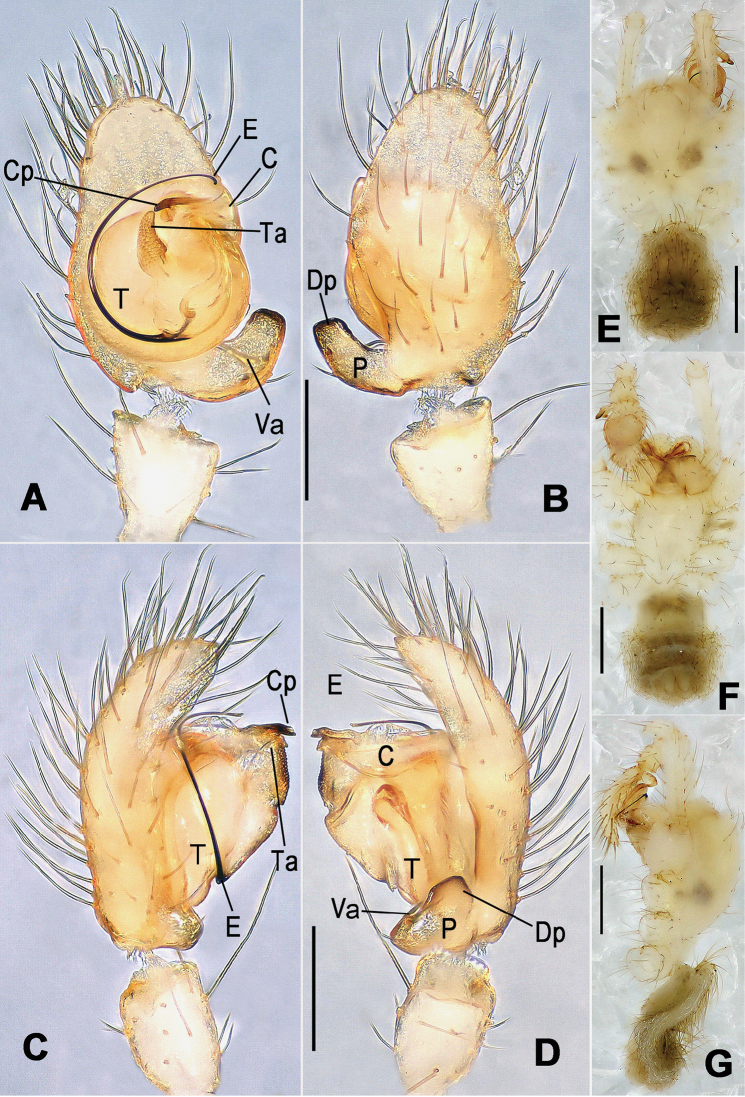
*Nesticella rongtangensis* sp. n., holotype (male). **A** Palp, ventral view **B** Ditto, dorsal view **C** Ditto, prolateral view **D** Ditto, retrolateral view **E** Habitus, dorsal view **F** Ditto, ventral view **G** Ditto, lateral view. Scale bars: 0.10 mm.

Males of *Nesticella mogera* are recognized from those of the other species belonging to the *mogera*-group, with the exception of *Nesticella rongtangensis* sp. n., by the very thin ventral apophysis and the blunter distal process of the paracymbium (see Marusik and Guseinov 2003: 38, figs 17, 18, 20 and Liu and Li 2013b: 521, fig. 17B–C). Females are distinguished by the well-developed, protruding scape, which is shorter and stockier in the other species (see Marusik and Guseinov 2003: 38, fig. 21 and Liu and Li 2013b: 521, fig. 18C–D). *Nesticella mogera* can be easily separated from *Nesticella rongtangensis* sp. n. by the presence of eyes which are missing in the other species (see Liu and Li 2013b: 521, fig. 18A–B vs. Fig. 44E).

##### Description.

See Marusik and Guseinov (2003).

##### Habitat.

A wide range of different habitats, like forest leaf litter, fields, caves, including greenhouses and city parks.

##### Distribution.

Azerbaijan, China, Korea, Japan, Hawaii, Fiji and Europe (introduced).

#### Nesticella rongtangensis sp. n.

Taxon classificationAnimaliaAraneaeNesticidae

http://zoobank.org/F5EF9D2F-9B07-43E2-ADE4-4D7D10E38B9F

Figs 44 , 83

##### Type material.

Holotype ♂ (IZCAS), CHINA: Hainan Province, Haikou City, Xiuying District, Rongtang Village, nearby Volcano Geological Park, Wolong Cave (19.92847°N, 110.21794°E, 143 m), 16.III.2005, collector unknown.

##### Etymology.

The specific name is derived from the type locality; adjective.

##### Diagnosis.

The lack of eyes is a clear, diagnostic character that allows the distinction of *Nesticella rongtangensis* sp. n. from all other species of the *mogera*-group (Fig. 44E, G). Furthermore, the new species can be recognized by the shorter and stockier distal process of the paracymbium (Dp), by the different shape of the process of the conductor (Cp) and the terminal apophysis (Ta) and by the narrower ventral apophysis (Va) (Fig. 44A–D).

##### Description.

Habitus as in Fig. 44E–G. Carapace pale yellow, with sparse short setae. Ocular area with few long setae, eyes completely lacking (Fig. 44E, G). Cervical furrow and fovea indistinct. Mouthparts pale yellow. Sternum pale yellow. Legs uniformly pale yellowish. Opisthosoma grey with long setae.

Male palp (Fig. 44A–D): paracymbium with a sharp, straight, ventral apophysis and a blunt, strongly sclerotized distal process (Fig. 44A–B, D). Terminal apophysis well-developed with granulate surface (Fig. 44A). Conductor ending with a strongly sclerotized, finger-like process (Fig. 44A, C–D).

Male (holotype). Total length 1.60. Carapace 0.92 long, 0.88 wide. Opisthosoma 0.70 long, 0.64 wide. Clypeus height 0.15. Sternum 0.62 long, 0.45 wide. Leg measurements: see Appendix A.

Female. Unknown.

##### Habitat.

Cave.

##### Distribution.

Known only from the type locality (Fig. 83).

#### Nesticella wanzaiensis sp. n.

Taxon classificationAnimaliaAraneaeNesticidae

http://zoobank.org/211FC576-6819-4036-BD39-F4F9EDF48686

Figs 45 , 46 , 83

##### Type material.

Holotype ♂ (IZCAS), CHINA: Jiangxi Province, Wanzai County, Dongkou Village, Zhushan Cave (28.04813°N, 114.36958°E, 125 m), 14.V.2013, Y. Luo & J. Liu leg. Paratypes 5♀ (IZCAS), Jiangxi Province, Wanzai County, Dongkou Village, Shihu Cave (27.03603°N, 114.35468°E), 19.IX.2015, G. Zhou & Z. Chen leg.

##### Etymology.

The specific name is derived from the type locality; adjective.

##### Diagnosis.

**Figure 45.**
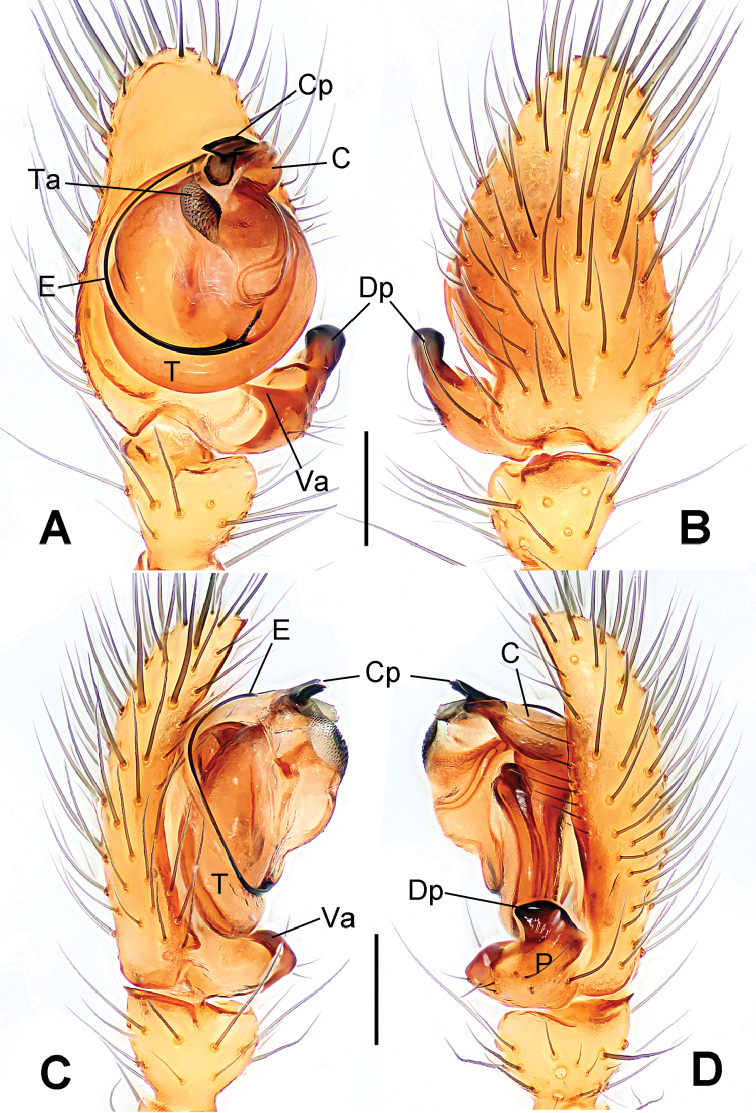
*Nesticella wanzaiensis* sp. n., holotype (male). **A** Palp, ventral view **B** Ditto, dorsal view **C** Ditto, prolateral view **D** Ditto, retrolateral view. Scale bars: 0.10 mm.

**Figure 46.**
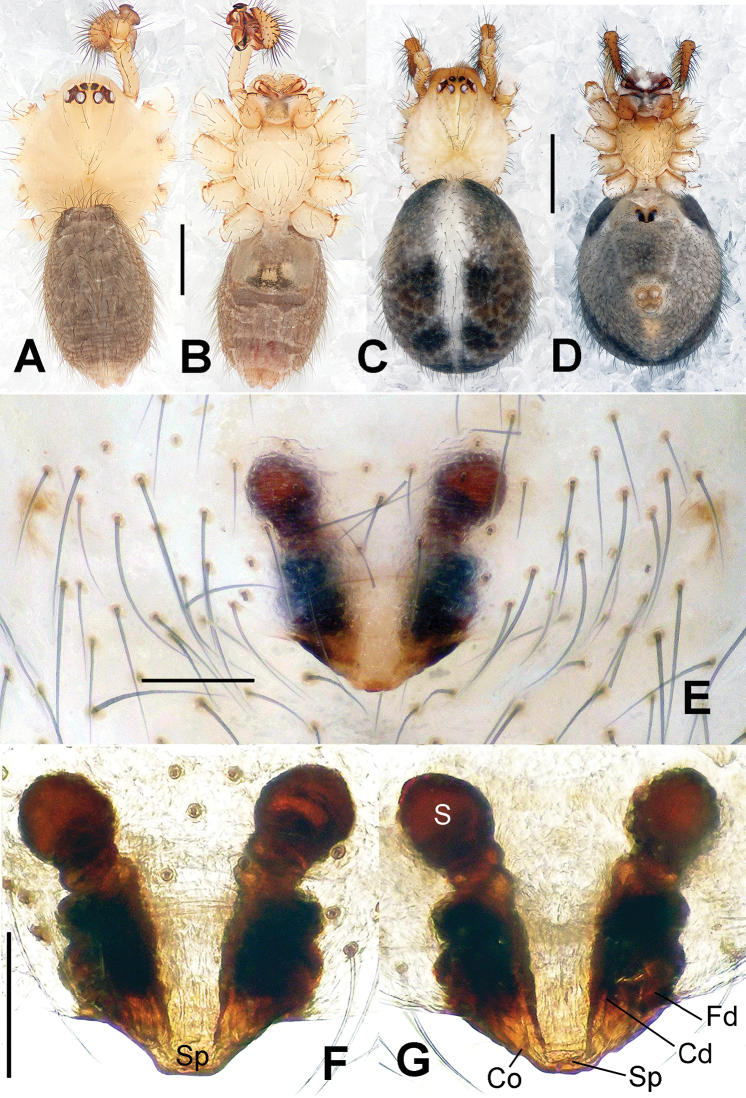
*Nesticella wanzaiensis* sp. n., holotype (male) and paratype (female). **A** Male habitus, dorsal view **B** Ditto, ventral view **C** Female habitus, dorsal view **D** Ditto, ventral view **E** Epigyne, ventral view **F** Vulva, ventral view **G** Vulva, dorsal view. Scale bars: **A–D** = 0.50 mm; **E–G** = 0.10 mm.

This new species is closely related to *Nesticella huomachongensis* sp. n. (see Figs 42A–D, 43A–G) and *Nesticella fuliangensis* sp. n. (see Figs 40A–D, 41 A–G). Males can be separated from those of the former species by the longer cymbium (shorter and rounder in *Nesticella huomachongensis* sp. n.), by the different shape of the process of the conductor (Cp) and by the narrower distal process of the paracymbium (Dp) (Fig. 45A, D vs. Fig. 42A, D). *Nesticella wanzaiensis* sp. n. can be easily distinguished from *Nesticella fuliangensis* sp. n. by the stockier and thicker process of the conductor (Cp) (Fig. 45A, D vs. Fig. 40A, D). Females are distinguished by the narrower and protruding scape, which is shorter and stockier in females of the other two species, and by the narrower part of where the fertilization ducts (Fd) coils reach the spermathecae, much slimmer than the copulatory ducts (Cd) (almost of the same diameter in females of the other species) (Fig. 46E–G vs. Fig. 43E–G vs. Fig. 41E–G).

##### Description.

Habitus as in Fig. 46A–D. Carapace yellowish. Cervical furrow and fovea indistinct. Mouthparts uniformly yellowish, darker in females. Sternum yellowish. Legs uniformly pale yellow. Opisthosoma uniformly grey in males, with a longitudinal lighter strip and two pairs of black dots in females.

Male palp (Fig. 45A–D): paracymbium with several sparse setae, ventral apophysis elongate and sharp, distal process broad, strongly sclerotized with a blunt tip (Fig. 45A–B, D). Terminal apophysis well-developed, nearly square and with a granulate surface (Fig. 45A). Conductor with a triangular stocky process, strongly sclerotized, with a tiny furcation at the tip (Fig. 45C–D).

Epigyne (Fig. 46E–G): posterior margin of the scape weakly sclerotized (Fig. 46E). Scape short and narrow, lightly protruding out of the epigynal posterior margin, about as wide as the diameter of the spermathecae (Fig. 46E). Fertilization ducts short and swollen, reaching the spermathecae with two loops; this area considerably narrower than the copulatory ducts (Fig. 46G). Copulatory ducts short and swollen, strongly sclerotized (Fig. 46G). Spermathecae small and globular, as wide as the copulatory ducts, separated by about two diameters.

Male (holotype). Total length 2.66. Carapace 1.27 long, 1.19 wide. Opisthosoma 1.50 long, 0.94 wide. Clypeus height 0.22. Sternum 0.83 long, 0.71 wide. Leg measurements: see Appendix A.

Female (one of the paratypes). Total length 3.15. Carapace 1.34 long, 1.25 wide. Opisthosoma 1.82 long, 1.63 wide. Clypeus height 0.21. Sternum 0.76 long, 0.73 wide. Leg measurements: see Appendix A.

##### Habitat.

Cave.

##### Distribution.

Jiangxi, China (Fig. 83).

#### Nesticella yanbeiensis sp. n.

Taxon classificationAnimaliaAraneaeNesticidae

http://zoobank.org/B9E36D6C-757B-4C17-A844-05338C0BB89D

Figs 47 , 48 , 83

##### Type material.

Holotype ♂ and paratypes 3♀ (IZCAS), CHINA: Guangxi Zhuang Autonomous Region, Lingchuan County, Yanbei Village, Yanbei Cave (25.51294°N, 110.24344°E, 175 m), 7.XII.2012, Z. Chen & Z. Zhao leg.

##### Etymology.

The specific name is derived from the type locality; adjective.

##### Diagnosis.

**Figure 47.**
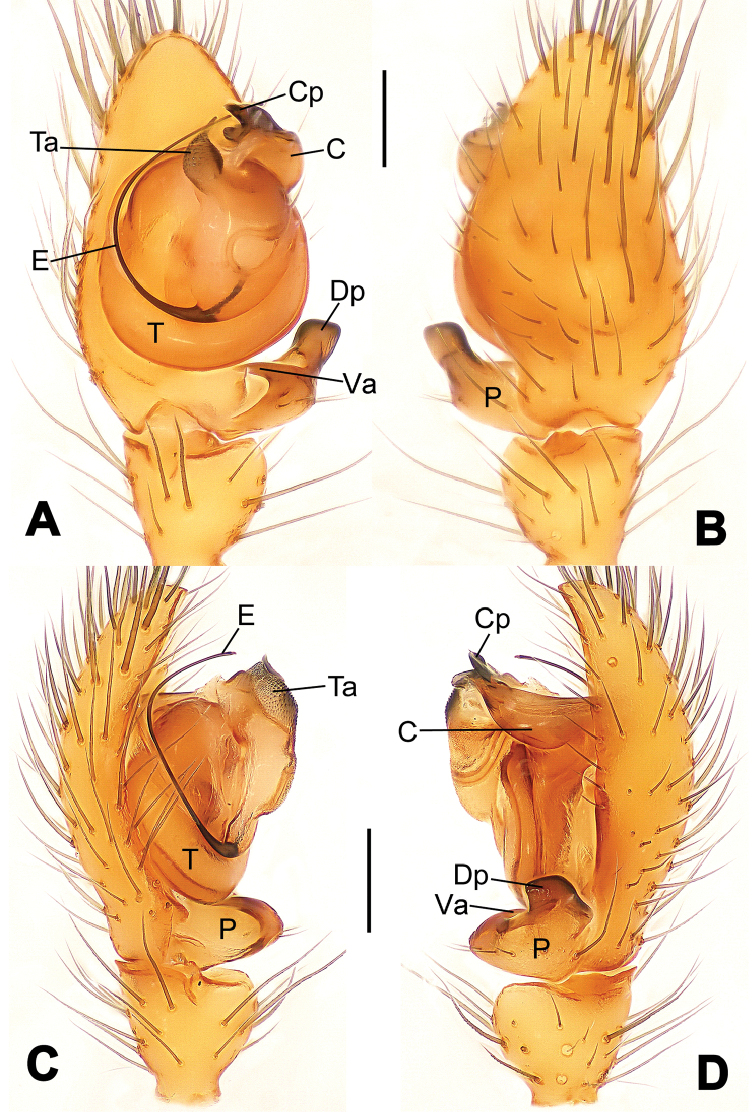
*Nesticella yanbeiensis* sp. n., holotype (male). **A** Palp, ventral view **B** Ditto, dorsal view **C** Ditto, prolateral view **D** Ditto, retrolateral view. Scale bars: 0.10 mm.

**Figure 48.**
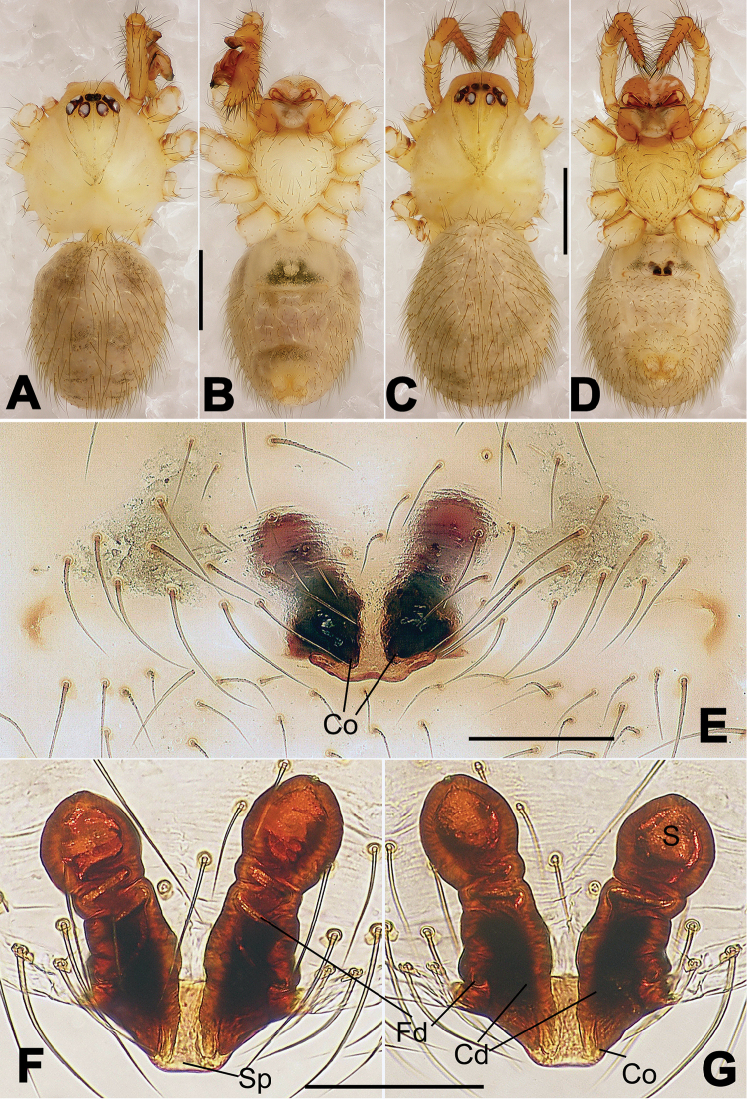
*Nesticella yanbeiensis* sp. n., holotype (male) and paratype (female). **A** Male habitus, dorsal view **B** Ditto, ventral view **C** Female habitus, dorsal view **D** Ditto, ventral view **E** Epigyne, ventral view **F** Vulva, ventral view **G** Vulva, dorsal view. Scale bars: **A–D** = 0.50 mm; **E–G** = 0.10 mm.

Males can be separated from other species of the *mogera*-group by the relatively slim and wavy shape of the process of the conductor (Cp) and by the solid, rectangular distal process of the paracymbium (Dp) (Fig. 47A, D); diagnostic characters for the females are the short, lightly protruding scape (Sp) and the copulatory ducts (Cd) close to each other (Fig. 48E–G). *Nesticella fuliangensis* sp. n. (see Figs 40A–D, 41A–G) seems to be more closely related to *Nesticella yangbeiensis* sp. n., but the males can be distinguished by the thicker and more undulate process of the conductor (Cp) and by the wider and rectangular distal process of the paracymbium (Dp) (slimmer in males of *Nesticella fuliangensis* sp. n.) (Fig. 47A, D vs. Fig. 40A, D). Females of the new species can be recognized by the relatively narrower and more protruding scape (Sp) and by the copulatory ducts which are closer to each other (Cd) (Fig. 48E–G vs. Fig. 41E–G).

##### Description.

Habitus as in Fig. 48A–D. Carapace pale yellow in the male, yellow in females. Cervical groove and fovea distinct. Mouthparts brown-yellowish in males, brownish in females. Sternum pale in males, yellowish in females. Legs uniformly pale yellow. Female tibia and tarsus brown. Opisthosoma greyish, covered with long setae, with four faint greyish dorsal marks. Epigastric area pigmented in males.

Male palp (Fig. 47A–D): paracymbium with a sharp ventral apophysis and a rectangular, stumpy, strongly sclerotized distal process (Fig. 47A–B, D). Well-developed terminal apophysis weakly sclerotized, with a granulate surface (Fig. 47A, C). Conductor with a short and thick, horn-like, strongly sclerotized apophysis at the tip (Fig. 47A, D).

Epigyne (Fig. 48E–G): posterior margin of the scape weakly sclerotized (Fig. 48E). Scape short and square, with a straight posterior margin (Fig. 48F). Fertilization ducts short and swollen, (Fig. 48G). Copulatory ducts wide and twisted, strongly sclerotized and close to each other (Fig. 48G). Spermathecae nearly globular, about as wide as the copulatory ducts and separated by about one diameter (Fig. 48F–G).

Male (holotype). Total length 2.94. Carapace 1.38 long, 1.25 wide. Opisthosoma 1.66 long, 1.17 wide. Clypeus height 0.21. Sternum 0.86 long, 0.79 wide. Leg measurements: see Appendix A.

Female (one of the paratypes). Total length 3.36. Carapace 1.49 long, 1.31 wide. Opisthosoma 2.00 long, 1.41 wide. Clypeus height 0.22. Sternum 0.90 long, 0.82 wide. Leg measurements: see Appendix A.

##### Habitat.

Cave.

##### Distribution.

Known only from the type locality (Fig. 83).

### *Nesticella nepalensis*-group

**Group features.** Males belonging to this species-group can be recognized by the following combination of characters: a ventral apophysis of the paracymbium with two lobed processes bent inward (Va-I, Va-II), Va-I longer and thicker than Va-II and usually ending with an almost round tip; a distal process of the paracymbium with one (more rarely) or two processes (Dp-I and Dp-II), Dp-I protruded ventrally, Dp-II retrolaterally protruded; a usually elongate terminal apophysis (Ta); a small, strongly sclerotized, tegular apophysis (Tg) protruding outward, an additional more or less developed tegular apophysis (Tg-II), and a sclerotized, hook-like process of the conductor (Cp). A diagnostic character for the females is the short or very short, narrow scape (Sp) (approximately as long as wide but remarkably narrower than the species of the *mogera*-group), which is barely visible in some species (wider, longer or with a rounded tip in the females of the other species-groups). Furthermore, the smaller size of the spermathecae (S) easily distinguish females of the *nepalensis*-group from those of the *phami*-group while the thick and bent copulatory ducts (Cd) separate them from those of the *mogera* and *quelpartensis*-groups

**Composition.** *Nesticella aelleni* (Brignoli, 1972), *Nesticella africana* (Hubert, 1970), *Nesticella baobab* sp. n., *Nesticella beccus* Grall & Yäger, 2016, *Nesticella benoiti* (Hubert, 1970), *Nesticella chillagoensis* Wunderlich, 1995, *Nesticella connectens* Wunderlich, 1995, *Nesticella ducke* Rodrigues & Buckup, 2007, *Nesticella gongshanensis* sp. n., *Nesticella griswoldi* sp. n., *Nesticella laotica* Grall & Yäger, 2016, *Nesticella murici* Rodrigues & Buckup, 2007, *Nesticella nepalensis* (Hubert, 1973), *Nesticella potala* sp. n., *Nesticella proszynkii* (Lehtinen & Saaristo, 1980), *Nesticella renata* (Bourne, 1980), *Nesticella robinsoni* Lehtinen & Saaristo, 1980, *Nesticella sechellana* (Simon, 1898), *Nesticella sogi* Lehtinen & Saaristo, 1980, *Nesticella sulawesi* sp. n., *Nesticella utuensis* (Bourne, 1980), *Nesticella taurama* Lehtinen & Saaristo, 1980, *Nesticella tibetana* sp. n., *Nesticella vanlang* sp. n., *Nesticella yui* Wunderlich & Song, 1995, and *Nesticella zhiyuani* sp. n.

#### Nesticella baobab sp. n.

Taxon classificationAnimaliaAraneaeNesticidae

http://zoobank.org/7349B69A-ED88-45E5-BAA4-34FCDCA79C22

Figs 49 , 83

##### Type material.

Holotype ♂ and paratype 1♂ (CASC), MADAGASCAR: Fianarantsoa Province, Parc National Ranomafana, Vohiparara, 3.6 km West of Ranomafana, primary montane rain forest (21.23738°S, 47.39737°E, 1150 m), 13–14.I.2009, C. Griswold, A. Saucedo & H. Wood leg.

##### Etymology.

The specific name is derived from from the word “*baobab*”, the common name for trees of the genus *Adansonia* which are common in Madagascar; noun in apposition.

##### Diagnosis.

**Figure 49.**
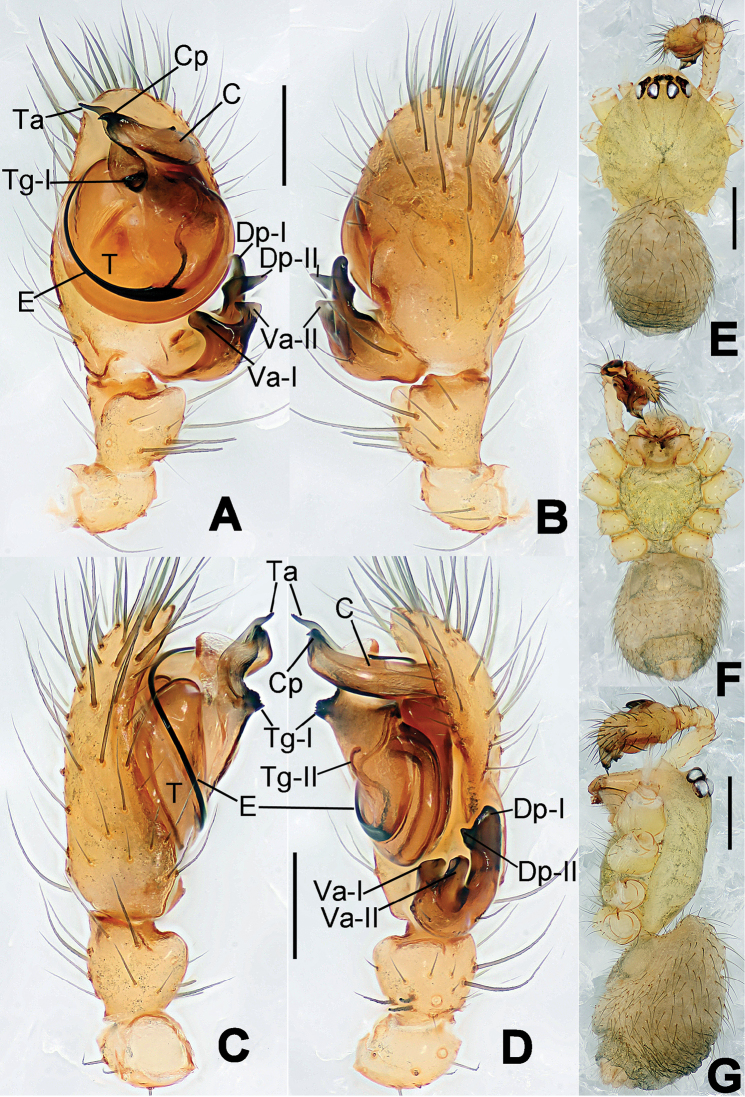
*Nesticella baobab* sp. n., holotype (male). **A** Palp, ventral view **B** Ditto, dorsal view **C** Ditto, prolateral view **D** Ditto, retrolateral view **E** Habitus, dorsal view **F** Ditto, ventral view **G** Ditto, lateral view. Scale bars: **A–D** = 0.10 mm; **E–G** = 0.50 mm.

The new species is similar to *Nesticella benoiti* (see Hubert 1970: 364, figs 5–8) and *Nesticella griswoldi* sp. n. (Figs 53A–D, 54A–B), although the barcode can be used to separate the latter species (see Appendix B). It can be distinguished from *Nesticella benoiti* by the longer terminal apophysis (Ta), the absence of a clearly serrated tegular apophysis (Tg) and the different shape of the distal processes of the paracymbium (Dp-I, Dp-II) (Fig. 49C–D vs. fig. 6). *Nesticella baobab* sp. n. can be distinguished from *Nesticella griswoldi* sp. n. by the slightly protruding process II of the ventral apophysis (Va-II) and the slightly shorter distal process II (Dp-II) (Fig. 49C–D vs. Fig. 53C–D). The same combination of characters allows separation of this species from all the others of the *nepalensis*-group.

##### Description.

Habitus as in Fig. 49E–G. Carapace pale yellow. Cervical groove indistinct, fovea deep. Thoracic area faintly pigmented around the radial furrows. Mouthparts pale yellow. Sternum faintly pigmented, grey, with sparse setae. Legs uniformly yellowish. Opisthosoma greyish, covered with long setae.

Male palp (Fig. 49A–D): paracymbium strongly sclerotized, Va-I elongate with an almost round tip, Va-II short and triangular. Bifurcated distal process with two branches, Dp-I blunt and stocky, Dp-II elongate and sharp (Fig. 49A, B). Terminal apophysis long and sharp, wrinkled and mesially curved (Fig. 49C), basally wide and gradually thinning (Fig. 49A). Tegular apophysis short and strongly sclerotized, located at the base of terminal apophysis and protruding outward (Fig. 49A, C–D); Tg-II barely visible (Fig. 49D). Conductor distally with a sclerotized, short beak-shaped process (Fig. 49A, D).

Male (holotype). Total length 1.96. Carapace 1.03 long, 0.96 wide. Opisthosoma 1.02 long, 0.80 wide. Clypeus height 0.18. Sternum 0.60 long, 0.57 wide. Leg measurements: see Appendix A.

Female. Unknown.

##### Habitat.

Rain forest leaf litter.

##### Distribution.

Known only from the type locality (Fig. 83).

#### Nesticella connectens

Taxon classificationAnimaliaAraneaeNesticidae

Wunderlich, 1995

Figs 50 , 51 , 83

Nesticella connectens Wunderlich, 1995: 568, figs 40–42 (♂).

##### Material examined.

1♂2♀ (IZCAS), THAILAND: Satun Province, Trang District, Beating Cave, Cave A, short wet branch with mud and vegetal remains (07.15965°N, 99.80058°E, 11 m), 03.XII.2013, F. Ballarin leg; 4♀ (IZCAS), Phang Nga Province, Tap pud District, Tharn Lod Cave (08.51897°N, 98.565251°E, 116 m), 27.X.2014, H. Zhao, Y. Li & Z. Chen leg.

##### Diagnosis.

**Figure 50.**
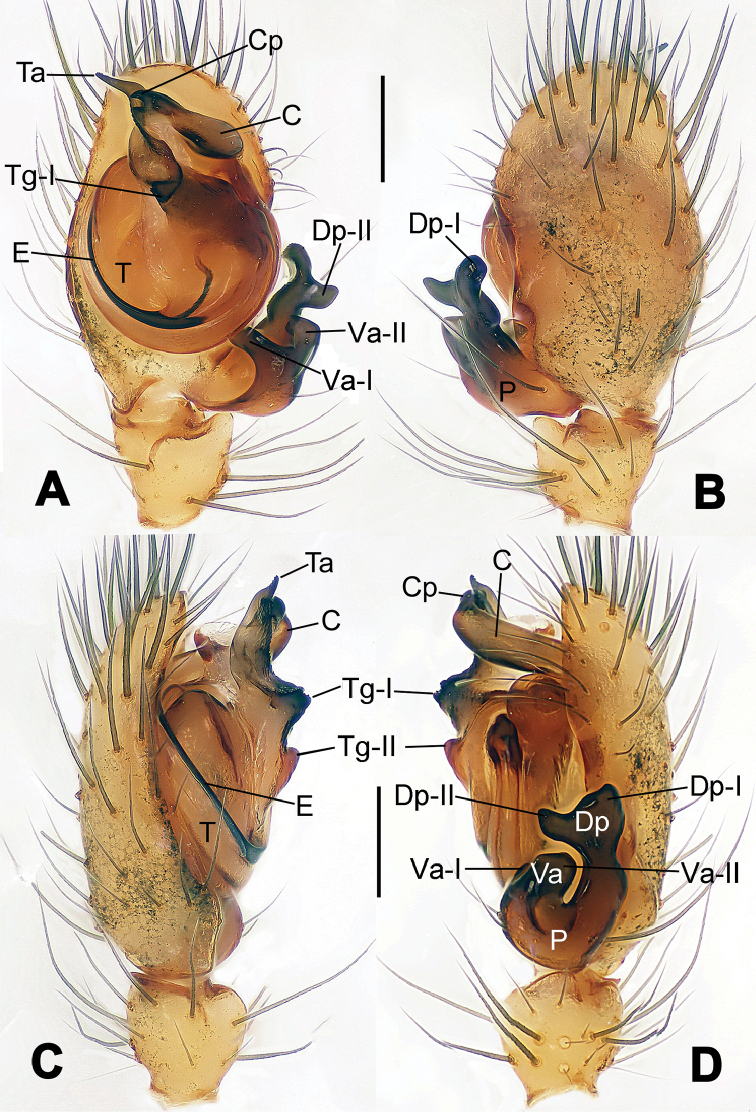
*Nesticella connectens*, male from Beating Cave. **A** Palp, ventral view **B** Ditto, dorsal view **C** Ditto, prolateral view **D** Ditto, retrolateral view. Scale bars: 0.10 mm.

**Figure 51.**
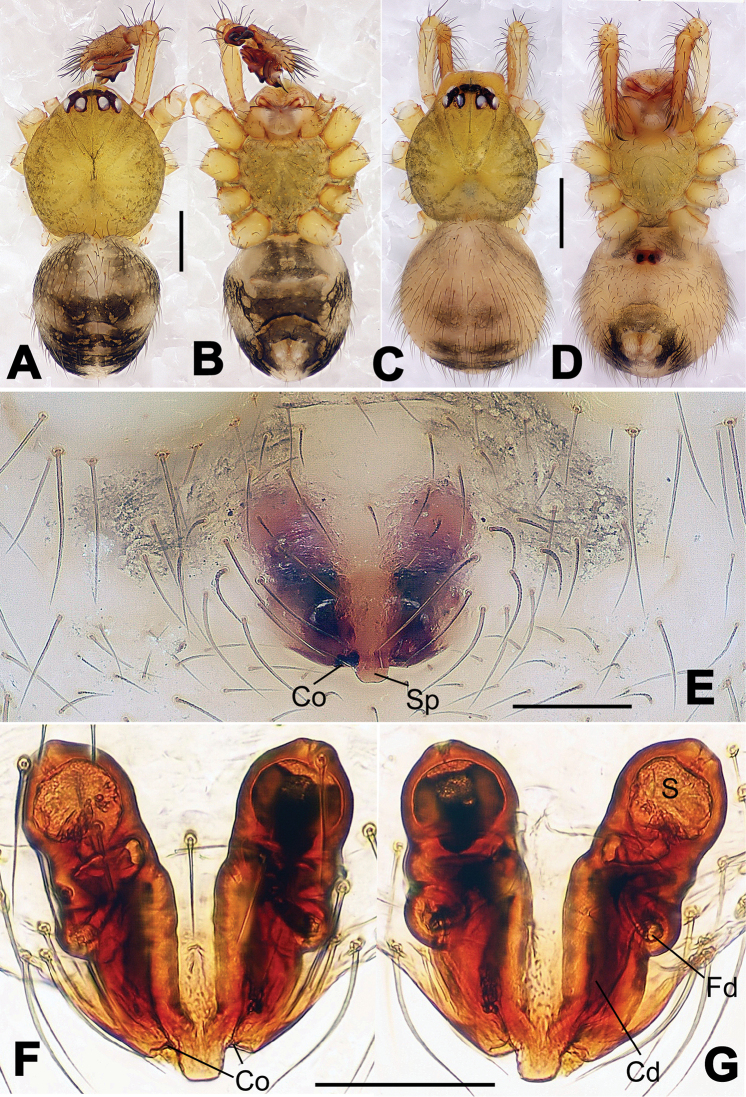
*Nesticella connectens*, male and female from Beating Cave. **A** Male habitus, dorsal view **B** Ditto, ventral view **C** Female habitus, dorsal view **D** Ditto, ventral view **E** Epigyne, ventral view **F** Vulva, ventral view **G** Vulva, dorsal view. Scale bars: **A–D** = 0.50 mm; **E–G** = 0.10 mm.

This species is similar to *Nesticella tibetana* sp. n. (see Figs 59A–D, 60A–G), *Nesticella nepalensis* (see Figs 55A–D, 56A–G) and *Nesticella potala* sp. n. (see Figs 57A–F). Males can be distinguished by the prominent additional tegular apophysis (Tg-II) which is reduced in males of the other two species, by the longer and sharper terminal apophysis (Ta) and by the different shape of the ventral process I (Va-I) which is more bent in males of *Nesticella tibetana* sp. n. and slimmer in those of *Nesticella nepalensis* (Fig. 50A, C–D vs. Fig. 59A, 59C–D vs. Fig. 55A, C–D). Females can be separated from those of *Nesticella tibetana* sp. n. by the less coiled fertilization ducts (Fd) and the longer copulatory ducts (Cd) (Fig. 51G vs. Fig. 60G), from those of *Nesticella nepalensis* by the more prominent scape (Sp) (Fig. 51G vs. Fig. 56G) and from those of *Nesticella potala* sp. n. by the more convoluted copulatory ducts (Cd) and the rounder spermathecae (S) (Fig. 51G vs. Fig. 57G).

##### Description.

Habitus as in Fig. 51A–D. Carapace yellow, faintly pigmented around the radial furrows and the margins. Cervical groove distinct. Fovea shallow. Thoracic area with distinct radial furrows. Mouthparts yellow in males, brown-yellowish in females. Sternum light yellow, with sparse long setae. Legs and female palps yellowish, distally darker in tibiae, metatarsi and tarsi. Opisthosoma light yellow with dark marks in the posterior half and around the spinnerets, darker pigmentation in males.

Male palp (Fig. 50A–D): paracymbium strongly sclerotized, Va-I elongate, ending with an almost round tip, Va-II short and rectangular; bifurcated distal process with two branches, Dp-I blunt, stocky and swollen, Dp-II elongate and sharper (Fig. 50A–B, D). Terminal apophysis long and sharp, wrinkled, basally wide and distally narrow and curved, located behind the process of the conductor (Fig. 50A, C). Tegular apophysis sclerotized and nodular, secondary tegular apophysis well developed, protruding outward from the tegulum. (Fig. 50A, C–D). Conductor with a sclerotized, short, beak-shaped process (Fig. 50A).

Epigyne (Fig. 51E–G): weakly wrinkled and faintly pigmented. Scape square, about as wide as long and weakly sclerotized (Fig. 51E–F). Spermathecae nearly globular, separated by about 2/3 of their diameter (Fig. 51F–G). Fertilization ducts long, reaching the spermathecae with two loops (Fig. 51G). Copulatory ducts thick and straight, distally as wide as the spermathecal diameter (Fig. 51F–G).

Male. Total length 1.93. Carapace 1.05 long, 0.98 wide. Opisthosoma 1.00 long, 0.76 wide. Clypeus height 0.19. Sternum 0.65 long, 0.63 wide. Leg measurements: see Appendix A.

Female. Total length 2.00. Carapace 1.06 long, 0.90 wide. Opisthosoma 1.18 long, 0.90 wide. Clypeus height 0.18. Sternum 0.65 long, 0.59 wide. Leg measurements: see Appendix A.

##### Habitat.

Forest leaf litter, cave.

##### Distribution.

Malaysia, Thailand (first record) (Fig. 83).

##### Remarks.

*Nesticella connectens* was described by Wunderlich (1995) from Peninsular Malaysia based only on males. The collection of both sexes on the Thai side of the Malay Peninsula (approximately 300 km north of the type locality) allows a detailed analysis of this species and the description of the male for the first time.

#### Nesticella gongshanensis sp. n.

Taxon classificationAnimaliaAraneaeNesticidae

http://zoobank.org/97E35365-47CF-4A45-AA42-5CBE0EA206EC

Figs 52 , 83

##### Type material.

Holotype ♀ (IZCAS), CHINA: Yunnan Province, Gongshan County, Langdang Village, beside Dulongjiang River, collected by sieving the leaf litter (27.68844°N, 98.27773°E, 1300 m), 31.VIII.2006, J.A. Miller & D.H. Kavanaugh leg.

##### Etymology.

The specific name is derived from the type locality; adjective.

##### Diagnosis.

**Figure 52.**
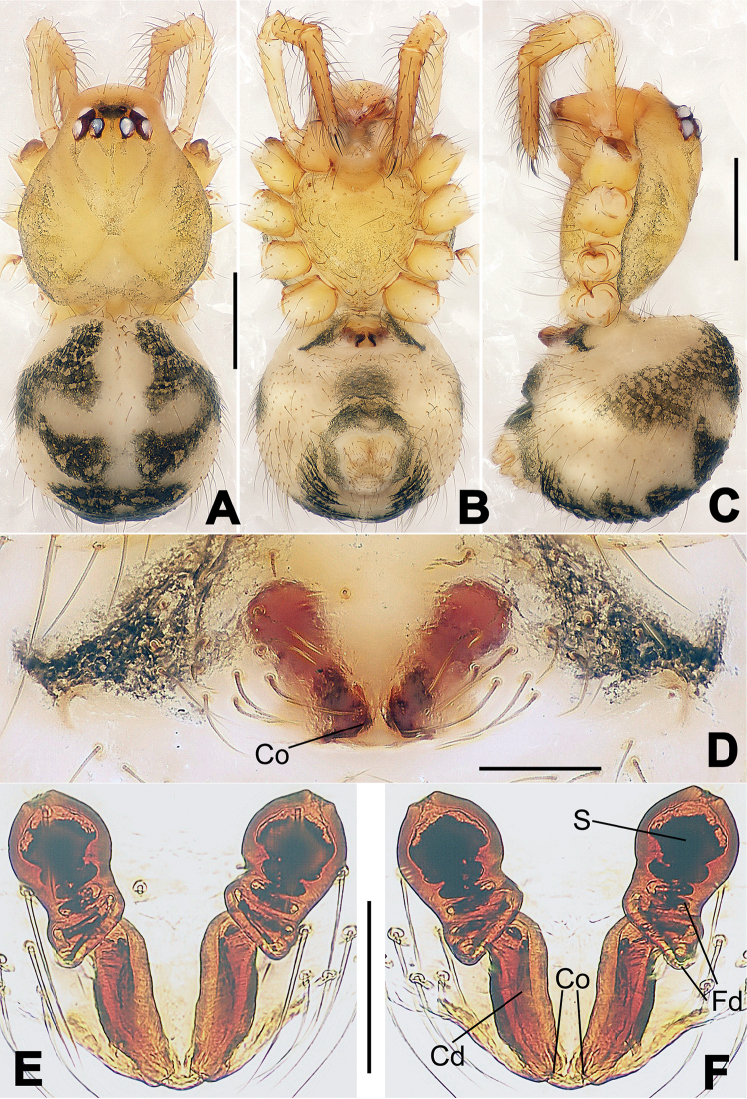
*Nesticella gongshanensis* sp. n., holotype (female). **A** Habitus, dorsal view **B** Ditto, ventral view **C** Ditto, lateral view **D** Epigyne, ventral view **E** Vulva, ventral view **F** Vulva, dorsal view. Scale bars: **A–C** = 0.50 mm; **D–F** = 0.10 mm.

This new species is closely related to *Nesticella yui* (see Fig. 62A–F), but can be distinguished from the latter by the narrower scape (Sp), the narrower space between the copulatory openings (Co) and the different shape of copulatory ducts (Cd) (Fig. 52F vs. Fig. 62F). The small scape (Sp), only faintly visible, and the V-shaped copulatory ducts (Cd) allow the separation of this species from all the others belonging to the *nepalensis*-group.

##### Description.

Habitus as in Fig. 52A–C. Carapace yellow, faintly pigmented in the cephalic area and at margins. Cervical groove and fovea distinct. Mouthparts yellowish. Sternum yellow with sparse long setae and additional thin setae at margins. Legs and female palps yellowish, distally darker in metatarsi and tarsi. Opisthosoma pale yellowish, and with paired dark marks, partially connected each other in the dorsal area, bordering a light, cross-shaped mark in center. Other dark markings present laterally, at the epigastric and hypogastric area and around the spinnerets.

Epigyne (Fig. 52D–F): wrinkled and translucent, scape strongly reduced and barely visible (Fig. 52D–F). Copulatory openings close to each other near the posteromargin of the epigyne (Fig. 52F–G). Spermathecae almost round, separated by about 1.3 diameters. Fertilization ducts thin and long, coiled into 2.5 loops until reaching the spermathecae (Fig. 52F). Copulatory ducts thick (Fig. 52E–F).

Female (holotype). Total length 2.38. Carapace 1.24 long, 1.07 wide. Opisthosoma 1.22 long, 1.10 wide. Clypeus height 0.23. Sternum 0.73 long, 0.66 wide. Leg measurements: see Appendix A.

Male. Unknown.

##### Habitat.

Forest leaf litter.

##### Distribution.

Known only from the type locality (Fig. 83).

#### Nesticella griswoldi sp. n.

Taxon classificationAnimaliaAraneaeNesticidae

http://zoobank.org/BCE51948-D7D5-4E37-9214-4800DD5C46FA

Figs 53 , 54 , 83

##### Type material.

Holotype ♂ and paratypes 1♂1♀ (CASC), MADAGASCAR: Toliara Province, Forest Classee Tsitongambarika, Cascade hiking trail, 7.5 km Northwest of Taolagnaro, primary montane rain forest (24.98664°S, 46.92631°E, 100 m), 24.XII.2008, F. Alvarez-Padilla & H. Wood leg.

##### Etymology.

The new species is named after Dr. Charles Griswold, a leading spider taxonomist from the USA; noun (name) in genitive case.

##### Diagnosis.

**Figure 53.**
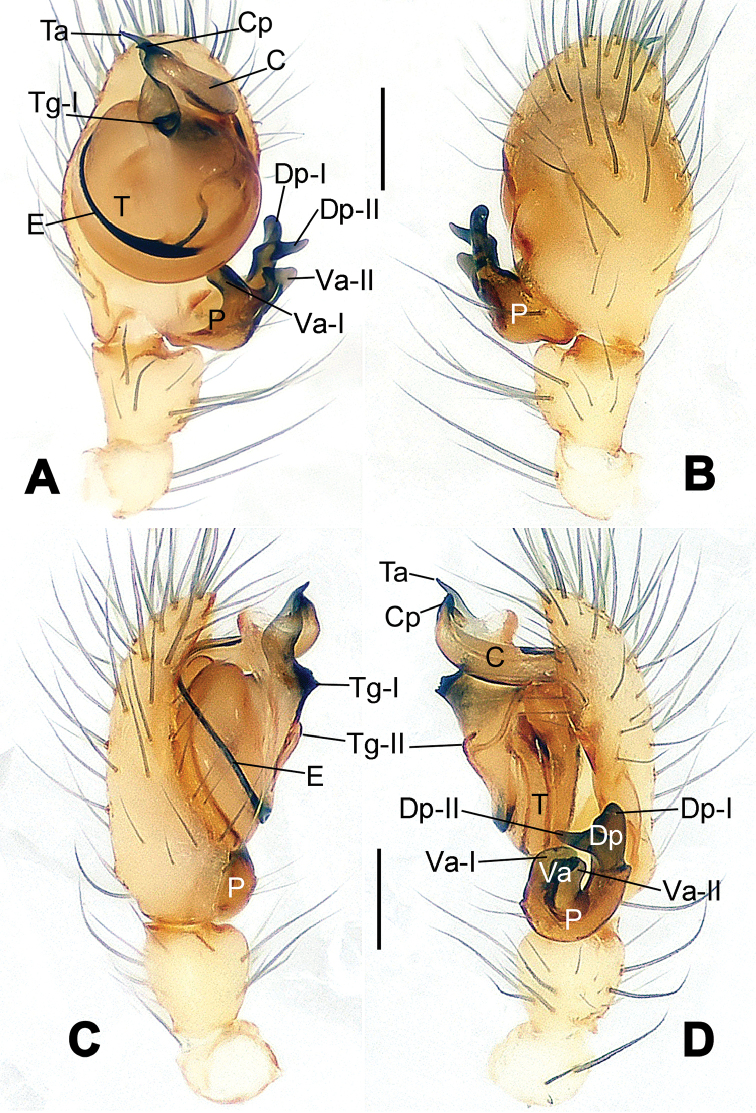
*Nesticella griswoldi* sp. n., holotype (male). **A** Palp, ventral view **B** Ditto, dorsal view **C** Ditto, prolateral view **D** Ditto, retrolateral view. Scale bars: 0.10 mm.

**Figure 54.**
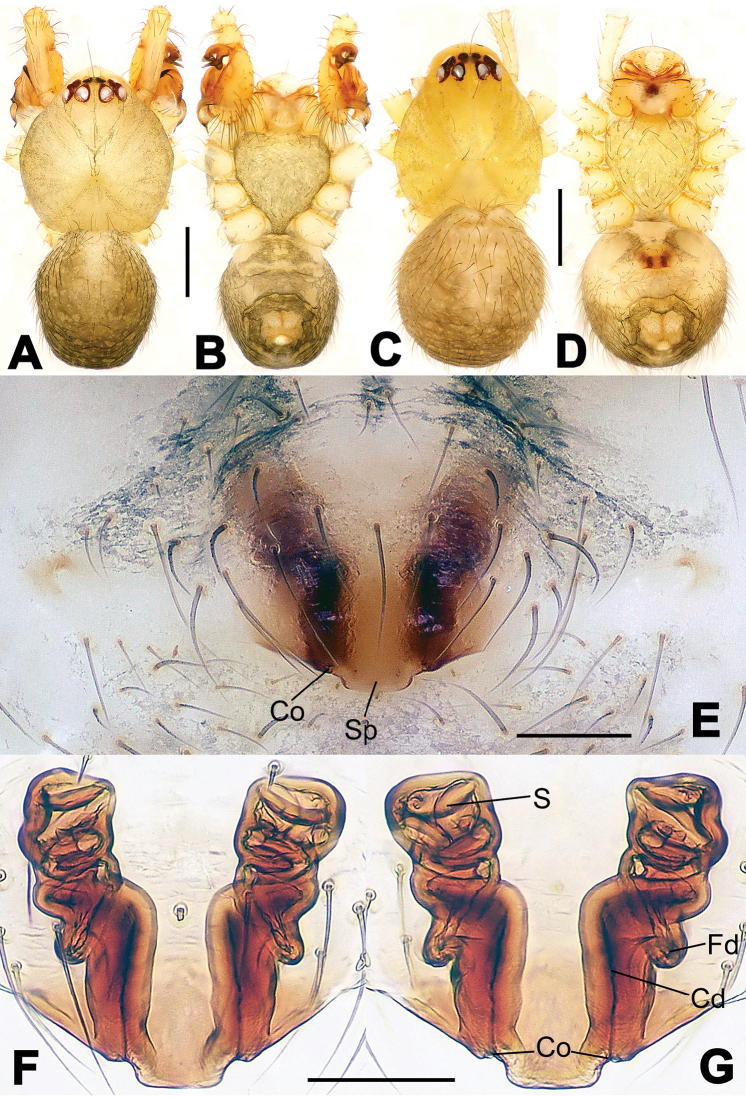
*Nesticella griswoldi* sp. n., holotype (male) and paratype (female). **A** Male habitus, dorsal view **B** Ditto, ventral view **C** Female habitus, dorsal view **D** Ditto, ventral view **E** Epigyne, ventral view **F** Vulva, ventral view **G** Vulva, dorsal view. Scale bars: **A–D** = 0.50 mm; **E–G** = 0.10 mm.

The new species is similar to *Nesticella benoiti* (see Hubert 1970: 364, figs 5–8), *Nesticella baobab* sp. n. (see Fig. 49A–G) and *Nesticella vanlang* sp. n. (see Fig. 61A–F). Males can be distinguished from those of *Nesticella benoiti* by the longer terminal apophysis (Ta), the shorter tegular apophysis (Tg) lack of a deep serration, and by the longer, sharper distal process II of the paracymbium (Dp-II) (Fig. 53A–B, D vs. fig. 6). Males of *Nesticella griswoldi* sp. n. can be separated from those of *Nesticella baobab* sp. n. by the slightly wider process II of the ventral apophysis (Va-II) and the slightly longer distal process II (Dp-II) (Fig. 53A–B, D vs. Fig. 49A–B, D). Females can be recognized from those of *Nesticella benoiti* and *Nesticella vanlang* sp. n. by the short, rectangular scape (Sp) (narrower and pointed in *Nesticella benoiti*, shorter and less evident in *Nesticella vanlang* sp. n.) and by the distance between the spermathecae (S) (close to each other in *Nesticella vanlang* sp. n.) (Fig. 54E–G vs. figs 7–8 vs. Fig. 61D–F). The same combination of characters allows separating this species from all the others belonging to the *nepalensis*-group.

##### Description.

Habitus as in Fig. 54A–D. Carapace pale yellow in males, yellow in females. Cervical groove and fovea distinct. Mouthparts yellow. Sternum yellow, darkish pigmented in males. Legs and female palps pale yellow, distally darker in metatarsi and tarsi. Opisthosoma pale yellow, with faint grey dark and covered with long setae.

Male palp (Fig. 53A–D): paracymbium wide, Va-I elongate and an almost round tip, Va-II short and triangular with a double point; bifurcated distal process with two branches, Dp-I blunt and stocky, Dp-II elongate and sharp. Terminal apophysis long and twisted, basally laminar and distally sharp, weakly sclerotized (Fig. 53A). Tegular apophysis located at the base of the terminal apophysis, sclerotized and protruding outward, apically lightly serrated, tegular apophysis II small (Fig. 53A, D). Conductor with a short, sclerotized hooked distal process (Fig. 53A, D).

Epigyne (Fig. 54E–G): faintly pigmented with a translucent tegument (Fig. 54E). Scape short and rectangular (Fig. 54E, G). Spermathecae ovate (slightly compressed after treated with lactic acid) (Fig. 54F–G). Fertilization ducts thin and coiled, forming only one loop before reaching the spermathecae (Fig. 54G). Copulatory ducts thick, ventrally oriented in spermathecae, distally bent outward (Fig. 54G).

Male (holotype). Total length 1.93. Carapace 1.05 long, 0.98 wide. Opisthosoma 1.00 long, 0.76 wide. Clypeus height 0.19. Sternum 0.65 long, 0.63 wide. Leg measurements: see Appendix A.

Female (one of the paratypes). Total length 2.00. Carapace 1.06 long, 0.90 wide. Opisthosoma 1.18 long, 0.90 wide. Clypeus height 0.18. Sternum 0.65 long, 0.59 wide. Leg measurements: see Appendix A.

##### Habitat.

Rain forest leaf litter.

##### Distribution.

Known only from the type locality (Fig. 83).

#### Nesticella nepalensis

Taxon classificationAnimaliaAraneaeNesticidae

(Hubert, 1973)

Figs 55 , 56 , 83

Nesticus nepalensis Hubert, 1973: 165, figs 2–5 (♂♀).
Nesticella nepalensis : Lehtinen and Saaristo 1980: 55 (transfer from Nesticus).
Nesticella nepalensis : Marusik and Guseinov 2003: 38, figs 22–24 (♂).

##### Material examined.

1♂ (IBPN), INDIA: Uttarakhand State, Gobind Ghat Village (30.625°N, 79.55833°E, 1900 m), 17–23.V.1999, Y.M. Marusik leg. 2♀ (IZCAS), CHINA: Tibet Autonomous Region, Dinggye County, Chentang Town (27.91458°N, 87.48098°E, 3267 m), 3.VIII.2014, Y. Li leg.

##### Diagnosis.

**Figure 55.**
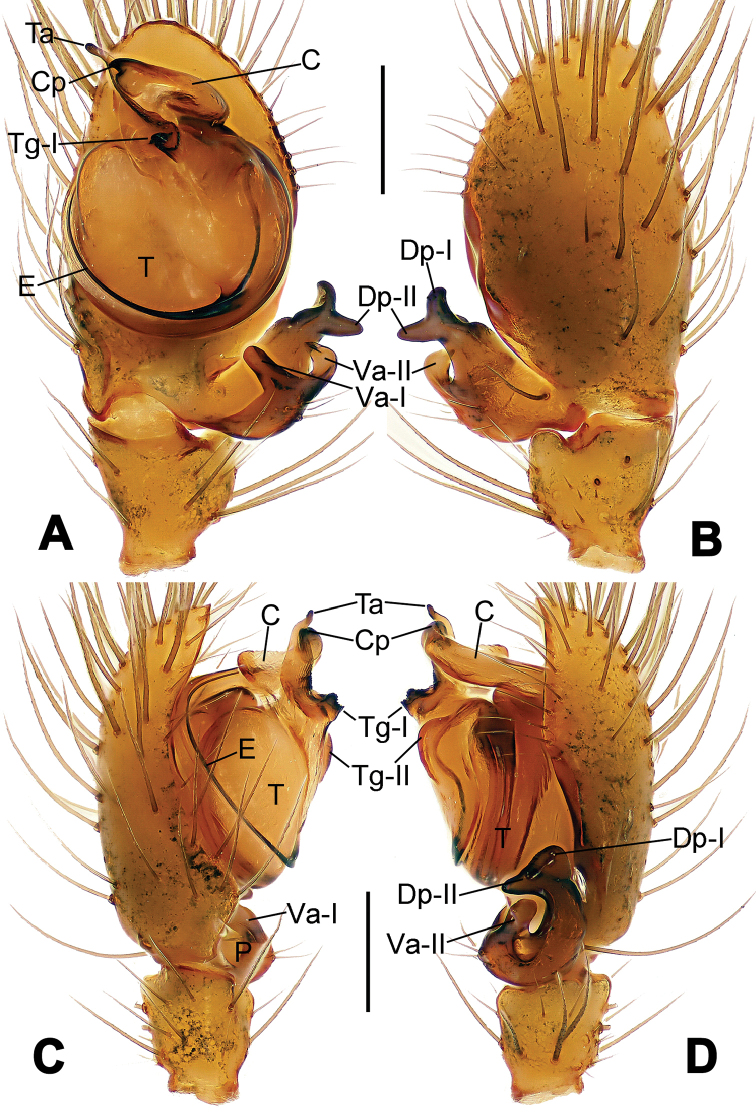
*Nesticella nepalensis*, male from Gobind Ghat. **A** Male palp, ventral view **B** Ditto, dorsal view **C** Ditto, prolateral view **D** Ditto, retrolateral view. Scale bars: 0.10 mm.

**Figure 56.**
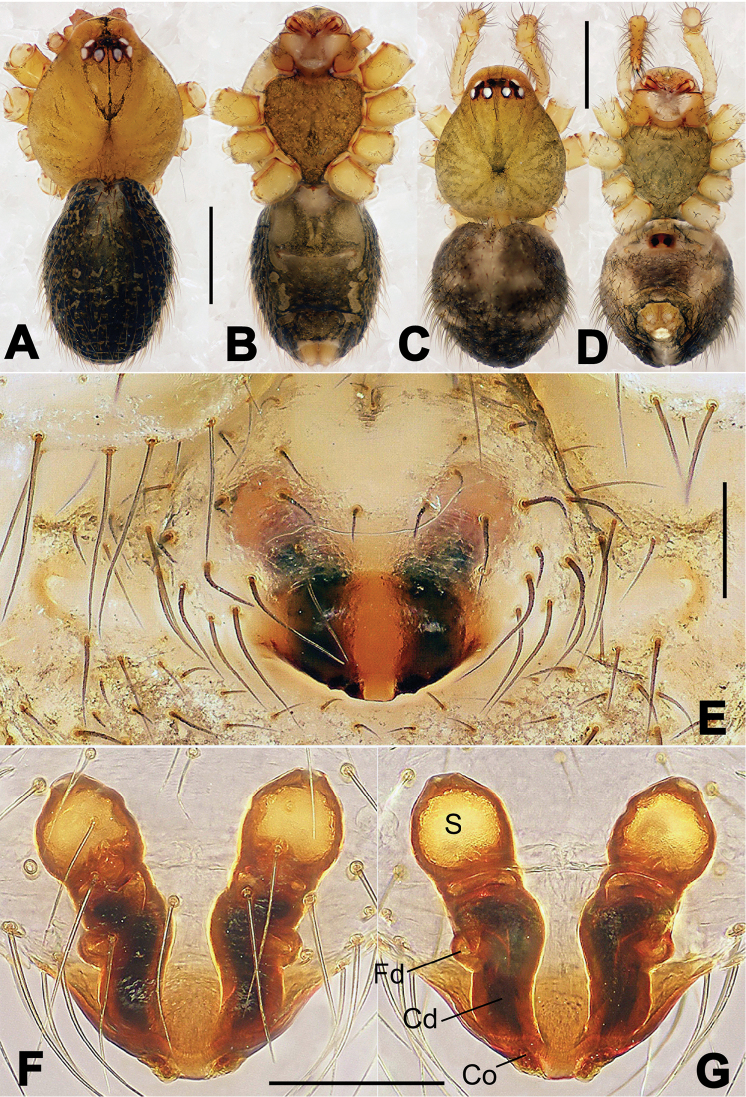
*Nesticella nepalensis*, male from Gobind Ghat and female from Chentang. **A** Male habitus, dorsal view **B** Ditto, ventral view **C** Female habitus, dorsal view **D** Ditto, ventral view **E** Epigyne, ventral view **F** Vulva, ventral view **G** Ditto, dorsal view. Scale bars: **A–D** = 0.50 mm; **E–G** = 0.10 mm.

**Figure 57.**
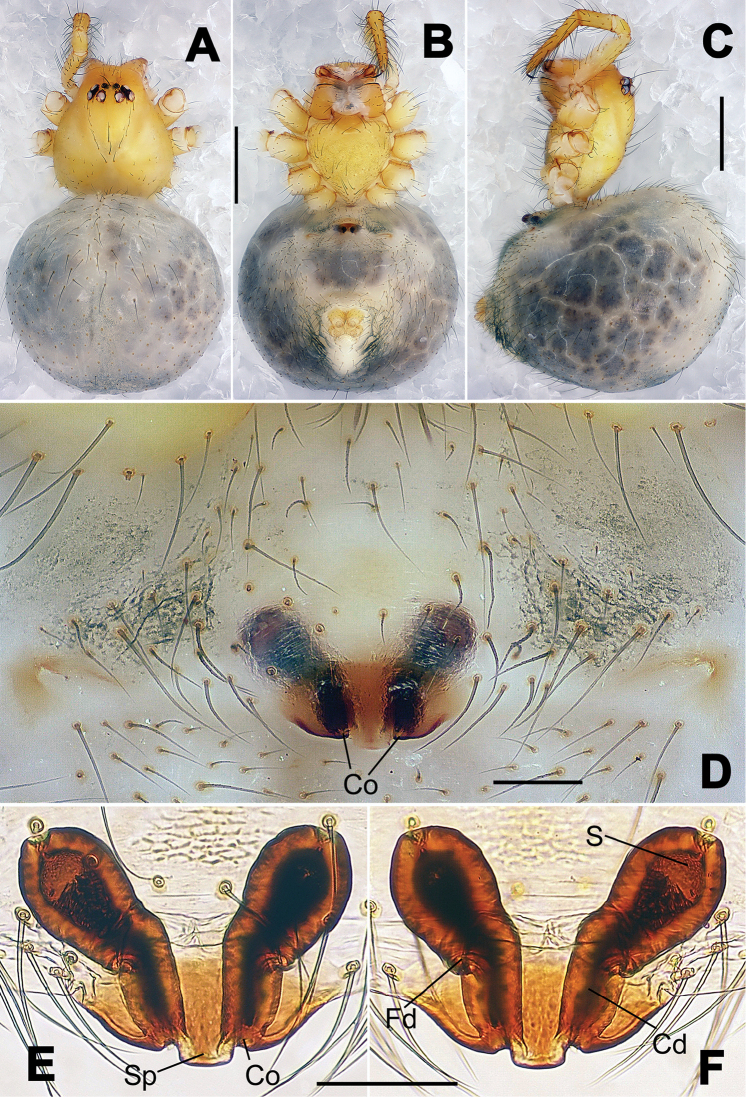
*Nesticella potala* sp. n., holotype (female). **A** Habitus, dorsal view **B** Ditto, ventral view **C** Ditto, lateral view **D** Epigyne, ventral view **E** Vulva, ventral view **F** Vulva, dorsal view. Scale bars: **A–C** = 0.50 mm; **D–F** = 0.10 mm.

*Nesticella nepalensis* is similar to *Nesticella connectens* (see Figs 50A–D, 51A–G), *Nesticella tibetana* sp. n. (see Figs 59A–D, 60A–G) and *Nesticella potala* sp. n. (see Figs 57D–F). Males can be recognized by the blunt tip of the terminal apophysis (Ta) and by the straighter and slimmer ventral process I of the paracymbium (Va-I) which are respectively sharper, more bent and thicker in males of the other species (Fig. 55A, C–D vs. Fig. 50A, C–D vs. Fig. 59A, C–D). Females can be separated from those of *Nesticella connectens* by the less prominent scape (Sp) (Fig. 56 E–G vs. Fig. 51E–G), from those of *Nesticella potala* sp. n. by the almost round spermathecae (S) instead of triangular spermathecae and by the coiled fertilization ducts (Fd), which are lacking in *Nesticella potala* sp. n. (Fig. 56 E–G vs. see Figs 57D–F), and finally from those of *Nesticella tibetana* sp. n. by the shorter scape (Sp), the smaller spermathecae (S) and the less twisted fertilization ducts (Fd) (Fig. 56E–G vs. Fig. 60E–G).

##### Description.

See Figs 55A–D, 56A–G and Hubert (1973).

##### Habitat.

Forest leaf litter, under stone.

##### Distribution.

China (Tibet) (first record) (Fig. 83), India (Uttarakhand), Nepal.

#### Nesticella potala sp. n.

Taxon classificationAnimaliaAraneaeNesticidae

http://zoobank.org/5173B6B5-A3AF-4E69-8165-B9326A46B381

Figs 57 , 83

##### Type material.

Holotype ♀ (IZCAS), CHINA: Tibet Autonomous Region, Gyirong County, Zongga Town, Zalong Village (28.38108°N, 85.35263°E, 2715 m), 31.VII.2014, X. Li & Y. Li leg.

##### Etymology.

The specific name derives from the Potala Palace, one of the most famous Buddhist holy land in the world, which is located near the type locality of the species; noun in apposition.

##### Diagnosis.

The new species is closely related to *Nesticella tibetana* sp. n. (see Fig. 60C–G), *Nesticella nepalensis* (see Fig. 56C–G) and *Nesticella connectens* (see Fig. 51C–G). It can be distinguished by the triangular spermathecae (S), the 45° angle made by the copulatory ducts (Cd) (approx. 90° in the other species), the short fertilization ducts lacking coils, and the lack of a clear abdominal pattern (Fig. 57A–F). All these charaters have no similarities to all the other species of the *nepalensis*-group.

##### Description.

Habitus as in Fig. 57A–C. Carapace yellow, with short setae near the clypeus and the thoracic area, long setae near the cephalic midline and cervical groove. Cervical groove distinct, fovea shallow. Mouthparts brown-yellowish. Sternum flat, faintly reticulated in the center, with both long and short setae at its margins. Legs and female palps yellowish, distally darker in metatarsi and tarsi. Opisthosoma greyish and faintly pigmented with darkish spots. Spinnerets yellow, colulus yellowish.

Epigyne (Fig. 57D–F): wrinkled and translucent (Fig. 57D). Scape short and rectangular, approximately two times wider than long (Fig. 57E–F). Spermathecae triangular, separated by about their length. Fertilization ducts thin, without complicated coils and only one loop before reaching the spermathecae (Fig. 57F). Copulatory ducts thick and straight, laterally bent in the middle (Fig. 57E–F).

Female (holotype). Total length 3.20. Carapace 1.37 long, 1.20 wide. Opisthosoma 2.13 long, 2.18 wide. Clypeus height 0.25. Sternum 0.84 long, 0.83 wide. Leg measurements: see Appendix A.

Male. Unknown.

##### Habitat.

Forest leaf litter.

##### Distribution.

Known only from the type locality (Fig. 83).

#### Nesticella sulawesi sp. n.

Taxon classificationAnimaliaAraneaeNesticidae

http://zoobank.org/23AE24D4-C73D-46A7-A1C0-2327095C97C8

Figs 58 , 83

##### Type material.

Holotype ♀ and paratypes 2♀ (IZCAS), INDONESIA: South Sulawesi, Cenrana Village, 0–4 km east to Maros Water Park (05.05429°S, 119.73958°E, 229 m), 24.VIII.2014, H. Zhao & Z. Yao leg.

##### Etymology.

The specific name is derived from the Island of Sulawesi were this species was collected; noun in apposition.

##### Diagnosis.

**Figure 58.**
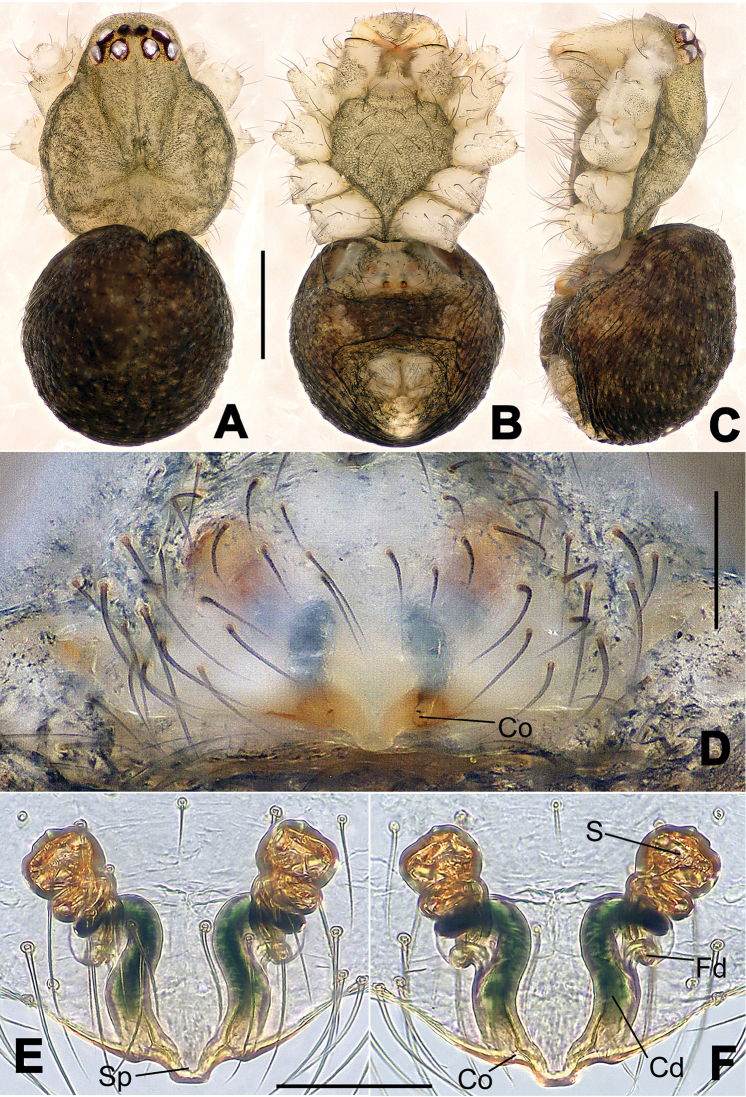
*Nesticella sulawesi* sp. n., holotype (female). **A** Female habitus, dorsal view **B** Ditto, ventral view **C** Ditto, lateral view **D** Epigyne, ventral view **E** Vulva, ventral view **F** Vulva, dorsal view. Scale bars: **A–C** = 0.50 mm; **D–F** = 0.10 mm.

This new species can be distinguished from all the others of the *nepalensis*-group with the exception of *Nesticella yui* by the small spermathecae (S) and the twisted copulatory ducts (Cd) firstly bent inward and then outward before reaching the spermathecae (Fig. 58E–F). It differs from *Nesticella yui* (see Fig. 62A–F; Wunderlich and Song 1995: 347, fig. 19) by the presence of a clear, short and narrow scape (Sp), almost absent in the other species, by the shorter fertilization ducts (Fd) with fewer coils, and the distally wider copulatory ducts (Cd) (Fig. 58F vs. Fig. 62F). A further diagnostic character is the uniformly dark color of the opisthosoma lacking any pattern (Fig. 58A–C vs. Fig. 62A–C).

##### Description.

Habitus as in Fig. 58A–C. Carapace pale yellow, with dense, dark marks. Cervical groove, fovea and radial furrows distinct. Thoracic area dark at its margins. Mouthparts pale yellow, faintly pigmented. Sternum with a very sharp posterior corner, with a reticulated and finely pigmented surface. Legs and female palps pale, lacking spines. Opisthosoma black, densely covered with setae.

Epigyne (Fig. 58D–F): light-colored (Fig. 58D). Scape short and narrow, rectangular and slightly protruding beyond the epigynal posterior margin (Fig. 58D–E). Spermathecae weakly sclerotized, ovoid, separated by about two diameters (Fig. 58E). Fertilization ducts thin and long, forming a small and a large loop before reaching the spermathecae (Fig. 58F). Copulatory ducts thick and dark, firstly bent inward and then outward, mesially swollen and distally narrower (Fig. 58E–F).

Female (holotype). Total length 2.33. Carapace 1.18 long, 1.05 wide. Opisthosoma 1.15 long, 1.00 wide. Clypeus height 0.19. Sternum 0.71 long, 0.68 wide. Leg measurements: see Appendix A.

Male. Unknown.

##### Habitat.

Rain forest leaf litter.

##### Distribution.

Known only from the type locality (Fig. 83).

#### Nesticella tibetana sp. n.

Taxon classificationAnimaliaAraneaeNesticidae

http://zoobank.org/8DB3F9C9-4C03-4712-96F5-7E8FC253F76A

Figs 59 , 60 , 83

##### Type material.

Holotype ♂ and paratypes 1♂1♀ (IZCAS), CHINA: Tibet Autonomous Region, Bowo County, Yigong Town (30.26715°N, 94.77855°E, 2183 m), 13.VIII.2014, Y. Li leg; paratypes 1♂3♀ (IZCAS): Tibet Antonomous Region, Nyingchi Prefecture, at 80 km of Bomi to Motuo Road (29.68590°N, 95.83965°E, 2290 m), 11.VIII.2013, Y. Li leg.

##### Etymology.

The specific name is derived from the type locality; adjective.

##### Diagnosis.

**Figure 59.**
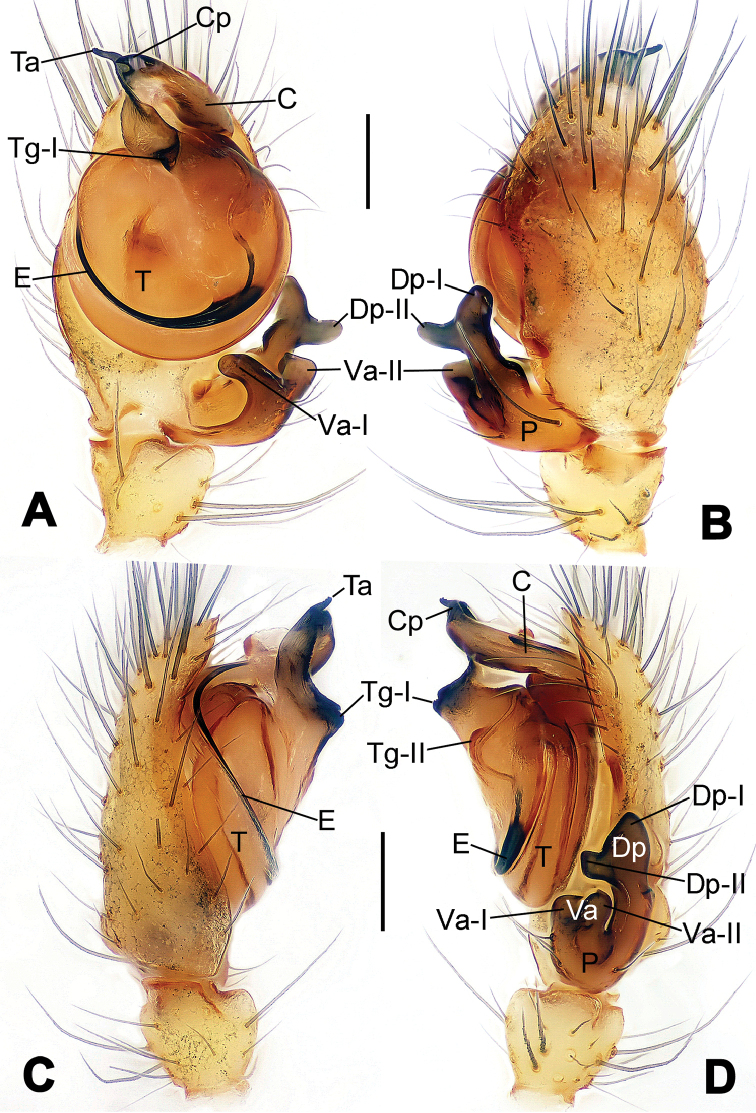
*Nesticella tibetana* sp. n., holotype (male). **A** Palp, ventral view **B** Ditto, dorsal view **C** Ditto, prolateral view **D** Ditto, retrolateral view. Scale bars: 0.10 mm.

**Figure 60.**
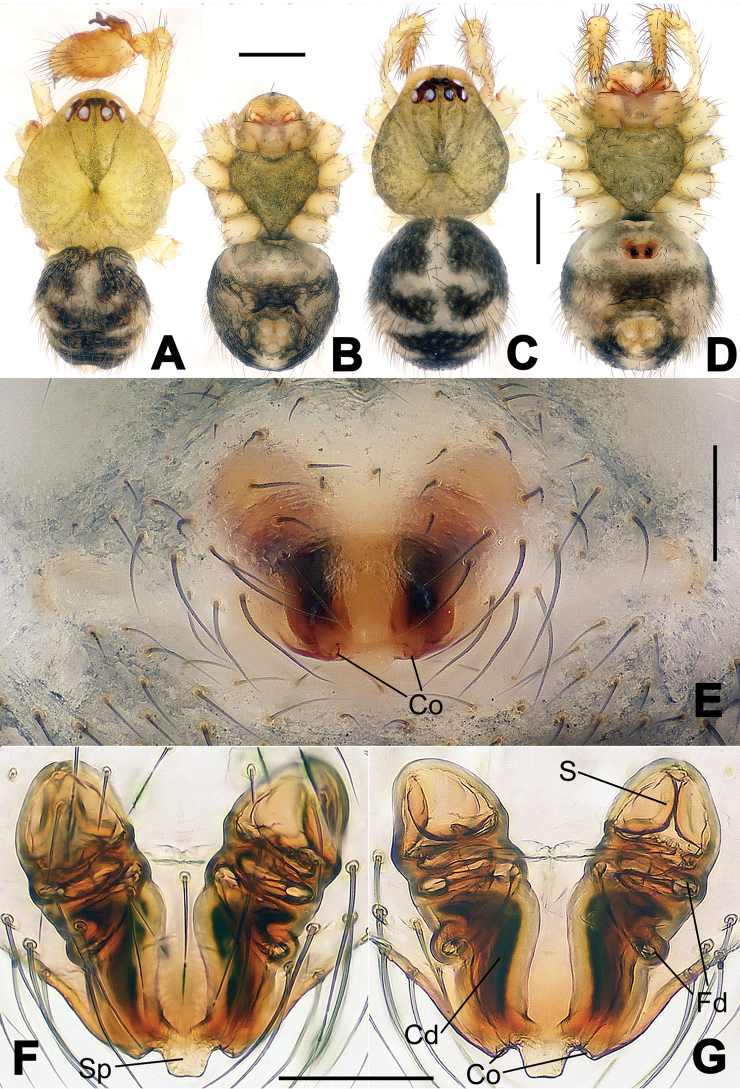
*Nesticella tibetana* sp. n., holotype (male) and paratype (female). **A** Male habitus, dorsal view **B** Ditto, ventral view **C** Female habitus, dorsal view **D** Ditto, ventral view **E** Epigyne, ventral view **F** Vulva, ventral view **G** Vulva, dorsal view. Scale bars: **A–D** = 0.50 mm; **E–G** = 0.10 mm.

The new species is closely related to *Nesticella connectens* (see Figs 50A–D, 51A–G), *Nesticella nepalensis* (see Figs 55A–D, 56A–G) and *Nesticella potala* sp. n. (see Fig. 57A–F). Males can be recognized by the curved process II of the ventral apophysis (Va-II), the stockier distal process II (Dp-II) and the different shape of the terminal apophysis (Ta) (Fig. 59A–B, D vs. Fig. 50A–B, D vs. Fig. 55A–B, D). Females can be distinguished from those of *Nesticella connectens* by the more coiled fertilization ducts (Fd) and the shorter copulatory ducts (Cd) (Fig. 60G vs. Fig. 51G); from those of *Nesticella nepalensis* by the longer scape (Sp), the wider spermathecae (S) and the more twisted fertilization ducts (Fd) (Fig. 60E–G vs. Fig. 56E–G); and finally from those of *Nesticella potala* sp. n. by the almost round spermathecae (S), the 90° angle of the copulatory ducts (Cd) (approx. 45° in *Nesticella potala* sp. n.) and by the presence of a clear abdominal pattern which is absent in the other species (Fig. 60E–G vs. Fig. 57D–F).

##### Description.

Habitus as in Fig. 60A–D. Carapace yellowish in males, darker in females. Cervical groove distinct, fovea deep. Thoracic area with weak radial furrows. Mouthparts pale yellow. Sternum greyish. Legs and female palps yellowish, distally darkish in metatarsi and tarsi. Opisthosoma covered with long setae, yellowish with paired dark marks, partially merged each other on the dorsal area and bordering a cross-like light mark.

Male palp (Fig. 59A–D): paracymbium strongly sclerotized, Va-I elongate and curved with an almost round tip, Va-II short and rectangular. Bifurcated distal process with two branches, Dp-I longer and sharp, Dp-II blunt and stocky. Terminal apophysis elongate, thinner and curved, with a rounded tip (Fig. 59A). Tegular apophysis protruding outward with two sclerotized nodules, Tg-II small and barely visible (Fig. 59A, C–D). Conductor with a sclerotized, short, beak-shaped process (Fig. 59A, D).

Epigyne (Fig. 60E–G): weakly sclerotized (Fig. 60E). Scape short and translucent, rectangular, with slightly protruding, concaved lateral margins (Fig. 60E–F). Spermathecae wide, ovoid, separated by about 0.8 diameter (Fig. 60G). Fertilization ducts thin and long, coiled into three loops before reaching the spermathecae (Fig. 60G). Copulatory ducts thick and relatively short, distally bent outward (Fig. 60F–G).

Male (holotype). Total length 2.08. Carapace 1.09 long, 1.00 wide. Opisthosoma 1.00 long, 0.98 wide. Clypeus height 0.20. Sternum 0.67 long, 0.67 wide. Leg measurements: see Appendix A.

Female (one of the paratypes). Total length 2.20. Carapace 1.10 long, 1.01 wide. Opisthosoma 1.20 long, 1.04 wide. Clypeus height 0.20. Sternum 0.66 long, 0.66 wide. Leg measurements: see Appendix A.

##### Habitat.

Forest leaf litter.

##### Distribution.

Known only from the type locality (Fig. 83).

#### Nesticella vanlang sp. n.

Taxon classificationAnimaliaAraneaeNesticidae

http://zoobank.org/A7B242B3-74E9-4A1A-960F-5009C5D633FF

Figs 61 , 83

##### Type material.

Holotype ♀ (IZCAS), VIETNAM: Ninh Thuan Province, Nui Chua National Park (11.73305°N, 107.18550°E, 102 m), 31.VIII.2015, Q. Zhao, Y. Li & Z. Chen leg.

##### Etymology.

The specific name is derived from Van Lang, an ancient name for Vietnam; noun in apposition.

##### Diagnosis.

**Figure 61.**
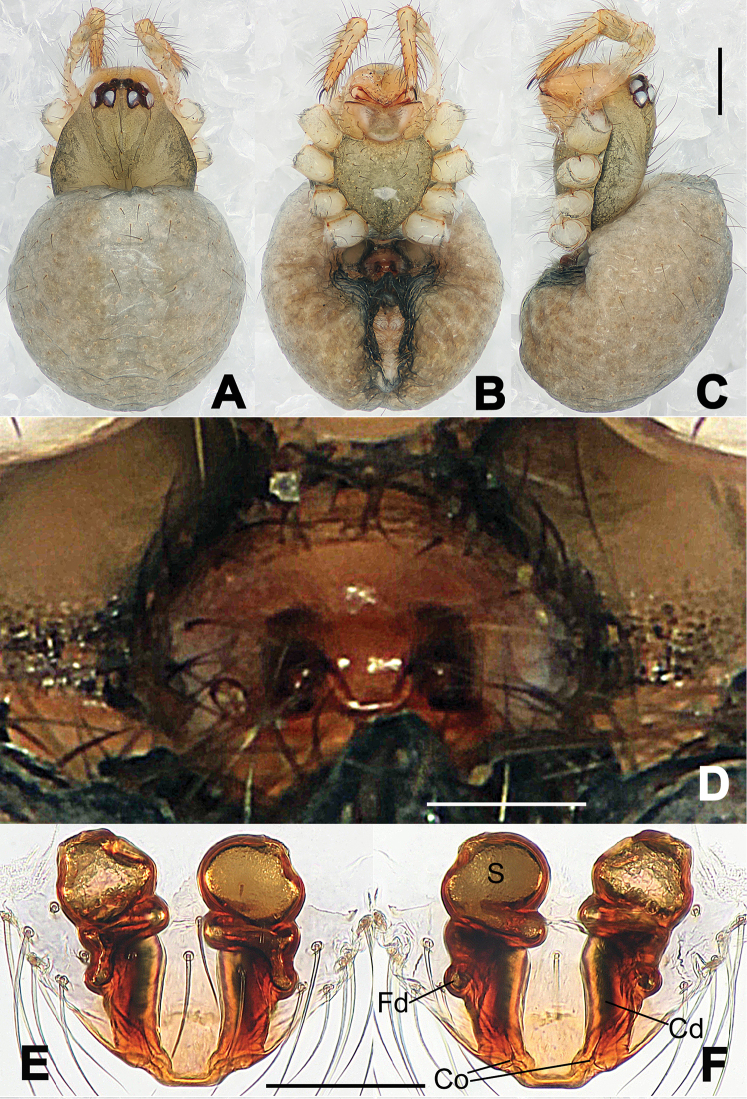
*Nesticella vanlang* sp. n., holotype (female). **A** Habitus, dorsal view **B** Ditto, ventral view **C** Ditto, lateral view **D** Epigyne, ventral view **E** Vulva, ventral view **F** Vulva, dorsal view. Scale bars: **A–C** = 0.50 mm; **D–F** = 0.10 mm.

This new species is distinguished from all the other species of the *nepalensis*-group with the exception of *Nesticella sogi* by the straight fertilization ducts (Fd) and the wide spermathecae (S) which are close to each other, separated by less than a half of their diameter (Fig. 61E–F). *Nesticella vanlang* sp. n. can be distinguished from *Nesticella sogi* (see Lehtinen and Saaristo1980: 57, fig. 21) by the smaller size of the spermathecae (S) and the longer copulatory ducts (Cd) (Fig. 61F vs. fig. 21).

##### Description.

Habitus as in Fig. 61A–C. Carapace greyish and faintly pigmented. Cephalic area with several long setae along the midline and the thoracic groove. Cervical groove and fovea distinct. Thoracic area pigmented at margins. Mouthparts pale yellow. Labium very wide. Sternum smooth and greyish. Legs and female palps yellowish, metatarsi and tarsi distally darker. Opisthosoma pale greyish, darker ventrally.

Epigyne (Fig. 61D–F): strongly clerotized, reddish-brown (Fig. 61E). Scape rectangular, very short and barely visible, (Fig. 61E–F). Copulatory openings near the posteromargin of the epigyne, separated from each other by more than a half of the spermathecal diameter (Fig. 61E–F). Spermathecae almost round, close to each other and separated by less than a half of their diameter (Fig. 61F). Fertilization ducts thick, coiled into a single loop before reaching the spermathecae (Fig. 61E–F). Copulatory ducts thick and short (Fig. 61F).

Female (holotype). Total length 2.23. Carapace 1.11 long, 0.96 wide. Opisthosoma 1.45 long, 1.55 wide. Clypeus height 0.17. Sternum 0.61 long, 0.65 wide. Leg measurements: see Appendix A.

Male. Unknown.

##### Habitat.

Forest leaf litter.

##### Distribution.

Known only from the type locality (Fig. 83).

#### Nesticella yui

Taxon classificationAnimaliaAraneaeNesticidae

Wunderlich & Song, 1995

Figs 62 , 83

Nesticella yui Wunderlich & Song, 1995: 347, fig. 19 (♀).
Nesticella yui : Song et al. 1999: 86, fig. 37D (♀).
Nesticella yui : Grall and Jäger 2016: 250, figs 6–9, 24–25 (♂♀).

##### Type material examined.

Holotype ♀ and paratype 1♀ (IZCAS), CHINA: Yunnan Province, Mengla County, Xishuangbanna Nature Reserve, Menglun Tropical Botanic Garden, near a rain forest (21.91300°N, 101.26700°E), litter, 2.X.1987, L. Yu leg.

##### Other material.

2♂ (IZCAS), same locality as holotype, 12.VII.2015, S. Li leg.

##### Diagnosis.

**Figure 62.**
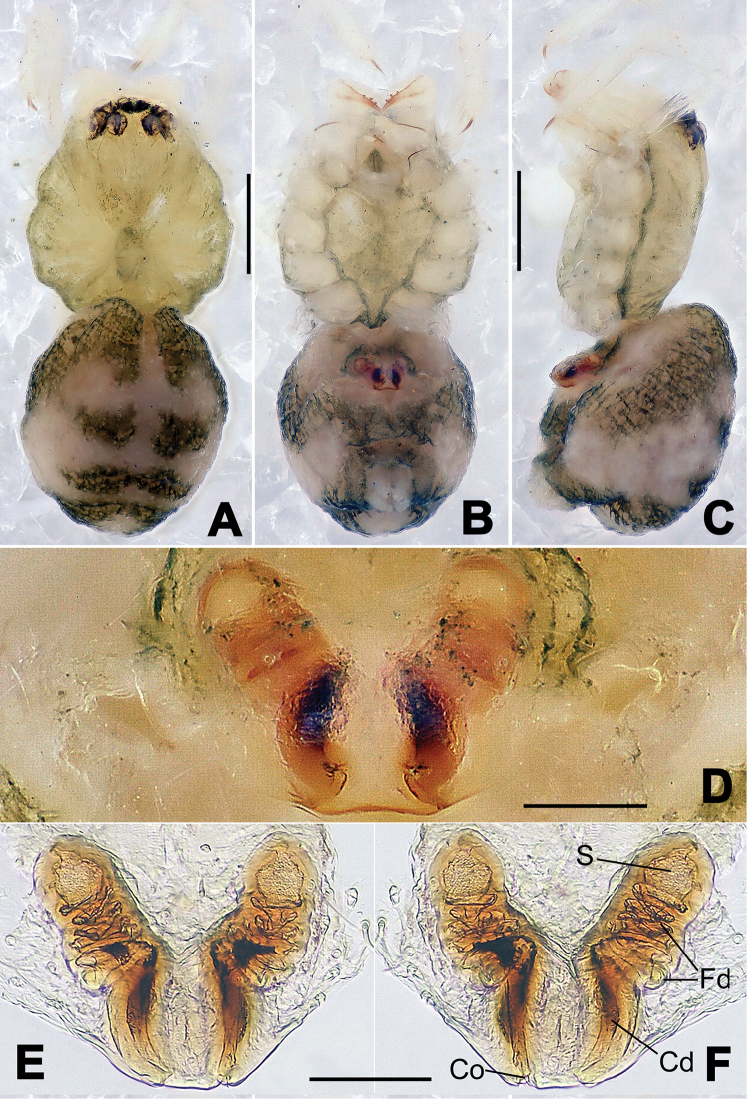
*Nesticella yui*, holotype (female). **A** Habitus, dorsal view **B** Ditto, ventral view **C** Ditto, lateral view **D** Epigyne, ventral view **E** Vulva, ventral view **F** Ditto, dorsal view. Scale bars: **A–C** = 0.50 mm; **D–F** = 0.10 mm.

Males of this species can be easily distinguished from those of the congeneric members by the single, massive distal process of the paracymbium (see Grall and Jäger 2016: fig. 7). Females are distinguished by the reduced scape (Sp) (Fig. 62D), the copulatory ducts (Cd) shaped like brackets and the very long, spiral fertilization ducts (Fd) with at least five coils (Fig. 62E–F). *Nesticella yui* is closely related to *Nesticella gongshanensis* sp. n. (see Fig. 52A–F) from which it can be separated by the wider scape (Sp), the larger distance between the copulatory openings (Co) and the generally different shape of the copulatory ducts (Cd) (Fig. 62D–F vs. Fig. 52D–F).

##### Description.

See Grall and Jäger (2016).

##### Habitat.

Forest leaf litter.

##### Distribution.

China (Yunnan) (Fig. 83), Laos.

#### Nesticella zhiyuani sp. n.

Taxon classificationAnimaliaAraneaeNesticidae

http://zoobank.org/F786A79F-1E62-4DBD-A156-36EBAABBCB48

Figs 63 , 64 , 83

##### Type material.

Holotype ♂ and paratypes 1♂4♀ (IZCAS), INDONESIA: West Sumatra Province, Payakumbuh City, Koto Tiggi Village, a cave without name, close to the Imam Bonjol Cave (2.08057°S, 101.37412°E, 962 m), 17.V.2014, Z. Yao leg.

##### Etymology.

The new species is named after Dr. Zhiyuan Yao who extensively collected and studied spiders from Southeast Asia; noun (name) in genitive case.

##### Diagnosis.

**Figure 63.**
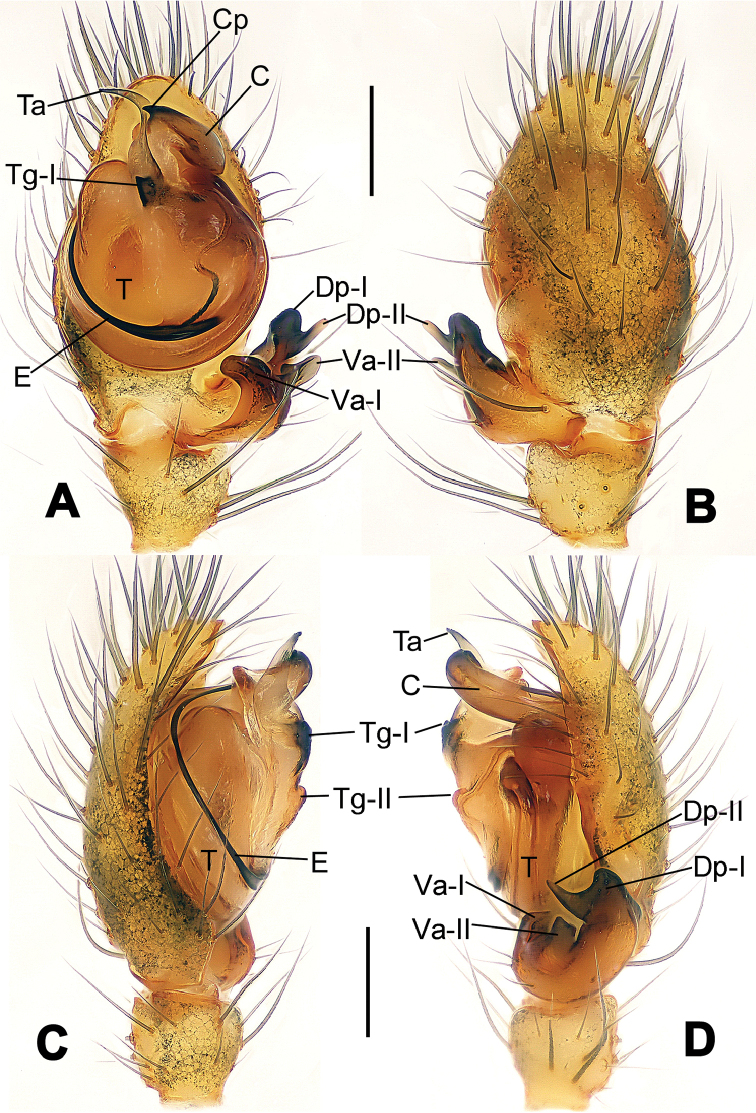
*Nesticella zhiyuani* sp. n., holotype (male). **A** Palp, ventral view **B** Ditto, dorsal view **C** Ditto, prolateral view **D** Ditto, retrolateral view. Scale bars: 0.10 mm.

**Figure 64.**
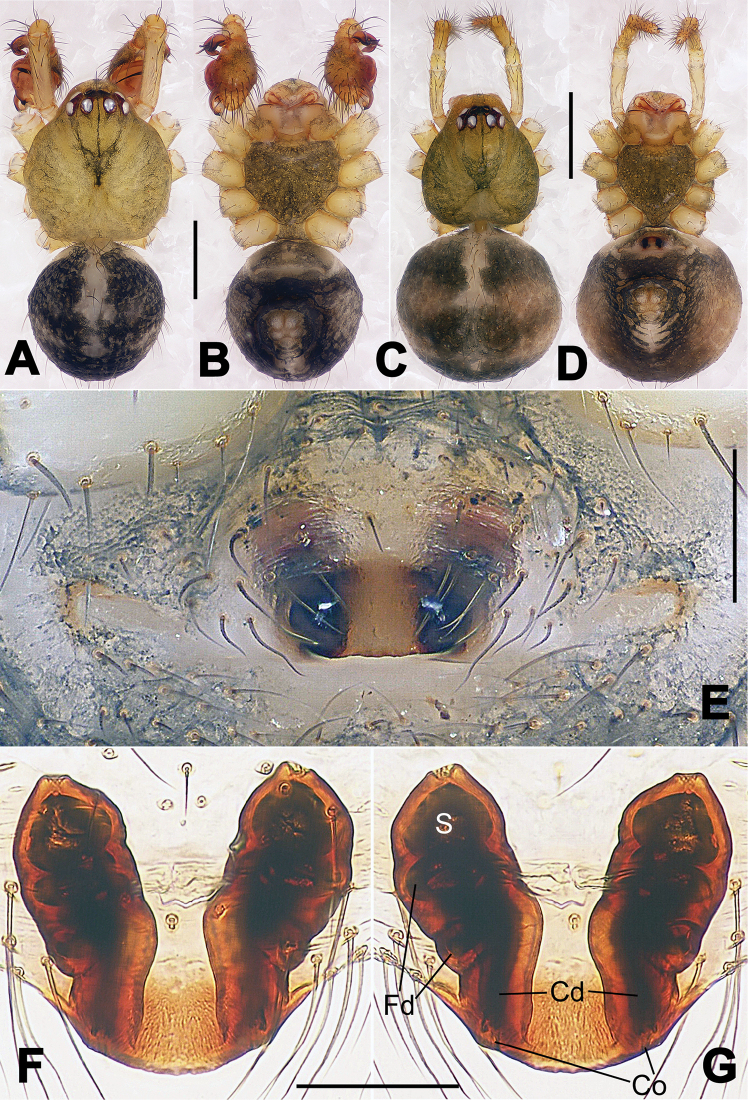
*Nesticella zhiyuani* sp. n., holotype (male) and paratype (female). **A** Male habitus, dorsal view **B** Ditto, ventral view **C** Female habitus, dorsal view **D** Ditto, ventral view **E** Epigyne, ventral view **F** Vulva, ventral view **G** Vulva, dorsal view. Scale bars: **A–D** = 0.50 mm; **E–G** = 0.10 mm.

Males of *Nesticella zhiyuani* sp. n. can be recognized from those of the other species belonging to the *nepalensis*-group by the long, sickle-like terminal apophysis (Ta) with a very sharp tip, by the reduced, blunt tegular apophysis (Tg-I) and by the almost round distal process I (Dp-I) and the elongate, sharp process II (Dp-II) (Fig. 63A–B, D). Females can be easily recognized by the strongly reduced, almost absent, scape (Sp), by the piriform spermathecae (S) and by the short copulatory ducts (Cd) (Fig. 64E–G) which are not shared with any other species of the group.

##### Description.

Habitus as in Fig. 64A–D. Carapace yellowish in males, darker in females; dark area present before the fovea. Cervical groove and fovea distinct. Thoracic area dark, mouthparts yellow. Legs and female palps yellowish, distally darker in metatarsi and tarsi, faintly dark in the other segments. Opisthosoma dark with a dorsal vertical pale stripe and a posterior irregular spot, covered with long setae.

Male palp (Fig. 63A–D): cymbium with dark pigmentation (Fig. 63B). Paracymbium with two differently oriented processes, Va-I elongate, bent inward and ending with an almost round tip, Va-II lobed and directed outward; bifurcated distal process with two branches Dp-I blunt and almost round, Dp-II elongate and very sharp (Fig. 63A, D). Terminal apophysis long, translucent, laminar in the middle and ending with a sharp, sickle-like tip (Fig. 63A). Tegular apophysis vestigial and blunt, Ta-II slightly protruding outward from the tegulum (Fig. 63A). Conductor ending with a short, horn-like process (Fig. 63A).

Epigyne (Fig. 64E–G): dark colored. Scape strongly reduced, very short and wide, with a faintly convex posterior margin (Fig. 64E). Copulatory openings located at the posterior margin (Fig. 64G). Spermathecae piriform, pointed on the dorsal side, separated by about 0.8 diameters (Fig. 64F). Fertilization ducts long, reaching the spermathecae with two coils (Fig. 64G). Copulatory ducts thick and short (Fig. 64F–G).

Male (holotype). Total length 2.13. Carapace 1.15 long, 1.05 wide. Opisthosoma 1.05 long, 0.95 wide. Clypeus height 0.22. Sternum 0.65 long, 0.68 wide. Leg measurements: see Appendix A.

Female (one of the paratypes). Total length 2.50. Carapace 1.14 long, 0.99 wide. Opisthosoma 1.30 long, 1.35 wide. Clypeus height 0.20. Sternum 0.66 long, 0.66 wide. Leg measurements: see Appendix A.

##### Habitat.

Cave.

##### Distribution.

Known only from the type locality (Fig. 83).

### *Nesticella phami*-group

**Group features.** Males of the species belonging to this group can be distinguished by having a short, flat and unbranched ventral apophysis (Va), sometimes strongly reduced, by a flat, an elongate or a squared distal process of the paracymbium (Dp), a well-developed tegular apophysis (Tg) and a hook-shaped process of the conductor (Cp). Females are easily recognized by the short fertilization and copulatory ducts (Fd and Cd) and the wide, ovoid spermathecae (S).

**Composition.** *Nesticella phami* sp. n. and *Nesticella sumatrana* sp. n.

#### Nesticella phami sp. n.

Taxon classificationAnimaliaAraneaeNesticidae

http://zoobank.org/67D16289-3C70-4252-A439-69098B767AEE

Figs 65 , 66 , 83

##### Type material.

Holotype ♂ and paratypes 1♂3♀ (IZCAS), VIETNAM: Quang Ninh Province: Phong Nha Ke bang National Park, Sung Sot Cave (17.53932°N, 106.2549°E, 200 m), 5.VIII.2011, D.S. Pham leg.

##### Etymology.

The new species is named after Dr. Pham Dinh Sac, a pioneer spider researcher in Vietnam; noun (name) in genitive case.

##### Diagnosis.

**Figure 65.**
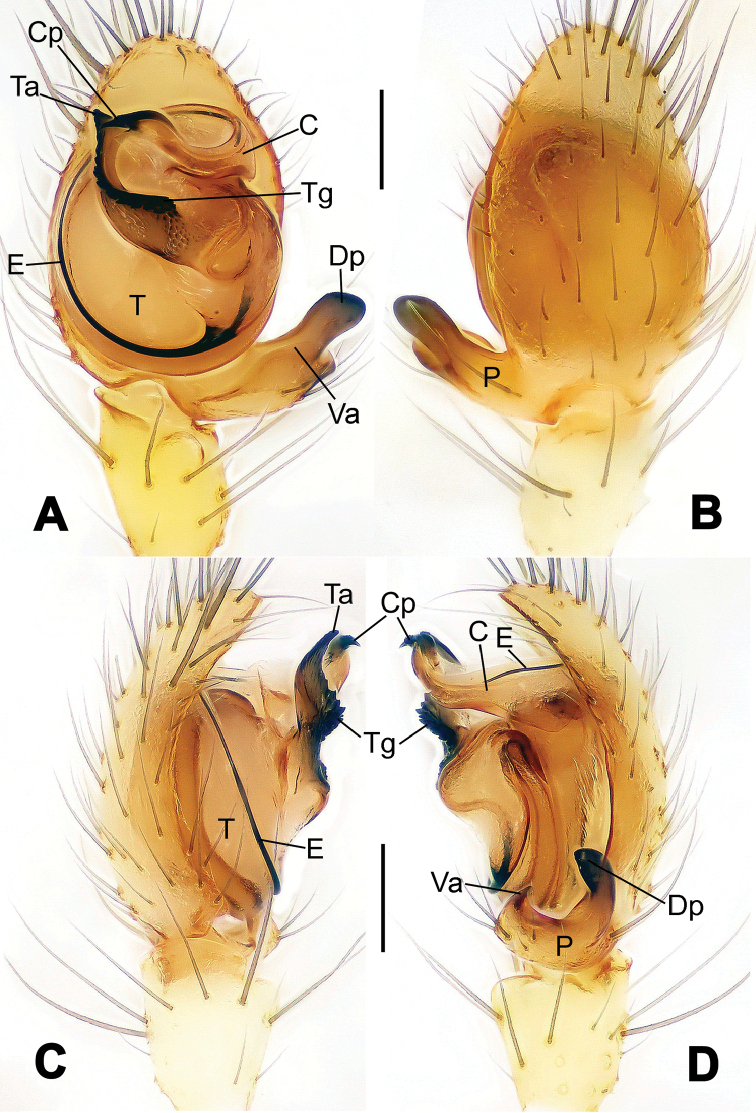
*Nesticella phami* sp. n., holotype (male). **A** Palp, ventral view **B** Ditto, dorsal view **C** Ditto, prolateral view **D** Ditto, retrolateral view. Scale bars: 0.10 mm.

Males of the new species can be distinguished from those of *Nesticella sumatrana* sp. n. (Fig. 67A–G) by the strongly sclerotized terminal apophysis (Ta), the clearly serrated tegular apophysis (Tg) (Fig. 65A, C vs. Fig. 67A, C), the longer, and wider distal process of the paracymbium (Dp) (Fig. 65A–B, D vs. Fig. 67A–B, D) and the reduced ventral apophysis (Va) (Fig. 65A vs. Fig. 67A). Females can be easily separated from those of the other *Nesticella* species by the very short and wide scape (Sp), the short fertilization and copulatory ducts (Fd and Cd) and the wide, ovoid spermathecae (S).

**Figure 66.**
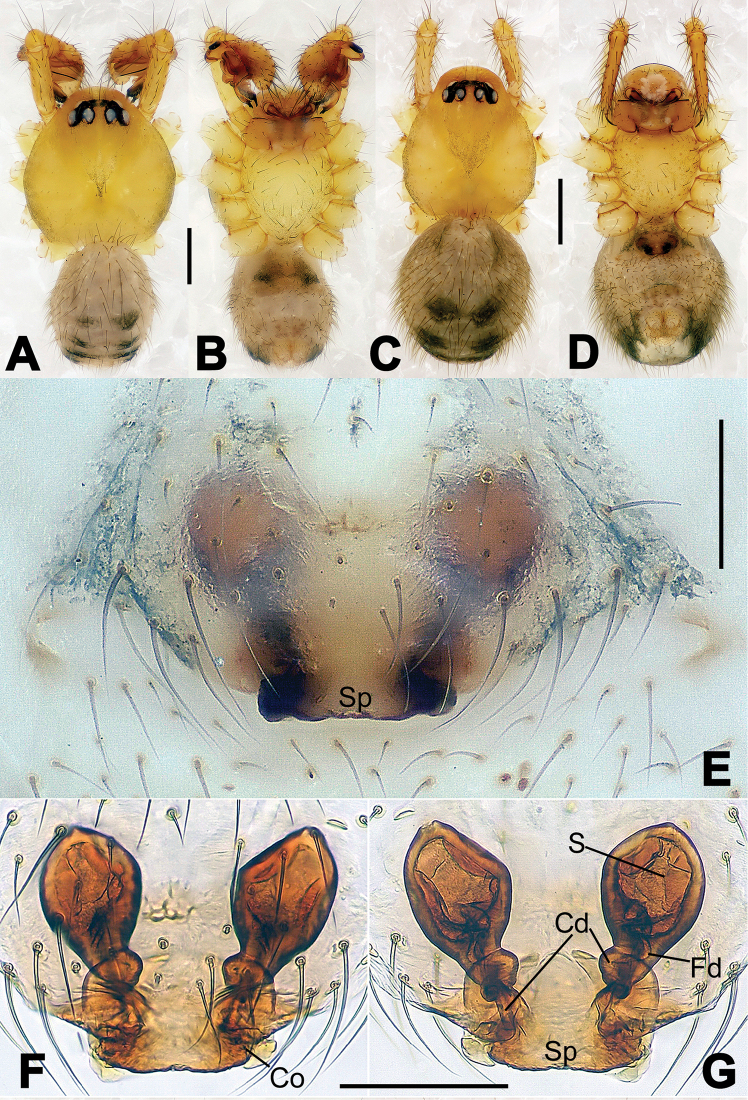
*Nesticella phami* sp. n., holotype (male) and paratype (female). **A** Male habitus, dorsal view **B** Ditto, ventral view **C** Female habitus, dorsal view **D** Ditto, ventral view **E** Epigyne, ventral view **F** Vulva, ventral view **G** Vulva, dorsal view. Scale bars: **A–D** = 0.50 mm; **E–G** = 0.10 mm.

**Figure 67.**
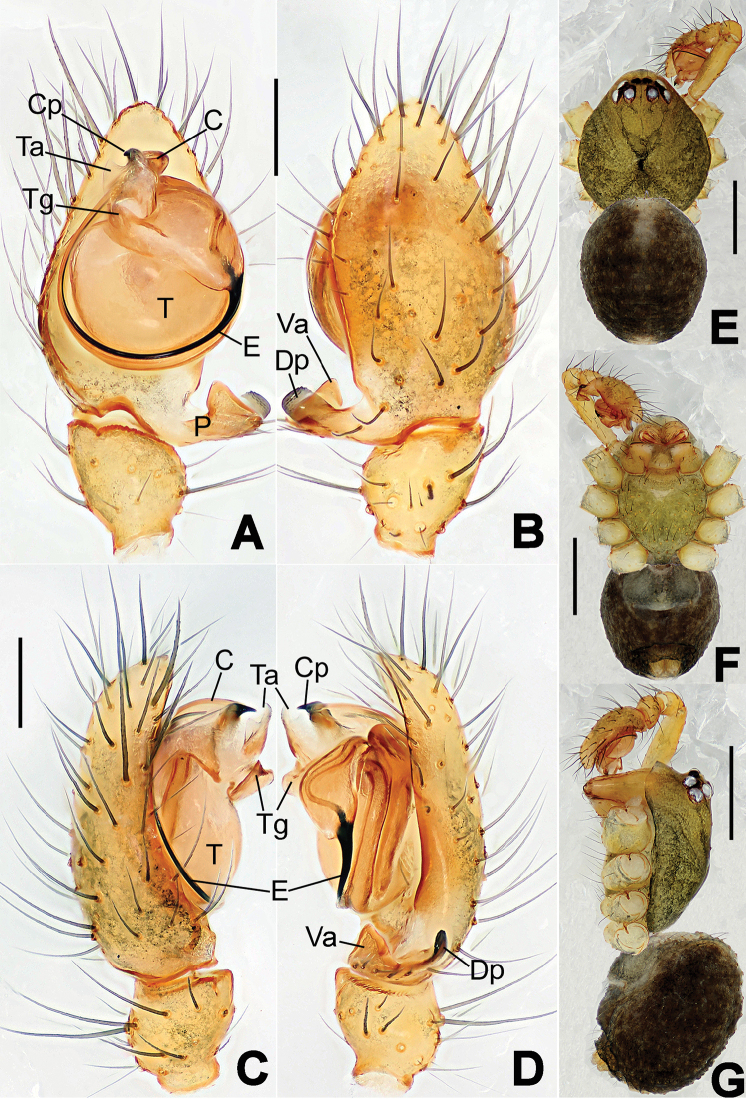
*Nesticella sumatrana* sp. n., holotype (male). **A** Palp, ventral view **B** Ditto, dorsal view **C** Ditto, prolateral view **D** Ditto, retrolateral view **E** Habitus, dorsal view **F** Ditto, ventral view **G** Ditto, lateral view. Scale bars: **A–D** = 0.10 mm; **E–G** = 0.50 mm.

##### Description.

Habitus as in Fig. 66A–D. Carapace uniformly yellow and faintly pigmented at center and at margins. Cervical groove faint, fovea shallow. Mouthparts brown-yellowish. Sternum with sparse long setae and shorter setae along margins. Legs and female palps yellowish, distally brownish in metatarsi and tarsi. Opisthosoma pale in males, darker in females, with paired black marks on the dorsal part, partially fused each other in the posterior side.

Male palp (Fig. 65A–D): paracymbium with a single, ventral apophysis strongly reduced, flat and short; distal process strongly sclerotized, elongate and flattened, with a blunt end (Fig. 65A–B, D). Terminal apophysis long, pointed, wrinkled, and sclerotized (Fig. 65A, C). Tegular apophysis strongly sclerotized, with a serrated margin and a granulate base (Fig. 65A, 65C–D). Conductor with a hook-like, sclerotized apical process (Fig. 65A, C–D).

Epigyne (Fig. 66E–G): faintly dark (Fig. 66E). Scape very short and wide, laterally sclerotized (Fig. 66E). Copulatory openings tiny, located below the lateral corners of the scape (Fig. 66F). Spermathecae wide and ovoid (slightly wilted after being treated with lactic acid) (Fig. 66E–G), separated by about 1 diameter. Fertilization ducts thin, coiling only once before reaching the spermathecae (Fig. 66G). Copulatory ducts thick, basally broad, and distally thinner, twisted in the middle (Fig. 66G).

Male (holotype). Total length 1.72. Carapace 1.00 long, 0.93 wide. Opisthosoma 0.83 long, 0.63 wide. Clypeus height 0.19. Sternum 0.60 long, 0.57 wide. Leg measurements: see Appendix A.

Female (one of the paratypes). Total length 2.28. Carapace 1.10 long, 0.98 wide. Opisthosoma 1.25 long, 0.96 wide. Clypeus height 0.20. Sternum 0.68 long, 0.67 wide. Leg measurements: see Appendix A.

##### Habitat.

Cave.

##### Distribution.

Known only from the type locality (Fig. 83).

#### Nesticella sumatrana sp. n.

Taxon classificationAnimaliaAraneaeNesticidae

http://zoobank.org/EBC2EB39-FADA-4265-BBEF-A5584D2DC848

Figs 67 , 83

##### Type material.

Holotype ♂ (IZCAS), INDONESIA: West Sumatra Province, Payakumbuh City, Koto Tiggi Village, a cave without name, close to the Imam Bonjol Cave (00.06368°S, 100.34513°E, 962 m), 17.V.2014, Z. Yao leg.

##### Etymology.

The specific name is derived from the type locality; adjective.

##### Diagnosis.

*Nesticella sumatrana* sp. n. can be distinguished from *Nesticella phami* sp. n. (Figs 65A–D, 66A–B) by the lobed ventral apophysis of the paracymbium (Va), strongly reduced in the latter species (Fig. 67A vs. Fig. 65A), by the shorter distal process of the paracymbium (Dp) (Fig. 67B vs. Fig. 65B), by the smaller tegular apophysis (Tg) lacking a serrated boarder and by the membranous, translucent terminal apophysis (Ta) (Fig. 67A vs. Fig. 65A). Furthermore, the two species can be distinguished by the darker body color of *Nesticella sumatrana* sp. n. (Fig. 67E–G vs. Fig. 66A–B).

##### Description.

Habitus as in Fig. 67E–G. Carapace very dark. Cervical groove distinct, fovea deep and black. Thoracic area dark. Mouthparts brown-yellowish. Sternum greyish, with sparse long setae. Legs yellowish, distally darker in each segment. Opisthosoma uniformly black, with a yellowish lanceolate mark on the dorsal side.

Male palp (Fig. 67A–D): paracymbium relatively simple. Ventral apophysis lobed, distal process triangular (Fig. 67A, D), short, flat and squared, apically wrinkled (Fig. 67A–B, D). Terminal apophysis membranous, triangular and laminar, located near the process of the conductor (Fig. 67A, D). Tegular apophysis laminar, weakly sclerotized and protruding outward (Fig. 67A, 67C). Conductor ending with a small, hook-shaped, sclerotized process (Fig. 67A, C–D).

Male (holotype). Total length 1.81. Carapace 0.92 long, 0.85 wide. Opisthosoma 0.96 long, 0.86 wide. Clypeus height 0.17. Sternum 0.56 long, 0.57 wide. Leg measurements: see Appendix A.

Female. Unknown.

##### Habitat.

Cave.

##### Distribution.

Known only from the type locality (Fig. 83).

### *Nesticella quelpartensis*-group

**Group features.** Males belonging to this species-group can be recognized by having a paracymbium with a very wide, laminar distal process (Dp), a short ventral apophysis (Va), a protruding, crest-like terminal apophysis (Ta), a missing tegular apophysis (Tg) and a wide, flat process of the conductor (Cp). Females are distinguished by the presence of a protruding scape with a rounded apex and by the straight copulatory ducts (Cd), strongly diverging to each other and forming a V visible through the transparent tegument of the epigyne.

**Composition.** *Nesticella kaohsiungensis* sp. n. and *Nesticella quelpartensis* (Paik & Namkung, 1969).

#### Nesticella kaohsiungensis sp. n.

Taxon classificationAnimaliaAraneaeNesticidae

http://zoobank.org/3687CC4F-E5B5-4EFA-B8C0-94AA815BBDC9

Figs 68 , 69 , 83

Nesticella taiwan Tso & Yoshida, 2000: 13, figs 7–11 (only ♀, mismatched with the holotype ♂).

##### Type material.

Holotype ♂ (IZCAS) and paratypes 4♀ (IZCAS), CHINA: Taiwan, Kaohsiung City, Gushan District, Shoushan Mountain, a cave without name (22.64814°N, 120.26240°E, 323 m), 29.VI.2013, S. Li leg.

##### Etymology.

The specific name is derived from the type locality; adjective.

##### Diagnosis.

**Figure 68.**
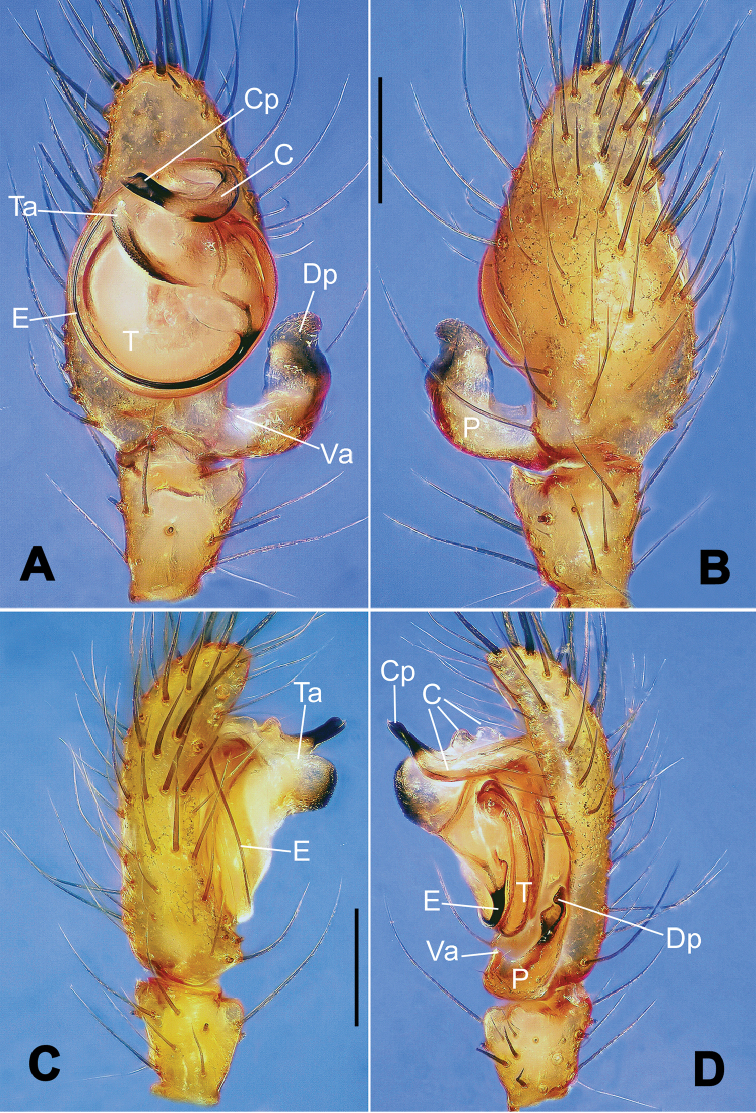
*Nesticella kaohsiungensis* sp. n., holotype (male). **A** Palp, ventral view **B** Ditto, dorsal view **C** Ditto, prolateral view **D** Ditto, retrolateral view. Scale bars: 0.10 mm.

**Figure 69.**
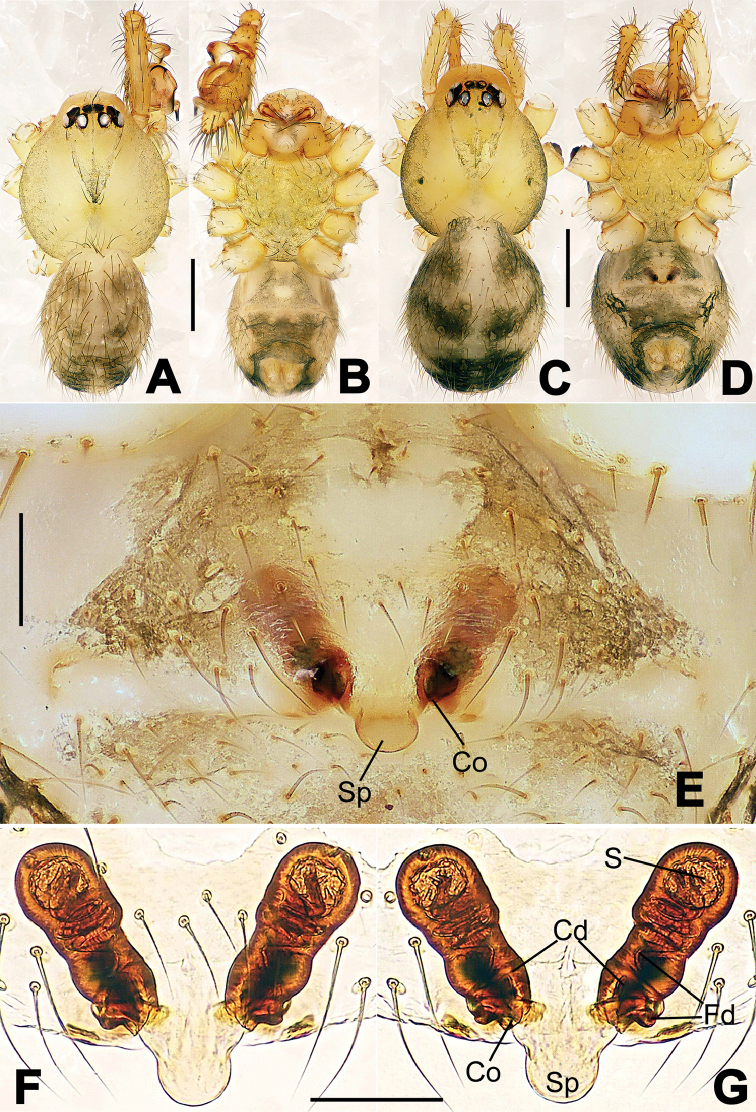
*Nesticella kaohsiungensis* sp. n., holotype (male) and paratype (female). **A** Male habitus, dorsal view **B** Ditto, ventral view **C** Female habitus, dorsal view **D** Ditto, ventral view **E** Epigyne, ventral view **F** Vulva, ventral view **G** Vulva, dorsal view. Scale bars: **A–D** = 0.50 mm; **E–G** = 0.10 mm.

Males can be distinguished from those of *Nesticella quelpartensis* (see Paik & Namkung, in Paik et al. 1969: 812, figs 37–42) by the shorter ventral apophysis of the paracymbium (Va) and the thicker and blunter process of the conductor (Cp) (Fig. 68A, C vs. figs 40–41). Females are distinguished by the narrower, longer scape (Fig. 69F–G vs. figs 37–38).

##### Description.

Habitus as Fig. 69A–D. Carapace pale yellow in males, darker in females, faint dark at margins and near the center. Cervical groove and fovea distinct. Mouthparts brown-yellowish. Sternum yellow, with sparse long setae. Legs and female palps yellowish, distally darker in metatarsi and tarsi. Opisthosoma light yellow with paired dark marks, partially merged with each other on the posterior, forming a light, cross-shaped mark on the background. Pattern fainter in males.

Male palp (Fig. 68A–D): paracymbium with a laminar, pointed ventral apophysis and a wide, laminar, finely textured distal process (Fig. 68A–B, 68D). Terminal apophysis crest-like, wide and protruding outward from the bulb with a finely granulate surface (Fig. 68A, C). Conductor with a long, well-developed distal process, apically flat and strongly sclerotized (Fig. 68A, C–D).

Epigyne (Fig. 69E–G): wrinkled and translucent. Scape long and protruding beyond the epigynal posterior margin (Fig. 69E–G), basally wider and ending with an almost round lobe, about as wide as the diameter of a spermatheca. Vulva forming a rough “V” visible trough the tegument of the epigyne (Fig. 69F). Copulatory openings tiny (Fig. 69E, 69G). Spermathecae small and globular, separated by at least 1.6 diameters (Fig. 69F–E). Fertilization ducts thin and long, dorsally oriented, reaching the spermathecae with at least three coils (Fig. 69G). Copulatory ducts thick and short, basally narrower (Fig. 69F–G).

Male (holotype). Total length 1.88. Carapace 1.06 long, 0.92 wide. Opisthosoma 0.94 long, 0.68 wide. Clypeus height 0.18. Sternum 0.64 long, 0.61 wide. Leg measurements: see Appendix A.

Female (one of the paratypes). Total length 2.29. Carapace 1.20 long, 1.00 wide. Opisthosoma 1.30 long, 0.93 wide. Clypeus height 0.21. Sternum 0.71 long, 0.67 wide. Leg measurements: see Appendix A.

##### Habitat.

Cave, forest leaf litter.

##### Distribution.

China (Taiwan) (Fig. 83)

##### Remarks.

A female paratype of *Nesticella taiwan* Tso & Yoshida, 2000 was mismatched with the holotype male, synonymized here with *Nesticella odonta*. A detailed morphological comparison of the samples, together with the an in-progress molecular analysis of newly collected specimens from the type locality of *Nesticella taiwan*, allow the correct matching of females and males and demonstrate that they belong to a new species.

#### Nesticella quelpartensis

Taxon classificationAnimaliaAraneaeNesticidae

(Paik & Namkung, 1969)

Nesticus quelpartensis Paik & Namkung, in Paik et al., 1969: 812, figs 37–42 (♂♀).
Nesticus quelpartensis : Paik 1978: 234, figs 99.1–6 (♂♀).
Howaia quelpartensis : Lehtinen and Saaristo 1980: 54 (♂♀) (transfer from Nesticus).
Nesticella quelpartensis : Namkung 2002: 79, figs 12.3a–b (♂♀).
Nesticella quelpartensis : Namkung 2003: 81, figs 12.3a–b (♂♀).

##### Diagnosis.

Males can be separated from those of *Nesticella kaohsiungensis* sp. n. by the wider ventral apophysis of the paracymbium (Va) and the slimmer and sharper process of the conductor (Cp) (see Paik et al. 1969: figs 40–41 vs. Fig. 68A, C). Females of *Nesticella quelpartensis* can be recognized by the wider and shorter scape (see Paik et al. 1969: figs 37–38 vs. Fig. 69F–G).

##### Description.

See Paik et al. (1969).

##### Habitat.

Cave.

##### Distribution.

Korea.

#### Genus Pseudonesticus

Taxon classificationAnimaliaAraneaeNesticidae

Liu & Li, 2013

Pseudonesticus Liu & Li, 2013a: 790.

##### Type species.

*Pseudonesticus clavatus* Liu & Li, 2013 from Yunnan, China.

##### Diagnosis.

Males belonging to *Pseudonesticus* can be distinguished from those of *Cyclocarcina* Komatsu, 1942 by the hooked or finger-like terminal apophysis (Ta), always well-developed (shorter and simpler in *Cyclocarcina*), the longer embolus, the flat tegular apophysis (Tg) and the less ramified paracymbium, rather than having several apophyses. It can be separated from the other Nesticini by the slimmer or hook shaped terminal apophysis (Ta) and by the usually very long embolus (E) which are thicker and shorter in the other genera. Females can be distinguished from those belonging to the other Nesticini by the shape of the fertilization and copulatory ducts, coiled before reaching the spermathecae, and by the wide or triangular scape which is not shared with any other genus.

##### Description.

Total length: 1.76–2.72 (male), 1.82–3.24 (female). Carapace almost round in males, ovate in females, usually uniformly yellow. Legs of the same color. Eyes generally reduced or absent. When present, AER and PER straight, MOA trapezoidal, narrower in the front. Eight well-developed eyes and a clear pattern only in *Pseudonesticus dafangensis* sp. n. Cervical groove and fovea usually indistinct. Chelicera with three promarginal teeth and multiple retromarginal tiny denticles on the fang furrow. Opisthosoma with long setae, yellowish or greyish, rarely heavily pigmented.

Male palp: tibia short, wider than long, basally narrower, with three retrolateral trichobothria and several long setae. Paracymbium well-developed with a laminar distal process, pointed or lobe-shaped, and generally lacking a ventral apophysis, which is always simple when present (e.g. *Pseudonesticus clavatus*). Some species with a long and flat dorsal apophysis. Bulb with a well-developed terminal apophysis, hooked or finger-like. Tegular apophysis flat, *Pseudonesticus spinosus* sp. n. and *Pseudonesticus ziyunensis* sp. n. with a second tegular apophysis (Tg-II). Conductor wide and laminar, with one or two processes, sometimes with a long apex (e.g. *Pseudonesticus miao* sp. n., *Pseudonesticus ziyunensis* sp. n.). Embolus usually thin and long, with the terminal part strongly coiled, reduced only in *Pseudonesticus clavatus*.

Epigyne: scape always present, wide and lobed or triangularly-shaped. Copulatory openings located under the scape. Spermathecae slightly visible through the tegument. Spermathecae small and globular separated by at least two diameters. Fertilization and copulatory ducts ventrally oriented, thin and coiled with two to three loops. Vulval pockets well developed, located near the spermathecae.

##### Composition.

*Pseudonesticus clavatus* Liu & Li, 2013, *Pseudonesticus dafangensis* sp. n., *Pseudonesticus miao* sp. n., *Pseudonesticus spinosus* sp. n., *Pseudonesticus wumengensis* sp. n., and *Pseudonesticus ziyunensis* sp. n.

##### Distribution.

China (Guizhou, Yunnan).

#### Pseudonesticus clavatus

Taxon classificationAnimaliaAraneaeNesticidae

Liu & Li, 2013

Pseudonesticus clavatus Liu & Li, 2013a: 790, figs 1–14 (♂♀).

##### Diagnosis.

Males can be recognized from those of other *Pseudonesticus* species by the wider distal process (Dp) of the paracymbium, the big and thick process of the conductor (Cp), the wide and well-developed ventral apophysis of the paracymbium (Va) and the thick embolus which are respectively, narrower, smaller and thinner, less developed or absent and slimmer in the other species (see Liu and Li 2013a: figs 1–2, 7–8). Females of this species can be distinguished by the wider, piriform spermathecae rather than small and almost round spermathecae, by the U-shaped scape (Sp) and by the smaller vulval pockets (Vp) (respectively slimmer, triangular or U-shaped but with a larger proximal part and wider in the other species) (see Liu and Li 2013a: figs 4–5, 12–14).

##### Description.

See Liu and Li (2013a).

##### Distribution.

China (Yunnan).

#### Pseudonesticus dafangensis sp. n.

Taxon classificationAnimaliaAraneaeNesticidae

http://zoobank.org/81670B92-0180-4215-AD56-7E11B410D8CE

Figs 70 , 71 , 81

##### Type material.

Holotype ♂ and paratypes 8♂14♀ (IZCAS), CHINA: Guizhou Province, Dafang County, Sanhe Village, Yelaoda Cave (27.18167°N, 105.47130°E, 1438 m), 3.V.2007, J. Liu & Y. Lin leg.

##### Etymology.

The specific name is derived from the type locality; adjective.

##### Diagnosis.

**Figure 70.**
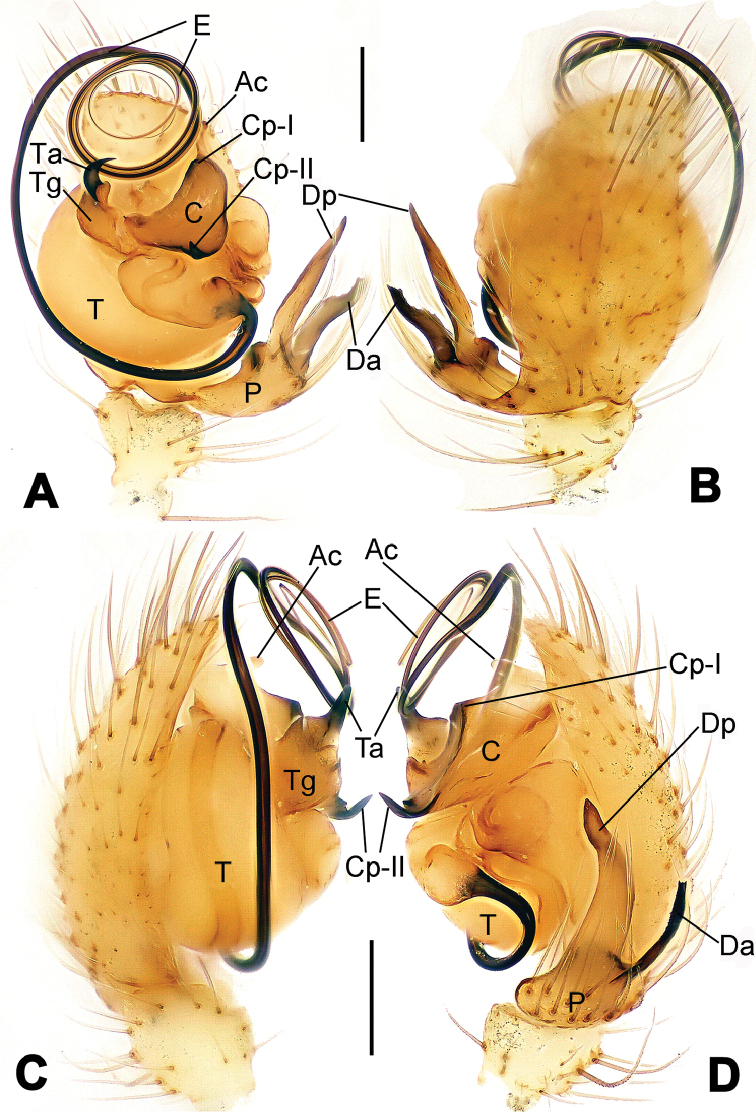
*Pseudonesticus dafangensis* sp. n., holotype (male). **A** Palp, ventral view **B** Ditto, dorsal view **C** Ditto, prolateral view **D** Ditto, retrolateral view. Scale bars: 0.10 mm.

**Figure 71.**
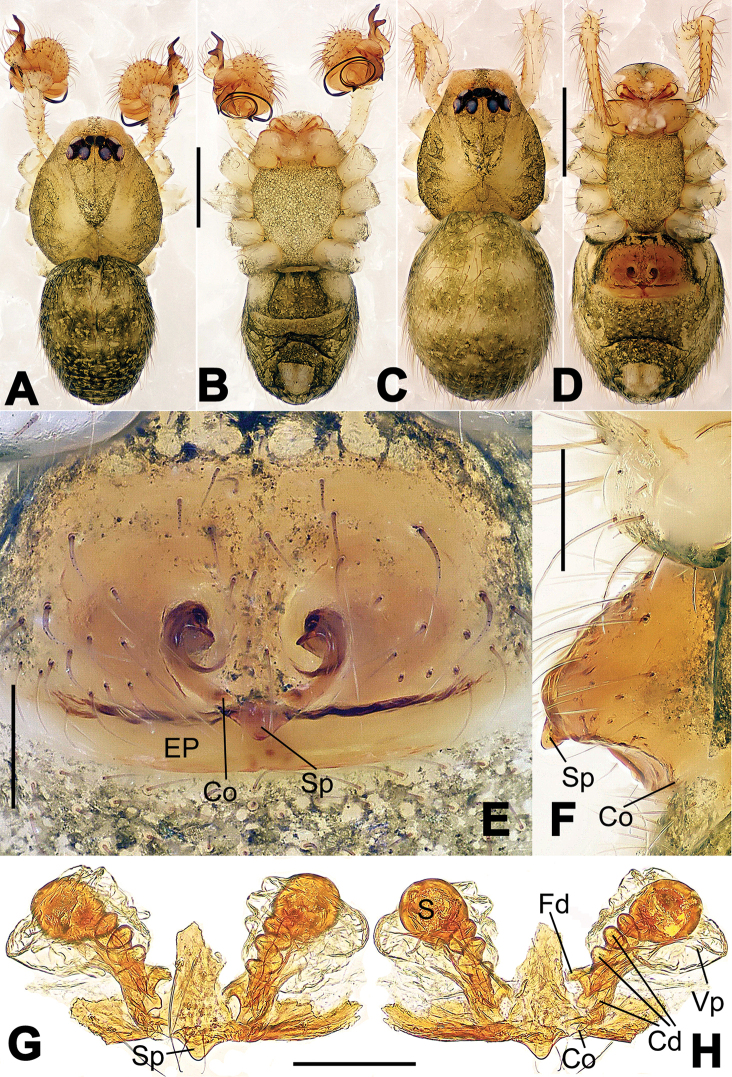
*Pseudonesticus dafangensis* sp. n., holotype (male) and paratype (female). **A** Male habitus, dorsal view **B** Ditto, ventral view **C** Female habitus, dorsal view **D** Ditto, ventral view **E** Epigyne, ventral view **F** Ditto, lateral view **G** Vulva, ventral view **H** Vulva, dorsal view. Scale bars: **A–D** = 0.50 mm; **E–H** = 0.10 mm.

The new species is closely related to *Pseudonesticus spinosus* sp. n. (Figs 74A–D, 75E–H) and *Pseudonesticus wumengensis* sp. n. (Fig. 76D–G). Males of this species can be distinguished from those of *Pseudonesticus spinosus* sp. n. by the longer dorsal apophysis (Da) (Fig. 70A–B, D vs. Fig. 74A–B, D), the compact distal process (Dp) lacking any serrated margin (Fig. 70D vs. Fig. 74D), the absence of a clear additional tegular apophysis (Tg-II) (Fig. 70A vs. Fig. 74A), the sharper and longer terminal apophysis (Ta) (Fig. 70A vs. Fig. 74A) and the wider conductor with two clear processes (Cp-I and Cp-II) (Fig. 70C–D vs. Fig. 74C–D). Females can be distinguished from those of *Pseudonesticus spinosus* sp. n. by the smaller, triangular scape (Sp), the ducts (Fd and Cd) closer to each other and the darker coloration (Fig. 71E, H vs. Fig. 75E, H); from those of *Pseudonesticus wumengensis* sp. n. by the narrower epigynal plate (EP) (Fig. 71E–F vs. Fig. 76D–E) and the shorter and more triangular scape (Sp) (Fig. 71G–H vs. Fig. 76F–G). The narrow, triangular shape of the scape (Sp) with a sharp tip and the general configuration of the ducts (Fd and Cd), strongly coiled, allow an easy separation of *Pseudonesticus dafangensis* sp. n. from all other species in this genus.

##### Description.

Habitus as in Fig. 71A–D. Carapace pale yellow, with dark marks at margins and around cephalic area. Eyes ALE>PLE>PME>AME. Cervical groove and fovea distinct. Thoracic area with faint radial furrows. Mouthparts pale yellow, darker in females. Sternum yellowish, lightly pigmented. Legs and female palps yellowish, distally darker in metatarsi and tarsi. Opisthosoma very dark, especially in males, with lighter horizontal strips.

Male palp (Fig. 70A–D): paracymbium complex, with very long and sharp distal process and dorsal apophysis (Fig. 70A–B, D). Sharp, hook-like terminal apophysis (Fig. 70A). Tegular apophysis flat and wide (Fig. 70A, 70C). Conductor concave in the middle, with two processes, Cp-I proximal, sclerotized and hooked, Cp-II apical and laminar (Fig. 70C–D). Embolus long, coiled with 3.5 loops in the apical side of the bulb (Fig. 70A, C–D).

Epigyne (Fig. 71E–H): broad, slightly ventrally protruded, weakly sclerotized (Fig. 71E–F). Scape small, triangular, with a sharp tip (Fig. 71E). Spermathecae small and globular, separated by at least two diameters (Fig. 71G). Fertilization and copulatory ducts thin, long and coiling into at least three loops (Fig. 71G–H). Vulval pockets wide, located near the spermathecae and the fertilization and copulatory ducts (Fig. 71G–H).

Male (holotype). Total length 1.76. Carapace 0.95 long, 0.81 wide. Opisthosoma 0.92 long, 0.71 wide. Clypeus height 0.20. Sternum 0.58 long, 0.55 wide. Leg measurements: see Appendix A.

Female (one of the paratypes). Total length 2.07. Carapace 1.01 long, 0.80 wide. Opisthosoma 1.23 long, 0.92 wide. Clypeus length 0.18. Sternum 0.62 long, 0.48 wide. Leg measurements: see Appendix A.

##### Habitat.

Cave.

##### Distribution.

Known only from the type locality (Fig. 81).

#### Pseudonesticus miao sp. n.

Taxon classificationAnimaliaAraneaeNesticidae

http://zoobank.org/0DC6CA75-B678-409E-BF70-BE1982ADF33F

Figs 72 , 73 , 81

##### Type material.

Holotype ♂ and paratypes 18♀ (IZCAS), CHINA: Guizhou Province, Anshun City, Xixiu District, Xiaguantun Village, Duofan Cave (26.24117°N, 106.00230°E, 1396 m), 25.II.2011, H. Chen & Z. Zha leg.

##### Etymology.

Named after the Miao people, an ethnic minority living in Guizhou Province; noun in apposition.

##### Diagnosis.

**Figure 72.**
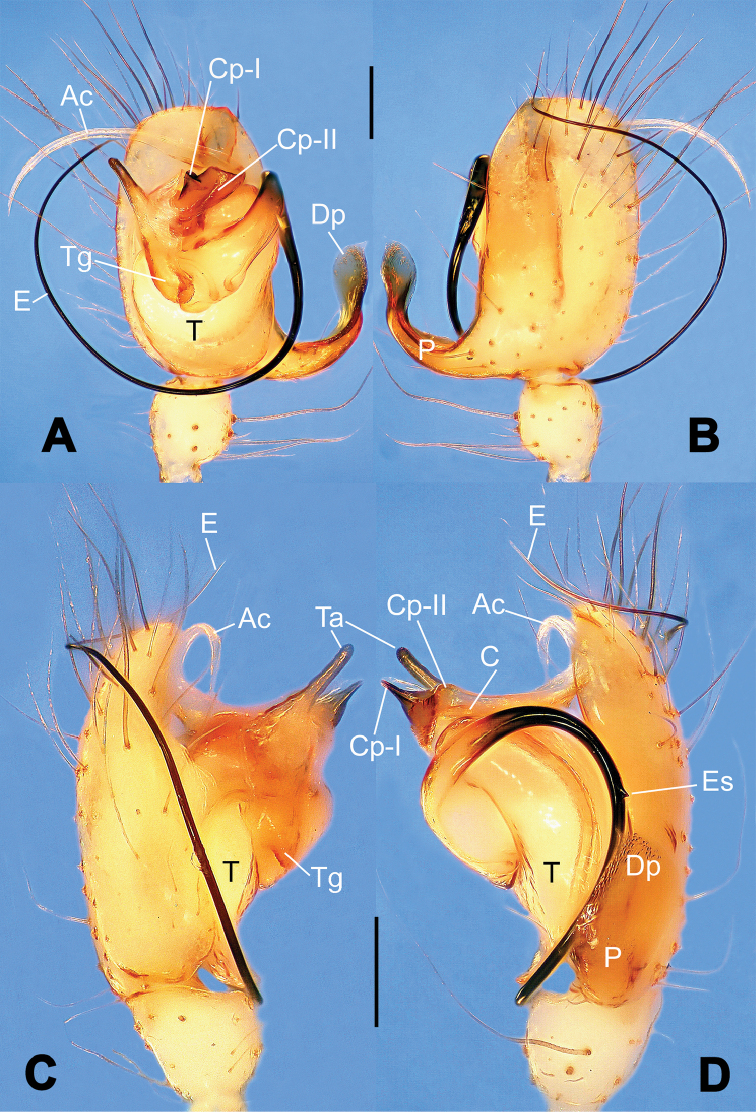
*Pseudonesticus miao* sp. n., holotype (male). **A** Palp, ventral view **B** Ditto, dorsal view **C** Ditto, prolateral view **D** Ditto, retrolateral view. Scale bars: 0.10 mm.

**Figure 73.**
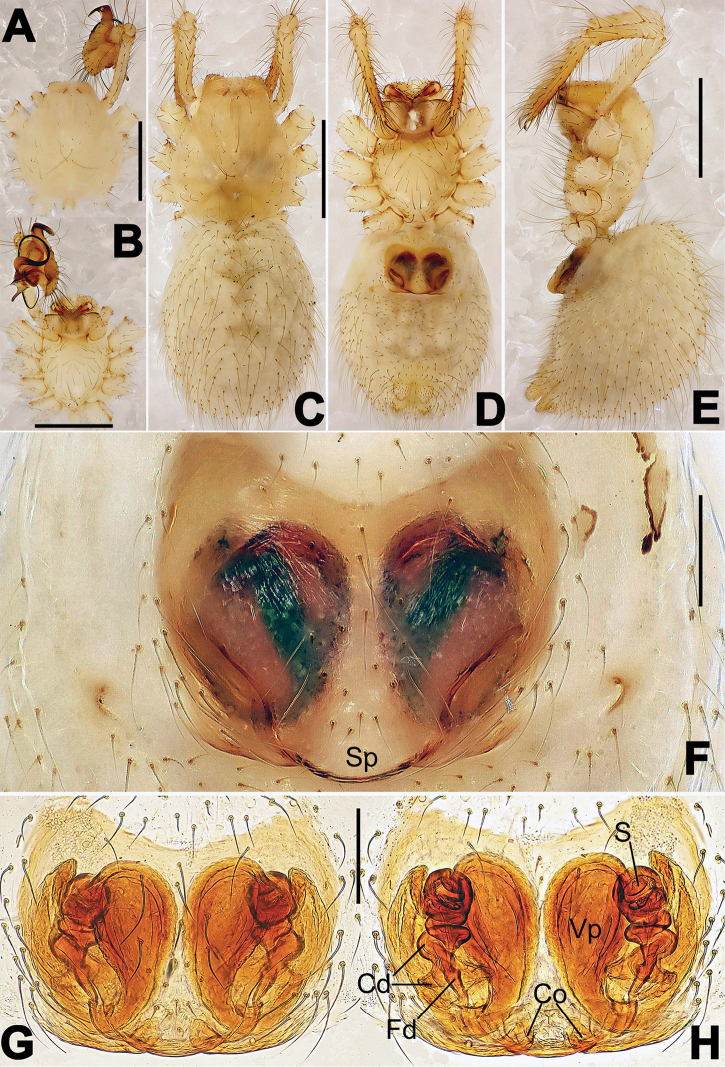
*Pseudonesticus miao* sp. n, holotype (male) and paratype (female). **A** Male prosoma, dorsal view **B** Ditto, ventral view **C** Female habitus, dorsal view **D** Ditto, ventral view **E** Ditto, lateral view **F** Epigyne, ventral view **G** Vulva, ventral view **H** Vulva, dorsal view. Scale bars: **A–E** = 0.50 mm; **F–H** = 0.10 mm.

The new species is closely related to *Pseudonesticus ziyunensis* sp. n. (Figs 77A–D, 78E–G) and *Pseudonesticus clavatus* (see Liu and Li 2013a: 790, figs 1–14). Males can be distinguished from those of *Pseudonesticus ziyunensis* sp. n. by the longer and straighter terminal apophysis (Ta), the shorter process I of the conductor (Cp-I) and the blunter distal process of the paracymbium (Dp) (Fig. 72A–B vs. Fig. 77A–B). Females are recognized by the narrower and longer scape (Sp) (Fig. 73E–F vs. Fig. 78E–F). Males of *Pseudonesticus miao* sp. n. can be also distinguished from those of *Pseudonesticus clavatus* by the lack of a ventral apophysis (Va) and the narrower distal process of the paracymbium (Dp) and the longer embolus (E) (Fig. 72A–B, D vs. figs 1–2, 7–9). Females can be separated from those of *Pseudonesticus clavatus* by the slimmer scape (Sp), the spiral fertilization and copulatory ducts (Fd and Cd), which are not coiled in the other species, and the wider vulval pockets (Vp) (Fig. 73F–H vs. figs 4–5, 12–14). The general shape of the paracymbium (P), the long terminal apophysis (Ta) and the wide scape (Sp) and, for the females, the broad lateral grooves of the epigyne, are all diagnostic characters which allow an easy separation from the other species of the genus *Pseudonesticus*.

##### Description.

Habitus as in Fig. 73A–E. Carapace pale yellow. Eyes absent. Cervical groove and fovea indistinct. Mouthparts pale yellow in males, light brown-yellowish in females. Sternum pale yellow. Legs and female palps yellowish, distally darker in each tarsus. Opisthosoma uniformly pale yellowish and with long setae.

Male palp (Fig. 72A–D): cymbium broad, nearly rectangular in dorsal view, with long setae, lateral cymbial furrow absent (Fig. 72B). Paracymbium with a lamellar, wide distal process, weakly sclerotized, ending with a thin, translucent sharp spur, weakly rugose on the margin (Fig. 72A–B, D). Dorsal and ventral apophysis absent. Terminal apophysis long, finger-like, tegular apophysis flat (Fig. 72A, C–D). Conductor sclerotized, with a long, flat and translucent apex, and two short processes, Cp-I ending with a round tip, Cp-II sharp (Fig. 72A, F). Embolus long and ending at the apex of the cymbium, tiny embolic spur present in the first part (Es) (Fig. 72A).

Epigyne (Fig. 73F–H): broad, with a weakly sclerotized ventral plate (Fig. 73F). Scape wide, with lateral grooves (Fig. 73F). Copulatory openings separated approximately by the spermathecal diameter (Fig. 73G–H). Spermathecae partially visible at the sides of the scape through the semi-transparent tegument. Spermathecae small, globular, separated by about three diameters (Fig. 73H). Fertilization and copulatory ducts thin, long and distally coiled into two loops (Fig. 73H). Vulval pockets broad, sac-shaped, close to each others (Fig. 73H).

Male (holotype). Total length 2.58. Carapace 1.13 long, 1.01 wide. Opisthosoma 1.56 long, 1.05 wide. Sternum 0.65 long, 0.65 wide. Leg measurements: see Appendix A.

Female (one of the paratypes). Total length 2.64. Carapace 1.18 long, 0.99 wide. Opisthosoma 1.60 long, 1.19 wide. Sternum 0.66 long, 0.69 wide. Leg measurements: see Appendix A.

##### Habitat.

Cave.

##### Distribution.

Known only from the type locality (Fig. 81).

#### Pseudonesticus spinosus sp. n.

Taxon classificationAnimaliaAraneaeNesticidae

http://zoobank.org/8434BECC-524B-493B-BE3B-E9AC2C321FEB

Figs 74 , 75 , 81

##### Type material.

Holotype ♂ and paratypes 4♀ (IZCAS), CHINA: Guizhou Province, Suiyang County, Guihua Village, Mahuang Cave (28.24365°N, 107.28908°E, 730 m), 13.V.2007, J. Liu & Y. Lin leg.

##### Etymology.

The specific name is derived from the Latin word “*spinosus*” = spiny, thorny, and it is related to the spiked shape of the distal process of the paracymbium in the male; adjective.

##### Diagnosis.

**Figure 74.**
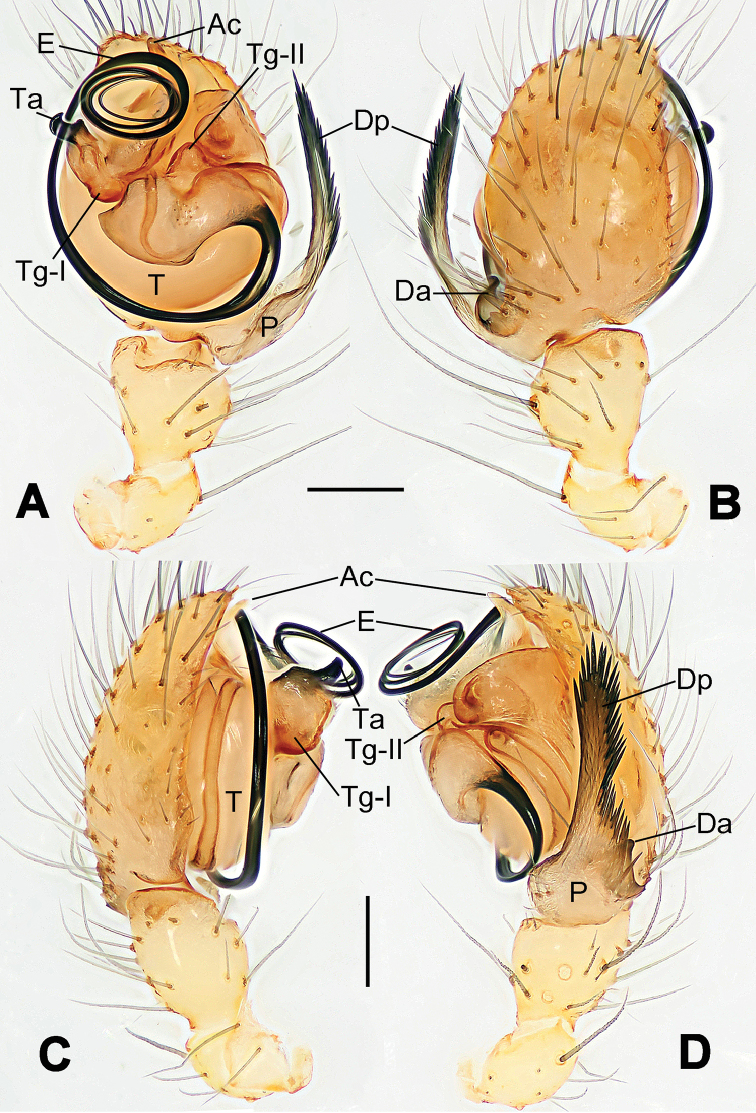
*Pseudonesticus spinosus* sp. n., holotype (male). **A** Palp, ventral view **B** Ditto, dorsal view **C** Ditto, prolateral view **D** Ditto, retrolateral view. Scale bars: 0.10 mm.

**Figure 75.**
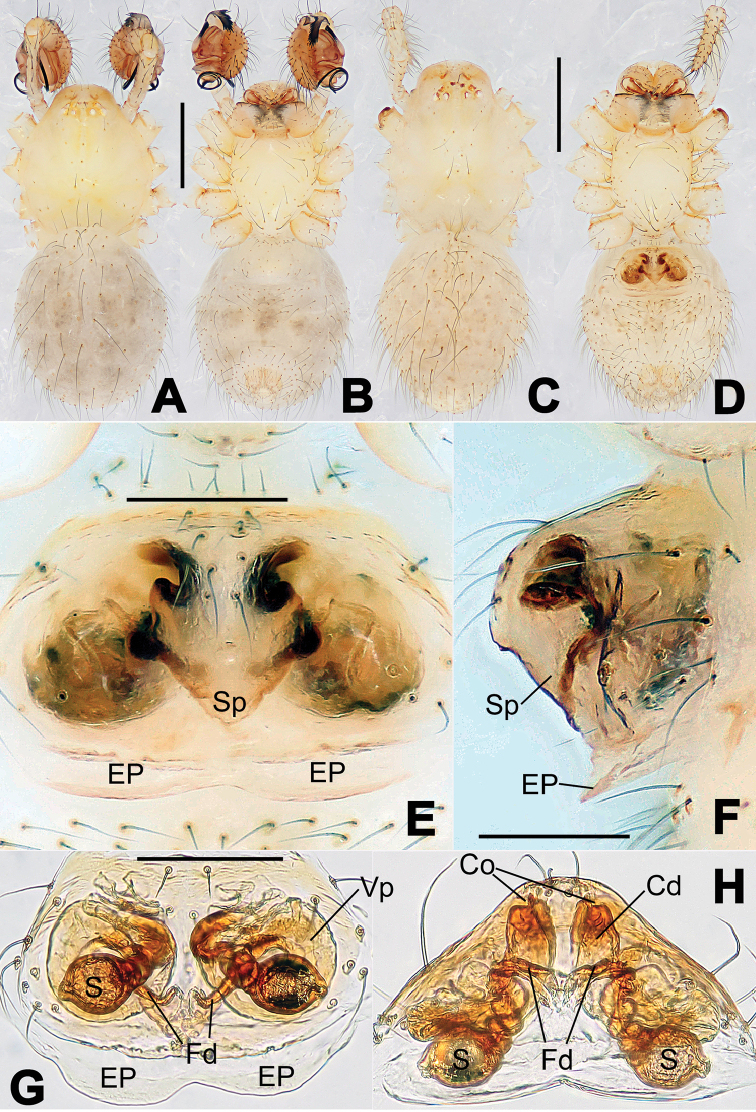
*Pseudonesticus spinosus* sp. n., holotype (male) and paratype (female). **A** Male habitus, dorsal view **B** Ditto, ventral view **C** Female habitus, dorsal view **D** Ditto, ventral view **E** Epigyne, ventral view **F** Ditto, lateral view **G** Vulva, dorsal view **H** Vulva, posterior view. Scale bars: **A–D** = 0.50 mm; **E–H** = 0.10 mm.

**Figure 76.**
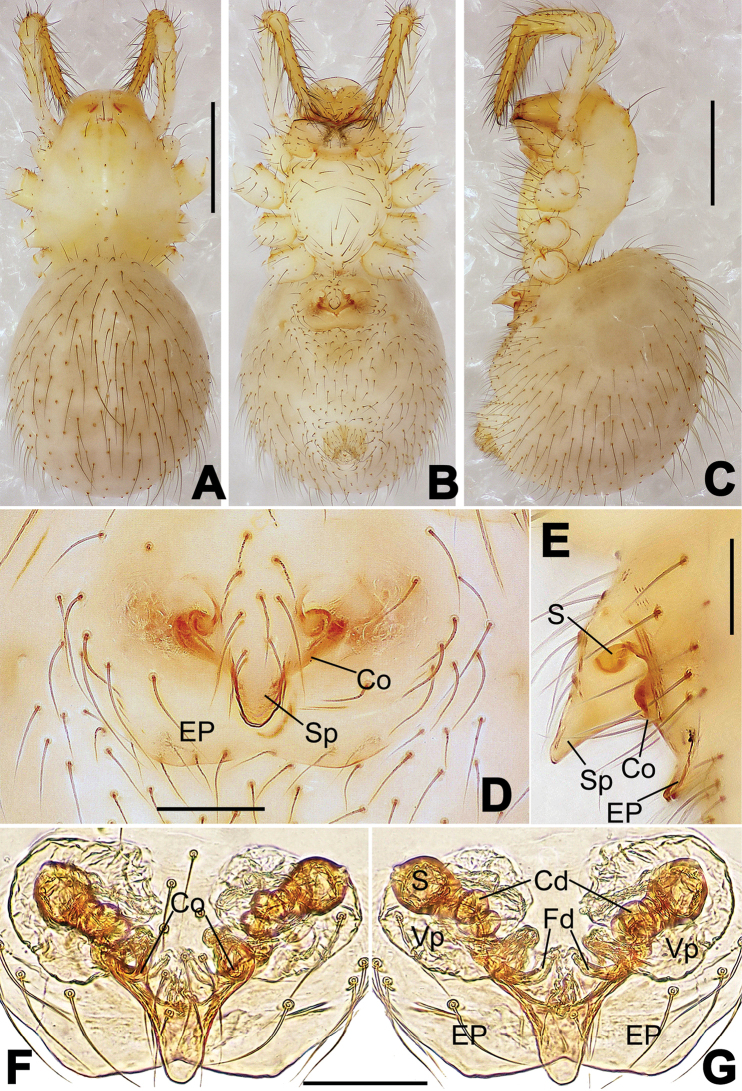
*Pseudonesticus wumengensis* sp. n., holotype (female). **A** Female habitus, dorsal view **B** Ditto, ventral view **C** Ditto, lateral view **D** Epigyne, ventral view **E** Ditto, lateral view **F** Vulva, ventral view **G** Vulva, dorsal view. Scale bars: **A–C** = 0.50 mm; **D–G** = 0.10 mm.

The new species is closely related to *Pseudonesticus dafangensis* sp. n. (Figs 70A–D, 71E–H) and *Pseudonesticus wumengensis* sp. n. (Fig. 76A–G). Males can be easily separated from those of the former species by the spiked distal process of the paracymbium (Dp), the shorter dorsal apophysis (Da) (Fig. 74A–B, D vs. Fig. 70A–B, D), the presence of a secondary tegular apophysis (Tg-II) (Fig. 74A vs. Fig. 70A) and the stouter, terminal apophysis (Ta) (Fig. 74A vs. Fig. 70A). Females can be distinguished from those of *Pseudonesticus dafangensis* sp. n. by the wider arrow-like scape (Sp), the ducts (Fd and Cd) closer to each other and the lighter coloration (Fig. 75A–H vs. Fig. 71A–H). They can be recognized from those of *Pseudonesticus wumengensis* sp. n. by the wider and more triangular scape (Sp) with a more pointed tip (Fig. 75E–H vs. Fig. 76D–G). The spiny distal process of the paracymbium (Dp) and the triangular, pointed epiginal scape (Sp) allow easy separation from all the other species of *Pseudonesticus*.

##### Description.

Habitus as in Fig. 75A–D. Carapace with sparse setae, pale yellow. Cephalic area flat. Eyes reduced to white eyespots. ALE=PLE=PME>AME. ALE and PLE adjoined. Cervical groove and fovea indistinct. Mouthparts and sternum pale yellow. Legs and female palps yellowish. Opisthosoma uniformly pale yellowish.

Male palp (Fig. 74A–D): paracymbium with a short dorsal apophysis (Fig. 74B, D) and a long distal process bearing long and thick spines at the distal and dorsal margins (Fig. 74D). Terminal apophysis stout, hook-like, located at the tip of the tegular apophysis (Fig. 74A). Tegular apophysis wide (Fig. 74A, C). Conductor curved in the middle, with a finger-like apex (Fig. 74A, C–D). Embolus long, coiled into four loops at apex of bulb (Fig. 74A, C–D).

Epigyne (Fig. 75E–H): broad and slightly, ventrally protruded, weakly sclerotized and partially translucent (Fig. 75E–F). Scape wide, arrowhead-like, basally strongly sclerotized (Fig. 75E). Inner structures nearly perpendicular to the opisthosoma (Fig. 75G–H). Spermathecae small, globular, separated by almost two diameters (Fig. 75H). Fertilization and copulatory ducts thin, long and coiled into three loops (Fig. 75G–H). Vulval pockets wide, located around the spermathecae and the fertilization and copulatory ducts (Fig. 75G–H).

Male (holotype). Total length 1.78. Carapace 0.84 long, 0.73 wide. Opisthosoma 1.03 long, 0.83 wide. Clypeus height 0.17. Sternum 0.54 long, 0.47 wide. Leg measurements: see Appendix A.

Female (one of the paratypes). Total length 1.82. Carapace 0.74 long, 0.69 wide. Opisthosoma 1.00 long, 1.08 wide. Clypeus length 0.15. Sternum 0.50 long, 0.45 wide. Leg measurements: see Appendix A.

##### Habitat.

Cave.

##### Distribution.

Known only from the type locality (Fig. 81).

#### Pseudonesticus wumengensis sp. n.

Taxon classificationAnimaliaAraneaeNesticidae

http://zoobank.org/17842682-72BD-4A8E-91B9-EA0B45DD373E

Figs 76 , 81

##### Type material.

Holotype ♀ and paratype 1♀ (IZCAS), CHINA: Guizhou Province, Hezhang County, Gaoyan Village, Mt. Wumeng, Tanjiayan Cave (27.20030°N, 104.59103°E, 2135 m), 17.XI.2011, H. Chen & Z. Zha leg.

##### Etymology.

The specific name is derived from the type locality; adjective.

##### Diagnosis.

This species is closely related to *Pseudonesticus dafangensis* sp. n. (see Fig. 71E–H) and *Pseudonesticus spinosus* sp. n. (see Fig. 75E–H). It can be distinguished from these two species by the longer and narrower scape (Sp), the wider distance between the spermathecae (S) and the wider epigynal plate (Ep) (Fig. 76E–G vs. Figs 71F–H, 75F–H). The same combination of characters allows to separate *Pseudonesticus wumengensis* sp. n. from all the other species of *Pseudonesticus*.

##### Description.

Habitus as in Fig. 76A–C. Carapace pale yellow. Eyes almost absent. Cervical groove and fovea indistinct. Mouthparts pale yellow, darker than the carapace. Sternum pale, heart-shaped, with long setae, posterior corner truncated. Legs and female palps yellowish, tarsi distally darker. Opisthosoma ovoid, pale yellow, covered with long setae.

Epigyne (Fig. 76D–G): weakly sclerotized. Scape long and narrow, with a blunt tip, protruding outward (Fig. 76E). Spermathecae small, globular, separated by about three diameters (Fig. 76F–G). Fertilization and copulatory ducts long, coiled into 2.5 loops (Fig. 76G). Vulval pockets wide, close to each others and located around the spermathecae and the fertilization and copulatory ducts (Fig. 76G).

Female (holotype). Total length 2.13. Carapace 0.95 long, 0.77 wide. Opisthosoma 1.38 long, 1.05 wide. Clypeus height 0.20. Sternum 0.60 long, 0.55 wide. Leg measurements: see Appendix A.

Male. Unknown.

##### Habitat.

Cave.

##### Distribution.

Known only from the type locality (Fig. 81).

#### Pseudonesticus ziyunensis sp. n.

Taxon classificationAnimaliaAraneaeNesticidae

http://zoobank.org/A2087014-CCC6-4DBF-9AD5-25B9B1FF7B34

Figs 77 , 78 , 81

##### Type material.

Holotype ♂ and paratypes 2♂12♀ (IZCAS), CHINA: Guizhou Province, Ziyun County, Mt. Wufeng, Wufeng Cave (25.75607°N, 106.07243°E, 1165 m), 25.XII.2010, H. Chen & Z. Zha leg.

##### Etymology.

The specific name is derived from the type locality; adjective.

##### Diagnosis.

**Figure 77.**
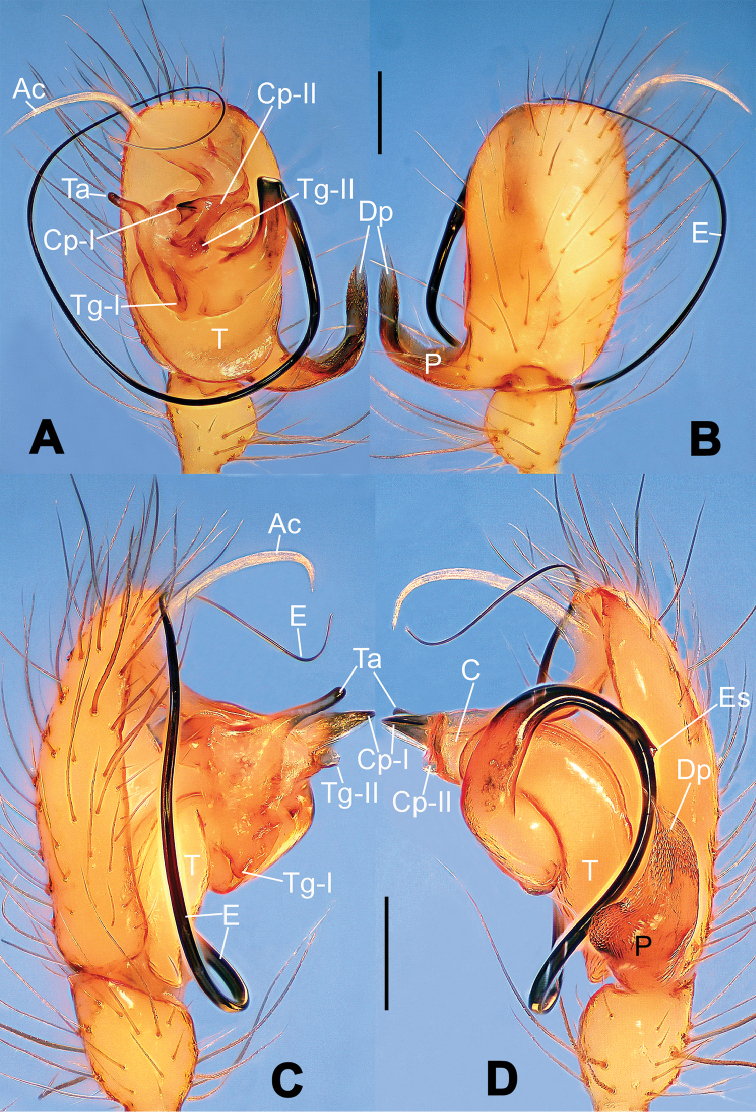
*Pseudonesticus ziyunensis* sp. n., holotype (male). **A** Palp, ventral view **B** Ditto, dorsal view **C** Ditto, prolateral view **D** Ditto, retrolateral view. Scale bars: 0.10 mm.

**Figure 78.**
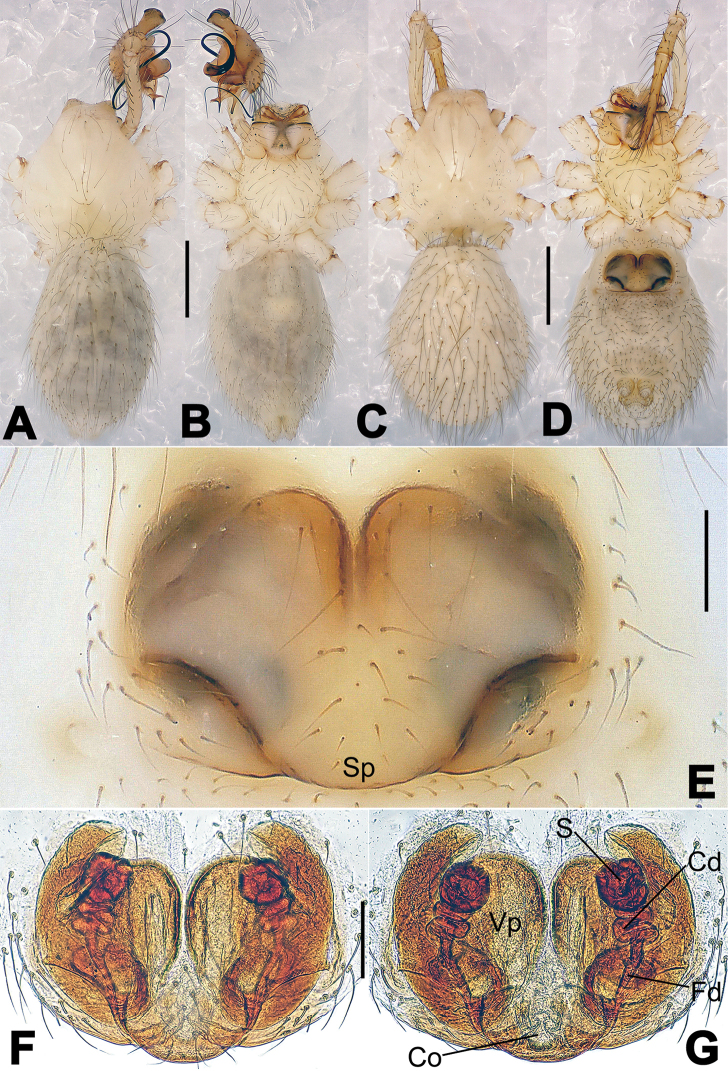
*Pseudonesticus ziyunensis* sp. n., holotype (male) and paratype (female). **A** Male habitus, dorsal view **B** Ditto, ventral view **C** Female habitus, dorsal view **D** Ditto, ventral view **E** Epigyne, ventral view **F** Vulva, ventral view **G** Vulva, dorsal view. Scale bars: **A–D** = 0.50 mm; **E–G** = 0.10 mm.

The new species is closely related to *Pseudonesticus miao* sp. n. (Figs 72A–D, 73F–H) and *Pseudonesticus clavatus* (see Liu and Li 2013a: 790, figs 1–14). Males can be distinguished from those of the former species by the shorter and undulate terminal apophysis (Ta), the longer process I of the conductor (Cp-I) and the pointed distal process of the paracymbium (Dp) (Fig. 77A–B, 77D vs. Fig. 72A–B, D). Females can be distinguished from those of *Pseudonesticus miao* sp. n. by the shorter and wider scape (Sp) (Fig. 78E–G). Males of *Pseudonesticus ziyunensis* sp. n. can be separated from those of *Pseudonesticus clavatus* by the shorter terminal apophysis (Ta), the longer distal process of the paracymbium (Dp), the shorter process I of the conductor (Cp-I), the lack of a ventral apophysis (Va); females are distinguished by the shorter and wider scape (Sp), the smaller spermathecae (S), the coiled ducts (missing any coil in the other species) and the wider and cystiform vulval pockets (Vp) (Figs 77A, 78E–G vs. figs 1, 12–14). The general shape of the paracymbium (P), the long terminal apophysis (Ta) and the general shape of the scape (Sp) allow an easy separation from all other species of *Pseudonesticus*.

##### Description.

Habitus as in Fig. 78A–D. Carapace pale yellow. Eyes absent. Legs and female palps pale yellowish, distally darker in each tarsus. Cervical groove and fovea indistinct. Mouthparts pale yellow, darker in females. Sternum pale yellow. Opisthosoma ovoid, greyish.

Male palp (Fig. 77A–D): cymbium sub-rectangular in dorsal view (Fig. 77B). Paracymbium with a flat and pointed distal process and a granulate and reticulate surface (Fig. 77A–B, D). Tegulum compressed and posteriorly protruding (Fig. 77A). Terminal apophysis sclerotized, long and bent, finger-like (Fig. 77A, C). Tg-I wide, Tg-II small (Fig. 77A, C–D). Conductor with a long, sharp apex and two small, flat processes (Fig. 77A, C–D). Embolus long, coiled in only one loop near the apex of the palp (Fig. 77A, C–D).

Epigyne (Fig. 78E–G): broad (Fig. 78E). Inner structure partially visible through the tegument (Fig. 78E). Scape short and very wide. Lateral grooves distinct and curved (Fig. 78E). Spermathecae small, globular, separated by less than 2.5 diameters (Fig. 78G). Fertilization ducts thin, long and proximally coiled into less than two loops and distally straight (Fig. 78G). Copulatory ducts broad, divided into two parts, the ovoid inner part shorter than the compressed outer part (Fig. 78G).

Male (holotype). Total length 2.72. Carapace 1.19 long, 1.06 wide. Opisthosoma 1.64 long, 1.00 wide. Sternum 0.66 long, 0.70 wide. Leg measurements: see Appendix A.

Female (one of the paratypes). Total length 3.16. Carapace 1.27 long, 1.06 wide. Opisthosoma 1.82 long, 1.24 wide. Sternum 0.70 long, 0.74 wide. Leg measurements: see Appendix A.

##### Habitat.

Cave.

##### Distribution.

Known only from the type locality (Fig. 81).

#### Genus Speleoticus

Taxon classificationAnimaliaAraneaeNesticidae

Ballarin & Li gen. n.

http://zoobank.org/BA55143C-D351-4DA7-BB82-BC627523EE9D

##### Type species.

*Speleoticus navicellatus* Liu & Li, 2013 from Guangxi, China.

##### Etymology.

The generic name is a combination of the Greek word “*Speleo*-” = cave and the contract name of *Nesticus*, the nominal genus of the family. It alludes to the troglophyllic lifestyle of these species. The gender is masculine.

##### Diagnosis.

Males belonging to *Speleoticus* gen. n. can be distinguished from those of the other Nesticini by the relatively simple, sickle-shaped paracymbium (P) with only a few short apophyses, in contrast to the other genera where it can be more complex, usually with wide and long processes. Furthermore, the elongate tibia and the triangular or rectangular protruding terminal apophysis (Ta) allow quick separation from the other Asian genera of Nesticidae. Females can be separated from those of the other Nesticini, with the exception of *Cyclocarcina*, by the wide, balloon-shaped vulval pockets (Vp) located above the spermathecae which are usually around or below the spermathecae in the other genera. Females of *Speleoticus* gen. n. can be easily separated from those of *Cyclocarcina* by the very short and narrow scape (Sp) which is well-developed and strongly protruding in the latter genus.

##### Description.

Total length: 2.84–3.15 (male), 2.97–4.36 (female). Carapace almost round in males, ovate in females, uniformly pale yellow as the legs. Six eyes in two rows, AME absent. Cervical groove and fovea indistinct. Chelicera with three promarginal teeth and multiple retromarginal tiny denticles on the fang furrow. Opisthosoma yellowish with long setae (Fig. 80A–B).

**Figure 79.**
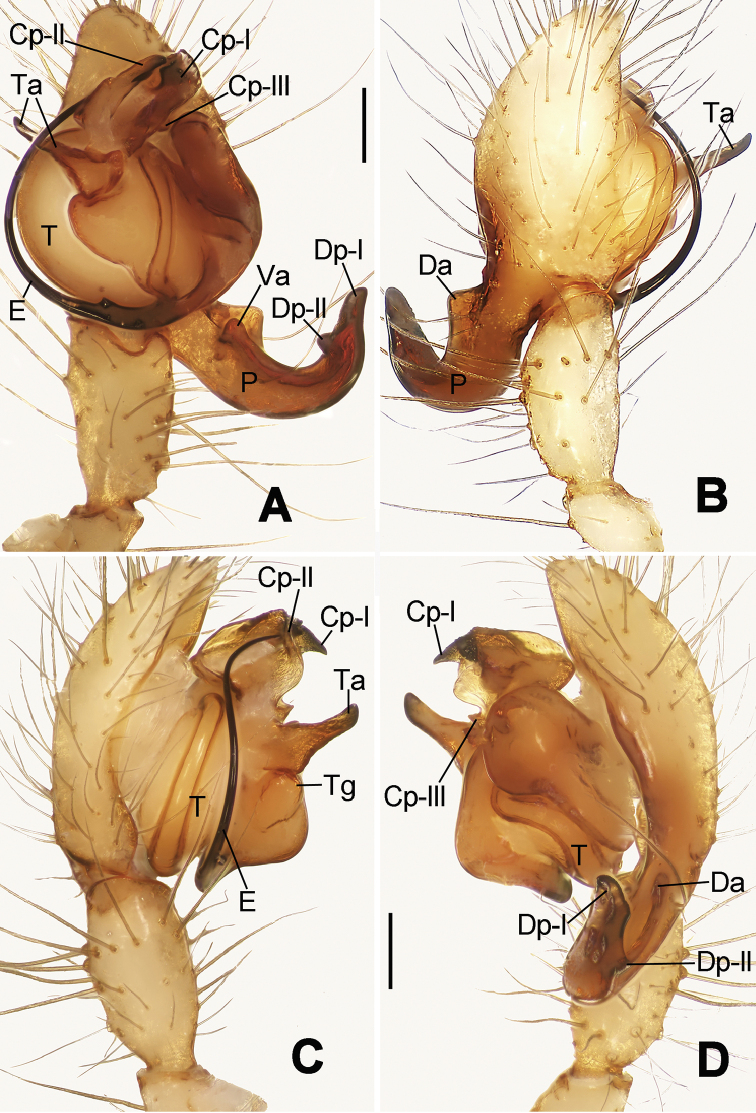
*Speleoticus navicellatus*, male from Du’an. **A** Palp, ventral view **B** Ditto, dorsal view **C** Ditto, prolateral view **D** Ditto, retrolateral view. Scale bars: 0.10 mm.

Male palp (Fig. 79A–D): tibia elongate (Fig. 79A–B). Paracymbium well-developed, sickle-like, with a single ventral apophysis usually short and squared; a short, flat dorsal apophysis and a sclerotized distal process with two ramifications (Fig. 79A–B, D). Terminal apophysis triangular or rectangular, elongate and protruding prolaterally. Tegular apophysis reduced (Fig. 79C). Conductor wide and convoluted, with three distinct processes, two elongate and one flat and laminar (Fig. 79A). Embolus filamentous, starting from the posterior side of the bulb and reaching the apex of the conductor with a half loop (Fig. 79A, C).

**Figure 80.**
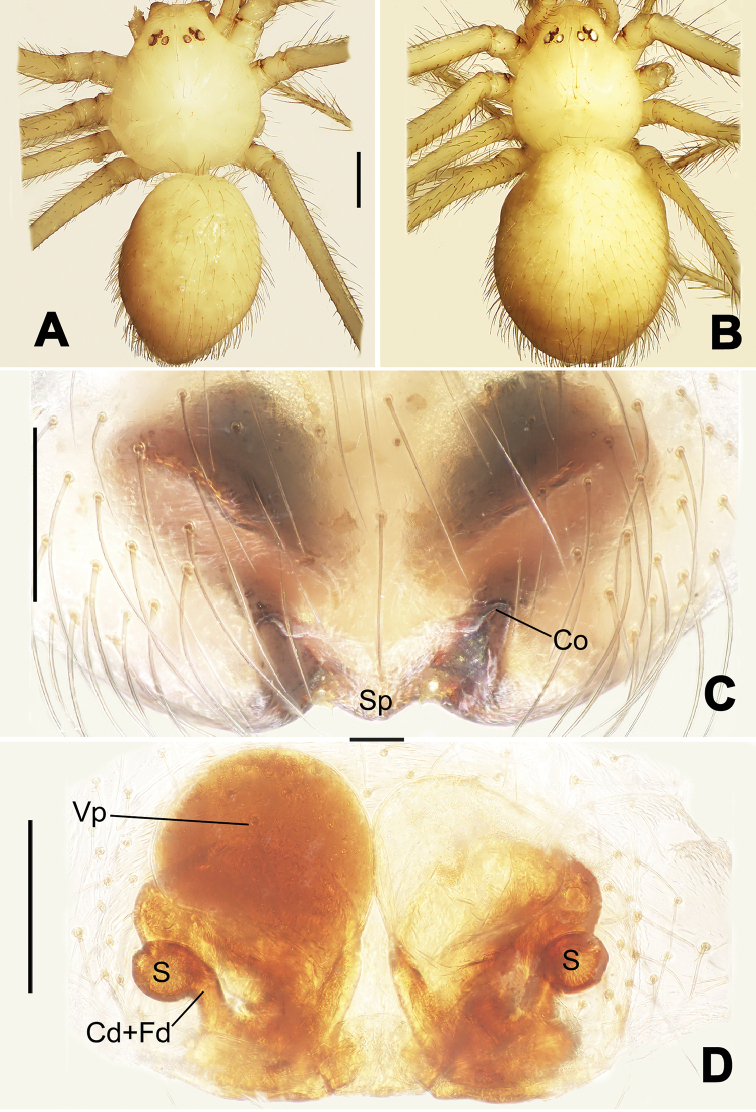
*Speleoticus navicellatus*, male and female from Du’an. **A** Male habitus, dorsal view **B** Female habitus, dorsal view **C** Epigyne, ventral view **D** Vulva, dorsal view. Scale bars: 0.10 mm.

Epigyne (Fig. 80C–D): broad, with a very short scape (Fig. 80C). Well sclerotized ducts partially visible through the tegument. Copulatory openings wide, located at the lateral side of the scape (Fig. 80C). Spermathecae small and almost round (Fig. 80D). Fertilization and copulatory ducts short and slightly convoluted (Fig. 80D). Vulval pockets well-developed, with a wide, balloon-like shape, located above the spermathecae (Fig. 80D).

**Figure 81.**
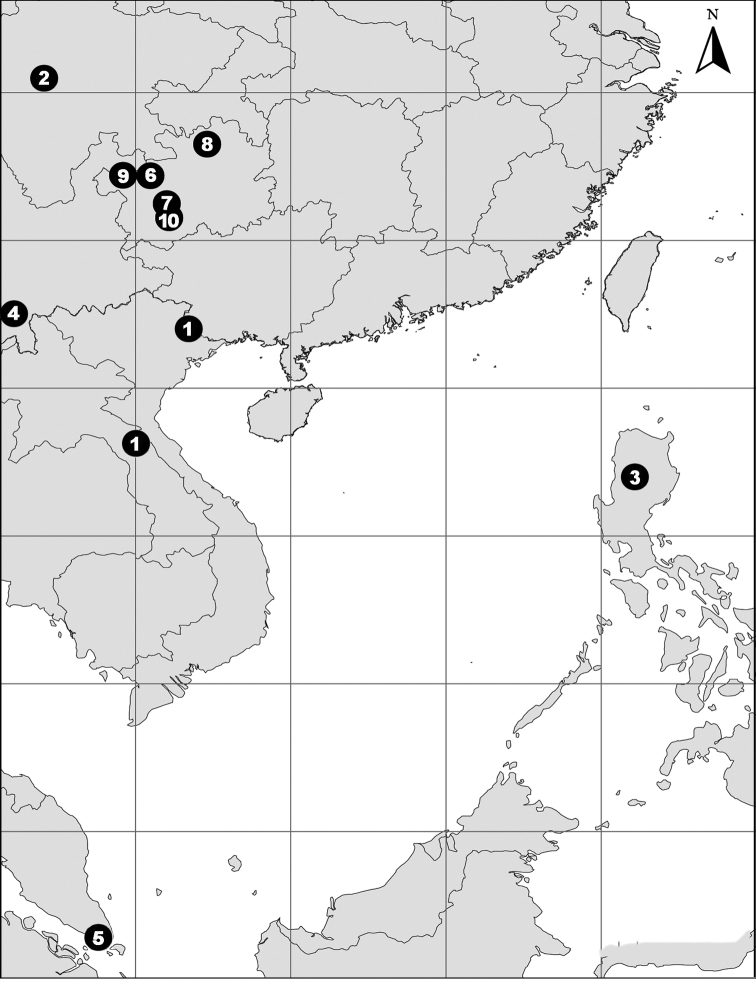
Distribution records of *Hamus*, *Nescina*, and *Pseudonesticus* spp. in China and Southeast Asia. **1** *Hamus cornutus* sp. n. **2** *Hamus kangdingensis* sp. n. **3** *Hamus luzon* sp. n. **4** *Hamus mangunensis* sp. n. **5** *Nescina kohi* sp. n. **6** *Pseudonesticus dafangensis* sp. n. **7** *Pseudonesticus miao* sp. n. **8** *Pseudonesticus spinosus* sp. n. **9** *Pseudonesticus wumengensis* sp. n. **10** *Pseudonesticus ziyunensis* sp. n.

**Figure 82.**
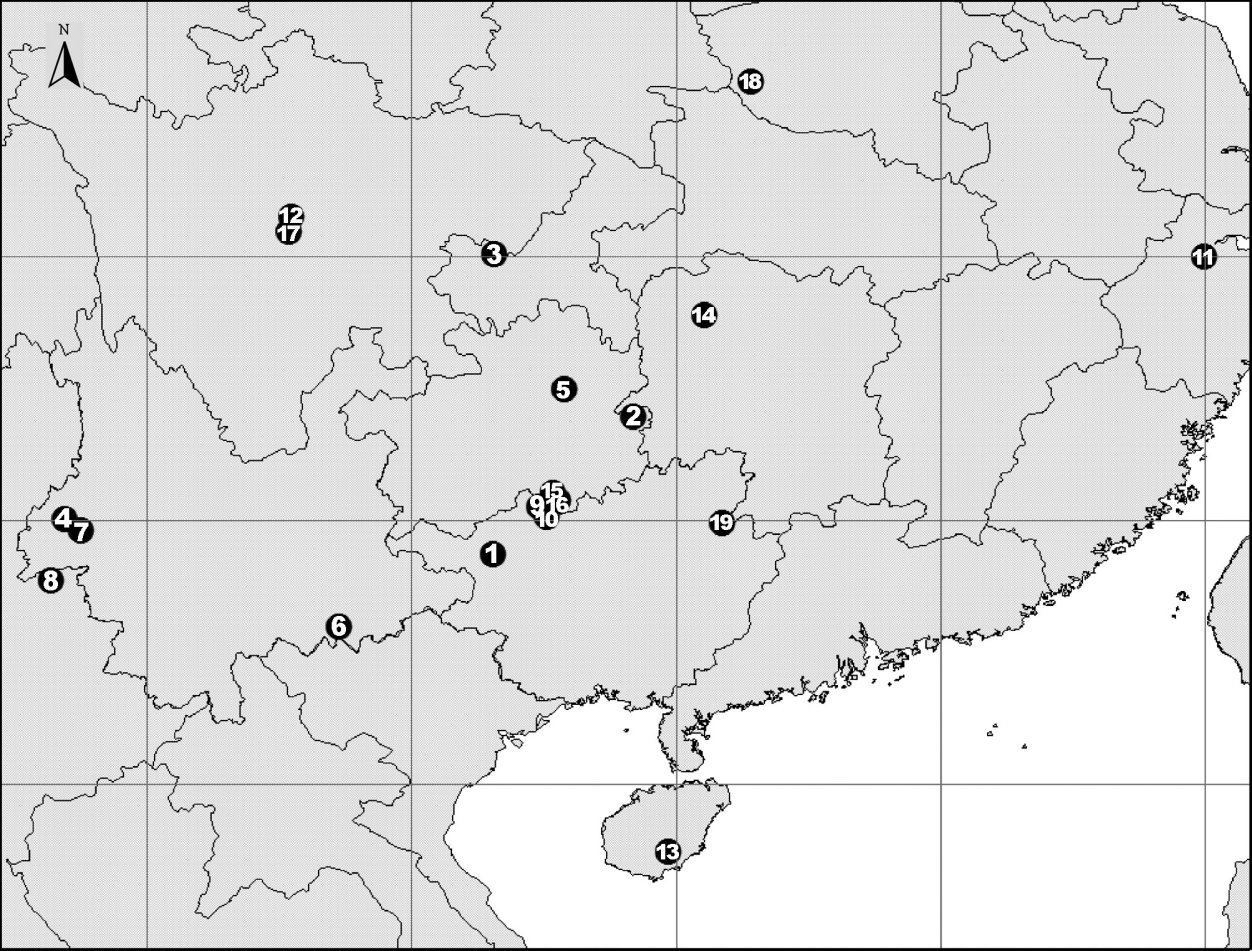
Distribution records of the *Nesticella* spiders belonging to the *brevipes*-group in China. **1** *Nesticella baiseensis* sp. n. **2** *Nesticella caeca* sp. n. **3** *Nesticella chongqing* sp. n. **4** *Nesticella dazhuangensis* sp. n. **5** *Nesticella gazuida* sp. n. **6** *Nesticella hongheensis* sp. n. **7** *Nesticella jingpo* sp. n. **8** *Nesticella lisu* sp. n. **9** *Nesticella liuzhaiensis* sp. n. **10** *Nesticella nandanensis* sp. n. **11** *Nesticella odonta* **12** *Nesticella qiaoqiensis* sp. n. **13** *Nesticella qiongensis* sp. n. **14** *Nesticella robusta* sp. n. **15** *Nesticella sanchaheensis* sp. n. **16** *Nesticella songi* **17** *Nesticella xiongmao* sp. n. **18** *Nesticella xixia* sp. n. **19** *Nesticella yao* sp. n.

**Figure 83.**
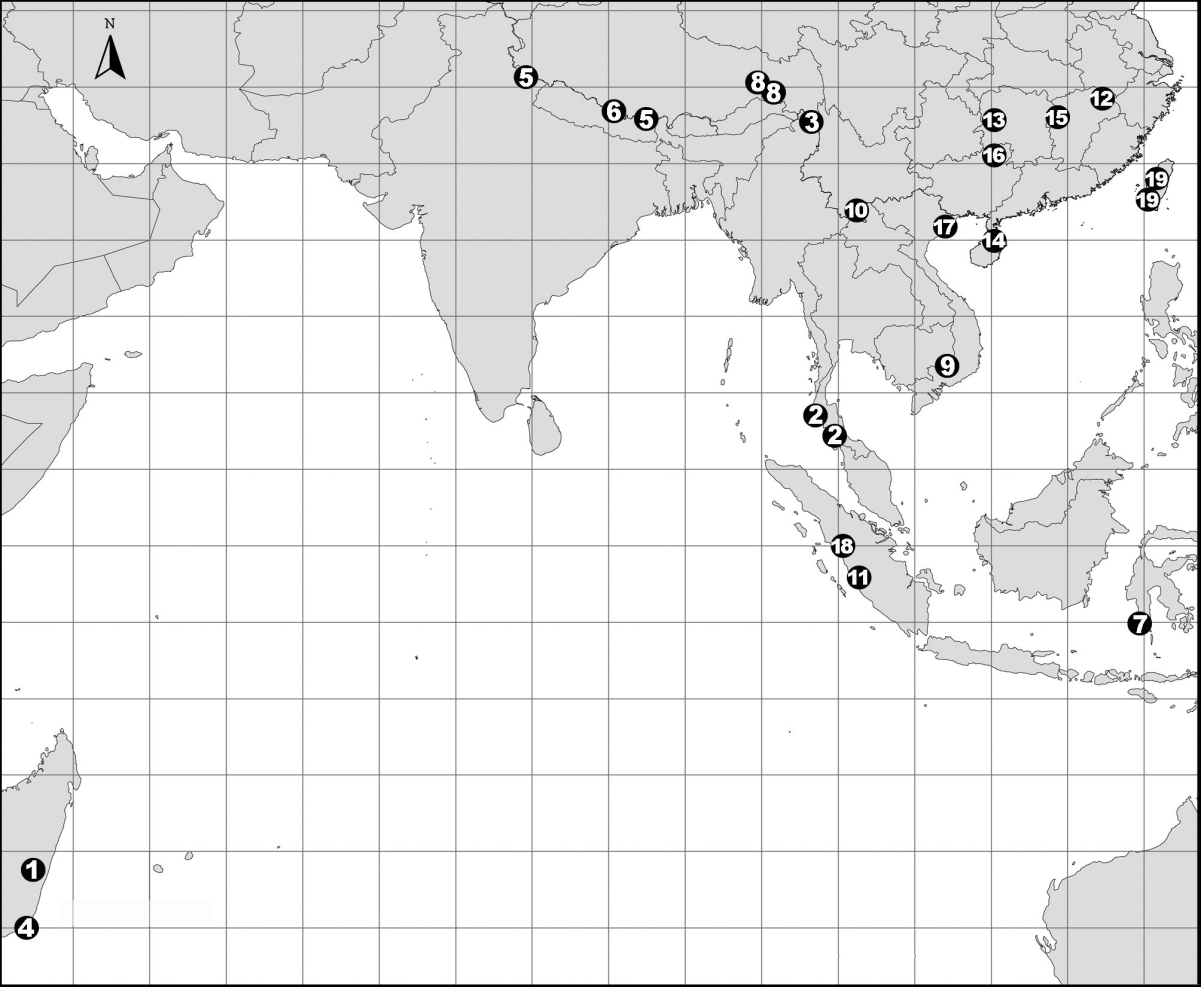
Distribution records of the *Nesticella* spiders belonging to the *nepalensis*, *mogera*, *phami* and *quelpartensis*-groups in Asia and Madagascar. **1** *Nesticella baobab* sp. n. **2** *Nesticella connectens* **3** *Nesticella gongshanensis* sp. n. **4** *Nesticella griswoldi* sp. n. **5** *Nesticella nepalensis* **6** *Nesticella potala* sp. n. **7** *Nesticella sulawesi* sp. n. **8** *Nesticella tibetana* sp. n. **9** *Nesticella vanlang* sp. n. **10** *Nesticella yui* Wunderlich et Song, 1995 **11** *Nesticella zhiyuani* sp. n. **12** *Nesticella fuliangensis* sp. n. **13** *Nesticella huomachongensis* sp. n. **14** *Nesticella rongtangensis* sp. n. **15** *Nesticella wanzaiensis* sp. n. **16** *Nesticella yanbeiensis* sp. n. **17** *Nesticella phami* sp. n. **18** *Nesticella sumatrana* sp. n. **19** *Nesticella kaohsiungensis* sp. n.

##### Composition.

*Speleoticus globosus* (Liu & Li, 2013), comb. n., *Speleoticus libo* (Chen & Zhu, 2005), comb. n., *Speleoticus navicellatus* (Liu & Li, 2013), comb. n., *Speleoticus uenoi* (Yaginuma, 1972), comb. n., and *Speleoticus yaginumai* (Yin, 2012), comb. n. All the species listed above are transferred from the genus *Nesticus*. All new combinations are supported by our molecular phylogenetic analysis.

##### Distribution.

China (Guangxi, Guizhou, Hunan), Japan (Honshu Island, Shizuoka Prefecture).

##### Remarks.

*Speleoticus* gen. n., together with the *Pseudonesticus* Liu & Li, 2013, show typical adaptations to cave life, such as the absence or reduction of the eyes (in particular the AME), long legs, lack of pigmentation, etc. The close relationship between these two genera, rather than with the genera from the West Palaearctic and North America, can be cautiously hypothesized based on the morphological comparison and preliminary molecular analysis of Nesticidae. Nevertheless, further studies are necessary to understand their correct systematic position within the family. Here we illustrate male and female of *Speleoticus navicellatus*, the type species of *Speleoticus* gen. n.

#### Speleoticus navicellatus

Taxon classificationAnimaliaAraneaeNesticidae

(Liu & Li, 2013) comb. n.

Figs 79 , 80

Nesticus navicellatus Liu & Li, 2013b: 542, figs 35A–B, 36A–D, 37A–D, 38A–E (♂♀).

##### Type material examined.

Holotype ♂, paratype 1♀ (IZCAS) CHINA: Guangxi Zhuang Autonomous Region, Da’ua County, Qibailong Town, Qiaoxu Village, Qiaoxu Cave (24.07606°N, 107.67063°E, 550 m), 9.III.2007, Y. Lin & J. Liu leg.

##### Other material.

1♂ (IZCAS) CHINA: Guangxi Zhuang Autonomous Region, Du’an County, Huangqi Village, Cave 1 (24.38745°N, 108.24698°E), 25.VI.2013, Y. Lin leg.; 1♀ (IZCAS) CHINA: Guangxi Zhuang Autonomous Region, Du’an County, Nongqv Village, Cave 1 (24.24431°N, 108.05053°E, 287 m), 27.VI.2013, Y. Lin leg.

##### Diagnosis.

See Liu and Li (2013b).

##### Description.

See Figs 79A–D, 80A–D and Liu and Li (2013b).

##### Habitat.

Cave.

##### Distribution.

China (Guangxi).

## Supplementary Material

XML Treatment for Hamus

XML Treatment for Hamus bowoensis

XML Treatment for Hamus cornutus

XML Treatment for Hamus kangdingensis

XML Treatment for Hamus luzon

XML Treatment for Hamus mangunensis

XML Treatment for Nescina

XML Treatment for Nescina minuta

XML Treatment for Nescina kohi

XML Treatment for Nesticella

XML Treatment for Nesticella baiseensis

XML Treatment for Nesticella brevipes

XML Treatment for Nesticella caeca

XML Treatment for Nesticella chongqing

XML Treatment for Nesticella dazhuangensis

XML Treatment for Nesticella gazuida

XML Treatment for Nesticella hongheensis

XML Treatment for Nesticella jingpo

XML Treatment for Nesticella lisu

XML Treatment for Nesticella liuzhaiensis

XML Treatment for Nesticella mollicula

XML Treatment for Nesticella nandanensis

XML Treatment for Nesticella odonta

XML Treatment for Nesticella qiaoqiensis

XML Treatment for Nesticella qiongensis

XML Treatment for Nesticella robusta

XML Treatment for Nesticella sanchaheensis

XML Treatment for Nesticella songi

XML Treatment for Nesticella xiongmao

XML Treatment for Nesticella xixia

XML Treatment for Nesticella yao

XML Treatment for Nesticella fuliangensis

XML Treatment for Nesticella huomachongensis

XML Treatment for Nesticella mogera

XML Treatment for Nesticella rongtangensis

XML Treatment for Nesticella wanzaiensis

XML Treatment for Nesticella yanbeiensis

XML Treatment for Nesticella baobab

XML Treatment for Nesticella connectens

XML Treatment for Nesticella gongshanensis

XML Treatment for Nesticella griswoldi

XML Treatment for Nesticella nepalensis

XML Treatment for Nesticella potala

XML Treatment for Nesticella sulawesi

XML Treatment for Nesticella tibetana

XML Treatment for Nesticella vanlang

XML Treatment for Nesticella yui

XML Treatment for Nesticella zhiyuani

XML Treatment for Nesticella phami

XML Treatment for Nesticella sumatrana

XML Treatment for Nesticella kaohsiungensis

XML Treatment for Nesticella quelpartensis

XML Treatment for Pseudonesticus

XML Treatment for Pseudonesticus clavatus

XML Treatment for Pseudonesticus dafangensis

XML Treatment for Pseudonesticus miao

XML Treatment for Pseudonesticus spinosus

XML Treatment for Pseudonesticus wumengensis

XML Treatment for Pseudonesticus ziyunensis

XML Treatment for Speleoticus

XML Treatment for Speleoticus navicellatus

## Appendix A

**Table:** Leg measurements of the new species described in the text.

***Hamus cornutus* sp. n.**					
Male					
	I	II	III	IV	
Femur	1.23	1.03	0.76	1.05	
Patella	0.33	0.31	0.27	0.30	
Tibia	1.14	0.84	0.55	0.89	
Metatarsus	0.78	0.68	0.46	0.63	
Tarsus	0.70	0.65	0.54	0.65	
**Total**	**4.18**	**3.51**	**2.58**	**3.52**	
Female					
	I	II	III	IV	Palp
Femur	1.25	1.06	0.79	1.19	0.35
Patella	0.36	0.36	0.31	0.35	0.15
Tibia	1.08	0.87	0.55	1.00	0.19
Metatarsus	0.79	0.68	0.47	0.66	‒
Tarsus	0.69	0.66	0.52	0.64	0.41
**Total**	**4.17**	**3.63**	**2.64**	**3.84**	**1.10**
***Hamus kangdingensis* sp. n.**					
Male					
	I	II	III	IV	
Femur	1.12	0.94	0.70	1.05	
Patella	0.30	0.28	0.24	0.28	
Tibia	0.92	0.72	0.49	0.84	
Metatarsus	0.63	0.53	0.40	0.53	
Tarsus	0.64	0.57	0.47	0.59	
**Total**	**3.61**	**3.04**	**2.30**	**3.29**	
***Hamus luzon* sp. n.**					
Female					
	I	II	III	IV	Palp
Femur	1.21	1.02	0.80	1.22	0.39
Patella	0.39	0.36	0.32	0.35	0.17
Tibia	0.99	0.80	0.58	1.01	0.21
Metatarsus	0.71	0.61	0.47	0.65	‒
Tarsus	0.62	0.57	0.52	0.61	0.42
**Total**	**3.92**	**3.36**	**2.69**	**3.84**	**1.19**
***Nescina kohi* sp. n.**					
Male					
	I	II	III	IV	
Femur	0.67	0.57	0.50	0.61	
Patella	0.23	0.20	0.19	0.22	
Tibia	0.54	0.46	0.32	0.47	
Metatarsus	0.37	0.32	0.29	0.35	
Tarsus	0.31	0.26	0.25	0.28	
**Total**	**2.12**	**1.81**	**1.55**	**1.93**	
Female					
	I	II	III	IV	Palp
Femur	0.68	0.62	0.53	0.64	0.33
Patella	0.22	0.22	0.19	0.21	0.18
Tibia	0.53	0.46	0.33	0.49	0.23
Metatarsus	0.36	0.33	0.30	0.35	‒
Tarsus	0.30	0.29	0.26	0.27	0.40
**Total**	**2.09**	**1.92**	**1.61**	**1.96**	**1.14**
***Nesticella baiseensis* sp. n.**					
Male					
	I	II	III	IV	
Femur	2.90	2.37	1.92	2.60	
Patella	0.65	0.60	0.52	0.61	
Tibia	2.71	1.98	1.40	2.08	
Metatarsus	2.68	2.00	1.12	1.92	
Tarsus	1.15	0.94	0.76	0.88	
**Total**	**10.09**	**7.89**	**5.72**	**8.09**	
Female					
	I	II	III	IV	Palp
Femur	2.55	2.00	1.60	2.21	0.69
Patella	0.64	0.58	0.52	0.61	0.24
Tibia	2.32	1.60	1.08	1.76	0.35
Metatarsus	2.08	1.52	1.14	1.48	‒
Tarsus	1.02	0.88	0.71	0.82	0.81
**Total**	**8.61**	**6.58**	**5.05**	**6.88**	**2.09**
***Nesticella caeca* sp. n.**					
Male					
	I	II	III	IV	
Femur	2.43	1.99	1.60	2.15	
Patella	0.53	0.50	0.41	0.45	
Tibia	2.30	1.68	1.18	1.78	
Metatarsus	2.32	1.67	1.20	1.63	
Tarsus	1.04	0.85	0.73	0.83	
**Total**	**8.62**	**6.69**	**5.12**	**6.84**	
Female					
	I	II	III	IV	Palp
Femur	2.45	1.98	1.58	2.19	0.65
Patella	0.55	0.48	0.41	0.50	0.23
Tibia	2.23	1.63	1.06	1.72	0.32
Metatarsus	2.10	1.50	1.10	1.44	‒
Tarsus	1.05	0.90	0.72	0.84	0.75
**Total**	**8.38**	**6.49**	**4.87**	**6.69**	**1.95**
***Nesticella chongqing* sp. n.**					
Female					
	I	II	III	IV	Palp
Femur	1.98	‒	1.14	1.75	0.55
Patella	0.53	‒	0.40	0.50	0.23
Tibia	1.75	‒	0.78	1.35	0.29
Metatarsus	1.63	‒	0.85	1.15	‒
Tarsus	0.78	‒	0.55	0.63	0.64
**Total**	**6.67**	‒	**3.72**	**5.38**	**1.71**
***Nesticella dazhuangensis* sp. n.**					
Male					
	I	II	III	IV	
Femur	2.08	1.70	1.40	1.90	
Patella	0.58	0.53	0.44	0.53	
Tibia	1.83	1.35	0.96	1.53	
Metatarsus	1.93	1.36	1.06	1.48	
Tarsus	0.80	0.70	0.62	0.73	
**Total**	**7.22**	**5.64**	**4.48**	**6.17**	
Female					
	I	II	III	IV	Palp
Femur	1.72	1.38	1.05	1.58	0.52
Patella	0.51	0.43	0.40	0.50	0.21
Tibia	1.55	1.13	0.75	1.25	0.27
Metatarsus	1.44	1.04	0.80	1.10	‒
Tarsus	0.72	0.63	0.55	0.60	0.60
**Total**	**5.94**	**4.61**	**3.55**	**5.03**	**1.60**
***Nesticella gazuida* sp. n.**					
Female					
	I	II	III	IV	Palp
Femur	2.47	2.00	1.58	2.16	0.65
Patella	0.56	0.50	0.44	0.52	0.23
Tibia	2.25	1.66	1.06	1.73	0.34
Metatarsus	2.13	1.56	1.13	1.53	‒
Tarsus	0.88	0.78	0.69	0.75	0.73
**Total**	**8.29**	**6.50**	**4.90**	**6.69**	**1.95**
***Nesticella hongheensis* sp. n.**					
Male					
	I	II	III	IV	
Femur	1.88	1.44	1.15	1.63	
Patella	0.50	0.44	0.38	0.41	
Tibia	1.72	1.16	0.78	1.31	
Metatarsus	1.78	1.19	0.94	1.25	
Tarsus	0.77	0.64	0.52	0.62	
**Total**	**6.65**	**4.87**	**3.77**	**5.22**	
Female					
	I	II	III	IV	Palp
Femur	1.82	1.38	1.11	1.62	0.54
Patella	0.51	0.48	0.38	0.46	0.22
Tibia	1.54	1.02	0.70	1.22	0.28
Metatarsus	1.45	1.03	0.79	1.04	‒
Tarsus	0.73	0.61	0.51	0.58	0.60
**Total**	**6.05**	**4.52**	**3.49**	**4.92**	**1.64**
***Nesticella jingpo* sp. n.**					
Female					
	I	II	III	IV	Palp
Femur	1.48	1.18	0.93	1.35	0.44
Patella	0.43	0.40	0.38	0.41	0.18
Tibia	1.33	0.90	0.63	1.08	0.22
Metatarsus	1.08	0.88	0.69	0.93	‒
Tarsus	0.70	0.56	0.49	0.55	0.53
**Total**	**5.02**	**3.92**	**3.12**	**4.32**	**1.37**
***Nesticella lisu* sp. n.**					
Male					
	I	II	III	IV	
Femur	1.85	1.45	1.15	1.66	
Patella	0.49	0.46	0.38	0.43	
Tibia	1.69	1.14	0.80	1.35	
Metatarsus	1.68	1.13	0.83	1.25	
Tarsus	0.75	0.65	0.55	0.65	
**Total**	**6.46**	**4.83**	**3.71**	**5.34**	
Female					
	I	II	III	IV	Palp
Femur	1.75	1.41	1.15	1.63	0.54
Patella	0.49	0.47	0.38	0.48	0.21
Tibia	1.54	1.06	0.75	1.29	0.27
Metatarsus	1.40	1.00	0.81	1.05	‒
Tarsus	0.71	0.63	0.56	0.65	0.59
**Total**	**5.89**	**4.57**	**3.65**	**5.10**	**1.61**
***Nesticella liuzhaiensis* sp. n.**					
Female					
	I	II	III	IV	Palp
Femur	1.53	1.18	0.93	1.40	0.40
Patella	0.40	0.37	0.33	0.38	0.19
Tibia	1.33	0.89	0.62	1.35	0.21
Metatarsus	1.18	0.81	0.63	0.88	‒
Tarsus	0.64	0.55	0.49	0.58	0.51
**Total**	**5.08**	**3.80**	**3.00**	**4.59**	**1.31**
***Nesticella nandanensis* sp. n.**					
Male					
	I	II	III	IV	
Femur	2.30	1.81	1.53	2.06	
Patella	0.59	0.53	0.45	0.53	
Tibia	2.13	1.50	1.10	1.69	
Metatarsus	2.19	1.56	1.21	1.59	
Tarsus	0.94	0.78	0.65	0.75	
**Total**	**8.15**	**6.18**	**4.94**	**6.62**	
Female					
	I	II	III	IV	Palp
Femur	2.44	1.91	1.52	2.15	0.71
Patella	0.66	0.60	0.49	0.61	0.25
Tibia	2.23	1.53	1.03	1.73	0.36
Metatarsus	2.08	1.50	1.09	1.49	‒
Tarsus	0.91	0.78	0.65	0.74	0.80
**Total**	**8.32**	**6.32**	**4.78**	**6.72**	**2.12**
***Nesticella qiaoqiensis* sp. n.**					
Female					
	I	II	III	IV	Palp
Femur	‒	1.53	1.16	1.72	0.55
Patella	‒	0.47	0.41	0.49	0.21
Tibia	‒	1.19	0.75	1.28	0.27
Metatarsus	‒	1.14	0.84	1.13	‒
Tarsus	‒	0.69	0.59	0.63	0.63
**Total**	‒	**5.02**	**3.75**	**5.25**	**1.66**
***Nesticella qiongensis* sp. n.**					
Male					
	I	II	III	IV	
Femur	1.93	1.42	1.13	1.62	
Patella	0.45	0.40	0.33	0.42	
Tibia	1.83	1.16	0.78	1.36	
Metatarsus	1.85	1.14	0.87	1.28	
Tarsus	0.74	0.60	0.52	0.62	
**Total**	**6.80**	**4.72**	**3.63**	**5.30**	
Female					
	I	II	III	IV	Palp
Femur	1.60	1.21	0.95	1.58	0.45
Patella	0.46	0.41	0.35	0.45	0.19
Tibia	1.36	0.89	0.59	1.34	0.25
Metatarsus	1.24	0.86	0.67	1.22	‒
Tarsus	0.64	0.56	0.48	0.63	0.53
**Total**	**5.30**	**3.93**	**3.04**	**5.22**	**1.42**
***Nesticella robusta* sp. n.**					
Male					
	I	II	III	IV	
Femur	2.03	1.47	1.15	1.66	
Patella	0.46	0.42	0.34	0.43	
Tibia	1.87	1.18	0.80	1.39	
Metatarsus	1.84	1.15	0.91	1.30	
Tarsus	0.76	0.61	0.54	0.63	
**Tatal**	**6.96**	**4.83**	**3.74**	**5.41**	
Female					
	I	II	III	IV	Palp
Femur	1.82	1.38	1.08	1.67	0.46
Patella	0.47	0.44	0.33	0.45	0.19
Tibia	1.38	1.12	0.78	1.34	0.26
Metatarsus	1.25	1.08	0.73	1.23	‒
Tarsus	0.68	0.60	0.50	0.63	0.55
**Tatal**	**5.60**	**4.62**	**3.42**	**5.32**	**1.46**
***Nesticella sanchaheensis* sp. n.**					
Male					
	I	II	III	IV	
Femur	2.84	2.34	‒	‒	
Patella	0.59	0.53	‒	‒	
Tibia	2.56	1.69	‒	‒	
Metatarsus	2.50	1.88	‒	‒	
Tarsus	1.09	0.88	‒	‒	
**Total**	**9.58**	**7.32**	‒	‒	
Female					
	I	II	III	IV	Palp
Femur	3.16	2.75	2.04	2.64	0.78
Patella	0.64	0.61	0.48	0.60	0.26
Tibia	2.90	2.15	1.39	2.20	0.40
Metatarsus	2.72	1.90	1.48	2.06	‒
Tarsus	1.20	0.93	0.79	1.04	0.89
**Total**	**10.62**	**8.34**	**6.18**	**8.54**	**2.33**
***Nesticella xiongmao* sp. n.**					
Male					
	I	II	III	IV	
Femur	2.19	1.63	1.28	1.84	
Patella	0.48	0.47	0.41	0.48	
Tibia	2.05	1.00	0.91	1.48	
Metatarsus	1.97	1.27	0.97	1.36	
Tarsus	0.80	0.66	0.53	0.63	
**Total**	**7.49**	**5.03**	**4.10**	**5.79**	
Female					
	I	II	III	IV	Palp
Femur	1.83	1.38	1.12	1.63	0.52
Patella	0.50	0.46	0.38	0.48	0.22
Tibia	1.65	1.08	0.73	1.26	0.26
Metatarsus	1.48	1.00	0.78	1.05	‒
Tarsus	0.73	0.63	0.53	0.64	0.58
**Total**	**6.19**	**4.55**	**3.54**	**5.06**	**1.58**
***Nesticella xixia* sp. n.**					
Male					
	I	II	III	IV	
Femur	3.00	2.32	1.85	2.55	
Patella	0.68	0.64	0.53	0.59	
Tibia	2.76	1.91	1.33	2.03	
Metatarsus	2.68	1.88	1.45	1.91	
Tarsus	1.04	0.91	0.73	0.88	
**Total**	**10.16**	**7.66**	**5.89**	**7.96**	
Female					
	I	II	III	IV	Palp
Femur	2.88	2.20	1.80	2.50	0.78
Patella	0.64	0.60	0.52	0.63	0.22
Tibia	2.68	1.81	1.18	1.94	0.41
Metatarsus	2.36	1.68	1.31	1.63	‒
Tarsus	1.00	0.85	0.69	0.78	0.88
**Total**	**9.56**	**7.14**	**5.50**	**7.48**	**2.29**
***Nesticella yao* sp. n.**					
Female					
	I	II	III	IV	Palp
Femur	2.35	1.81	1.50	2.03	0.66
Patella	0.56	0.53	0.48	0.53	0.23
Tibia	2.09	1.45	0.98	1.63	0.32
Metatarsus	1.94	1.38	1.08	1.34	‒
Tarsus	0.91	0.78	0.65	0.70	0.76
**Total**	**7.85**	**5.95**	**4.69**	**6.23**	**1.97**
***Nesticella fuliangensis* sp. n.**					
Male					
	I	II	III	IV	
Femur	2.66	2.13	1.75	2.47	
Patella	0.66	0.63	0.53	0.63	
Tibia	2.59	1.88	1.38	2.22	
Metatarsus	2.61	1.91	1.47	2.16	
Tarsus	1.00	0.88	0.75	0.91	
**Total**	**9.52**	**7.43**	**5.88**	**8.39**	
Female					
	I	II	III	IV	Palp
Femur	2.63	2.06	1.72	2.38	0.80
Patella	0.69	0.63	0.56	0.63	0.30
Tibia	2.44	1.75	1.25	2.07	0.50
Metatarsus	2.34	1.69	1.30	1.75	‒
Tarsus	1.00	0.88	0.78	0.89	0.90
**Total**	**9.10**	**7.01**	**5.61**	**7.72**	**2.50**
***Nesticella huomachongensis* sp. n.**					
Male					
	I	II	III	IV	
Femur	2.34	1.84	1.59	2.19	
Patella	0.63	0.56	0.47	0.59	
Tibia	2.28	1.66	1.22	1.95	
Metatarsus	2.33	1.69	1.31	1.91	
Tarsus	1.04	0.88	0.72	0.89	
**Total**	**8.62**	**6.63**	**5.31**	**7.53**	
Female					
	I	II	III	IV	Palp
Femur	2.32	1.84	1.56	2.16	0.70
Patella	0.64	0.60	0.51	0.59	0.29
Tibia	2.12	1.53	1.09	1.63	0.35
Metatarsus	2.06	1.52	1.16	1.56	‒
Tarsus	1.00	0.88	0.75	0.83	0.87
**Total**	**8.14**	**6.37**	**5.07**	**6.77**	**2.21**
***Nesticella rongtangensis* sp. n.**					
Male					
	I	II	III	IV	
Femur	2.09	1.59	1.34	1.88	
Patella	0.47	0.41	0.35	0.42	
Tibia	2.03	1.44	1.03	1.69	
Metatarsus	2.00	1.38	1.06	1.53	
Tarsus	0.84	0.66	0.56	0.71	
**Total**	**7.43**	**5.48**	**4.34**	**6.23**	
***Nesticella wanzaiensis* sp. n.**					
Male					
	I	II	III	IV	
Femur	2.28	1.72	1.50	2.00	
Patella	0.61	0.53	0.45	0.50	
Tibia	2.23	1.59	1.14	1.81	
Metatarsus	2.31	1.56	1.22	1.69	
Tarsus	1.06	0.83	0.72	0.84	
**Total**	**8.49**	**6.23**	**5.03**	**6.84**	
Female					
	I	II	III	IV	Palp
Femur	2.36	1.77	1.56	2.13	
Patella	0.65	0.62	0.49	0.60	
Tibia	2.31	1.65	1.18	1.92	
Metatarsus	2.33	1.60	1.25	1.73	
Tarsus	1.12	0.93	0.80	0.88	
**Total**	**8.77**	**6.57**	**5.28**	**7.26**	
***Nesticella yanbeiensis* sp. n.**					
Male					
	I	II	III	IV	
Femur	2.22	1.83	1.53	2.00	
Patella	0.66	0.60	0.48	0.53	
Tibia	2.05	1.53	1.15	1.78	
Metatarsus	2.06	1.55	1.23	1.66	
Tarsus	1.03	0.91	0.73	0.91	
**Total**	**8.02**	**6.42**	**5.12**	**6.88**	
Female					
	I	II	III	IV	Palp
Femur	2.31	1.91	1.56	2.15	0.72
Patella	0.66	0.63	0.50	0.58	0.25
Tibia	2.13	1.53	1.10	1.80	0.38
Metatarsus	2.00	1.47	1.11	1.58	‒
Tarsus	0.97	0.88	0.70	0.85	0.82
**Total**	**8.07**	**6.42**	**4.97**	**6.96**	**2.17**
***Nesticella baobab* sp. n.**					
Male					
	I	II	III	IV	
Femur	1.55	1.23	0.96	1.43	
Patella	0.44	0.40	0.33	0.39	
Tibia	1.48	1.01	0.68	1.19	
Metatarsus	1.40	0.93	0.74	1.38	
Tarsus	0.63	0.53	0.45	0.55	
**Total**	**5.50**	**4.10**	**3.16**	**4.94**	
***Nesticella connectens* Wunderlich, 1995**					
Male					
	I	II	III	IV	
Femur	1.98	1.64	1.42	1.76	
Patella	0.55	0.51	0.49	0.52	
Tibia	1.83	1.41	0.96	1.48	
Metatarsus	1.75	1.35	0.92	1.43	
Tarsus	0.98	0.84	0.58	0.83	
**Total**	**7.09**	**5.75**	**4.37**	**6.02**	
Female					
	I	II	III	IV	Palp
Femur	1.94	1.58	1.28	1.80	0.63
Patella	0.54	0.49	0.46	0.51	0.24
Tibia	1.77	1.24	0.87	1.42	0.34
Metatarsus	1.62	1.22	0.98	1.37	‒
Tarsus	0.77	0.66	0.57	0.65	0.73
**Total**	**6.64**	**5.19**	**4.16**	**5.75**	**1.94**
***Nesticella gongshanensis* sp. n.**					
Female					
	I	II	III	IV	Palp
Femur	‒	1.44	1.14	1.64	0.54
Patella	‒	0.45	0.31	0.44	0.22
Tibia	‒	1.12	0.76	1.27	0.30
Metatarsus	‒	1.07	0.81	1.14	‒
Tarsus	‒	0.63	0.52	0.62	0.59
**Total**	‒	**4.71**	**3.54**	**5.11**	**1.65**
***Nesticella griswoldi* sp. n.**					
Male					
	I	II	III	IV	
Femur	1.60	1.25	0.98	1.41	
Patella	0.48	0.43	0.35	0.40	
Tibia	1.50	1.05	0.67	1.17	
Metatarsus	1.50	1.00	0.66	0.90	
Tarsus	0.81	0.56	0.40	0.49	
**Total**	**5.89**	**4.29**	**3.06**	**4.37**	
Female					
	I	II	III	IV	Palp
Femur	1.49	1.14	0.86	1.34	0.42
Patella	0.44	0.40	0.35	0.42	‒
Tibia	1.29	0.86	0.56	1.02	‒
Metatarsus	1.19	0.84	0.62	0.96	‒
Tarsus	0.60	0.50	0.45	0.52	‒
**Total**	**5.01**	**3.74**	**2.84**	**4.26**	‒
***Nesticella potala* sp. n.**					
Female					
	I	II	III	IV	Palp
Femur	2.56	2.09	1.65	‒	0.73
Patella	0.59	0.57	0.51	‒	0.26
Tibia	2.34	1.72	1.13	‒	0.36
Metatarsus	2.33	1.42	1.26	‒	‒
Tarsus	0.91	1.10	0.70	‒	0.89
**Total**	**8.73**	**6.90**	**5.25**	‒	**2.24**
***Nesticella sulawesi* sp. n.**					
Female					
	I	II	III	IV	Palp
Femur	1.76	1.40	‒	1.64	0.54
Patella	0.52	0.48	‒	0.48	0.21
Tibia	1.58	1.16	‒	1.33	0.29
Metatarsus	1.47	1.10	‒	1.22	‒
Tarsus	0.76	0.66	‒	0.68	0.64
**Total**	**6.09**	**4.80**	**0.00**	**5.35**	**1.68**
***Nesticella tibetana* sp. n.**					
Male					
	I	II	III	IV	
Femur	1.64	1.36	1.09	1.59	
Patella	0.43	0.41	0.34	0.46	
Tibia	1.28	1.14	0.75	1.22	
Metatarsus	1.32	1.36	0.84	1.10	
Tarsus	0.59	0.58	0.48	0.62	
**Total**	**5.26**	**4.85**	**3.50**	**4.99**	
Female					
	I	II	III	IV	Palp
Femur	1.58	1.24	1.01	1.52	0.49
Patella	0.49	0.46	0.38	0.44	0.20
Tibia	1.37	0.92	0.64	1.10	0.25
Metatarsus	1.28	0.93	0.71	1.06	‒
Tarsus	0.64	0.56	0.50	0.62	0.55
**Total**	**5.36**	**4.11**	**3.24**	**4.74**	**1.49**
***Nesticella vanlang* sp. n.**					
Female					
	I	II	III	IV	Palp
Femur	1.68	1.29	1.00	‒	0.48
Patella	0.50	0.43	0.38	‒	0.19
Tibia	1.50	1.04	0.63	‒	0.25
Metatarsus	1.45	1.00	0.73	‒	‒
Tarsus	0.73	0.59	0.50	‒	0.57
**Total**	**5.86**	**4.35**	**3.24**	‒	**1.49**
***Nesticella zhiyuani* sp. n.**					
Male					
	I	II	III	IV	
Femur	1.76	1.43	1.11	1.64	
Patella	0.50	0.46	0.38	0.44	
Tibia	1.70	1.22	0.81	1.41	
Metatarsus	1.66	1.21	0.89	1.40	
Tarsus	0.73	0.62	0.48	0.63	
**Total**	**6.35**	**4.94**	**3.67**	**5.52**	
Female					
	I	II	III	IV	Palp
Femur	1.39	1.19	0.97	1.14	0.48
Patella	0.46	0.41	0.37	0.42	0.19
Tibia	1.36	0.97	0.65	1.15	0.27
Metatarsus	1.26	0.90	0.70	1.04	‒
Tarsus	0.67	0.56	0.48	0.56	0.55
**Total**	**5.14**	**4.03**	**3.17**	**4.31**	**1.49**
***Nesticella phami* sp. n.**					
Male					
	I	II	III	IV	
Femur	1.50	1.24	1.00	1.42	
Patella	0.41	0.40	0.34	0.40	
Tibia	1.30	0.94	0.68	1.12	
Metatarsus	1.44	1.03	0.77	1.18	
Tarsus	0.62	0.57	0.48	0.56	
**Total**	**5.27**	**4.18**	**3.27**	**4.68**	
Female					
	I	II	III	IV	Palp
Femur	1.69	1.40	1.11	1.60	0.52
Patella	0.48	0.46	0.38	0.46	0.22
Tibia	1.44	1.05	0.73	1.24	0.27
Metatarsus	1.42	1.11	0.79	1.19	‒
Tarsus	0.70	0.62	0.54	0.63	0.61
**Total**	**5.73**	**4.64**	**3.55**	**5.12**	**1.62**
***Nesticella sumatrana* sp. n.**					
Male					
	I	II	III	IV	
Femur	1.43	1.20	1.03	1.28	
Patella	0.38	0.37	0.30	0.37	
Tibia	1.13	0.88	0.72	1.05	
Metatarsus	1.05	0.85	0.75	1.00	
Tarsus	0.55	0.51	0.49	0.53	
**Total**	**4.54**	**3.81**	**3.29**	**4.23**	
***Nesticella kaohsiungensis* sp. n.**					
Male					
	I	II	III	IV	
Femur	1.81	1.52	1.16	1.66	
Patella	0.47	0.44	0.36	0.44	
Tibia	1.72	1.19	0.83	1.34	
Metatarsus	1.75	1.22	0.90	1.30	
Tarsus	0.75	0.66	0.53	0.64	
**Total**	**6.50**	**5.03**	**3.78**	**5.38**	
Female					
	I	II	III	IV	Palp
Femur	1.78	1.34	1.16	1.69	0.54
Patella	0.47	0.44	0.39	0.46	0.22
Tibia	1.63	1.09	0.73	1.32	0.28
Metatarsus	1.50	1.08	0.81	1.13	‒
Tarsus	0.73	0.61	0.51	0.59	0.63
**Total**	**6.11**	**4.56**	**3.60**	**5.19**	**1.67**
***Pseudonesticus dafangensis* sp. n.**					
Male					
	I	II	III	IV	
Femur	1.44	1.19	0.98	1.43	
Patella	0.40	0.38	0.31	0.37	
Tibia	1.41	0.98	0.70	1.25	
Metatarsus	1.28	1.05	0.74	1.06	
Tarsus	0.76	0.65	0.52	0.65	
**Total**	**5.29**	**4.25**	**3.25**	**4.76**	
Female					
	I	II	III	IV	Palp
Femur	1.40	1.14	0.97	1.39	0.44
Patella	0.43	0.38	0.35	0.41	0.18
Tibia	1.31	0.93	0.66	1.22	0.28
Metatarsus	1.13	0.84	0.72	0.94	‒
Tarsus	0.75	0.63	0.54	0.62	0.56
**Total**	**5.02**	**3.92**	**3.24**	**4.58**	**1.46**
***Pseudonesticus miao* sp. n.**					
Male					
	I	II	III	IV	
Femur	3.50	2.90	2.23	3.00	
Patella	0.49	0.47	0.40	0.47	
Tibia	3.70	2.72	1.73	2.57	
Metatarsus	3.62	2.70	1.78	2.35	
Tarsus	1.35	1.15	0.83	0.98	
**Total**	**12.66**	**9.94**	**6.97**	**9.37**	
Female					
	I	II	III	IV	Palp
Femur	3.35	2.64	2.23	2.81	0.76
Patella	0.52	0.50	0.41	0.47	0.22
Tibia	3.24	2.36	1.45	2.23	0.41
Metatarsus	3.00	2.23	1.54	1.97	‒
Tarsus	1.22	1.03	0.75	0.91	0.86
**Total**	**11.33**	**8.76**	**6.38**	**8.39**	**2.25**
***Pseudonesticus spinosus* sp. n.**					
Male					
	I	II	III	IV	
Femur	1.88	1.53	1.23	1.78	
Patella	0.36	0.34	0.31	0.34	
Tibia	1.87	1.38	0.91	1.56	
Metatarsus	1.74	1.31	1.01	1.34	
Tarsus	0.90	0.81	0.66	0.75	
**Total**	**6.75**	**5.37**	**4.12**	**5.77**	
Female					
	I	II	III	IV	Palp
Femur	1.72	1.33	1.08	1.63	0.43
Patella	0.39	0.35	0.31	0.36	0.17
Tibia	1.60	1.13	0.79	1.33	0.24
Metatarsus	1.46	1.09	0.83	1.10	‒
Tarsus	0.88	0.75	0.60	0.70	0.5
**Total**	**6.05**	**4.65**	**3.61**	**5.12**	**1.34**
***Pseudonesticus wumengensis* sp. n.**					
Female					
	I	II	III	IV	Palp
Femur	1.65	1.43	1.14	1.66	0.47
Patella	0.39	0.38	0.36	0.38	0.16
Tibia	1.38	1.23	0.82	1.41	0.27
Metatarsus	1.08	1.08	0.80	1.12	‒
Tarsus	0.73	0.72	0.63	0.70	0.52
**Total**	**5.23**	**4.84**	**3.75**	**5.27**	**1.42**
***Pseudonesticus ziyunensis* sp. n.**					
Male					
	I	II	III	IV	
Femur	2.75	2.35	2.00	2.55	
Patella	0.53	0.48	0.43	0.48	
Tibia	2.69	2.13	1.50	2.29	
Metatarsus	2.63	2.11	1.65	2.03	
Tarsus	1.20	1.00	0.76	0.91	
**Total**	**9.80**	**8.07**	**6.34**	**8.26**	
Female					
	I	II	III	IV	Palp
Femur	2.92	2.38	1.99	2.66	0.83
Patella	0.57	0.56	0.48	0.52	0.25
Tibia	2.84	2.16	1.48	2.28	0.46
Metatarsus	2.68	2.05	1.67	2.00	‒
Tarsus	1.26	1.04	0.73	0.95	0.89
**Total**	**10.27**	**8.19**	**6.35**	**8.41**	**2.43**

## Appendix B

**Table:** Uncorrected genetic pairwise distance of the COI partial sequence between the species discussed in this text.

	Species	1	2	3	4	5	6	7	8	9	10	11	12	13	14	15	16	17	18	19	20	21	22	23	24	25	26	27	28	29	30	31	32	33	34	35	36	37	38	39	40	41
**1**	*Hamus bowoensis*																																									
**2**	*Hamus cornutus* sp. n.	0.215																																								
**3**	*Hamus luzon* sp. n.	0.163	0.236																																							
**4**	*Nescina kohi* sp. n.	0.248	0.234	0.234																																						
**5**	*Nescina minuta*	0.232	0.234	0.228	0.097																																					
**6**	*Nesticella baiseensis* sp. n.	0.190	0.206	0.218	0.218	0.204																																				
**7**	*Nesticella baobab* sp. n.	0.232	0.228	0.218	0.224	0.215	0.163																																			
**8**	*Nesticella caeca* sp. n.	0.226	0.215	0.242	0.254	0.200	0.127	0.178																																		
**9**	*Nesticella chongqing* sp. n.	0.213	0.204	0.228	0.228	0.200	0.118	0.165	0.120																																	
**10**	*Nesticella connectens*	0.236	0.261	0.236	0.213	0.210	0.165	0.151	0.190	0.190																																
**11**	*Nesticella dazhuangensis* sp. n.	0.208	0.220	0.236	0.222	0.204	0.133	0.184	0.157	0.137	0.208																															
**12**	*Nesticella fuliangensis* sp. n.	0.238	0.240	0.248	0.271	0.267	0.161	0.196	0.176	0.167	0.196	0.146																														
**13**	*Nesticella gazuida* sp. n.	0.215	0.213	0.236	0.248	0.211	0.135	0.182	0.079	0.129	0.194	0.174	0.184																													
**14**	*Nesticella gonshanensis* sp. n.	0.204	0.210	0.244	0.250	0.232	0.165	0.186	0.163	0.159	0.211	0.172	0.163	0.186																												
**15**	*Nesticella griswoldi* sp. n.	0.224	0.211	0.228	0.213	0.210	0.165	0.091	0.180	0.169	0.153	0.180	0.200	0.186	0.186																											
**16**	*Nesticella hongheensis* sp. n.	0.215	0.222	0.238	0.252	0.222	0.144	0.180	0.137	0.131	0.182	0.169	0.180	0.153	0.170	0.186																										
**17**	*Nesticella huomachongensis* sp. n.	0.222	0.234	0.248	0.269	0.250	0.151	0.190	0.167	0.159	0.186	0.148	0.051	0.163	0.170	0.186	0.176																									
**18**	*Nesticella jingpo* sp. n.	0.220	0.232	0.234	0.244	0.215	0.151	0.196	0.159	0.148	0.200	0.081	0.140	0.153	0.184	0.172	0.170	0.140																								
**19**	*Nesticella kaohsiungensis* sp. n.	0.236	0.218	0.248	0.236	0.215	0.148	0.180	0.182	0.153	0.165	0.172	0.184	0.188	0.165	0.151	0.172	0.157	0.172																							
**20**	*Nesticella liuzhaiensis* sp. n.	0.182	0.170	0.208	0.234	0.224	0.133	0.180	0.161	0.148	0.202	0.140	0.159	0.167	0.169	0.172	0.150	0.153	0.159	0.163																						
**21**	*Nesticella nandanensis* sp. n.	0.204	0.202	0.232	0.234	0.196	0.079	0.170	0.118	0.131	0.184	0.150	0.184	0.120	0.157	0.174	0.146	0.178	0.161	0.161	0.148																					
**22**	*Nesticella odonta*	0.215	0.213	0.228	0.215	0.192	0.107	0.169	0.115	0.048	0.178	0.140	0.165	0.133	0.167	0.169	0.127	0.153	0.151	0.170	0.153	0.122																				
**23**	*Nesticella phami* sp. n.	0.230	0.228	0.258	0.246	0.230	0.150	0.196	0.167	0.146	0.206	0.155	0.153	0.167	0.170	0.196	0.176	0.150	0.161	0.178	0.157	0.151	0.151																			
**24**	*Nesticella potala* sp. n.	0.211	0.240	0.242	0.246	0.211	0.144	0.178	0.150	0.133	0.182	0.155	0.165	0.140	0.172	0.161	0.172	0.153	0.131	0.146	0.186	0.150	0.150	0.146																		
**25**	*Nesticella qiaoqiensis* sp. n.	0.206	0.210	0.240	0.244	0.224	0.142	0.174	0.153	0.138	0.188	0.151	0.163	0.151	0.178	0.163	0.144	0.153	0.151	0.153	0.129	0.148	0.146	0.153	0.159																	
**26**	*Nesticella qiongensis* sp. n.	0.211	0.211	0.240	0.250	0.215	0.159	0.186	0.172	0.155	0.182	0.176	0.178	0.161	0.194	0.176	0.170	0.165	0.167	0.144	0.140	0.148	0.159	0.155	0.148	0.135																
**27**	*Nesticella robusta* sp. n.	0.200	0.211	0.198	0.208	0.206	0.124	0.167	0.144	0.122	0.182	0.144	0.174	0.135	0.169	0.165	0.151	0.170	0.146	0.169	0.163	0.113	0.122	0.140	0.151	0.150	0.151															
**28**	*Nesticella sanchaheensis* sp. n.	0.232	0.226	0.242	0.252	0.232	0.151	0.188	0.111	0.140	0.218	0.165	0.190	0.109	0.182	0.192	0.155	0.180	0.165	0.190	0.170	0.150	0.142	0.180	0.146	0.176	0.178	0.157														
**29**	*Nesticella sulawesi* sp. n.	0.236	0.242	0.230	0.213	0.206	0.157	0.157	0.176	0.167	0.151	0.169	0.182	0.174	0.170	0.157	0.174	0.167	0.174	0.157	0.165	0.174	0.165	0.174	0.150	0.170	0.174	0.163	0.192													
**30**	*Nesticella sumatrana* sp. n.	0.215	0.210	0.228	0.213	0.206	0.159	0.178	0.176	0.155	0.182	0.172	0.176	0.178	0.170	0.163	0.172	0.167	0.163	0.155	0.146	0.155	0.155	0.172	0.159	0.150	0.169	0.140	0.184	0.186												
**31**	*Nesticella tibetana* sp. n.	0.250	0.236	0.238	0.248	0.224	0.174	0.159	0.176	0.176	0.159	0.163	0.172	0.182	0.196	0.157	0.186	0.174	0.167	0.174	0.180	0.178	0.184	0.186	0.155	0.169	0.178	0.180	0.194	0.150	0.174											
**32**	*Nesticella vanlang* sp. n.	0.202	0.218	0.215	0.208	0.194	0.190	0.138	0.188	0.188	0.148	0.174	0.198	0.200	0.184	0.116	0.172	0.192	0.184	0.188	0.174	0.184	0.184	0.198	0.172	0.192	0.190	0.192	0.206	0.157	0.192	0.169										
**33**	*Nesticella wanzaiensis* sp. n.	0.218	0.215	0.236	0.263	0.234	0.146	0.169	0.176	0.159	0.184	0.148	0.086	0.186	0.178	0.172	0.180	0.072	0.150	0.180	0.151	0.170	0.155	0.167	0.167	0.163	0.172	0.174	0.194	0.182	0.157	0.148	0.178									
**34**	*Nesticella xixia* sp. n.	0.228	0.240	0.228	0.230	0.224	0.142	0.190	0.155	0.150	0.194	0.161	0.180	0.163	0.190	0.184	0.161	0.167	0.186	0.159	0.155	0.167	0.155	0.165	0.172	0.135	0.174	0.151	0.182	0.196	0.155	0.211	0.194	0.176								
**35**	*Nesticella yanbeiensis* sp. n.	0.230	0.228	0.242	0.254	0.234	0.159	0.198	0.192	0.161	0.190	0.148	0.063	0.194	0.170	0.188	0.180	0.065	0.135	0.174	0.159	0.184	0.163	0.170	0.165	0.153	0.180	0.182	0.204	0.180	0.169	0.169	0.180	0.090	0.196							
**36**	*Nesticella yao* sp. n.	0.174	0.222	0.206	0.222	0.210	0.090	0.194	0.150	0.144	0.176	0.146	0.169	0.150	0.159	0.176	0.146	0.170	0.146	0.159	0.146	0.084	0.144	0.157	0.144	0.137	0.135	0.125	0.165	0.163	0.163	0.184	0.172	0.159	0.157	0.157						
**37**	*Nesticella zhiyuani* sp. n.	0.244	0.226	0.248	0.218	0.188	0.180	0.155	0.190	0.148	0.153	0.165	0.194	0.186	0.180	0.163	0.172	0.184	0.161	0.163	0.188	0.165	0.163	0.188	0.142	0.190	0.188	0.170	0.192	0.153	0.172	0.157	0.151	0.178	0.194	0.176	0.178					
**38**	*Pseudonesticus dafangensis* sp. n.	0.238	0.238	0.236	0.234	0.230	0.182	0.220	0.218	0.186	0.213	0.192	0.228	0.220	0.218	0.226	0.210	0.226	0.202	0.200	0.202	0.211	0.194	0.234	0.222	0.232	0.234	0.208	0.218	0.228	0.208	0.226	0.215	0.228	0.188	0.222	0.196	0.190				
**39**	*Pseudonesticus miao* sp. n.	0.246	0.210	0.230	0.210	0.176	0.202	0.230	0.198	0.180	0.211	0.190	0.232	0.208	0.210	0.211	0.213	0.215	0.196	0.211	0.182	0.192	0.184	0.204	0.204	0.198	0.192	0.190	0.224	0.192	0.190	0.220	0.218	0.208	0.224	0.224	0.204	0.165	0.192			
**40**	*Pseudonesticus spinosus* sp. n.	0.228	0.208	0.230	0.220	0.211	0.178	0.215	0.208	0.169	0.222	0.188	0.222	0.204	0.213	0.202	0.198	0.210	0.172	0.192	0.192	0.198	0.174	0.211	0.188	0.204	0.204	0.196	0.208	0.210	0.184	0.190	0.210	0.215	0.196	0.210	0.190	0.184	0.072	0.163		
**41**	*Pseudonesticus wumengensis* sp. n.	0.238	0.228	0.230	0.234	0.226	0.184	0.220	0.213	0.180	0.220	0.202	0.228	0.215	0.210	0.220	0.210	0.224	0.204	0.200	0.200	0.208	0.190	0.236	0.220	0.228	0.232	0.202	0.220	0.224	0.196	0.220	0.224	0.224	0.194	0.218	0.196	0.184	0.018	0.184	0.069	
**42**	*Pseudonesticus ziyunensis* sp. n.	0.224	0.190	0.218	0.222	0.208	0.176	0.215	0.190	0.184	0.230	0.192	0.226	0.188	0.180	0.202	0.194	0.213	0.196	0.202	0.174	0.186	0.186	0.234	0.208	0.204	0.198	0.198	0.200	0.198	0.196	0.215	0.204	0.194	0.202	0.220	0.188	0.192	0.174	0.125	0.148	0.169

## References

[B1] Ballarin F Li S (2015) Three new genera of the family Nesticidae (Arachnida: Araneae) from Tibet and Yunnan, China. Zoological Systematics 40(2): 179–190.

[B2] Chen H Zhu M (2004) A new cave spider of the genus *Nesticella* from China (Araneae, Nesticidae). Acta Zootaxonomica Sinica 29: 87–88.

[B3] Chen Z (1984) A new species of spider of the genus *Nesticus* from China (Araneae: Nesticidae). Acta Zootaxonomica Sinica 9: 34–36.

[B4] Chen Z Zhang Z (1991) Fauna of Zhejiang: Araneida. Zhejiang Science and Technology Publishing House, Hangzhou, 356 pp.

[B5] Dunlop JA Penney D Jekel D (2016) A summary list of fossil spiders and their relatives. The World Spider Catalog, version 17.0, 286 pp.

[B6] Grall E Jäger P (2016) Four new species of the spider genus *Nesticella* Lehtinen & Saaristo, 1980 from Laos, Thailand and Myanmar and the first description of the male of *Nesticella yui* Wunderlich & Song, 1995 with a proposed new diagnostic character for the family Nesticidae Simon, 1894 (Arachnida, Araneae). Zootaxa 4085(2): 248–264. doi: 10.11646/zootaxa.4085.2.5 27394301 10.11646/zootaxa.4085.2.5

[B7] Gray MR (1989) Cavernicolous spiders (Arancae) from Undara, Queensland and Cape Range, Western Australia. Helictite 27: 87–89.

[B8] Hall TA (1999) BioEdit: a user-friendly biological sequence alignment editor and analysis program for Windows 95/98/NT. Nucleic Acids Sympposium Series 41: 95–98.

[B9] Hubert M (1970) Description de deux espèces nouvelles d’araignées africaines, appartenant au genre *Nesticus* (Araneae, Nesticidae). Revue de Zoologie et de Botanique Africaines 81: 361–368.

[B10] Hubert M (1971) Sur un *Nesticus* nouveau d’Angola: *N. machadoi* nov. sp. (Araneae, Nesticidae). Publicações Culturais da Companhia de Diamantes de Angola 84: 73–78.

[B11] Hubert M (1973) Araignées du Népal, II. *Nesticus nepalensis* n. sp. (Arachnida: Nesticidae). Senckenbergiana Biologica 54: 165–169.

[B12] Kamura T Irie T (2009) Nesticidae. In: Ono H (Ed.) The Spiders of Japan with keys to the families and genera and illustrations of the species. Tokai University Press, Kanagawa, 345–355.

[B13] Khmelik VV Kozub D Glazunov A (2006) Helicon Focus 3.10.3. http://www.heliconsoft.com/heliconfocus.html [accessed 20 August, 2013]

[B14] Lehtinen PT Saaristo MI (1980) Spiders of the Oriental-Australian region. II. Nesticidae. Annales Zoologici Fennici 17: 47–66.

[B15] Li S Lin Y (2016) Species Catalogue of China. Volume 2. Animals. Invertebrates (I), Arachnida: Araneae. Science Press, Beijing, 549 pp.

[B16] Liu J Li S (2013a) A new genus and species of the family Nesticidae from Yunnan, China (Arachnida, Araneae). Acta Zootaxonomica Sinica 38(4): 790–794.

[B17] Liu J Li S (2013b) New cave-dwelling spiders of the family Nesticidae (Arachnida, Araneae) from China. Zootaxa 3613(6): 501–547. doi: 10.11646/zootaxa.3613.6.1 24698836 10.11646/zootaxa.3613.6.1

[B18] Marusik YM Guseinov EF (2003) Spiders (Arachnia: Aranei) of Azerbaijan. 1. New family and genus records. Arthropoda Selecta 12(1): 29–46.

[B19] Marusik YM Kovblyuk MM (2011) Spiders (Arachnida, Aranei) of Siberia and Russian Far East. KMK Scientific Press, Moscow, 344 pp.

[B20] Namkung J (2002) The Spiders of Korea. Kyo-Hak Publishing Co., Seoul, 648 pp.

[B21] Namkung J (2003) The Spiders of Korea, 2nd. ed Kyo-Hak Publishing Co., Seoul, 648 pp.

[B22] Paik KY (1978) Araneae. Illustrated Fauna and Flora of Korea 21: 1–548.

[B23] Paik KY Yaginuma T Namkung J (1969) Results of the speleological survey in South Korea 1966 XIX. Cave-dwelling spiders from the southern part of Korea. Bulletin of the National Museum of Nature and Science, Tokyo 12: 795–844.

[B24] Platnick NI (1989) Advances in Spider Taxonomy 1981–1987: A Supplement to Brignoli’s A Catalogue of the Araneae described between 1940 and 1981. Manchester University Press, 673 pp.

[B25] Simon E (1894) Histoire naturelle des araignées. Paris 1: 489–760.

[B26] Song D Zhu M Chen J (1999) The Spiders of China. Hebei University of Science and Techology Publishing House, Shijiazhuang, 640 pp.

[B27] Tamura K Stecher G Peterson D Filipski A Kumar S (2013) MEGA6: Molecular Evolutionary Genetics Analysis Version 6.0. Molecular Biology and Evolution 30: 2725–2729. doi: 10.1093/molbev/mst197 24132122 10.1093/molbev/mst197 PMC3840312

[B28] Tanasevitch AV (2010) A revision of the *Erigone* species described by T. Thorell from Burma (Aranei: Linyphiidae). Arthropoda Selecta 19: 103–107.

[B29] Thorell T (1898) Viaggio di Leonardo Fea in Birmania e regioni vicine. LXXX. Secondo saggio sui Ragni birmani. II. Retitelariae et Orbitelariae. Annali del Museo Civico di Storia Naturale di Genova (2) 19: 271–378.

[B30] Tso IM Yoshida H (2000) A new species of the genus *Nesticella* (Araneae: Nesticidae) from Taiwan. Acta Arachnologica, Tokyo 49: 13–16.

[B31] World Spider Catalog (2016) World Spider Catalog. Natural History Museum Bern http://wsc.nmbe.ch version 17.0 [accessed 26 April, 2016]

[B32] Wunderlich J (1986) Spinnenfauna Gestern und Heute: Fossile Spinnen in Bernstein und ihre Heute Lebenden Verwandten. Quelle & Meyer, Wiesbaden, 283 pp.

[B33] Wunderlich J (1995) Beschreibung bisher unbekannter Spinnenarten und -Gattungen aus Malaysia und Indonesien (Arachnida: Araneae: Oonopidae, Tetrablemmidae, Telemidae, Pholcidae, Linyphiidae, Nesticidae, Theridiidae und Dictynidae). Beiträge zur Araneologie 4: 559–579.

[B34] Wunderlich J Song D (1995) Four new spider species of the families Anapidae, Linyphiidae and Nesticidae from a tropical rain forest area of SW-China. Beiträge zur Araneologie 4: 343–351.

[B35] Yaginuma T (1970) Two new species of small nesticid spiders of Japan. Bulletin of the National Museum of Nature and Science, Tokyo 13: 385–394.

[B36] Yaginuma T (1972) Revision of the short-legged nesticid spiders of Japan. Bulletin of the National Museum of Nature and Science, Tokyo 15: 619–622.

[B37] Yin C Peng X Yan H Bao Y Xu X Tang G Zhou Q Liu P (2012) Fauna Hunan: Araneae in Hunan, China. Hunan Science and Technology Press, Changsha, 1590 pp.

[B38] Zhang Y Li S (2013) Ancient lineage, young troglobites: recent colonization of caves by *Nesticella* spiders. BMC Evolutionary Biology 13: 183. doi: 10.1186/1471-2148-13-183 24006950 10.1186/1471-2148-13-183 PMC3766682

